# Safety and immunogenicity of two Tau-targeting active immunotherapies, ACI-35.030 and JACI-35.054, in participants with early Alzheimer's disease: a phase 1b/2a, multicentre, double-blind, randomised, placebo-controlled study

**DOI:** 10.1016/j.ebiom.2025.105940

**Published:** 2025-09-18

**Authors:** Olivier Sol, Julien Mermoud, Merja Hallikainen, Sudhir Kurl, Juha Rinne, Paul Dautzenberg, Everard G.B. Vijverberg, Catherine Mummery, Anne Börjesson-Hanson, Michael Jonsson, Craig Ritchie, Catherine Pennington, Marija Vukicevic, Eva Gollwitzer, Emma Fiorini, David T. Hickman, Valérie Hliva, Julian Gray, Viktoriia Gerasymchuk, Jonathan Wagg, Nicolas Fournier, Bénédicte Lê, Iva Kezic, Lennert Steukers, Gallen Triana-Baltzer, Clara Theunis, Johannes Streffer, Marie Kosco-Vilbois, Andrea Pfeifer, Philip Scheltens

**Affiliations:** a AC Immune SA, Lausanne, Switzerland; b University of Eastern Finland, School of Medicine, Kuopio, Finland; c Turku PET Centre, University of Turku and Turku University Hospital, Turku, Finland; d Clinical Research Services Turku (CRST), Turku, Finland; e Brain Research Center, Center Den Bosch, Den Bosch, Netherlands; f Amsterdam UMC, Amsterdam, Netherlands; g University College London, London, UK; h Karolinska Institute, Stockholm, Sweden; i Karolinska University Hospital, Stockholm, Sweden; j Sahlgrenska University Hospital, Sahlgrenska Academy, University of Gothenburg, Gothenburg, Sweden; k University of Edinburgh, Edinburgh, UK; l Johnson & Johnson Innovative Medicine, Beerse, Belgium; m Johnson & Johnson Innovative Medicine, San Diego, CA, USA; n Department of Biomedical Sciences, University of Antwerp, Antwerp, Belgium

**Keywords:** Alzheimer's disease, Active immunotherapy, Vaccine, Tau, phosphoTau, Paired helical filament, Immunogenicity

## Abstract

**Background:**

Active immunotherapies targeting C-terminal phosphorylated Tau species have the potential to efficiently reduce Tau spreading. ACI-35.030, a SupraAntigen®-based liposome, and JACI-35.054, a CRM197 carrier-protein conjugate, share the same immunogenic pTau sequence and were assessed to determine the best formulation for preferential activation of B cells specific to pathological Tau forms.

**Methods:**

Individuals with early AD were enrolled in this randomised, double-blind, placebo-controlled study (NCT04445831). Participants were randomly assigned to 2 cohorts (ACI-35.030 at 300, 900, 1800 μg or placebo; and JACI-35.054 at 15, 60 μg or placebo) and received 4 intramuscular injections over 48 weeks, followed up to week 74. Participants receiving at least one dose of study drug were included in the intention-to-treat analysis. The primary objectives were safety, tolerability and immunogenicity.

**Findings:**

Among the 57 randomised participants, 41 were assigned to the ACI-35.030 cohort and 16 to the JACI-35.054 cohort. The most frequent adverse events observed consistently in both active groups were injection site reactions (16.7%–100%) and headaches (16.7%–50%). No relevant MRI findings and no adverse events leading to study discontinuation were reported. ACI-35.030 required only one injection to induce anti-pTau IgG titres in all participants and consistently boosted levels with subsequent immunisations. JACI-35.054 raised a strong but more heterogenous anti-pTau IgG response and required multiple administrations to reach consistent titres in all participants. ACI-35.030 induced a robust polyclonal antibody response binding enriched PHF from AD brain tissue while concurrently sparing the response to non-phosphorylated Tau. A post-hoc statistical analysis revealed statistically significant differences between some randomised actively treated groups and the pooled placebo group on plasma pTau217 and brain-derived Tau changes from baseline.

**Interpretation:**

ACI-35.030 and JACI-35.054 were well tolerated. ACI-35.030 induced a more rapid and sustained antibody response selective to p-Tau species with evidence of altering AD-related plasma biomarkers and was selected for testing in the ongoing Phase 2b trial.

**Funding:**

AC Immune SA and 10.13039/100005565Johnson & Johnson Innovative Medicine.

**Research in context**

**Evidence before this study**

ACI-35.030 and JACI-35.054 are modifications of a first-generation active immunotherapy formulation, ACI-35, comprised of the same phosphorylated Tau peptide antigen and monosphosphoryl lipid A (MPLA) adjuvant embedded in a liposome. ACI-35 was investigated in a Phase 1 clinical trial (Study ACI-35-1201) where it was found to be safe and well tolerated. Although antibody responses were generated at all tested doses after the first immunisation, sustained anti-pTau antibody responses were not observed with subsequent injections of this first-generation formulation (data on file). ACI-35.030 and JACI-35.054 were, therefore, designed to retain the good safety profile of ACI-35 while improving immunogenicity, notably to maintain specific anti-pTau titres over time. A search in clinicaltrials.gov and PubMed using the following key words, “Tau vaccine”, “Tau active immunotherapy”, and “Alzheimer” was performed on 11 February 2025. Apart from ACI-35.030 and JACI-35.054 that were assessed in our study, AADVac1 was the only other anti-Tau active immunotherapy with published results from clinical trials. There was no evidence of any completed late-stage trial with anti-Tau-targeting immunotherapies or antisense oligonucleotides in Alzheimer's disease or with other tauopathies, which demonstrate that anti-Tau therapies are still currently in an early stage of clinical development.

**Added value of this study**

ACI-35.030 and JACI-35.054 are active immunotherapies being developed for the treatment of Alzheimer's Disease (AD). ACI-35.030 and JACI-35.054 are designed to stimulate the immune system of individuals with AD to produce antibodies against specific phosphorylated Tau species. Antibodies induced by treatment with ACI-35.030 and JACI-35.054 are aimed to inhibit tau spreading by targeting and clearing extracellular pathological tau species and consequently preventing their uptake by, and the induction of intracellular tau aggregation in, nearby brain healthy neurons. In this study, both tested active immunotherapies induced robust antibody responses against the immunogen peptide and pathological Tau species. The two compounds were observed to be different in the profile and magnitude of the generated antibody titres. ACI-35.030, derived from the SupraAntigen® liposome–based platform, induced an early and strong polyclonal antibody response that matured toward, and was mainly oriented and maintained against, key pathological forms of Tau. These pathological species are believed to drive Tau pathology in AD. In contrast, JACI-35.054, a CRM197 carrier-protein conjugate, triggered a more progressive and heterogenous antibody response and was less specifically oriented toward pathological Tau species. The safety and tolerability of these two active immunotherapies were good. This study has demonstrated that two different anti-Tau active immunotherapy formulations sharing the same immunogen can induce a differential effect in antibody response in humans. It also provides evidence that anti-Tau active immunotherapies in the clinical setting can generate an antibody response against the pathological form of the endogenous brain Tau (paired helical filament, PHF). Post-hoc analyses performed in the participating individuals showed statistically significant differences in changes from baseline of plasma pTau217 and brain-derived Tau at different timepoints between some of the actively treated groups and a pooled placebo group. These data, generated in a limited number of participants, will need to be replicated in a broader population. To date, only one other anti-Tau active immunotherapy, AADvac1, comprising tau peptide 294–305/4R coupled to Keyhole Limpet Haemocyanin and formulated with aluminium hydroxide, has published Phase 2 clinical data, in 196 participants with mild AD. This active immunotherapy targets the microtubule binding region, the main region involved in Tau aggregation. AADVac1 induced IgG titres against its immunogenic peptide, while no data on antibody titres against PHF were reported. This active immunotherapy was safe and well tolerated and over the 24-month treatment period showed a statistically significant reduction in plasma neurofilament light chain (NfL) level compared to placebo. Post-hoc analyses using a prediction model to identify the subgroup of participants likely to be A+/T+ (n = 91) revealed favourable trends in treatment effects on clinical measures of CDR-SB and ADCS-MCI-ADL.

**Implications of all the available evidence**

Our clinical study demonstrates a potent polyclonal antibody response that matures and is maintained against key pathological forms of Tau in response to active immunotherapy. These results support the continued development of the SupraAntigen® liposome-based active immunotherapy, ACI-35.030 (JNJ-64042056). This study drug is now being assessed in the Phase 2b Reτain trial (NCT06544616) enrolling participants with preclinical AD with the aim to arrest Tau pathology propagation, thus contributing to delaying, or potentially preventing, cognitive decline.

## Introduction

Due to the complexity of the disease, understanding Alzheimer's disease (AD) pathophysiology well enough to find effective, meaningful treatments has only recently witnessed therapeutic breakthroughs with certain amyloid targeting, disease modifying monoclonal antibodies (mAbs).^1^, ^2^, ^3^ These results have provided insights into the cascade hypothesis that begins with amyloid beta (Aβ) accumulation, followed by Tau aggregation, spreading and ultimately neurodegeneration and cognitive impairment.^4^, ^5^, ^6^, ^7^ Diagnostic criteria and staging of AD have been recently updated.^8^ In the Phase 3 CLARITY AD trial with lecanemab, clinical decline measured by using CDR-SB in individuals with early AD was found to be reduced by 27% in the treated group, compared to placebo after 18 months.^1^ Data from the TRAILBLAZER-ALZ 2 trial with donanemab, also conducted in an early AD population, significantly lowered amyloid plaque burden, as measured with PET-scan, and slowed clinical progression especially in participants with low/medium brain Tau burden.^2^ While these anti-amyloid mAbs are currently approved in several countries, it still leaves a continued unmet need for new treatments to further prevent or slow disease progression, notably in individuals with preclinical AD.^9^, ^10^, ^11^ In AD, cognitive decline correlates with the burden of pathological Tau inclusions, species that are prone to seeded aggregation and extracellular spreading.^12^^,^^13^ Aberrant hyperphosphorylation of Tau results in aggregation into PHF, the major component of neurofibrillary tangles (NFT). More precisely, the spread of NFTs across interconnected brain regions correlates with cognitive decline and disease progression.^14^ As pathological Tau species are released from neurons in which Tau aggregation has been initiated, immunotherapies using antibodies specifically binding in the interstitial fluid to pathological forms of Tau, that are closely related to PHF, may offer promise as treatments by impeding the spread of Tau pathology.^15^^,^^16^ While various Tau-targeting mAb-based therapies are currently being studied in early stages of AD, active immunotherapies are highly attractive to pursue, as immunisation offers several distinct advantages. Active immunotherapies instruct the immune system to defend itself against pathological immunogens. Post dosing, a natural and long-lasting polyclonal antibody response against a pathological target is generated. The maintenance of the antibody response can be achieved with less frequent doses as compared to mAbs, and as the protective antibodies are generated by the immune system, anti-drug antibodies, commonly observed with mAbs, are absent.^17^ Yet the most highly differentiating feature is the mechanism to instruct/inform, via an active process of affinity maturation, the evolution of the antibodies toward the endogenous pathological form driving disease within each person. This maturation enhances the ability to protect against the disease caused by the pathological Tau species spreading within an individual. Thus, in order to achieve a protective antibody response to the immunising peptide (i.e., pTau) as well as to promote evolution to the endogenous pathological species (represented by PHF enriched from AD brain–ePHF) within an individual with AD, the design of active immunotherapy required considerable forethought. Although safety, immunogenicity and efficacy can be established using laboratory animals, the ultimate goal is to observe these features in humans who are part of a vulnerable population.^18^ Therefore, in this trial, two active immunotherapy formulations, ACI-35.030 and JACI-35.054, were evaluated for safety, tolerability, and immunogenicity. Both contained the same phospho-Tau peptide from the C-terminal region of the Tau protein (Tau393-408 [pS396/pS404]), considered a relevant phosphorylated region related to AD pathology and abundantly present on PHF.^19^, ^20^, ^21^, ^22^ The formulations differed in the structural element used to present the phospho-Tau peptide to the immune system and the selection of adjuvants, key drivers of successful active immunotherapies. Therefore, different doses of the two formulations were individually assessed in non-human primates, in order to define an optimal range for each immunotherapy to be evaluated in humans. According to regulatory authority guidelines, the initial dose in humans was low and less immunogenic, followed by increasing doses, up to a maximal dose allowed by the format of the carrier. Here we present safety and immunogenicity data of both active immunotherapies along with the observed effects on fluid biomarkers, imaging parameters and clinical endpoints across all participants.

## Methods

### Formulations of ACI-35.030 and JACI-35.054 active immunotherapies and placebo

The liposome-based active immunotherapy formulation, ACI-35.030, presents a phosphorylated Tau (pTau) peptide, Tau393-408 (phosphorylated at Serine 396 and 404), double-lipidated to enable insertion at both the N-and C-terminal ends through Lys(palmitoyl). In addition, universal T-cell epitopes derived from PADRE (Pan DR-binding epitope; universal synthetic T-cell peptide) and Tetanus toxin are encapsulated or associated with the liposome while the two adjuvants, MPLA (3D-(6-acyl)-PHAD®) and CpG7909-cholesterol, are incorporated onto the liposomal surface. The carrier-based active immunotherapy formulation, JACI-35.054, presents the same immunogenic pTau peptide but without lipidation, as it is instead covalently linked to the common carrier protein, CRM197, then mixed with aluminium hydroxide and CpG7909 as adjuvants prior to administration. Both active immunotherapy approaches (liposome versus carrier-based) were chosen as they are well-known to enhance the immunogenicity to antigenic peptides.^23^, ^24^, ^25^, ^26^ Studies in nonclinical species (i.e., adult wildtype mice and nonhuman primates) were performed in order to determine the optimal formulation and dose level to achieve the best anti-pTau IgG response elicited by each formulation. The dose range of the final formulations used in this trial was determined from these nonclinical data. The appearance of the two active immunotherapy formulations and of the placebo (phosphate-buffered saline (PBS) solution) differed. To prevent unblinding of the patient and any blinded site staff, dedicated unblinded persons at the site pharmacy level were in charge of the preparation of allocated study drug using syringes wrapped in a coloured label.

### Study design

This Phase 1b/2a, multicentre, double-blind, randomised, placebo-controlled study was designed to evaluate the safety, tolerability and immunogenicity of different doses, regimens and combinations of ACI-35.030 and JACI-35.054 in early AD. The Phase 1b part of the study assessed immunogenicity, tolerability and safety, while the Phase 2a part was intended to provide a preliminary assessment of effects on biomarkers. Outpatients took part in the study between 13 August 2019 (first participant first visit) and 5 September 2023 (last participant last visit) in 9 clinical sites in Finland, Netherlands, United Kingdom and Sweden. The study protocol was reviewed and approved by health authorities, respectively by the Medicines Agency (ID KLnro 30/2019) in Finland, by the Medicines and Healthcare products Regulatory Agency (ID CTA 41996/0004/001-0001) in the UK, by the Medical Products Agency (ID 5.1-2020-37457) in Sweden and by the Medicines Evaluation Board Agency under the Ministry of Health, Welfare and Sport (ID NL69383.000.19) in the Netherlands. The study was initially registered in EudraCT (2015-000630-30) and the public registry onderzoekmetmensen.nl on 18 April 2019, and in research summaries pages of the UK Health Research Authority website. To facilitate broader public access, as phase 1 trials were not made publicly available in EudraCT registry, the trial was subsequently registered on ClinicalTrials.gov (NCT04445831) on 22 June 2020.

### Ethics

The study protocol and key study documents were reviewed and approved respectively by the Ethics Committees of the participating countries, respectively the Committee on Medical Research Ethics (ID 29/06.00.01/2019) in Finland, the West of Scotland Research Ethics Committee (ID 19/WS/0056) in the UK, the Ethical Review Authority (ID 2020-01470) in Sweden, and the Central Committee for Research involving Human subjects (ID CCMO19.0508/JvG/cb/69383) in the Netherlands. All study participants and their caregivers provided written informed consent before any study procedures.

### Participants

Participants had to meet the following main inclusion criteria: 50-75 years-old, male or female, diagnosis of Mild Cognitive Impairment (MCI) due to AD or mild AD according to NIA-AA criteria, Clinical Dementia Rating scale (CDR) global score of 0.5 or 1, Mini-Mental State Examination (MMSE) ≥22, abnormal level of CSF amyloid beta (Aβ) 42 consistent with AD pathology as per laboratory threshold, no intake of marketed treatment for AD or on stable dose of acetylcholinesterase inhibitor and/or memantine for at least 3 months prior to baseline. No particular guidance was given to study sites for the selection of participants according to sex, gender, race and ethnicity and these parameters were collected according to usual local site practice. The main exclusionary selection criteria were recent participation in previous clinical trials for AD and/or for neurological disorders, positive anti-nuclear antibodies (ANA) titres at a dilution ≥1/160, current or past history of autoimmune disease, use of immunosuppressive drugs or systemic steroids, any significant medical conditions which could confound the assessment of safety or immunogenicity. Details on eligibility criteria are listed in the protocol.

### Randomisation and masking

A randomisation list enabling study treatment allocation was computed by an Interactive Response Technology system using individual medication codes provided by the investigational drug product supplier. The study medication was labelled with the corresponding medication number. The blinded site staff used the IRT system to randomise eligible participants to a study subcohort and to assign study treatment. The randomisation block size of 4 with an active:placebo ratio of 3:1 was used in each study subcohort. Study participants, site personnel and sponsor were blinded to treatment.

### Procedures

The initial screening period of up to 42 days was followed by a treatment period of 50 weeks with study drug intramuscular administration in the deltoid muscle at weeks 0, 8, 24, and 48. A 24-week follow-up period up to week 74 completed the study. Participants were kept under clinical observation for 24 h after the first injection and for 4 h after the subsequent study drug administrations. A safety assessment by phone call was also performed 48–72 h after each immunisation. In each subcohort, the first dosing of the first 4 participants had to be performed 48–72 h after the safety assessment of the previous participant. Recording of adverse events and of concomitant medications were performed at all study visits. Clinical and neurological examinations and routine laboratory evaluations were performed during on-site visits, as per study protocol. Dose escalation was permitted once all participants in one dose-level subcohort had received the second injection of study drug and after review of interim safety and tolerability data by the independent Data Safety Monitoring Board. ACI-35.030 was sequentially assessed at doses of 300 μg (subcohort 1.1), 900 μg (subcohort 1.2), and 1800 μg (subcohort 1.3), while JACI-35.054 was tested at doses of 15 μg (subcohort 2.1) and 60 μg (subcohort 2.2). Both ACI-35.030 and JACI-35.054 cohorts were conducted independently from each other. Although the ACI-35.030 cohort was started before the JACI-35.054 cohort, the screening periods between cohort 1 (13 August 2019–22 February 2022) and cohort 2 (28 July 2020 and 26 August 2021) were globally overlapping, thus allowing to group participants on placebo for the analyses. The design of cohorts and subcohorts and study schedule are presented in Table 1. During the treatment period, study visits at the centres were organised the days of, and two weeks after each immunisation, and an additional visit was also to be performed at week 36. Visits at weeks 67 and 74 (last study visit) were performed during the follow-up period. During each study visit, blood samples were measured for antibody titres against Tau species and safety measures were assessed. Lumbar punctures were performed at screening, weeks 26 and 50 to assess fluid biomarkers and routine cytology and biochemistry. Brain MRIs were planned at baseline, two weeks after the second and subsequent injections and at week 74. For cognitive and clinical assessments, the Repeatable Battery for the Assessment of Neuropsychological Status (RBANS), Clinical Dementia Rating scale - Sum of Boxes (CDR-SB) and Columbia-Suicide Severity Rating Scale (C-SSRS) were performed at screening, weeks 0, 26, 50, and 74. The Neuropsychiatric Inventory (NPI) was performed at the same timepoints as the other scales from week 0 onwards. During the study, additional study visits were conducted at weeks 15, 20, 31, and 42 to assess more closely the kinetics of plasma biomarkers and antibody titres. Of note, due to the COVID-19 pandemic and travel restrictions, immunisations at week 24 were not performed in 7 of 8 participants from Finland in subcohort 1.1.

**Table 1:** Study cohort profile and administration schedule.

Cohort	Subcohort	IP	Dose (μg)		Screening	Treatment													Follow-up	
				Visit	S	V1	V2	V3	V4	V4.1	V4.2	V5	V6	V6.1	V7	V7.1	V8	V9	V10	V11
				Week	−6 to ∼0	0	2	8	10	15	20	24	26	31	36	42	48	50	67	74
				N																
1	1.1	ACE-35.030	300	6	CSF	↑		↑				↑	CSF				↑	CSF		
		Placebo	–	2	CSF	↑		↑				↑	CSF				↑	CSF		
	1.2	ACE-35.030	900	19	CSF	↑		↑				↑	CSF				↑	CSF		
		Placebo	–	6	CSF	↑		↑				↑	CSF				↑	CSF		
	1.3	ACE-35.030	1800	6	CSF	↑		↑				↑	CSF				↑	CSF		
		Placebo	–	2	CSF	↑		↑				↑	CSF				↑	CSF		
2	2.1	JACI-35.054	15	6	CSF	↑		↑				↑	CSF				↑	CSF		
		Placebo	–	2	CSF	↑		↑				↑	CSF				↑	CSF		
	2.2	JACI-35.054	60	6	CSF	↑		↑				↑	CSF				↑	CSF		
		Placebo	–	2	CSF	↑		↑				↑	CSF				↑	CSF		

↑: IP Injection. CSF: Cerebrospinal Fluid collection. IP: Investigational Product. N: number of study participants. S: screening. V: visit. Cohort 1 placebo group: N = 10. Cohort 2 placebo group: N = 4. Pooled placebo group (cohort 1 placebo + cohort 2 placebo): N = 14. Blood was collected at each visit. Safety phone calls (not depicted on this table) took place 48–72 h after each IP administration. During the study, additional study visits V4.1, V4.2, V6.1, and V7.1 were conducted at weeks 15, 20, 31, and 42, respectively, to assess more closely the kinetics of plasma biomarkers and antibody titres.

### Study objectives and endpoints

The primary objectives were to assess the safety and tolerability of the study active immunotherapies and their immunogenicity to generate an anti-pTau and anti-ePHF IgG responses. The primary endpoints for the safety and tolerability were respectively the collection of adverse events, immediate and delayed reactogenicity, suicidal ideation, behaviour, cognitive and functional assessments also intended to assess safety, vital signs, MRI imaging, electrocardiogram, routine haematology and biochemistry, measure of autoimmune antibodies including anti-dsDNA antibodies in blood; inflammatory markers in blood and CSF. The primary endpoint for immunogenicity was the measure of anti-pTau IgG titres in serum. The secondary outcome measures were intended to assess additional immunogenicity of the active immunotherapies, i.e., anti-Tau IgG and IgM responses. The planned secondary endpoints were the measures of anti-Tau IgG, anti-pTau and anti-Tau IgM titres in serum and the determination of the IgG response profile by avidity testing. The latter was only partially performed due to technical reasons and therefore no conclusion could be drawn on this dataset. Exploratory objectives were to evaluate effects on AD-related fluid biomarkers, inflammatory cytokines, additional immune response components (i.e., antibodies against other study treatment components, namely T50, a universal T-cell epitope contained in ACI-35.030; and CRM, the carrier protein used in JACI-35.054; functional capacity of active immunotherapy-induced antibodies) and behaviour, cognitive and functional performance.

### Statistical analysis

Statistical analyses were performed by ICON, except dedicated interim analyses performed in collaboration with Johnson & Johnson Innovative Medicine and a post-hoc statistical analysis performed by AC Immune. All analyses and tabulations of data were performed using SAS® version 9.4 or higher. The post-hoc analyses and all longitudinal plots were generated by AC Immune using the R-statistical programming language (Version 4.3.3). Missing data were excluded from the analyses and no missing data imputation was carried out. Descriptive summaries were tabulated by treatment group, with placebo groups separated by cohort (placebo in cohorts 1 and 2) or pooled across cohorts (Pooled placebo). Categorical data were presented using counts and percentages, with the number of participants in each category as the denominator for percentages. Continuous data were summarised using descriptive statistics. The enrolled population comprised all participants who signed the informed consent form. Baseline characteristics and primary, secondary, and exploratory endpoints were presented using the Intention-to-Treat (ITT)/safety population which consisted of all randomised participants receiving at least one dose of study drug. The Per Protocol (PP) population included participants from the ITT population without any important protocol deviations that could have significantly affected the completeness, accuracy, and/or reliability of the study data. Adverse events were coded using the Medical Dictionary for Regulatory Activities (MedDRA) version 26.1. The antibody response data for primary, secondary, and exploratory endpoints were presented using descriptive statistics of absolute values and changes from baseline. Predefined semi-blinded interim analyses of the antibody response and of safety/tolerability were performed in randomised participants at specific timepoints. The semi-blinded procedure (recoding of participant identifiers) enabled individual interim data review per treatment group without unblinding a participant's individual identifier. The number of participants in each subcohort (n = 8; 6 on active treatment and 2 on placebo) was considered appropriate to evaluate the preliminary antibody response, safety and tolerability. Up to 16 additional participants could be randomised in subcohort(s) showing a more robust antibody response to get a better appreciation of immunogenicity and safety. The decision was taken to expose additional participants to ACI-35.030 900 μg (n = 19, ITT) within the expanded sub-cohort 1.2 (n = 25, ITT). More details on statistical analysis methods can be found in the Supplementary Section.

### Role of the funding source

The sponsor of the study, AC Immune SA, participated in study design, study conduct, data collection, analysis and interpretation, clinical study report and writing, review, and approval of the manuscript. All authors had full access to the data, participated in the development and review of the manuscript, took full responsibility for the content and approved the manuscript for submission for publication. Johnson & Johnson Innovative Medicine provided support notably for interim data analyses, generating data on some fluid biomarkers and affiliated coauthors participating in the data interpretation and manuscript review and approval.

## Results

**Fig. 1:**
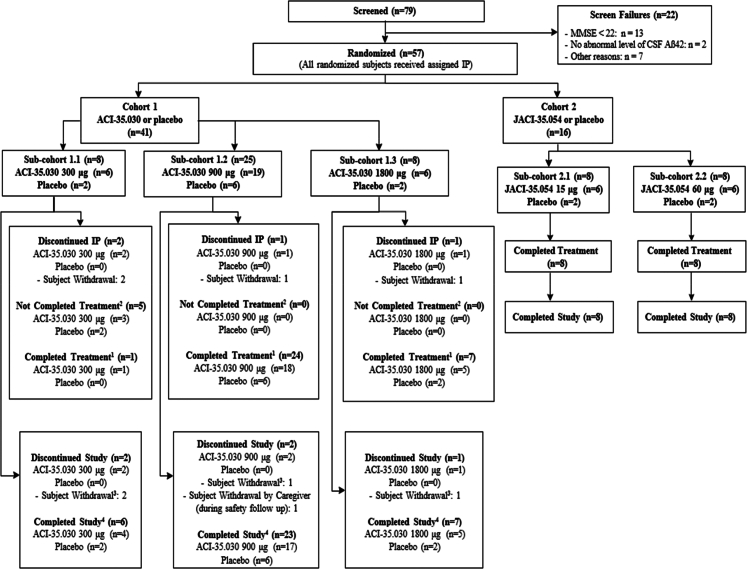
**Trial profile.**^1^A participant was considered to have completed the study treatment if he or she had reached the end of the treatment period (Visit 9 Week 50) and had received all planned injections (4 successive IM immunisations with IP). ^2^A participant was considered to have not completed the study treatment if he or she had reached the end of the treatment period (Visit 9 Week 50) and had not received all planned IP injections. ^3^Participants discontinuing study due to participant withdrawal are the same participants who discontinued IP. ^4^A participant was considered to have completed the study if he or she had completed the safety follow-up period. eCRF = electronic case report form; IM = intramuscular; IP = investigational product.

**Fig. 2:**
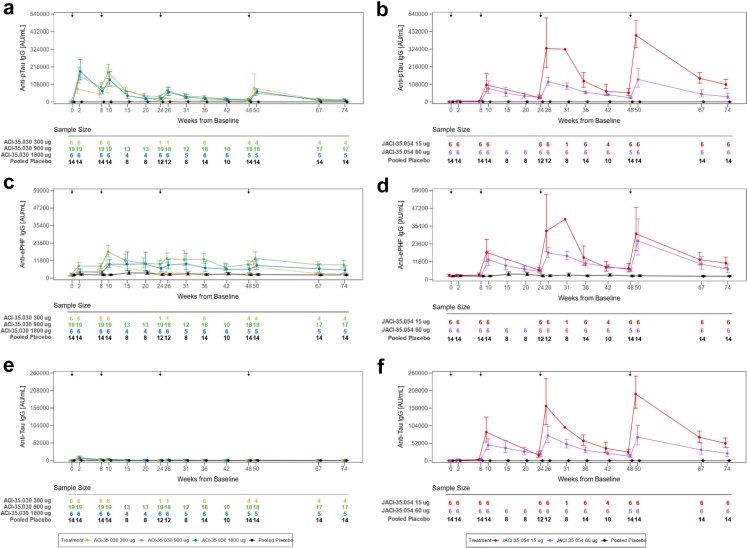
**Geometric mean of the anti-pTau, anti-ePHF, and anti-Tau IgG responses with active immunotherapies.** Plots of the geometric mean of anti-pTau (**a, b**), anti-ePHF (**c, d**), and anti-Tau IgG (**e, f**) responses versus nominal study visit time (in weeks) for active immunotherapy with ACI-35.030 (**a, c, e**) or JACI-35.054 (**b, d, f**). Error bars denote standard errors of the geometric means. Geometric means and standard errors are plotted by study arm and nominal study visit time. Sample sizes for plotted geometric means and standard errors are tabulated below each graph. The four vertical arrows at the top of each graph denote nominal study visit times for administration of ACI-35.030 or JACI-35.054.

**Fig. 3:**
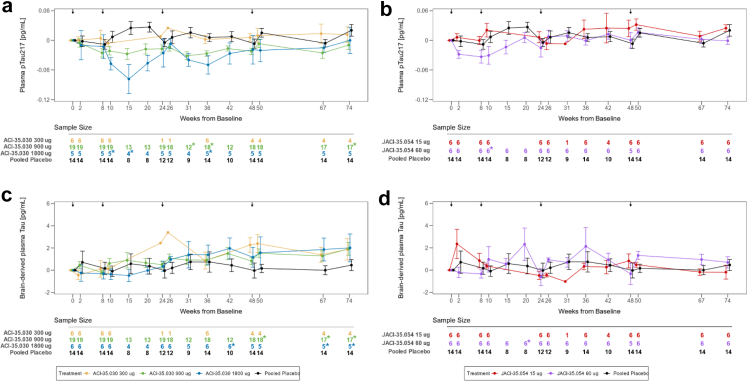
**Arithmetic mean change from baseline of plasma pTau217 and plasma brain derived (BD) Tau with active immunotherapy.** Plots of the mean change from baseline of plasma pTau217 (**a, b**) and plasma brain derived (BD) Tau (**c, d**) concentrations (pg/mL) versus nominal study visit time (in weeks) for active immunotherapy with ACI-35.030 (**a, c**) or JACI-35.054 (**b, d**). Error bars denote standard errors of the means. Means and standard errors are plotted by study arm and nominal study visit time. Sample sizes for plotted means and standard errors are tabulated below each graph. Stars denote a statistically significant (p < 0.05) difference from placebo group based on the post-hoc statistical analysis (Linear Mixed Model analysis, contrasts using Satterthwaite's degrees of freedom; see Supplementary Material for full details). The four vertical arrows at the top of each graph denote nominal study visit times for administration of ACI-35.030 or JACI-35.054.

Participants were recruited between 13 August 2019 and 22 February 2022. Among the 79 screened participants, 57 were randomised, 41 participants (male/female: 21/20; mean age: 67.1 (±5.49)) in cohort 1 received either ACI-35.030 or placebo and 16 participants (male/female: 6/10; mean age: 65.6 (±5.73)) in cohort 2 received either JACI-35.054 or placebo. Details on the trial profile are provided in Fig. 1. All randomised participants with early AD received at least one dose of study drug and were included in the Safety/ITT population. In cohort 1, 37/41 (90.2%) participants entered the follow-up period, and 32/41 (78%) were included in the PP population. In cohort 2, all 16 participants entered the follow-up period, and 15/16 (93.8%) were included in the PP population. All participants were white. Mean baseline MMSE total scores were respectively 26.3 (±2.29) and 26.3 (±2.93) in cohorts 1 and 2. Most participants had a CDR-global score of 0.5 at baseline, 36/41 (87.8%) and 14/16 (87.5%) in cohorts 1 and 2, respectively. Standard of care treatment for AD, i.e., acetylcholinesterase inhibitors and/or memantine, was prescribed in 32/41 (78%) participants in cohort 1 and in 12/16 (75%) in cohort 2. Comprehensive demographics and baseline characteristics are shown in Table 2. Overall, in cohort 1, 2/41 (4.9%) participants received only two injections, 7/41 (17.1%) received three injections and 32/41 (78.0%) participants received the planned four injections of study drug. All participants received four injections in cohort 2. No death was reported, and no adverse event (AE) led to discontinuation of study treatment or of the study. There were no notable differences in the incidence of treatment-emergent adverse events (TEAEs) in ACI-35.030 active treatment groups, i.e., 6/6 (100%) participants in 300 μg and 1800 μg cohorts, 17/19 (89.5%) participants in 900 μg subcohort, and 8/10 (80.0%) participants in placebo subcohort. TEAEs were mostly mild or moderate in severity. Nine serious adverse events (SAEs) were reported in six participants (2/6 participants (33.3%) in ACI-35.030 300 μg cohort, 2/19 (10.5%) in 900 μg cohort, and 2/6 (33.3%) in 1800 μg cohort), as shown in Table 3. There was no pattern to the SAEs observed, and all were considered as unlikely related to the study treatment, except for the SAEs of injection site rash and dizziness reported in one participant in the ACI-35.030 900 μg treatment group, which were considered as probably and possibly related to study treatment, respectively. As shown in Table 4, the most frequent adverse events which occurred more commonly on active treatment compared to placebo were injection site reactions (ISRs). They were reported in cohort 1 on one or more occasions in 2/6 participants (33.3%) at the 300 μg dose, 14/19 (73.7%) at the 900 μg dose and 6/6 (100%) at the 1800 μg dose, with no episode reported with placebo. ISRs were mild to moderate in severity except in two cases in which the reactions were rated as severe due to the magnitude of the area of redness; in all cases the reactions were self-limiting. Headache was more commonly observed at the 900 and 1800 μg dose levels (4/19 participants (21.1%) and 3/6 participants (50%), respectively), compared to the 300 μg dose and concurrent placebo subcohorts, in which no episode occurred. In cohort 2, all participants on active and placebo treatment reported TEAEs that were mostly mild or moderate in severity. One SAE was observed on placebo treatment. The commonest adverse events occurring in a dose dependent manner in the active treatment arms and not on placebo were ISRs, being reported in 1/6 participants (16.7%) in the 15 μg dose group and in 2/6 participants (33.3%) in the 60 μg dose group. There were no clinically meaningful changes in vital signs, ECGs, haematology, biochemistry, and urinalysis parameters during the study. Elevations of anti-dsDNA antibody titres (>15 IU/mL) were observed in 2/6 (33.3%), 10/19 (52.6%), and 2/6 (33.3%) participants treated with ACI-35.030 300 μg, 900 μg, and 1800 μg, respectively, when measured using a standard enzyme-linked immunoassay. When measured using a more specific Farr radioimmunoassay, the titres were slightly and transiently above the normal range in two participants treated with ACI-35.030 900 μg during the follow-up period, without any related symptoms. No ANA titres were above the 1:160 threshold, except transiently at week 67 in one participant, in whom the Farr assay was normal. No clinically relevant MRI changes or new lesions on MRI scans were observed. In cohort 1, two asymptomatic incidental microhaemorrhages (ARIA-H) were detected in one participant treated with ACI-35.030 900 μg at week 74, while in cohort 2, two new asymptomatic microhaemorrhages were noted in two participants, i.e., one treated with JACI-35.054 60 μg at week 50 and the second one on placebo at week 74. The lists of SAEs and of most frequent TEAEs reported in ≥20% of participants per group are reported in Tables 3 and 4. T-cell activation and inflammatory cytokines were not measured in the absence of related safety concerns. Overall, no clinically relevant safety and tolerability observations were reported at any doses in participants exposed to the two active immunotherapies. Immunogenicity of the active immunotherapies was assessed using three parameters: the antibody response to the immunogen, i.e., anti-pTau peptide IgG titres, the development of antibodies to brain-derived pathological Tau, i.e., anti-ePHF IgG titres, and the response to the non-phosphorylated version of the immunogen, here designated as anti-Tau IgG titres. Geometric means of the anti-pTau, anti-ePHF, and anti-Tau IgG responses with the two active immunotherapies are presented in Fig. 2. Tabulations of corresponding fold-changes from baseline for anti-pTau (Table S1), anti-ePHF (Table S2), and anti-Tau IgG (Table S3), and responder rates (based on analytical thresholds for the respective assays) for the same parameters (Tables S4–S6, respectively) are provided in the Supplementary Material. For the immunogen, anti-pTau IgG responses were observed after the first injection of ACI-35.030. All participants at all dose-levels were considered anti-pTau IgG responders at 2 weeks post treatment, and these rates were maintained between 94 and 100% until week 74 in the two high-dose cohorts. The transient decrease in responder rates observed with the low 300 μg dose at weeks 36 and 48 is likely explained by the absence of study drug administration at week 24 in 5/6 Finnish participants due to the Covid-19 pandemic. With JACI-35.054, a 100% anti-pTau IgG response was observed after the second injection at week 10 with both dose levels and was maintained until study end. Four participants from the pooled placebo group had anti-pTau IgG titres slightly above the responder threshold at sporadic timepoints due to some variability inherent to the assay. The majority of participants (66.7%–73.7%) generated an anti-ePHF IgG response against brain-derived pathological Tau after the first administration of ACI-35.030, observed at all doses at week 2. With additional treatments, the anti-ePHF IgG levels increased with responder rates ranging from 25% to 100% across the different dose-levels. The anti-ePHF IgG titres increased in all active groups after each injection with a slower rate of decline between dosing intervals than was observed with anti-pTau IgG titres. In particular, the responder rate at the mid-dose of 900 μg ranged from 70.6% to 94.7% at any timepoints until study end. For JACI-35.054, two administrations were required to observe a robust responder rate of anti-ePHF IgG titres ranging from 50% to 100% up to study end at both doses. Anti-ePHF IgG titres increased after each following administration in both JACI-35.054 treatment groups, with a subsequent consistent rate of decline after each consecutive immunisation. No apparent dose effect was observed between the two doses. No rise of anti-ePHF IgG titres was observed with placebo. Overall, the 900 μg dose of ACI-35.030 demonstrated a stronger and earlier (73.7% responder rate at week 2), more stable and sustained capacity (responder rate was constantly above 70% at all time points) to evolve the antibody response over time toward the endogenous pathological Tau (ePHF) present in the brain of participants with AD. A key element to compare and appreciate the value of immunotherapies was to also evaluate the level of the antibody response generated to the non-pathological form of Tau. While such anti-Tau IgG were observed with ACI-35.030 at week 2 in all active dose groups, these titres quickly decreased and were not maintained, nor boosted, with additional treatments in any ACI-35.030 treatment group. In contrast, with JACI-35.054, such anti-Tau IgG were measured after the second treatment at week 10 and found to have increased after each subsequent administration at either dose level, especially the low dose. Furthermore, the anti-Tau IgG responder rates were maintained at 100% in both JACI-35.054 doses at all measured timepoints from week 10 to 74. No increase of anti-Tau IgG titres and no responders were observed with placebo. Taken together, these results differentiate the two active immunotherapies, as ACI-35.030 demonstrated the ability to evolve the antibody response away from the non-phosphorylated Tau and to maintain the preference of the antibody repertoire toward binding the phosphorylated Tau species. Another differentiating feature between these active immunotherapies was the induction of anti-pTau IgM titres. While anti-pTau IgM responses were observed after the first injection of ACI-35.030, no noteworthy absolute values over time in anti-pTau IgM titres were observed with JACI-35.054 throughout the study (Supplementary Material, Figures S1 and S2, respectively). The profile of the anti-pTau IgM titres post ACI-35.030 were consistent, demonstrating a slight increase after each subsequent injection in the active treatment groups, yet an overall decrease in IgM titres over the study. The antibody response against other study drug components, specifically CRM and T50, can be found in the Supplementary Material (Figures S3 and S4, respectively). A panel of exploratory fluid biomarkers (plasma and CSF biomarkers), clinical assessments, as well as volumetric MRI analyses were performed, although the study was not powered to detect statistical changes of the different treatment groups or placebo (Supplementary Material, Figures S5–S20, inclusively for plasma fluid biomarkers; Figures S21–S40, inclusively for CSF fluid biomarkers; Figures S41–S50, Table S7 for C-SSRS, inclusively for clinical assessments and Figures S51–S56, inclusively for volumetric MRI analyses). Of these exploratory endpoints, plasma levels of brain-derived (BD)-Tau, a recently identified biomarker that selectively binds to CNS tau isoforms, and pTau217, which has demonstrated specific detection of AD-associated amyloid and tau pathology, provided further data that differentiated the two active immunotherapies (Fig. 3).^27^, ^28^, ^29^ A post-hoc analysis showed that the change from baseline of BD-Tau plasma levels with ACI-35.030 treatment, when compared to the corresponding changes from baseline for the pooled placebo group, reached nominal significance at weeks 50, 67, and 74 for the 900 μg dose and at weeks 42, 67, and 74 for the 1800 μg dose (Fig. 3 and Table 5). In contrast, treatment with JACI-35.054 compared to placebo reached significance only at the higher dose and only at one time point, i.e., week 20. For plasma pTau217, post-hoc analysis of the change from baseline with ACI-35.030 treatment, when compared to the corresponding change from baseline for the pooled placebo group, was nominally significant at weeks 31, 36, and 74 for the 900 μg dose and 10, 15, and 36 for the 1800 μg dose (Fig. 3 and Table 6). Again, in contrast, treatment with JACI-35.054 reached significance for a decrease in plasma pTau217 only at the higher dose and at only at one time point, i.e., week 10. For the other plasma biomarkers (i.e., pTau181, Amyloid-beta 1-42, 1-40 and 1-42/1-40 ratio, NfL, GFAP, YKL-40), as well as CSF biomarkers (i.e., pTau181, pTau217, Tau, Amyloid-beta 1-42, 1-40 and 1-42/1-40 ratio, NfL, GFAP, YKL-40, neurogranin), no consistent differences were observed with ACI-35.030 and JACI-35.054, as compared to placebo, except for a dose-independent increase of plasma pTau181 observed with the latter (Supplemental Material, Figures S5 and S6). A higher rate of whole brain volume loss and ventricular volume increase compared to placebo was observed at the 1800 μg dose of ACI-35.030 (Supplemental Material, Figures S51 and S55, respectively). Numerically greater hippocampal volume loss compared to placebo was observed only at 900 μg of ACI-35.030 and 60 μg dose of JACI-35.054 but with error bars overlapping with those on placebo (Supplemental Material, Figures S53 and S54, respectively). Given the low power for these endpoints, no conclusions regarding effects on brain volume can be drawn. As anticipated, based on the limited number of participants, the clinical exploratory endpoints did not reveal particular differences in CDR-SB, CDR global score, RBANS or NPI scores across the different study groups (Supplemental Material, Figures S41 and S42 for CDR-SB; Figures S43 and S44 for CDR global score; Figures S45–S48 for RBANS; and Figures S49 and S50 for NPI). Likewise, no notable differences were evident in the C-SSRS, specifically in the frequency of suicidal ideation and whether or not subjects exhibited suicidal behaviour across study groups (Supplemental Material, Table S7).

**Table 2:** Demographic and baseline characteristics (intention-to-treat population).

Cohort 1 with ACI-35.030					
Characteristic	ACI-35.030 300 μg (N = 6)	ACI-35.030 900 μg (N = 19)	ACI-35.030 1800 μg (N = 6)	Cohort 1 placebo (N = 10)	Total (N = 41)
Sex [n (%)]					
Female	4 (66.7)	9 (47.4)	2 (33.3)	5 (50.0)	20 (48.8)
Male	2 (33.3)	10 (52.6)	4 (66.7)	5 (50.0)	21 (51.2)
Race [n (%)]					
White	6 (100)	19 (100)	6 (100)	10 (100)	41 (100)
Age (years)					
n	6	19	6	10	41
Mean (SD)	65.5 (5.01)	68.0 (6.29)	64.7 (4.63)	67.7 (4.60)	67.1 (5.49)
Median (min, max)	64.5 (61, 75)	71.0 (51, 75)	66.5 (56, 68)	69.0 (60, 75)	67.0 (51, 75)
Q1, Q3	62.0, 66.0	63.0, 73.0	63.0, 68.0	66.0, 70.0	63.0, 71.0
BMI (kg/m^2^)					
n	6	19	6	10	41
Mean (SD)	26.4 (3.08)	24.6 (3.46)	27.8 (7.08)	25.1 (2.25)	25.5 (3.91)
Median (min, max)	26.8 (22, 30)	24.3 (19, 35)	25.8 (22, 40)	25.0 (22, 30)	24.7 (19, 40)
Q1, Q3	24.0, 28.7	22.1, 26.1	22.3, 31.1	24.2, 26.0	23.1, 26.8
Time since initial diagnosis of AD (years)					
n	6	19	6	10	41
Mean (SD)	1.2 (0.98)	1.2 (0.71)	2.0 (1.10)	1.9 (2.42)	1.5 (1.40)
Median (min, max)	1.5 (0, 2)	1.0 (0, 3)	2.0 (1, 4)	1.0 (0, 8)	1.0 (0, 8)
Q1, Q3	0.0, 2.0	1.0, 2.0	1.0, 2.0	1.0, 2.0	1.0, 2.0
MMSE^b^					
n	6	19	6	10	41
Mean (SD)	27.5 (0.84)	26.1 (2.02)	26.0 (2.68)	26.1 (3.11)	26.3 (2.29)
Median (min, max)	27.0 (27, 29)	26.0 (22, 29)	26.0 (23, 29)	26.0 (22, 30)	27.0 (22, 30)
Q1, Q3	27.0, 28.0	25.0, 28.0	23.0, 29.0	23.0, 29.0	25.0, 28.0
CDR baseline global score [n (%)]					
0.5	0	17 (89.5)	5 (83.3)	7 (70.0)	29 (70.7)
1	0	2 (10.5)	1 (16.7)	1 (10.0)	4 (9.8)
Missing^a^	6 (100)	0	0	2 (20.0)	8 (19.5)
Number (%) of participants with any prior concomitant medications [n (%)]					
Acetylcholinesterase inhibitors	5 (83.3)	9 (47.4)	3 (50.0)	5 (50.0)	22 (53.7)
Memantine	0	1 (5.3)	1 (16.7)	1 (10.0)	3 (7.3)
Acetylcholinesterase inhibitors and memantine	0	4 (21.1)	1 (16.7)	2 (20.0)	7 (17.1)
Neither acetylcholinesterase inhibitors nor memantine	1 (16.7)	5 (26.3)	1 (16.7)	2 (20.0)	9 (22.0)
*APOE* genotype at screening					
E2/E3	0	1 (5.3)	1 (16.7)	0	2 (4.9)
E3/E3	2 (33.3)	5 (26.3)	1 (16.7)	3 (30.0)	11 (26.8)
E3/E4	3 (50.0)	8 (42.1)	2 (33.3)	1 (10.0)	14 (34.1)
E4/E4	1 (16.7)	5 (26.3)	1 (16.7)	6 (60.0)	13 (31.7)
Missing	0	0	1 (16.7)	0	1 (2.4)

Cohort 2 with JACI-35.054				
Characteristic	JACI-35.054 15 μg (N = 6)	JACI-35.054 60 μg (N = 6)	Cohort 2 placebo (N = 4)	Total (N = 16)
Sex [n (%)]				
Female	3 (50.0)	3 (50.0)	4 (100)	10 (62.5)
Male	3 (50.0)	3 (50.0)	0	6 (37.5)
Race [n (%)]				
White	6 (100)	6 (100)	4 (100)	16 (100)
Age (years)				
n	6	6	4	16
Mean (SD)	66.7 (6.22)	63.0 (4.77)	68.0 (6.16)	65.6 (5.73)
Median (min, max)	67.0 (56, 73)	62.0 (58, 72)	68.5 (60, 75)	65.0 (56, 75)
Q1, Q3	65.0, 72.0	61.1, 63.0	64.0, 72.0	61.1, 70.5
BMI (kg/m^2^)				
n	6	6	4	16
Mean (SD)	23.2 (3.75)	26.6 (4.35)	25.4 (4.31)	25.0 (4.15)
Median (min, max)	23.4 (19, 28)	26.5 (21, 32)	26.2 (20, 30)	25.6 (19, 32)
Q1, Q3	19.0, 25.8	22.3, 31.1	22.4, 28.4	21.8, 27.6
Time since initial diagnosis of AD (years)				
n	6	6	4	16
Mean (SD)	0.7 (0.82)	3.0 (4.05)	2.3 (3.20)	1.9 (2.98)
Median (min, max)	0.5 (0, 2)	1.5 (0, 11)	1.0 (0, 7)	1.0 (0, 11)
Q1, Q3	0.0, 1.0	1.0, 3.0	0.6, 4.0	0.0, 2.0
MMSE^b^				
n	6	6	4	16
Mean (SD)	28.0 (1.55)	25.7 (3.61)	24.5 (2.52)	26.3 (2.93)
Median (min, max)	28.0 (26, 30)	24.5 (22, 30)	24.0 (22, 28)	26.5 (22, 30)
Q1, Q3	27.0, 29.0	23.0, 30.0	23.0, 26.0	23.5, 29.0
CDR baseline global score [n (%)]				
0.5	6 (100)	5 (83.3)	3 (75.0)	14 (87.5)
1	0	1 (16.7)	1 (25.0)	2 (12.5)
Missing	0	0	0	0
Number (%) of participants with any prior concomitant medications [n (%)]				
Acetylcholinesterase inhibitors	3 (50.0)	4 (66.7)	2 (50.0)	9 (56.3)
Memantine	0	1 (16.7)	1 (25.0)	2 (12.5)
Acetylcholinesterase inhibitors and memantine	0	0	1 (25.0)	1 (6.3)
Neither acetylcholinesterase inhibitors nor memantine	3 (50.0)	1 (16.7)	0	4 (25.0)
*APOE* genotype at screening				
E3/E4	2 (33.3)	2 (33.3)	2 (50.0)	6 (37.5)
E4/E4	3 (50.0)	4 (66.7)	2 (50.0)	9 (56.3)
Missing	1 (16.7)	0	0	1 (6.3)

APOE = apolipoprotein E gene; BMI = body mass index; n = number of participants; Q1/Q3 = first/third quartile; SD = standard deviation.

a CDR global score was evaluated at screening but recorded in the database only after the corresponding protocol amendment implementation. All 6 participants on ACI-35.030 300 μg (subcohort 1.1) had a CDR Global score of 0.5, while the 2 placebo participants from that sub-cohort had CDR global scores of 0.5 and 1, respectively.

b MMSE is presented as the total score.

**Table 3:** Serious treatment-emergent adverse events by system organ class and preferred term (safety population).

Cohort 1 with ACI-35.030						
SOC PT [n (%)]	ACI-35.030 300 μg (N = 6)	ACI-35.030 900 μg (N = 19)	ACI-35.030 1800 μg (N = 6)	Cohort 1 placebo (N = 10)	Pooled placebo (N = 14)	Total (N = 41)
Any serious TEAEs	2 (33.3)	2 (10.5)	2 (33.3)	0	1 (7.1)	6 (14.6)
Infections and infestations	1 (16.7)	1 (5.3)	0	0	0	2 (4.9)
Diverticulitis	1 (16.7)	0	0	0	0	1 (2.4)
Hemorrhagic fever with renal syndrome	0	1 (5.3)	0	0	0	1 (2.4)
Cardiac disorders	1 (16.7)	0	0	0	0	1 (2.4)
Sinus node dysfunction	1 (16.7)	0	0	0	0	1 (2.4)
Gastrointestinal disorders	0	0	1 (16.7)	0	0	1 (2.4)
Diverticulum	0	0	1 (16.7)	0	0	1 (2.4)
General disorders and administration site conditions	0	1 (5.3)	0	0	0	1 (2.4)
Injection site rash	0	1 (5.3)	0	0	0	1 (2.4)
Injury, poisoning, and procedural complications	0	1 (5.3)	0	0	0	1 (2.4)
Post-traumatic pain	0	1 (5.3)	0	0	0	1 (2.4)
Nervous system disorders	0	1 (5.3)	0	0	0	1 (2.4)
Dizziness	0	1 (5.3)	0	0	0	1 (2.4)
Vascular disorders	0	0	1 (16.7)	0	0	1 (2.4)
Aneurysm thrombosis	0	0	1 (16.7)	0	0	1 (2.4)
Peripheral artery aneurysm	0	0	1 (16.7)	0	0	1 (2.4)
Musculoskeletal and connective tissue disorders	0	0	0	0	1 (7.1)	0
Intervertebral disc protrusion	0		0	0	1 (7.1)	0

Cohort 2 with JACI-35.054					
SOC PT [n (%)]	JACI-35.054 15 μg (N = 6)	JACI-35.054 60 μg (N = 6)	Cohort 2 placebo (N = 4)	Pooled placebo (N = 14)	Total (N = 16)
Any serious TEAEs	0	0	1 (25.0)	1 (7.1)	1 (6.3)
Musculoskeletal and connective tissue disorders	0	0	1 (25.0)	1 (7.1)	1 (6.3)
Intervertebral disc protrusion	0	0	1 (25.0)	1 (7.1)	1 (6.3)

n = Number of participants; PT = preferred term; SOC = system organ class.

For each SOC and PT, participants are included only once.

**Table 4:** Most frequent (≥20% of the participants in any active treatment group) treatment-emergent adverse events by preferred term (safety population).

Cohort 1 with ACI-35.030						
PT [n (%)]	ACI-35.030 300 μg (N = 6)	ACI-35.030 900 μg (N = 19)	ACI-35.030 1800 μg (N = 6)	Cohort 1 placebo (N = 10)	Pooled placebo (N = 14)	Total (N = 41)
Injection site reaction	2 (33.3)	14 (73.7)	6 (100.0)	0	0	22 (53.7)
COVID-19	0	7 (36.8)	2 (33.3)	3 (30.0)	4 (28.6)	12 (29.3)
Fatigue	0	4 (21.1)	1 (16.7)	2 (20.0)	2 (14.3)	7 (17.1)
Headache	0	4 (21.1)	3 (50.0)	0	1 (7.1)	7 (17.1)
Nasopharyngitis	3 (50.0)	2 (10.5)	1 (16.7)	1 (10.0)	2 (14.3)	7 (17.1)

Cohort 2 with JACI-35.054					
PT [n (%)]	JACI-35.054 15 μg (N = 6)	JACI-35.054 60 μg (N = 6)	Cohort 2 placebo (N = 4)	Pooled placebo (N = 14)	Total (N = 16)
Headache	2 (33.3)	1 (16.7)	1 (25.0)	1 (7.1)	4 (25.0)
Injection site reaction	1 (16.7)	2 (33.3)	0	0	3 (18.8)
Malaise	2 (33.3)	1 (16.7)	0	0	3 (18.8)
Pyrexia	3 (50.0)	0	0	0	3 (18.8)
Epistaxis	2 (33.3)	0	0	0	2 (12.5)
Myalgia	2 (33.3)	0	0	0	2 (12.5)
Ventricular extrasystoles	2 (33.3)	0	0	0	2 (12.5)

n = number of participants; PT = preferred term.

For each PT, participants are included only once.

**Table 5:** Tabulation of post-hoc statistical analysis results comparing changes from baseline of plasma brain derived (BD) Tau in active treatment study arms (ACI-35.030 or JACI-35.054) versus corresponding changes in the pooled placebo study arm.

Week	Group	Change from baseline LS^a^ mean (95% CI^b^)	Change from baseline LS mean expressed as % relative to baseline	Difference from placebo (95% CI)	p-value^c^
42	Pooled placebo	0.22 (−0.83 to 1.27)	4.0%		
	ACI-35.030 900 μg	1.57 (0.62–2.51)	28.5%	1.35 (−0.07 to 2.76)	0.0625
	ACI-35.030 1800 μg	2.21 (0.77–3.66)	37.3%	1.99 (0.20–3.78)	0.0291
50	Pooled placebo	0.16 (−0.78 to 1.10)	2.9%		
	ACI-35.030 900 μg	1.59 (0.77–2.41)	28.8%	1.43 (0.18–2.68)	0.0250
	ACI-35.030 1800 μg	1.86 (0.33–3.40)	31.4%	1.71 (−0.09 to 3.50)	0.0625
67	Pooled placebo	−0.00 (−0.94 to 0.94)	0%		
	ACI-35.030 900 μg	1.36 (0.53–2.20)	24.7%	1.37 (0.11–2.62)	0.0332
	ACI-35.030 1800 μg	2.16 (0.63–3.70)	36.5%	2.17 (0.37–3.96)	0.0183
74	Pooled placebo	0.45 (−0.48 to 1.39)	8.3%		
	ACI-35.030 900 μg	1.98 (1.15–2.82)	35.9%	1.53 (0.27–2.79)	0.0175
	ACI-35.030 1800 μg	2.31 (0.78–3.84)	30.0%	1.86 (0.06–3.65)	0.0430
20	Pooled placebo	−0.06 (−1.39 to 1.26)	−1.1%		
	JACI-35.054 15 μg	Non estimated	Non estimated	Non estimated	–
	JACI-35.054 60 μg	2.43 (0.78–4.07)	39.8%	2.49 (0.37–4.61)	0.0219

a Least Squares.

b Confidence Interval.

c Linear Mixed Model with baseline and treatment arm∗visit as fixed factors, participant ID as random factor, Satterthwaite's method for degrees of freedom, uncorrected p-values.

**Table 6:** Tabulation of post-hoc statistical analysis results comparing changes from baseline of plasma pTau217 in active treatment study arms (ACI-35.030 or JACI-35.054) versus corresponding changes in the pooled placebo study arm.

Week	Group	Change from baseline LS^a^ mean (95% CI^b^)	Change from baseline LS mean expressed as % relative to baseline	Difference from placebo (95% CI)	p-value^c^
10	Pooled placebo	0.0025 (−0.0155 to 0.0205)	2.1%		
	ACI-35.030 900 μg	−0.0173 (−0.0328 to −0.0019)	−11.5%	−0.0198 (−0.0438 to 0.0041)	0.1036
	ACI-35.030 1800 μg	−0.0345 (−0.0652 to −0.0038)	−18.5%	−0.0370 (−0.0731 to −0.0010)	0.0443
15	Pooled placebo	0.0049 (−0.0162 to 0.0261)	4.2%		
	ACI-35.030 900 μg	−0.0206 (−0.0377 to −0.0034)	−13.6%	−0.0256 (−0.0530 to 0.0019)	0.0675
	ACI-35.030 1800 μg	−0.0555 (−0.0879 to −0.0231)	−29.8%	−0.0605 (−0.0997 to −0.0213)	0.0027
31	Pooled placebo	0.0006 (−0.0198 to 0.0209)	0.5%		
	ACI-35.030 900 μg	−0.0292 (−0.0468 to −0.0117)	−19.3%	−0.0298 (−0.0569 to −0.0027)	0.0311
	ACI-35.030 1800 μg	−0.0170 (−0.0495 to 0.0154)	−9.1%	−0.0176 (−0.0563 to 0.0211)	0.3717
36	Pooled placebo	0.0012 (−0.0168 to 0.0192)	1.0%		
	ACI-35.030 900 μg	−0.0263 (−0.0419 to −0.0106)	−17.4%	−0.0274 (−0.0516 to −0.0034)	0.0257
	ACI-35.030 1800 μg	−0.0367 (−0.0674 to −0.0060)	−19.7%	−0.0379 (−0.0740 to −0.0019)	0.0394
74	Pooled placebo	0.0159 (−0.0021 to 0.0339)	13.5%		
	ACI-35.030 900 μg	−0.0087 (−0.0246 to 0.0072)	−5.8%	−0.0246 (−0.0488 to −0.0003)	0.0469
	ACI-35.030 1800 μg	0.0129 (−0.0178 to 0.0436)	6.9%	−0.0030 (−0.0390 to 0.0331)	0.8707
10	Pooled placebo	0.0063 (−0.0123 to 0.0250)	5.3%		
	JACI-35.054 15 μg	0.0212 (−0.0070 to 0.0495)	13.8%	0.0149 (−0.0192 to 0.0490)	0.3868
	JACI-35.054 60 μg	−0.0296 (−0.0581 to −0.0011)	−17.9%	−0.0359 (−0.0704 to −0.0015)	0.0411

a Least Squares.

b Confidence Interval.

c Linear Mixed Model with baseline and treatment arm∗visit as fixed factors, participant ID as random factor, Satterthwaite's method for degrees of freedom, uncorrected p-values.

## Discussion

This study shows that active anti-Tau immunotherapies can safely induce the immune system of participants with early AD aged 50–75 years to produce and maintain antibodies targeting phosphorylated Tau. Moreover, dependent on the active immunotherapy formulation, a different polyclonal anti-Tau antibody response profile developed, despite using the same phospho-Tau peptide. While the two active immunotherapies generated robust titres against pathological Tau species, ACI-35.030 showed an earlier antibody response more specifically directed against phosphorylated Tau species compared to non-phosphorylated Tau, and a sustained and stable response against ePHF, which represents the endogenous pathological Tau in AD pathology. This process of antibody maturation is well established in humans and is fundamental for successfully fighting infectious agents. However, this has not been reported to date with another anti-Tau active immunotherapy, AADVac1, which has an immunogen covering the N-terminal cysteinylated tau 294–305/4R region and showed preliminary evidence of slowing of neurodegeneration using plasma NfL, a non-specific fluid biomarker, and of AD-related decline in an post-hoc analysis from one clinical trial conducted in 196 participants with mild AD.^30^^,^^31^ The fast onset of the targeted response, the high responder rate and the boosting of antibody breadth toward pathological Tau reflects the capacity of ACI-35.030 to harness the adaptability of our immune system to extend its reach, aimed at eliminating pathological Tau variants. Safety and tolerability, the other primary outcomes of the study were good with both active immunotherapies, with no participants withdrawing from the study due to adverse events. The most consistently observed adverse events, commonly reported with active immunisation, were headaches and local injection site reactions. They occurred in a dose-dependent manner, were generally mild to moderate in severity and were self-limiting. The numerous measures of fluid-based biomarkers gave two notable outcomes. Using the immunoassay that selectively measures brain-derived Tau (BD-Tau) in blood, a significant accumulation was observed with the 2 highest treatment doses of ACI-35.030.^27^ Moreover, on average, plasma BD-Tau levels remained constant post the last dosing at 48 weeks until the final analysis at 74 weeks. As the epitopes recognised by the ACI-35.030-induced antibodies do not interfere in the BD-Tau assay, the increase of plasma BD-Tau levels observed with ACI-35.030 immunisation, as compared to placebo, suggests stabilisation in the plasma via antibody-target engagement with these Tau variants. In contrast, plasma pTau217, a biomarker for detecting AD pathology and predicting future development of AD dementia, decreased in a dose-dependent manner post treatment with ACI-35.030, which could indicate a pharmacodynamic effect.^28^^,^^29^ These results may reflect that as the antibody response matures within each participant post-treatment with ACI-35.030, the antibody repertoire binds more effectively to the Tau species measured in the BD-Tau assay. The relevance of these observations needs to be confirmed in larger cohorts. In this study, ACI-35.030, derived from the SupraAntigen® liposome–based platform, induced a strong polyclonal antibody response that matured and was maintained against key pathological forms of Tau believed to drive Tau aggregation and AD. JACI-35.054, a CRM197 carrier-protein conjugate, triggered a more heterogenous antibody response, with high responder rates, that was less selective for pathological Tau species. Thus, based on a faster and more stable antibody response, which is selective for and maturing against the endogenous pathological form of Tau, i.e., ePHF, the active immunotherapy, ACI-35.030, was selected for further development. As compared to mAbs, less frequent dosing is required to maintain titres, and efficacy-modifying anti-drug antibodies are absent. Based on the fact that ACI-35.030 (JNJ-64042056) was well tolerated at all tested doses and induced a fast and selective response against endogenous pathological Tau, this SupraAntigen®-based active immunotherapy is in progress into the next phase of clinical development. The remarkably limited screen failure rate (22/79; ∼28%) observed may be explained by the very careful identification of participants selected in centres highly experienced in the conduct of early-stage AD studies. The study sites were not given any specific guidelines regarding the preselection or selection of participants and conducted recruitment in accordance with their local rules. This early-stage study by its nature, has certain limitations, notably it was not powered to investigate the effects of study drug on biomarkers, vMRI and on clinical endpoints. Consequently, the lack of statistical significance in this context does not exclude a potential effect. Brain Tau-PET imaging was not performed, precluding the assessment of the polyclonal antibody response against pathological Tau species needed to prevent or inhibit brain Tau spreading. In this early phase study, the treatment period was also limited, thus preventing measurement of the long-term antibody response and pharmacodynamic effects of the study treatment. Based on the limited number of participants, all of whom were white, the influence of sex, gender, race and ethnicity could not be adequately studied nor could the influence of socioeconomic factors be assessed. This will be addressed in larger studies, with efforts including community engagement to ensure a representative and diverse population for this purpose. The study data has shown that injection site reactions were observed exclusively in participants receiving active study treatments. While this observation was unlikely to induce a potential risk of unblinding during the course of this early-phase study, this possibility should be appropriately controlled in subsequent clinical phases by using an adjuvanted placebo formulation able to induce such local reactions while preserving the safety of participants. The phase 2b Retain study (NCT06544616) that is currently ongoing in study population with preclinical AD has been designed to address the above-mentioned points.

## Contributors

OS, JG, JS, and AP defined the overall study design, endpoints and study population with the support of PS, who was the international coordinating investigator. JW and NF took part in the statistical analysis plans and in interim and final study analyses with the support of IK, LS, and GTB for interim analyses. JM, BL, and VH were responsible for clinical operation-related activities during the study conduct. Safety data monitoring was ensured by JG and VG with the support of OS. MV, EG, EF, DTH, CT, GTB, MKV, and AP took part in the selection of antibody titres and fluid biomarker assays and in the analysis of related interim and final dataset. MH, SK, JR, PD, EGBV, CM, ABH, MJ, CR, and CP were principal investigators at the different study sites where study participants were screened and randomised. All authors from AC Immune SA and Johnson & Johnson Innovative Medicine took part in the final data analysis and reviewed the clinical study report. All authors had full access to all data in the study, approved the final manuscript and had final responsibility for the decision to submit for publication. All or part of the underlying data were verified by OS, JM, MV, VG, EG, JG, JW, NF, LS, GTB, MKV, and PS.

## Data sharing statement

AC Immune SA and Johnson & Johnson Innovative Medicine are making provisions to grant access to group-level data (analysis datasets) only in this early phase clinical trial in order to enhance public health and advance science. Qualified researchers who engage in rigorous independent scientific research may be given data access after review and approval of research proposal, Statistical Analysis Plan (SAP) and execution of a Data Use Agreement. Other information such as study protocol and statistical analysis plan can be accessed at clinicaltrials.gov and in Supplementary Material. One study participant did not consent to the storage and further use of samples for future research.

## Declaration of interests

OS, JM, MV, EG, EF, DTH, VH, VG, JW, NF, BL, MKV, and AP, who is the CEO, are full-time employees and shareholders of AC Immune SA. JS was a full-time employee and shareholder of AC Immune SA at the time of study conduct. JG received consulting fees as a clinical advisor of AC Immune SA. IK is a full-time employee and shareholder of Johnson & Johnson Innovative Medicine, she is also the co-holder of patent JPI6073USPSP2. LS is a full-time employee and shareholder of Johnson & Johnson Innovative Medicine, he is also the co-holder of patents JPl6059OMPCT1 and JPl6073USPSP2. GTB is a full-time employee and shareholder of Johnson & Johnson Innovative Medicine, he is also the co-holder of patents JAB6123USPSP2, JAB7064USPSP2, JAB7165USPSP2, and JAB721SUSPSP2. CT is a full-time employee and shareholder of Johnson & Johnson Innovative Medicine, she is also the co-holder of patents PCT/US2022/074902 and PCT/US2024/052885. JR was study investigator and fees related to the conduct of the study were paid to his institution, he had also academic research grants from Sigrid Juselius Foundation that were received by his institution and also from the Finnish Governmental Research Funding (VTR) that were received by Turku University Hospital. MH, SK, PD, EGBV, and ABH were study investigators and fees related to the conduct of the study were paid to their respective institutions. MJ was study investigator and fees related to the conduct of the study were paid to his institution, he received compensation for advisory board attendance with Biogen, Eli Lilly, Eisai, and BioArctic. CM was study investigator and fees related to the conduct of the study were paid to her institution, she was granted award for investigator led trial of ultrafast MRI in dementia with Biogen, she received compensation for advisory board attendance with Roche, Eli Lilly, Eisai, Novartis, Neurimmune, MSD, and GSK, she received honorarium for lecturing at sponsored symposium with Eli Lilly and Eisai, she received payments from Biogen, Eisai, Roche, and Alector for travel and accommodation for advisory board meetings held at different scientific conferences, she received compensation as DSMB chair from Imperial and Immunobrain, and she is also Director of NIHR UK Dementia Trials Network. CR was study investigator and fees related to the conduct of the study were paid to his institution, he is also the Founder, CEO, and majority shareholder of Scottish Brain Sciences, he received consulting fees from Biogen, Eisai, MSD, Actinogen, Roche, Eli Lilly, and Novo Nordisk, he received payment or honoraria for lectures and presentations from Roche, Eisai, and Eli Lilly, and he also received compensation from Novo Nordisk for advisory board attendance. CP was study investigator and fees related to the conduct of the study were paid to her institutions. PS is Professor Emeritus at Amsterdam UMC, is a DSMB member at Immunobrain Checkpoint and at Johnson & Johnson Innovative Medicine, is a co-chair at the steering committee of EVOKES studies at Novo-Nordisk, is a full-time employee of EQT Life Sciences and, as the ACI-35-1802 study coordinating investigator, he received consulting fees that were paid to Amsterdam UMC.

## Appendix A Supplementary data

Supplementary Materials

Clinical Study Protocol

Statistical Analysis Plan

Author Statement ICJME Transfer Form
