# Supplementary material for: Safety and immunogenicity of two Tau-targeting active immunotherapies, ACI-35.030 and JACI-35.054, in participants with early Alzheimer's disease: a phase 1b/2a, multicentre, double-blind, randomised, placebo-controlled study
Source: eBioMedicine. 2025 Sep 18;120:105940. doi: 10.1016/j.ebiom.2025.105940 (PMC12481106; doi:10.1016/j.ebiom.2025.105940)
Supplement: Supplementary Materials [file mmc1.docx]

**Appendix A. Supplementary material**

Materials and Methods page 2

Supplementary Figures pages 3−59 (Figures S1-S56)

Supplementary Tables pages 60−71 (Tables S1-S7)

**Materials and Methods**

**Statistical methods:** In the post-hoc analysis, Plasma brain derived Tau and plasma pTau217 levels changes from baseline were analysed using linear mixed models (LMMs) to assess differences between active treated arms and the pooled placebo arm at different timepoints. LMMs were adjusted separately for Cohort 1 active treated arms (ACI-35.030 300µg, 900µg and 1800µg) against pooled placebo arm and for Cohort 2 active treated arms (JACI-35.054 15µg and 60µg) against pooled placebo arm, to account for potential kinetics and variance structure differences. Each model comprised baseline values and treatment arm*visit as fixed factors and subject ID as random factor (intercept). Least-squares means confidence intervals and subsequent contrasts used Satterthwaite’s method for degrees of freedom, and resulting p-values were reported uncorrected. The R software and lme4 (v. 1.1-31) and emmeans (v1.8.5) packages were used to carry out this analysis.

**Immunogenicity assays to measure antibody levels against different Tau species:**IgG levels were measured by ECLIA (MesoScale Discovery), whereas IgM levels were measured by ELISA. Briefly, 96-well plates were coated with either Tau peptide, pTau peptide or ePHF (prepared according to Vandermeeren M, Borgers M, Van Kolen K, Theunis C, Vasconcelos B, Bottelbergs A, et al. Anti-Tau Monoclonal Antibodies Derived from Soluble and Filamentous Tau Show Diverse Functional Properties in vitro and in vivo. J Alzheimers Dis. 2018;65(1):265-281). Serial dilutions of each serum sample (starting from 1:100) were applied to the plates. IgG antibodies were detected by a polyclonal anti-human IgG, whereas IgM were detected using a polyclonal anti-human IgM (Jackson ImmunoResearch, UK). Arbitrary units (AU) were calculated based on a reference standard (polyclonal serum pool from rhesus monkeys immunized with either ACI-35, ACI-35.030 or JACI-35.054 or proprietary monoclonal antibodies). For peptide (phospho- and non-phospho Tau IgG and IgM) assays, AU assignment was based on Lower Limit of Quantification (LLOQ) of the assay, with 1 AU/mL corresponding to the LLOQ. For ePHF, LLOQ corresponds to 3 AU/mL. Responder rates were determined using pre-defined validated assay thresholds.

**Immunogenicity assays to measure anti-CRM and anti-T50 IgG:** IgG levels were measured by ELISA. Briefly, 96-well plates were coated with CRM or T50 protein. Serial dilutions of each serum sample (starting from 1:100) were applied to the plates. IgG antibodies were detected by a polyclonal anti-human IgG (Jackson ImmunoResearch, UK). Arbitrary units (AU) were calculated based on a reference standard (for anti-CRM IgG: anti-Diphtheria Toxin antibody (Abcam); for anti-T50 IgG: polyclonal serum pool from rhesus monkeys immunized with ACI-35.030). AU assignment was based on the LLOQ of the assay, with 1 AU/mL corresponding to the LLOQ.

**Evaluation of anti-dsDNA antibodies:**Presence of anti-dsDNA antibodies in serum was first evaluated using an anti-dsDNA ELIA assay (EliA dsDNA, Phadia 250, ThermoFisher Scientific) according to the manufacturers’ instructions. In cases of positive anti-dsDNA antibodies by the ELiA, the FARR assay (dsDNA-Ab RIA, Tecan) was used to further confirm the results.

**Supplementary Figures**


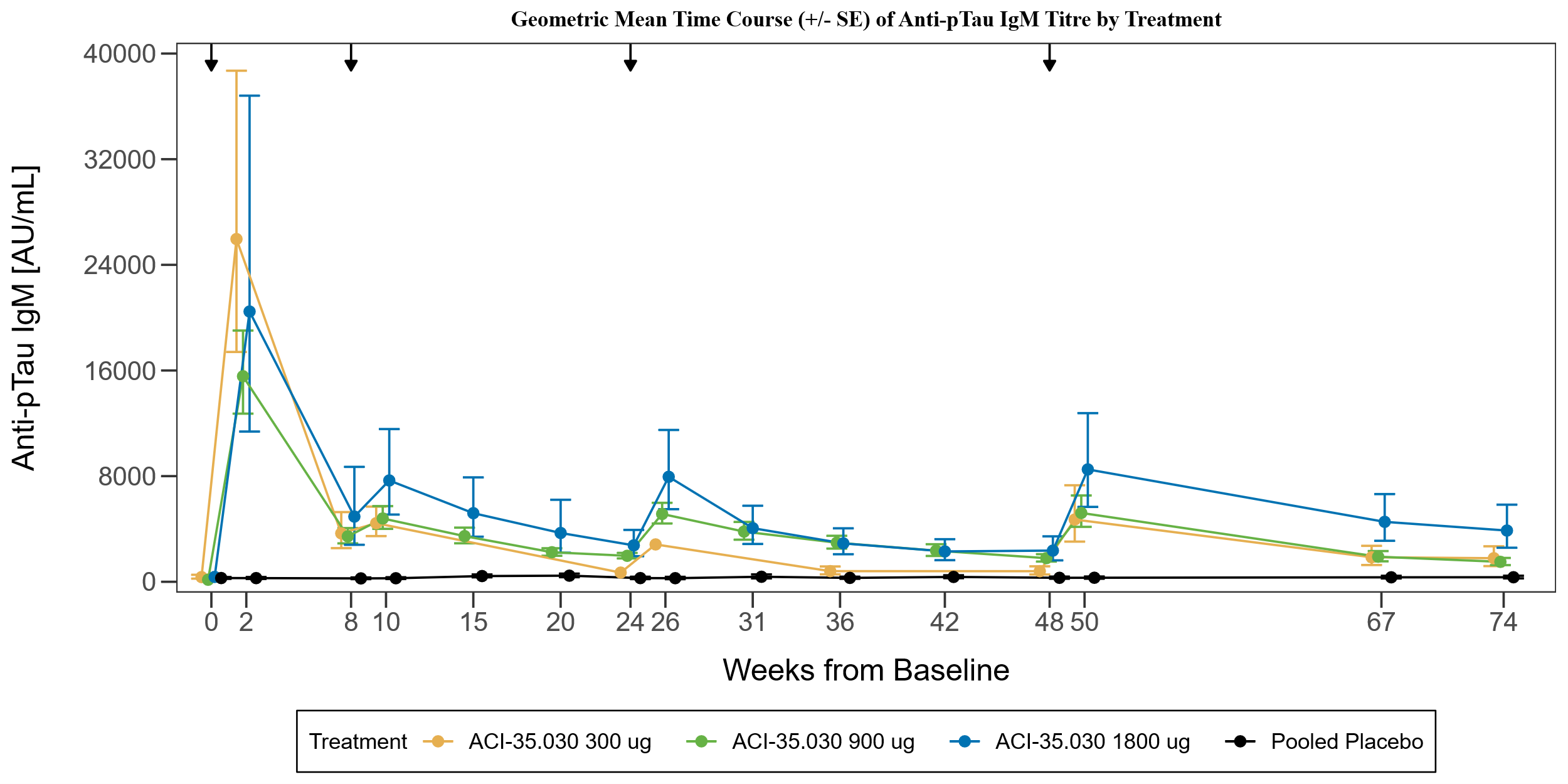

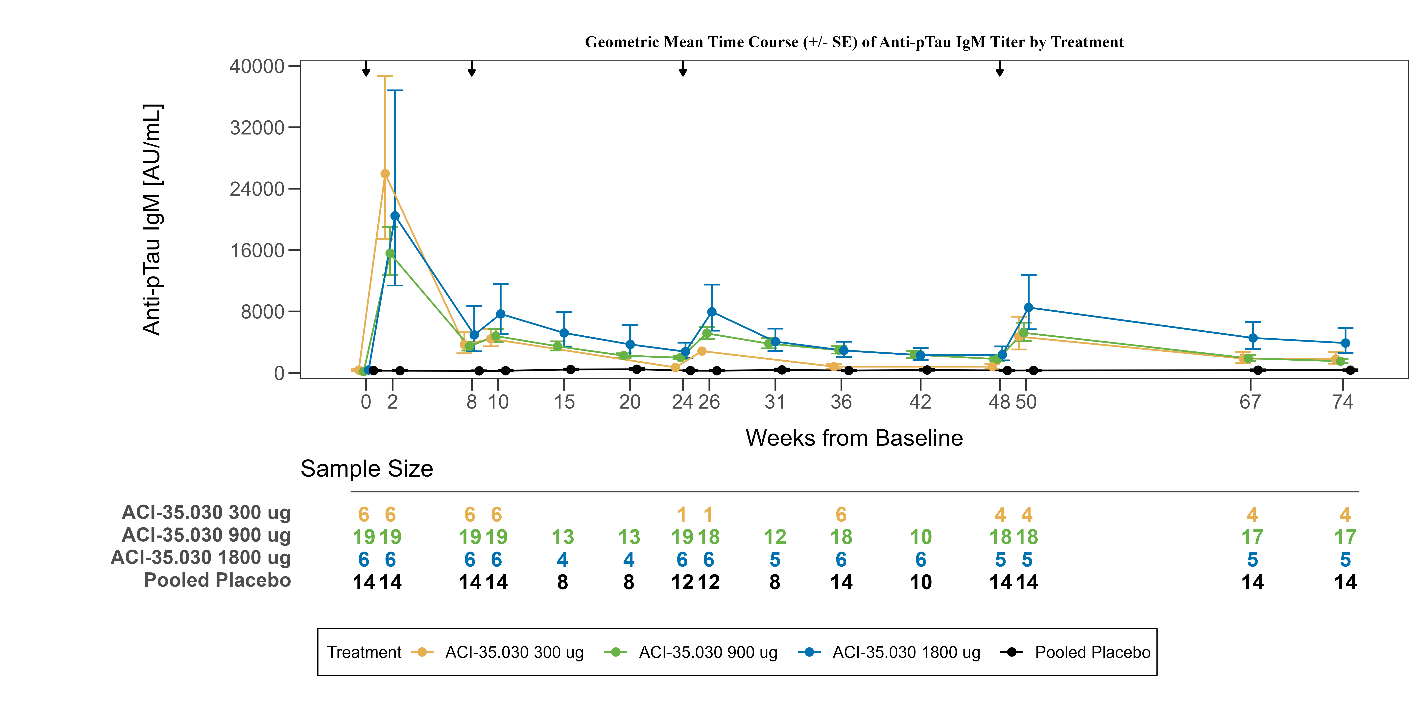
**Figure S1: Geometric mean of Anti-pTau IgM Titres vs nominal visit time by study treatment arm (Cohort 1).** Error bars denote standard error of the mean. Number of subjects by nominal visit and study treatment arm tabulated below. The four vertical arrows at the top of the graph denote nominal study visit times for administration of ACI-35.030 or placebo.


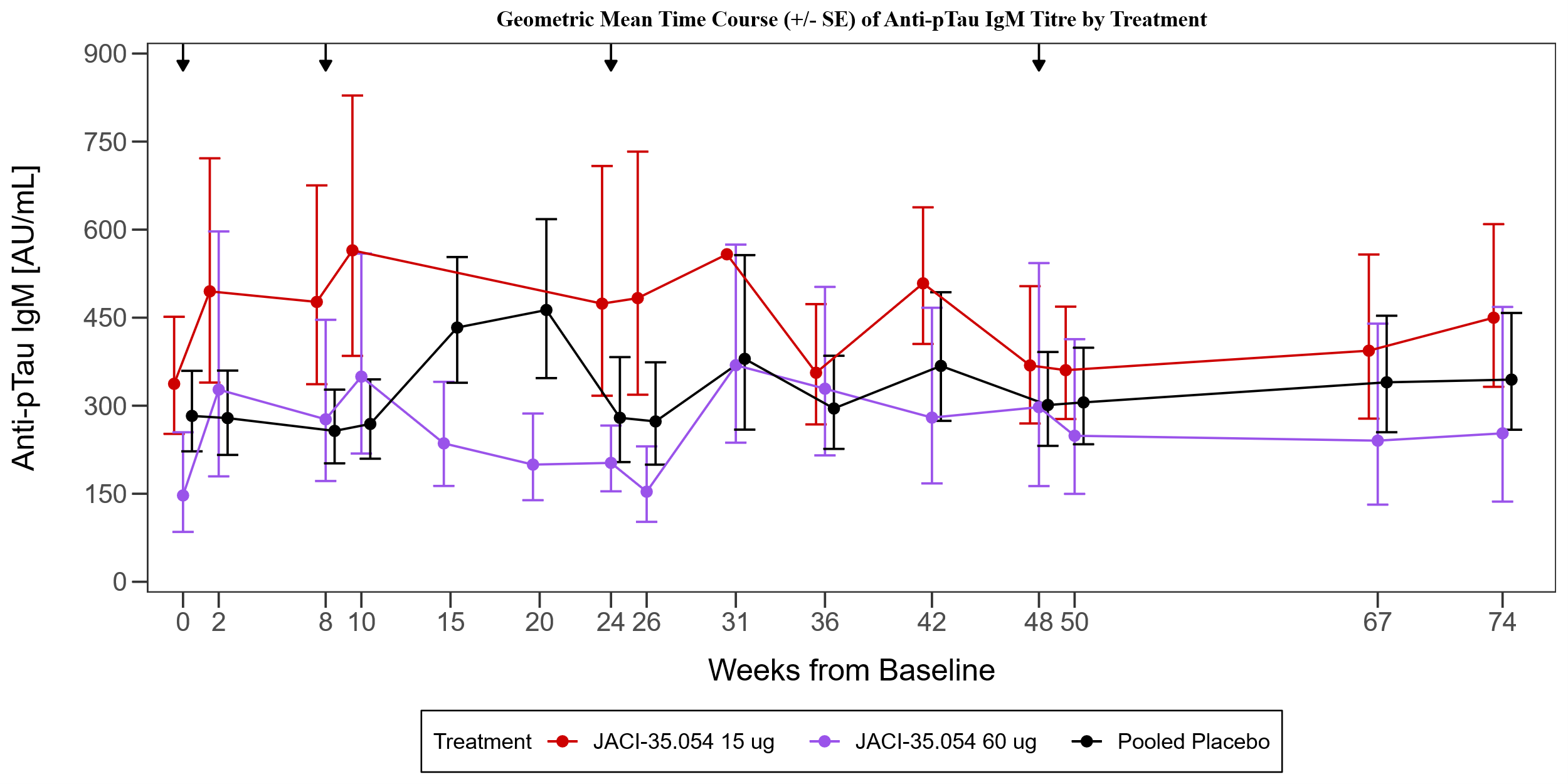
**Figure S2: Geometric mean of Anti-pTau IgM Titres vs nominal visit time by study treatment arm (Cohort 2).** Error bars denote standard error of the mean. Number of subjects by nominal visit and study treatment arm tabulated below. The four vertical arrows at the top of the graph denote nominal study visit times for administration of JACI-35.054 or placebo.


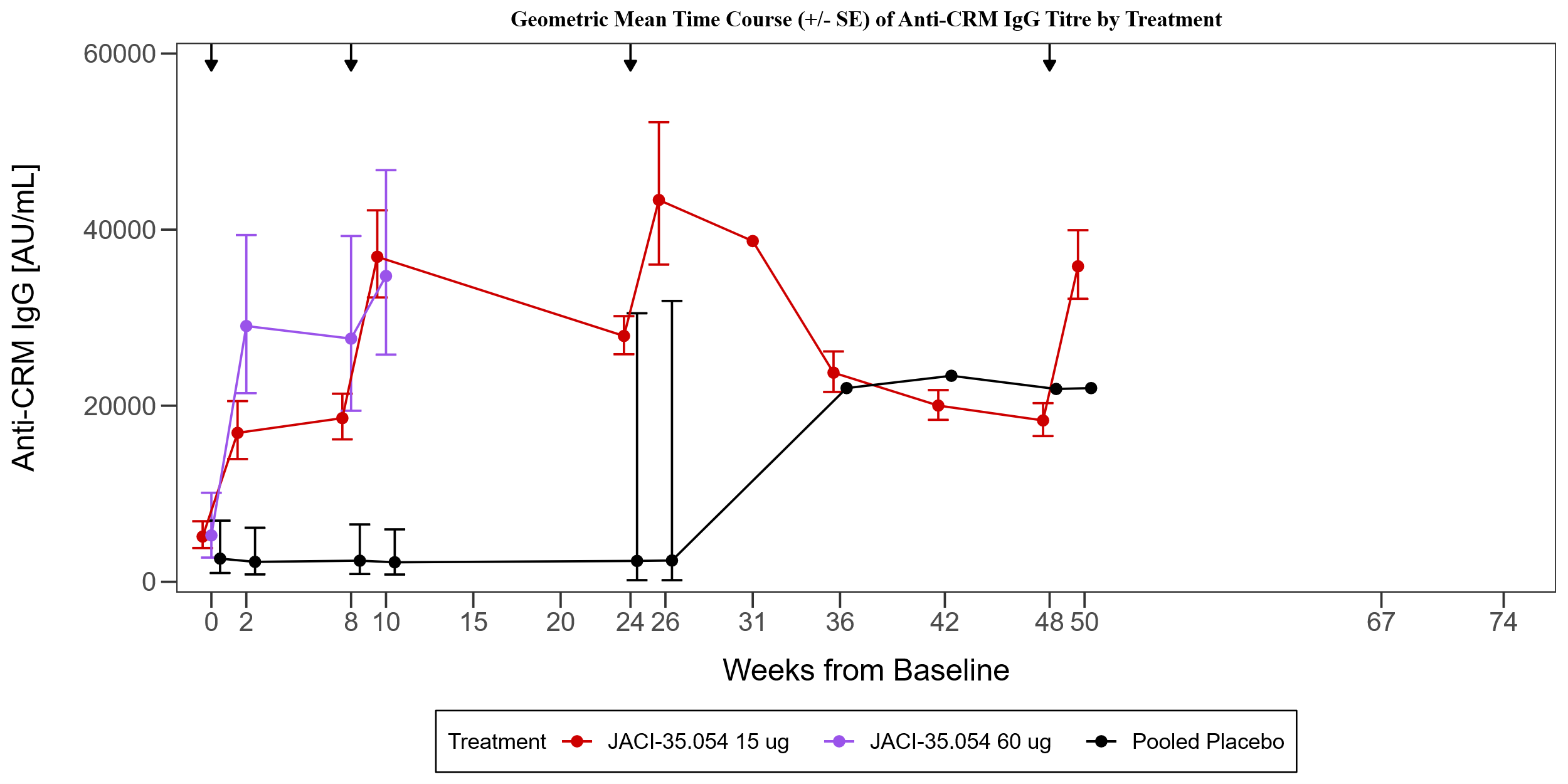
**Figure S3: Geometric mean of Anti-CRM IgG vs nominal visit time by study treatment arm (Cohort 2).** Error bars denote standard error of the mean. Number of subjects by nominal visit and study treatment arm tabulated below. The four vertical arrows at the top of the graph denote nominal study visit times for administration of JACI-35.054 or placebo.


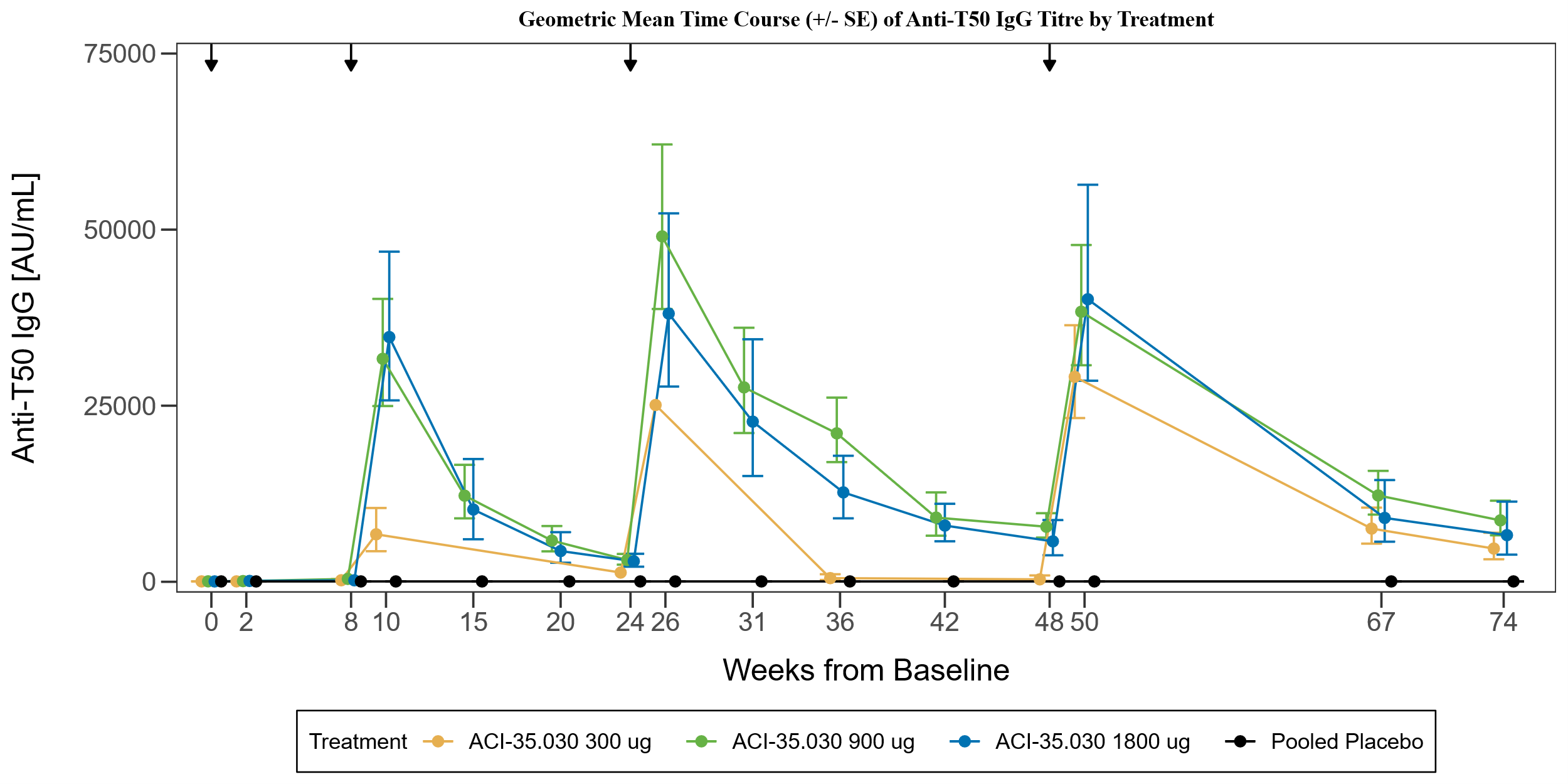
**Figure S4: Geometric mean of Anti-T50 IgG Titres vs nominal visit time by study treatment arm (Cohort 1).** Error bars denote standard error of the mean. Number of subjects by nominal visit and study treatment arm tabulated below. The four vertical arrows at the top of the graph denote nominal study visit times for administration of ACI-35.030 or placebo


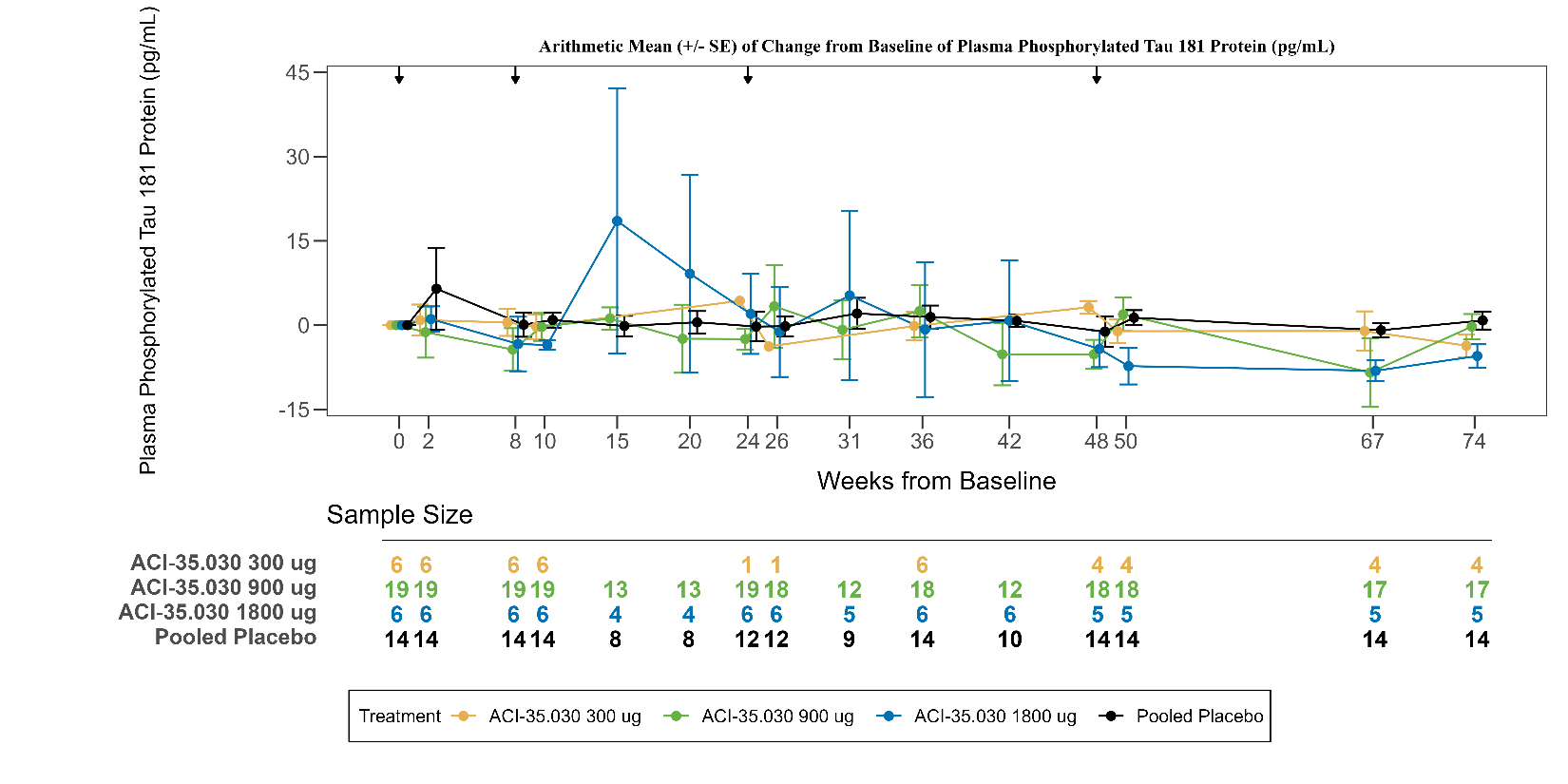
**Figure S5: Arithmetic mean of change from baseline of plasma Phosphorylated Tau 181 Protein vs nominal visit time by study treatment arm (Cohort 1).** Error bars denote standard error of the mean. Number of subjects by nominal visit and study treatment arm tabulated below. The four vertical arrows at the top of the graph denote nominal study visit times for administration of ACI-35.030 or placebo.


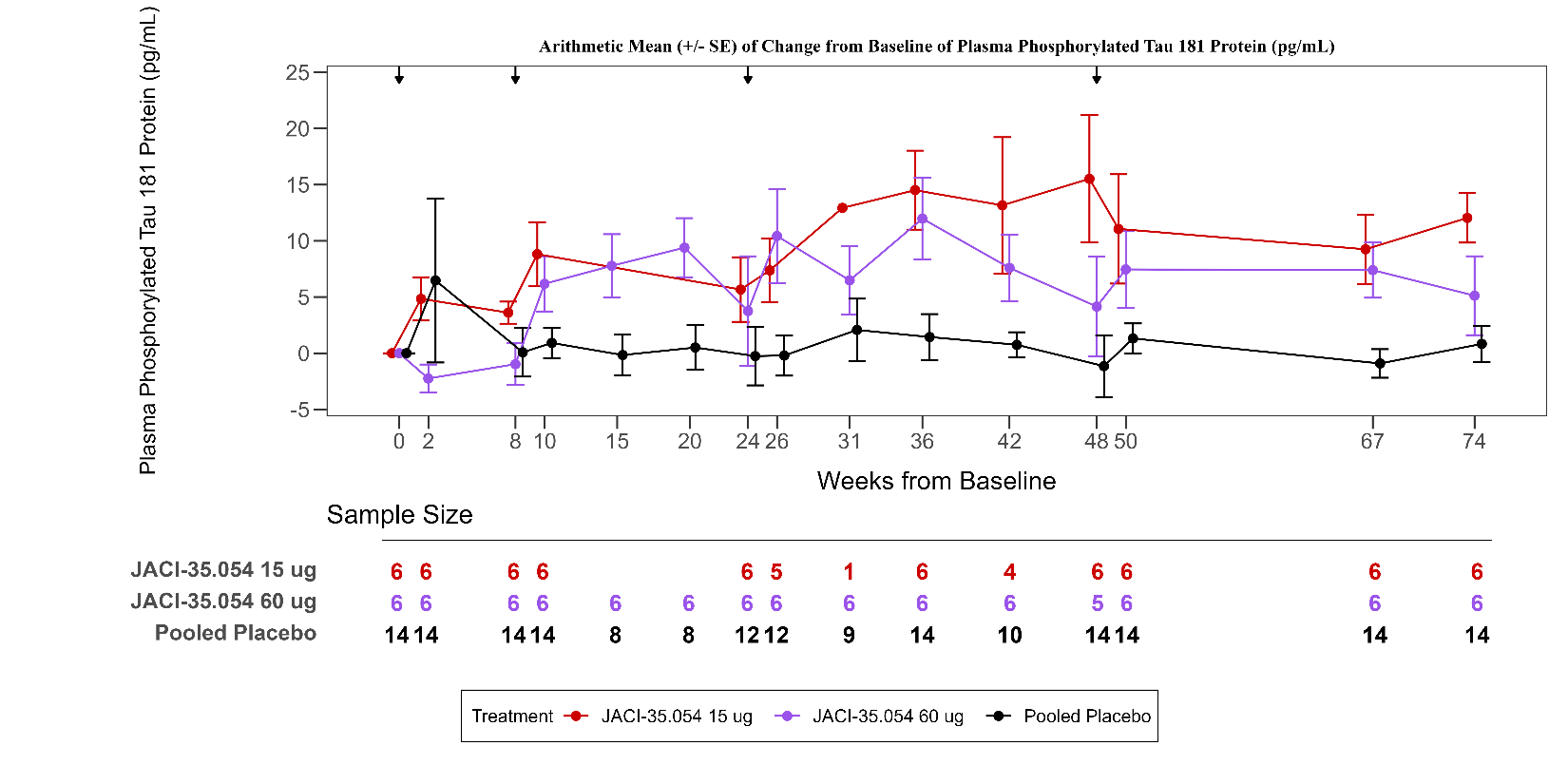


**Figure S6: Arithmetic mean of change from baseline of plasma Phosphorylated Tau 181 Protein vs nominal visit time by study treatment arm (Cohort 2).** Error bars denote standard error of the mean. Number of subjects by nominal visit and study treatment arm tabulated below. The four vertical arrows at the top of the graph denote nominal study visit times for administration of JACI-35.054 or placebo.


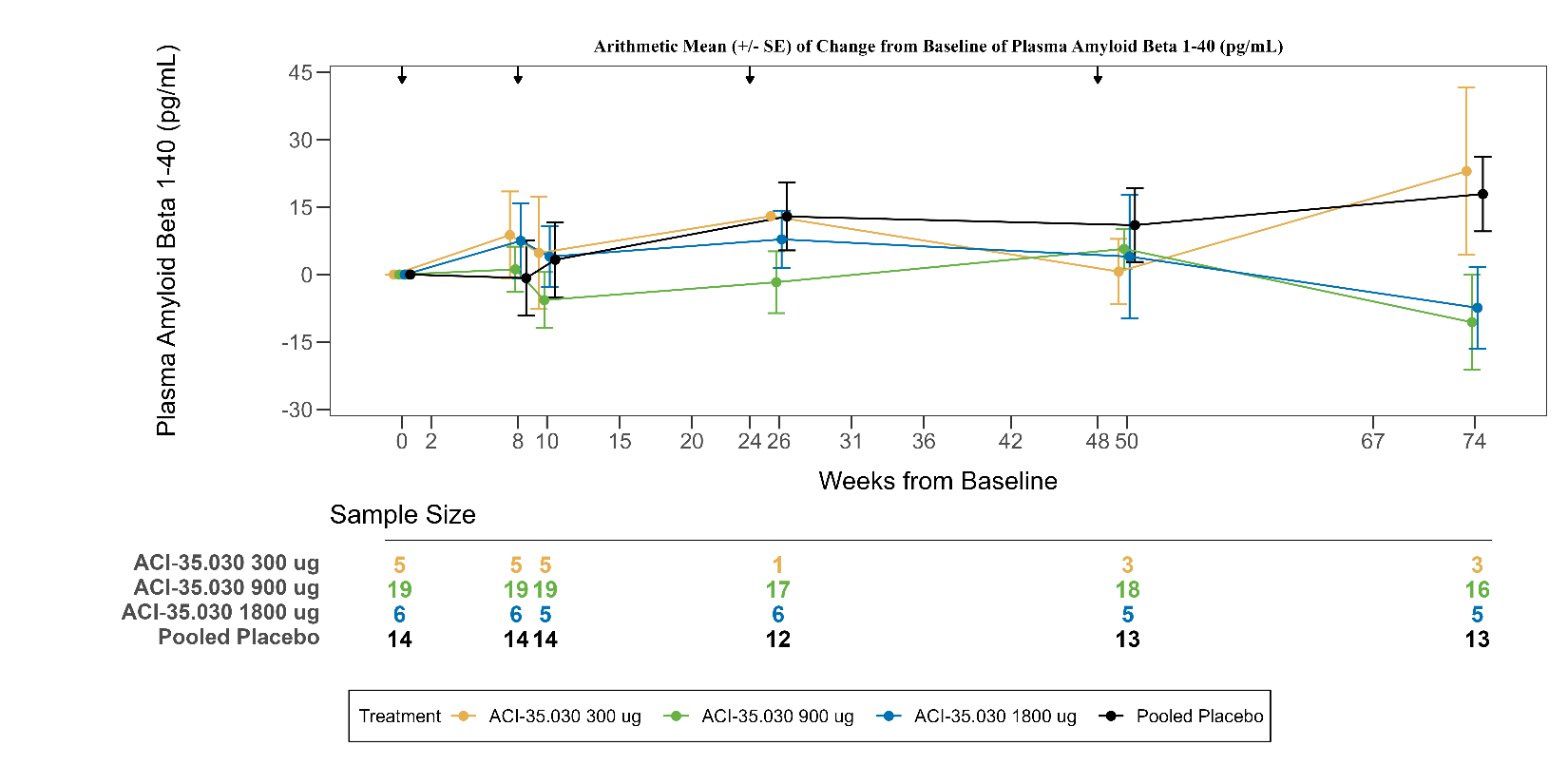
**Figure S7: Arithmetic mean of change from baseline of plasma Amyloid Beta 1-40 vs nominal visit time by study treatment arm (Cohort 1).** Error bars denote standard error of the mean. Number of subjects by nominal visit and study treatment arm tabulated below. The four vertical arrows at the top of the graph denote nominal study visit times for administration of ACI-35.030 or placebo.


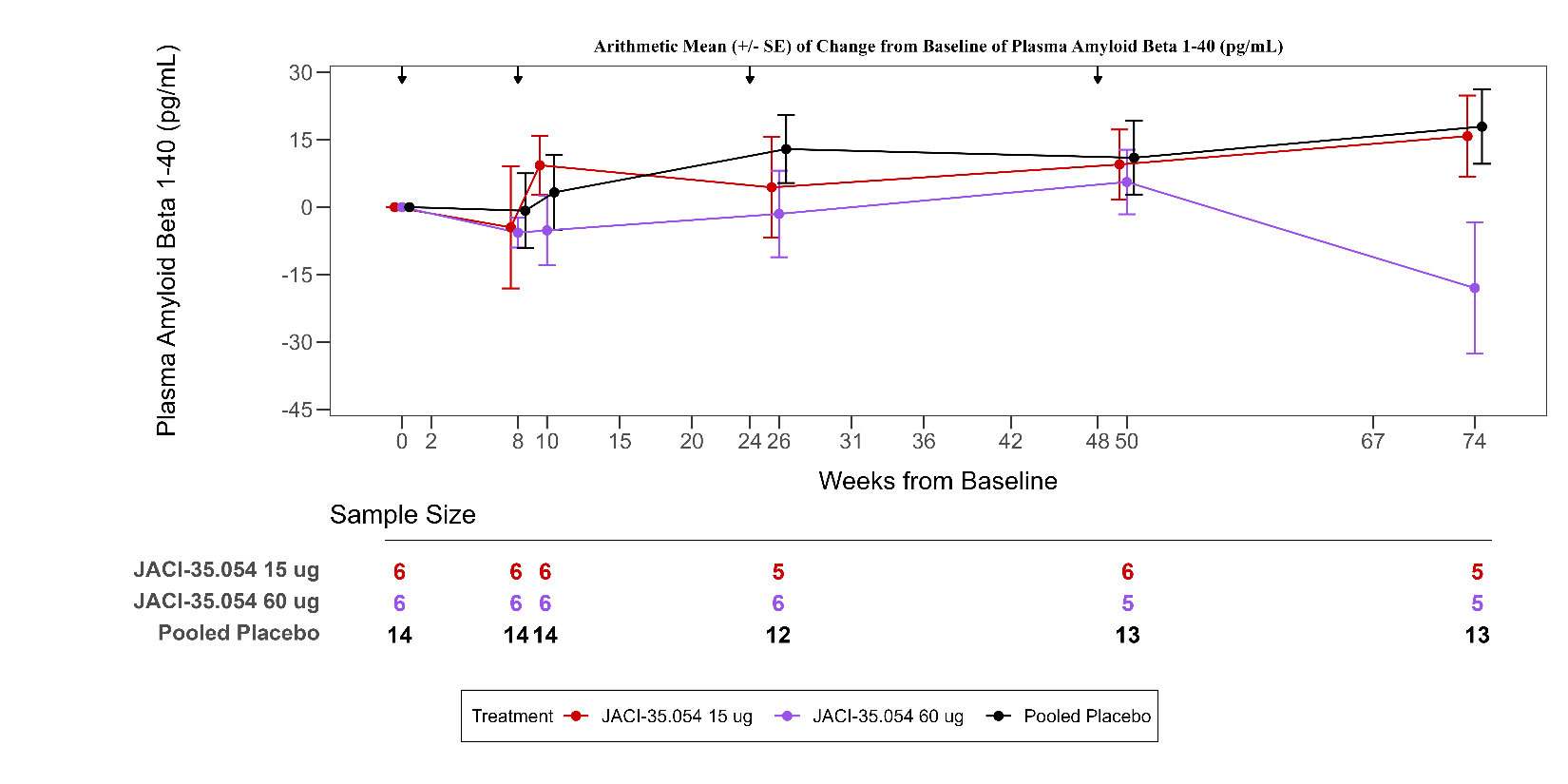
**Figure S8: Arithmetic mean of change from baseline of plasma Amyloid Beta 1-40 vs nominal visit time by study treatment arm (Cohort 2).** Error bars denote standard error of the mean. Number of subjects by nominal visit and study treatment arm tabulated below. The four vertical arrows at the top of the graph denote nominal study visit times for administration of JACI-35.054 or placebo.


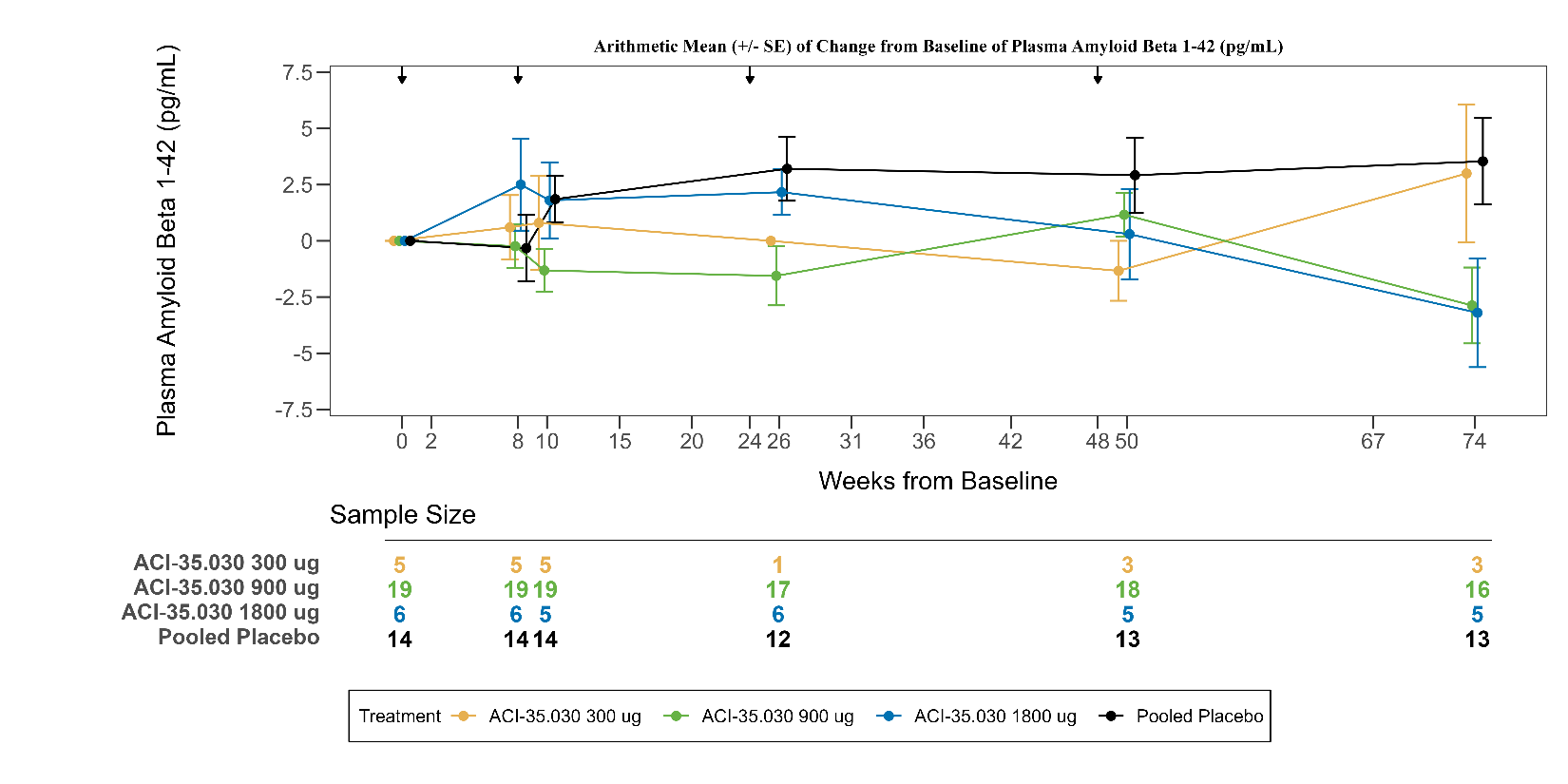
**Figure S9: Arithmetic mean of change from baseline of plasma Amyloid Beta 1-42 vs nominal visit time by study treatment arm (Cohort 1).** Error bars denote standard error of the mean. Number of subjects by nominal visit and study treatment arm tabulated below. The four vertical arrows at the top of the graph denote nominal study visit times for administration of ACI-35.030 or placebo.


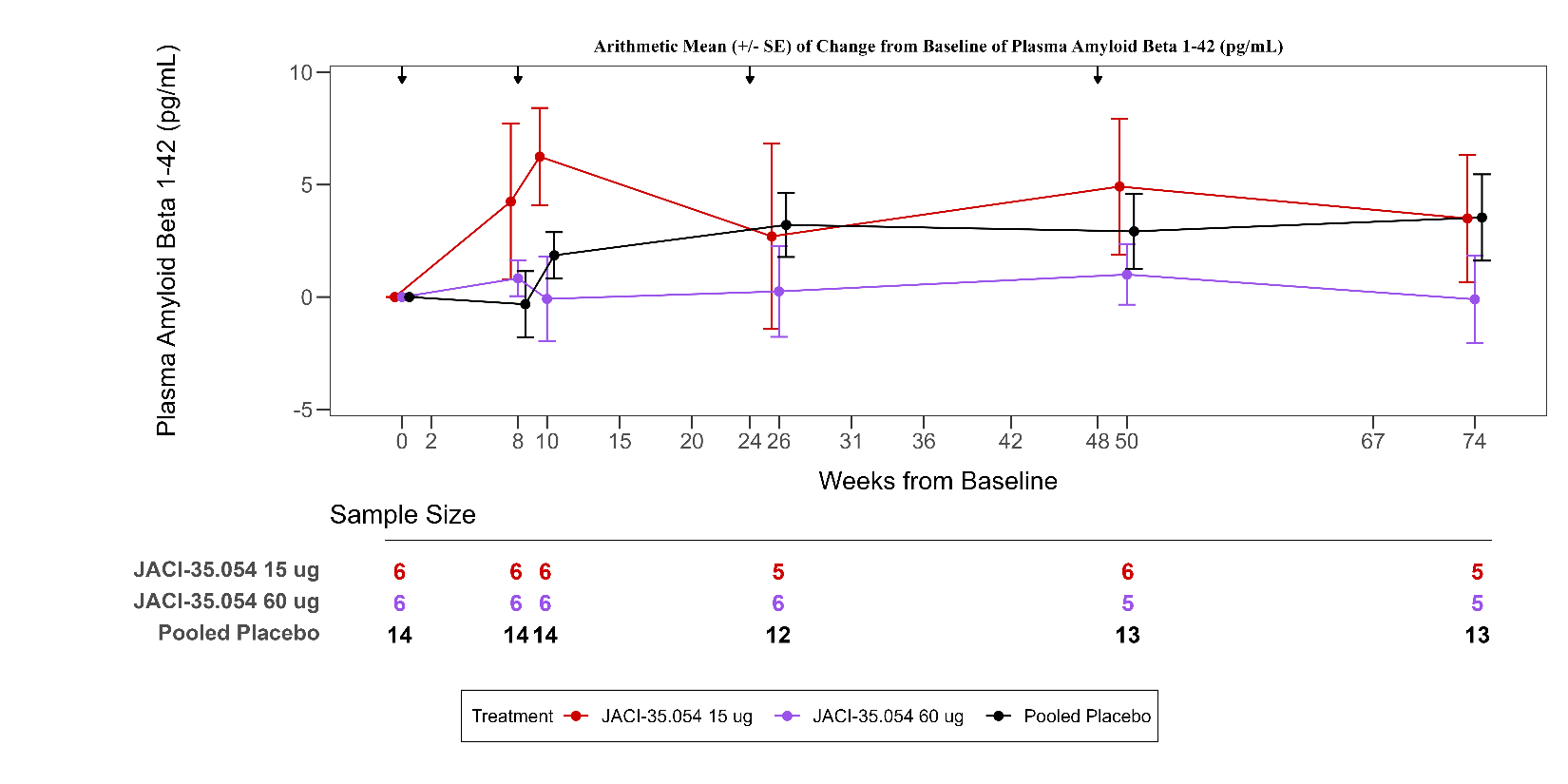
**Figure S10: Arithmetic mean of change from baseline of plasma Amyloid Beta 1-42 vs nominal visit time by study treatment arm (Cohort 2).** Error bars denote standard error of the mean. Number of subjects by nominal visit and study treatment arm tabulated below. The four vertical arrows at the top of the graph denote nominal study visit times for administration of JACI-35.054 or placebo.


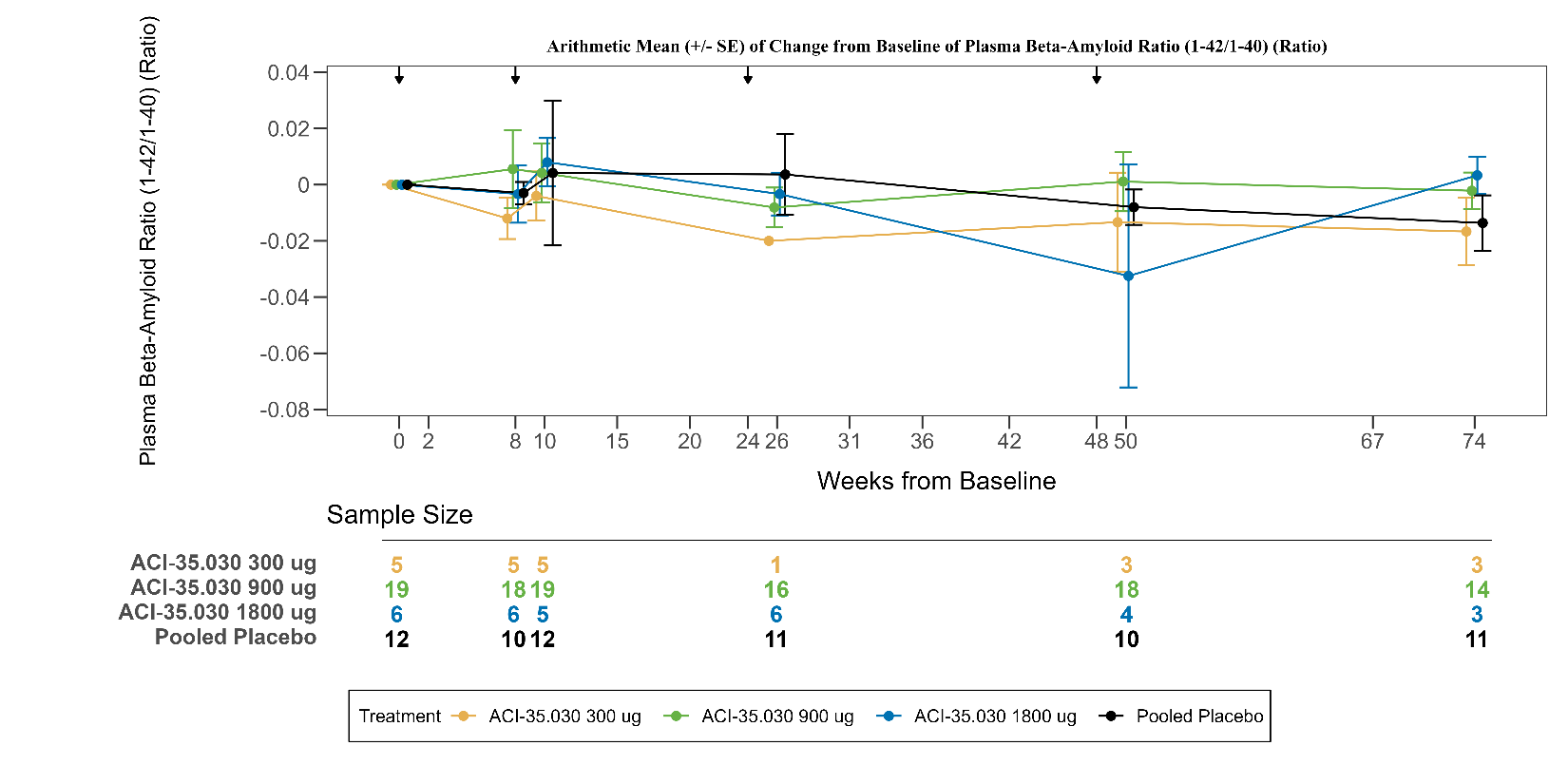
**Figure S11: Arithmetic mean of change from baseline of plasma Ratio of Amyloid Beta (1-42/1-40) vs nominal visit time by study treatment arm (Cohort 1).** Error bars denote standard error of the mean. Number of subjects by nominal visit and study treatment arm tabulated below. The four vertical arrows at the top of the graph denote nominal study visit times for administration of ACI-35.030 or placebo.


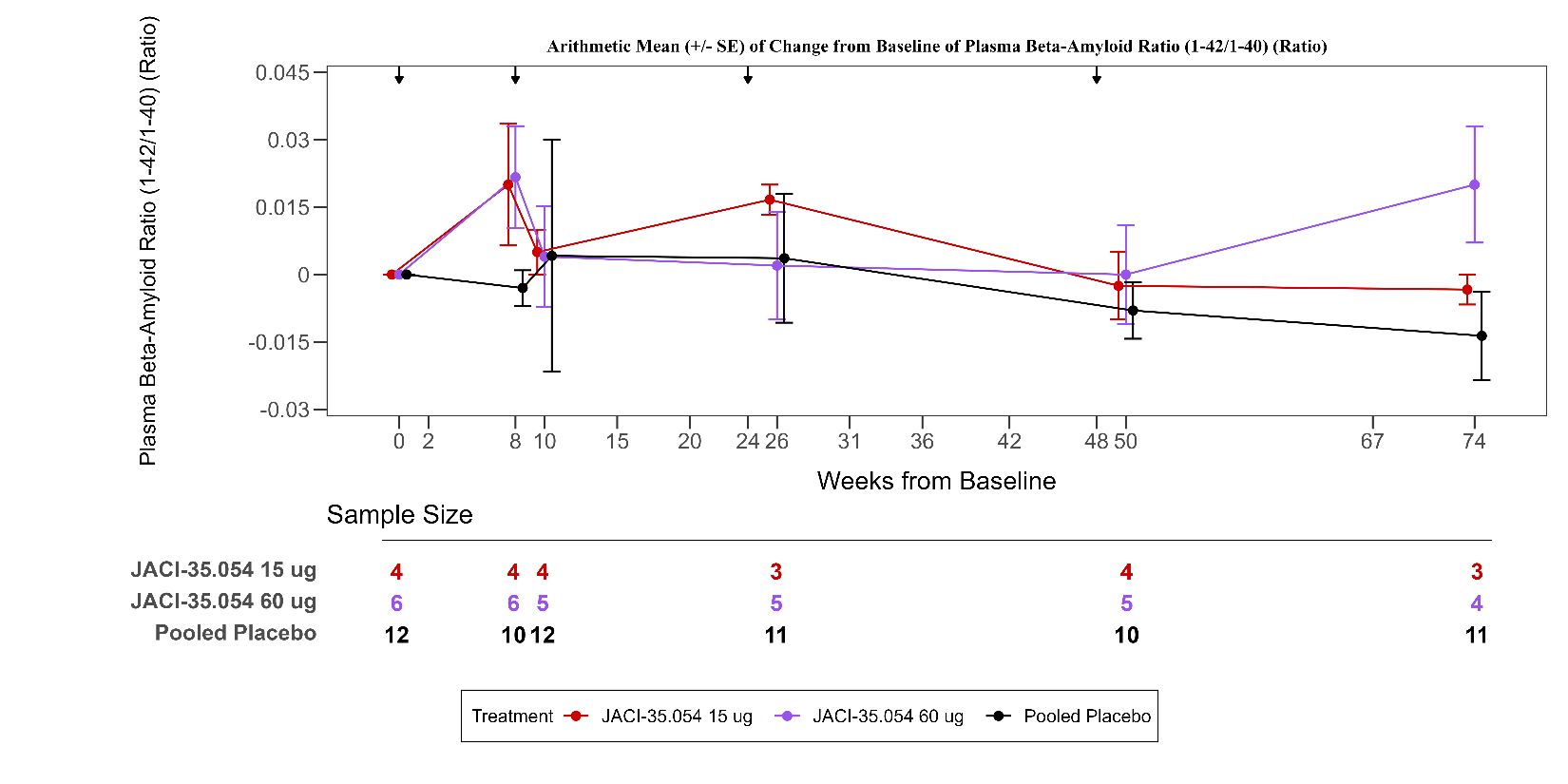
**Figure S12: Arithmetic mean of change from baseline of plasma Ratio of Amyloid Beta (1-42/1-40) vs nominal visit time by study treatment arm (Cohort 2).** Error bars denote standard error of the mean. Number of subjects by nominal visit and study treatment arm tabulated below. The four vertical arrows at the top of the graph denote nominal study visit times for administration of JACI-35.054 or placebo.


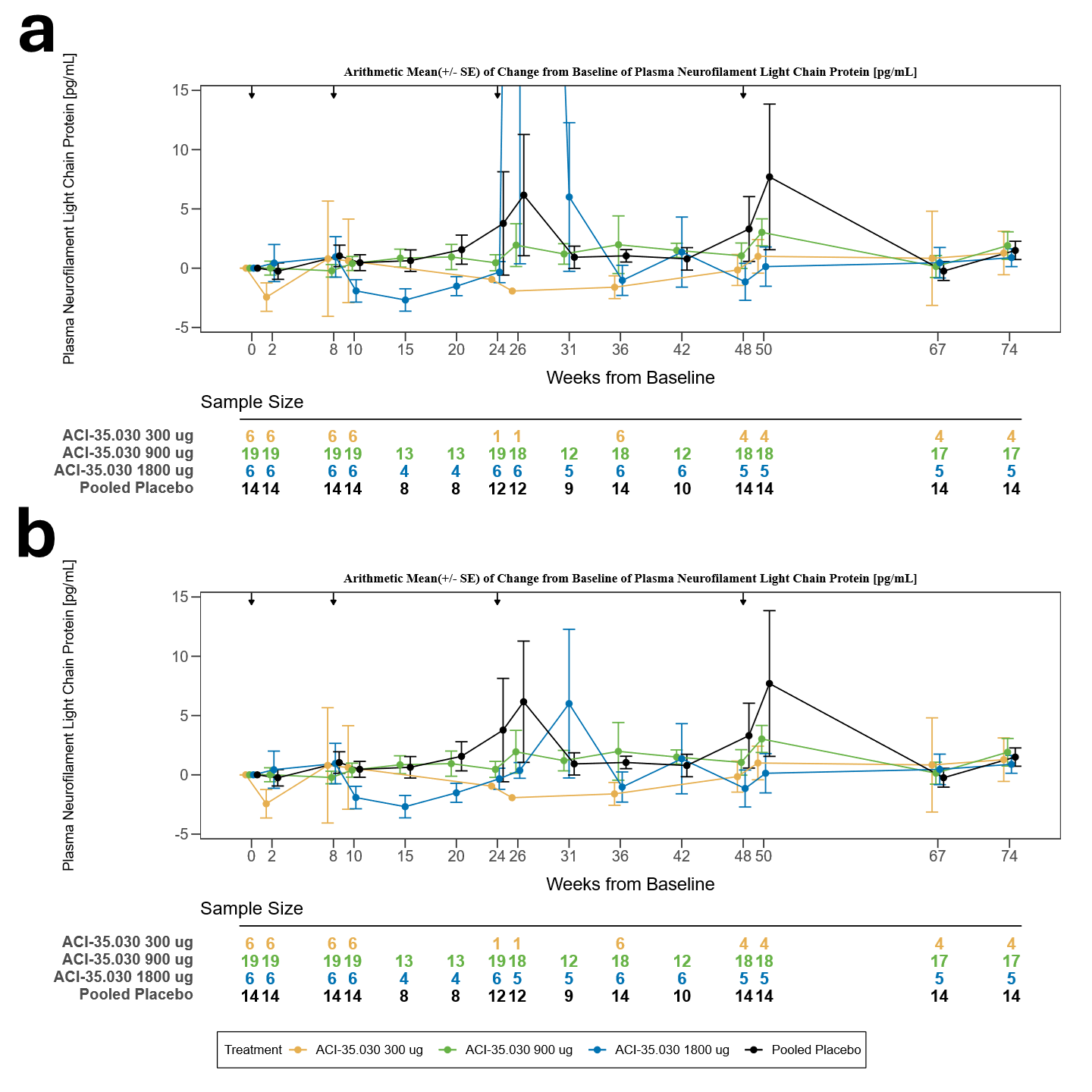
 **Figure S13: Arithmetic mean of change from baseline of plasma Neurofilament Light Chain Protein vs nominal visit time by study treatment arm (Cohort 1) including all subjects (a) and removing an outlier from the ACI-35.030 1800µg group at week 26 (b).** Error bars denote standard error of the mean. Number of subjects by nominal visit and study treatment arm tabulated below. The four vertical arrows at the top of the graph denote nominal study visit times for administration of ACI-35.030 or placebo.


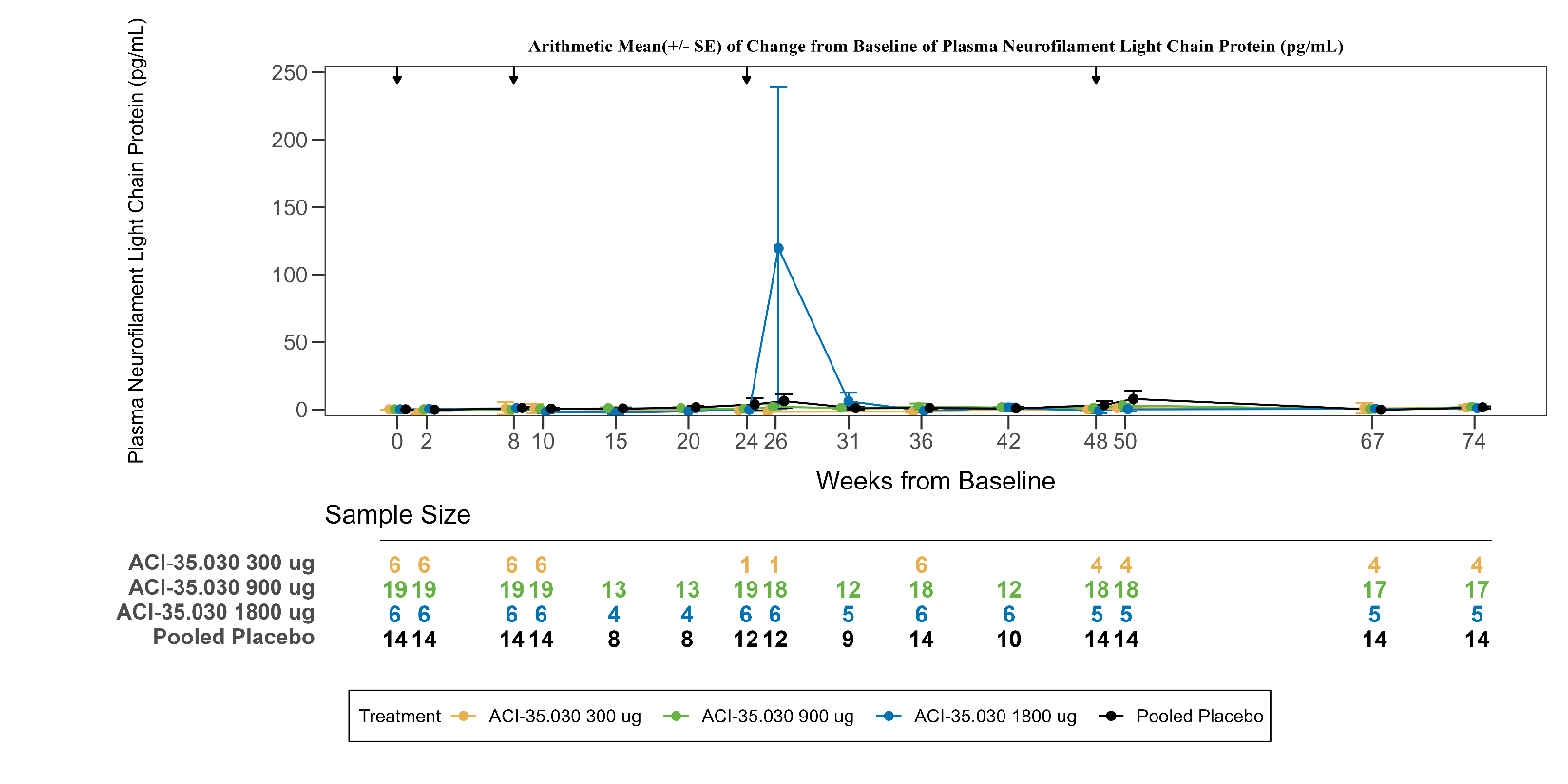
**Figure S14: Arithmetic mean of change from baseline of plasma Neurofilament Light Chain Protein vs nominal visit time by study treatment arm (Cohort 1).** Error bars denote standard error of the mean. Number of subjects by nominal visit and study treatment arm tabulated below. The four vertical arrows at the top of the graph denote nominal study visit times for administration of ACI-35.030 or placebo.


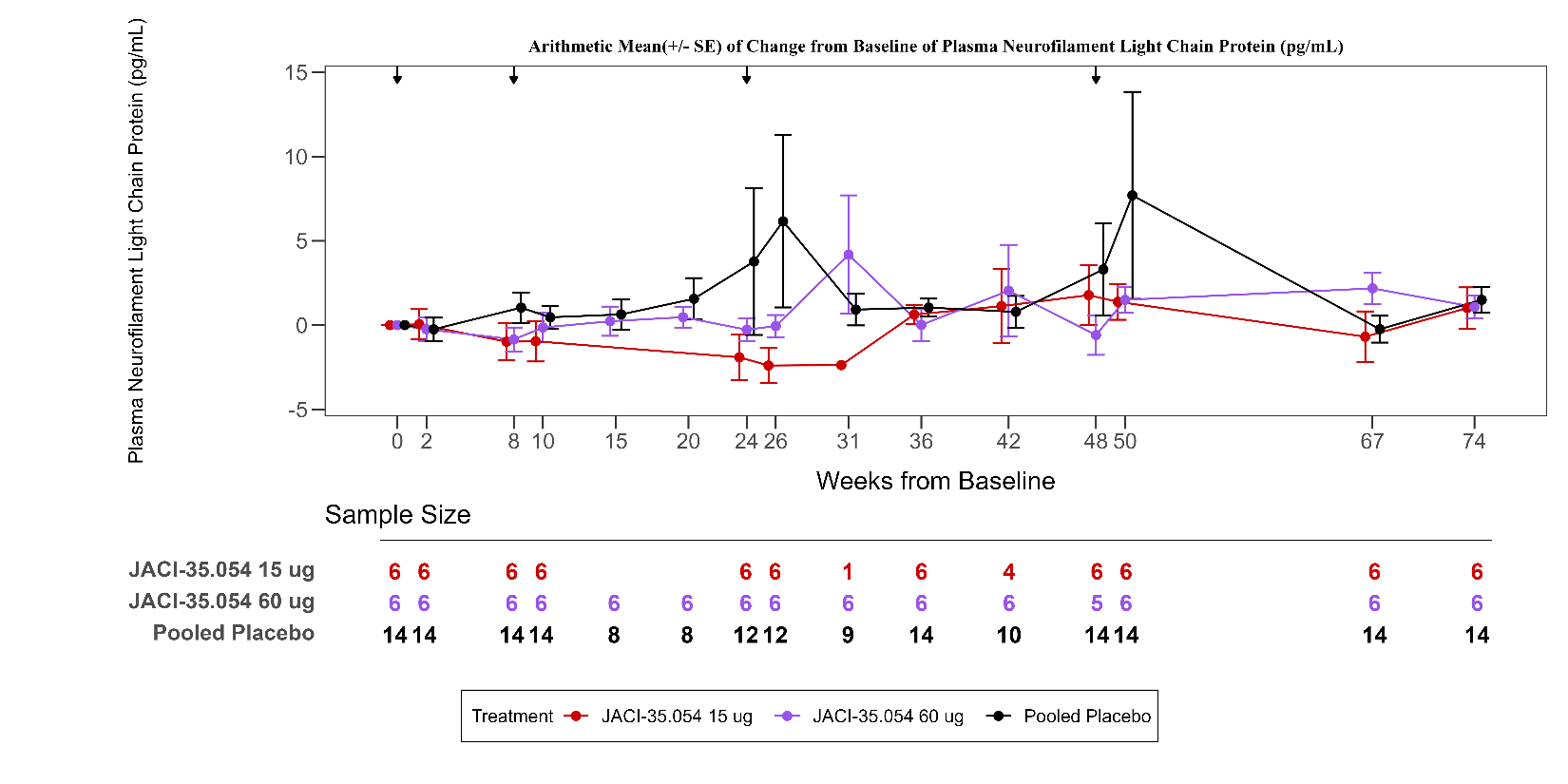
**Figure S15: Arithmetic mean of change from baseline of plasma Neurofilament Light Chain Protein vs nominal visit time by study treatment arm (Cohort 2).** Error bars denote standard error of the mean. Number of subjects by nominal visit and study treatment arm tabulated below. The four vertical arrows at the top of the graph denote nominal study visit times for administration of JACI-35.054 or placebo.


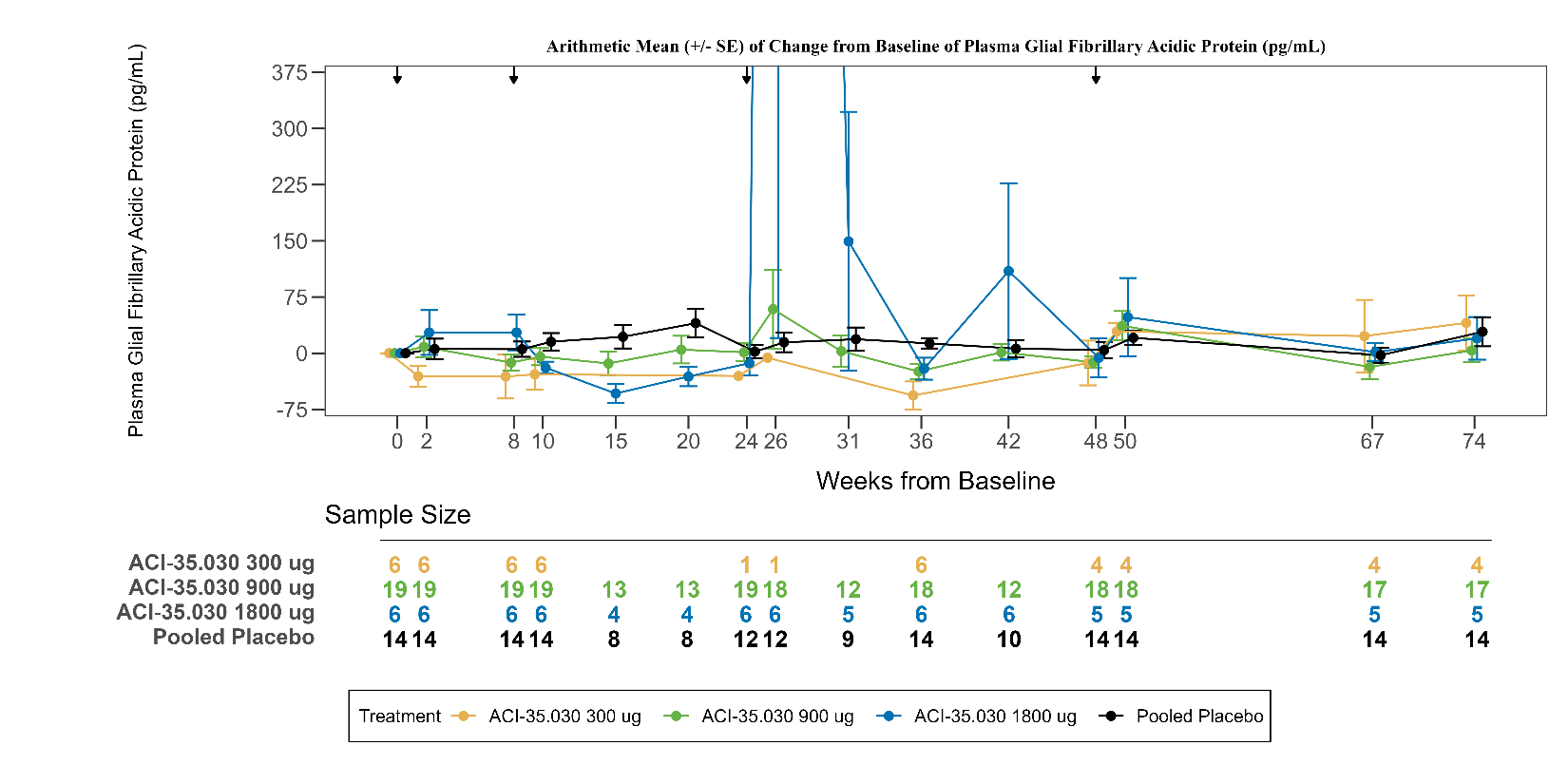
**Figure S16: Arithmetic mean of change from baseline of plasma Glial Fibrillary Acidic Protein vs nominal visit time by study treatment arm (Cohort 1).** Error bars denote standard error of the mean. Number of subjects by nominal visit and study treatment arm tabulated below. The four vertical arrows at the top of the graph denote nominal study visit times for administration of ACI-35.030 or placebo.


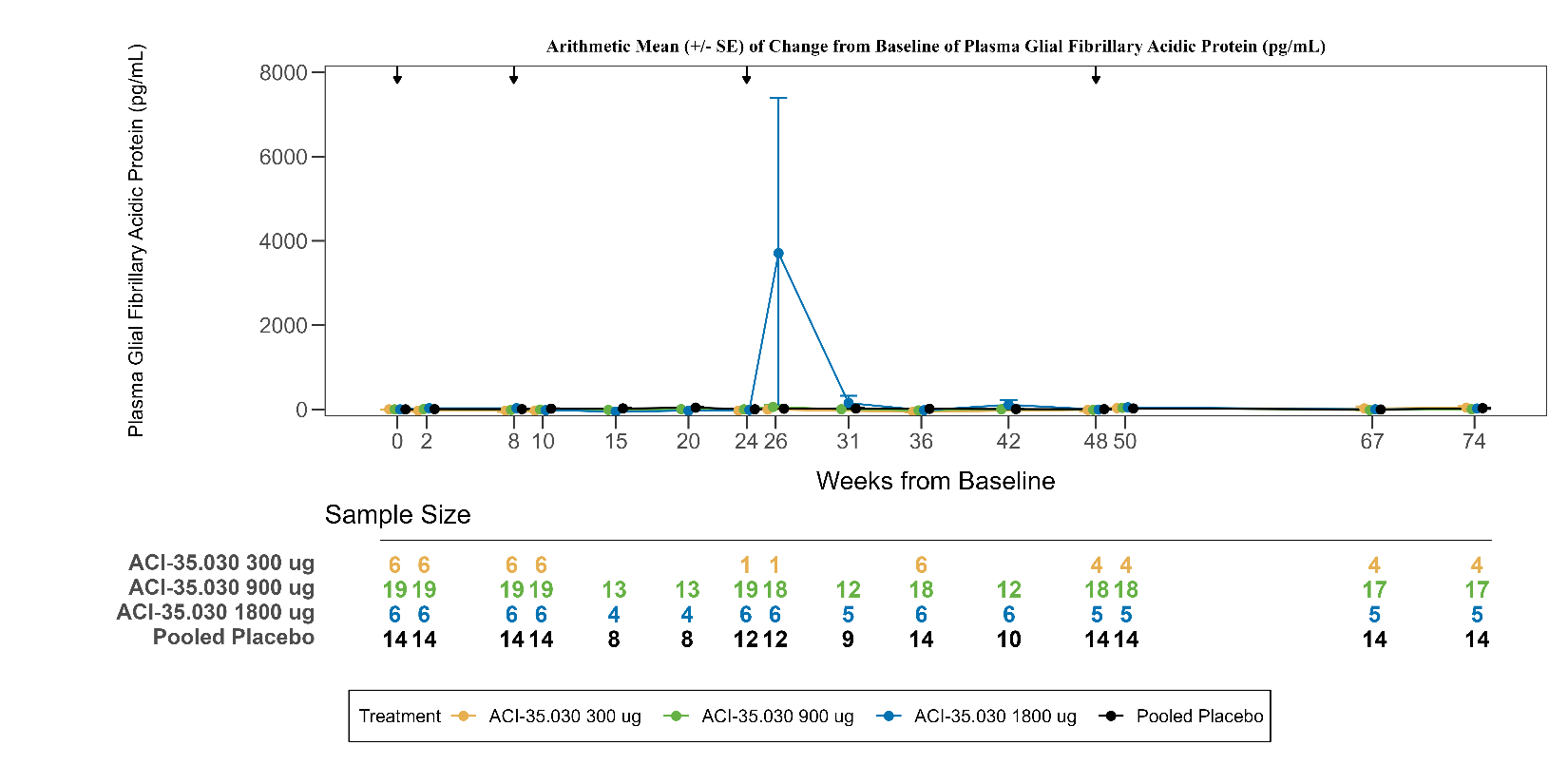
**Figure S17: Arithmetic mean of change from baseline of plasma Glial Fibrillary Acidic Protein vs nominal visit time by study treatment arm (Cohort 1).** Error bars denote standard error of the mean. Number of subjects by nominal visit and study treatment arm tabulated below. The four vertical arrows at the top of the graph denote nominal study visit times for administration of ACI-35.030 or placebo.


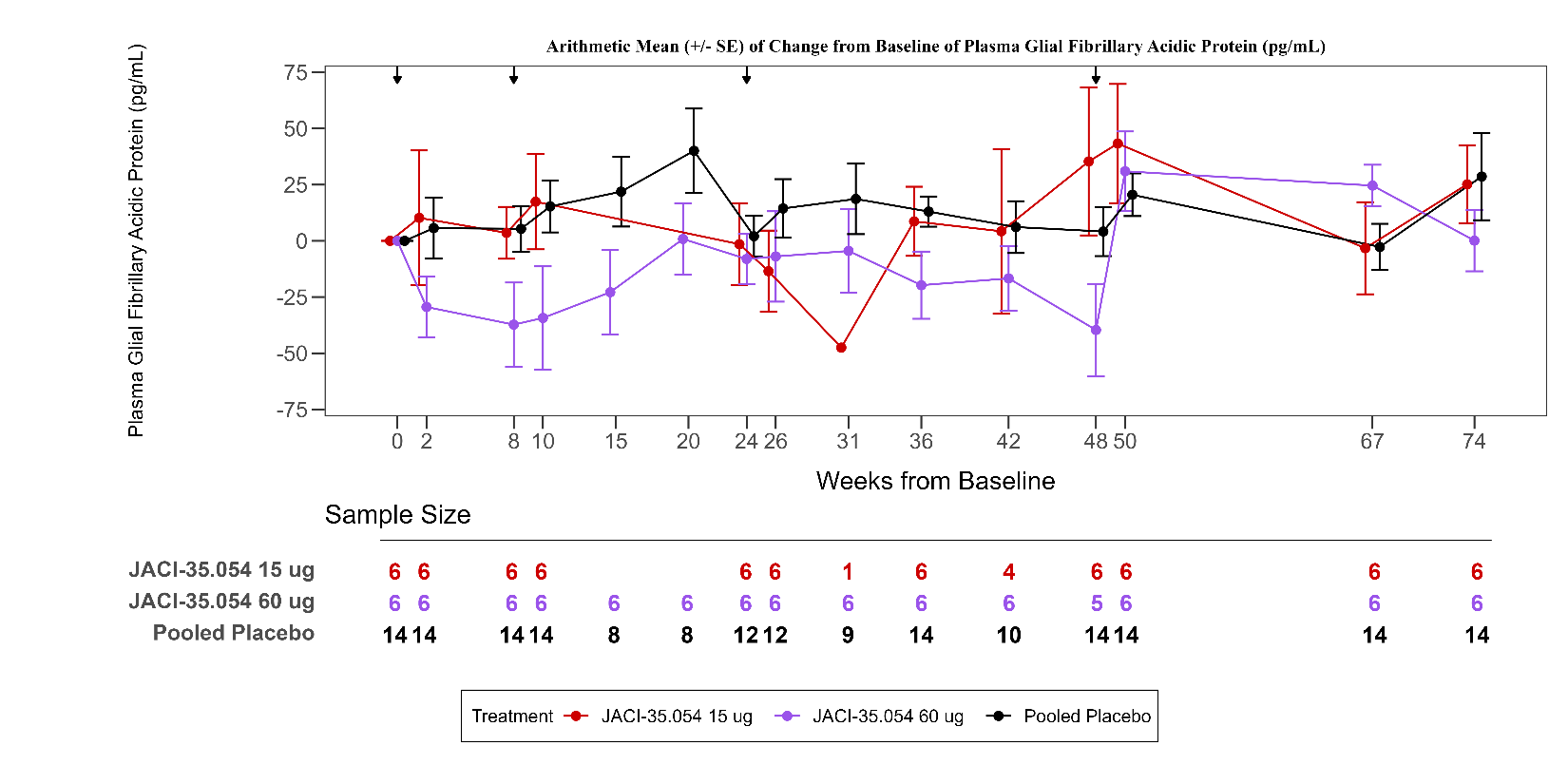
**Figure S18: Arithmetic mean of change from baseline of plasma Glial Fibrillary Acidic Protein vs nominal visit time by study treatment arm (Cohort 2).** Error bars denote standard error of the mean. Number of subjects by nominal visit and study treatment arm tabulated below. The four vertical arrows at the top of the graph denote nominal study visit times for administration of JACI-35.054 or placebo.


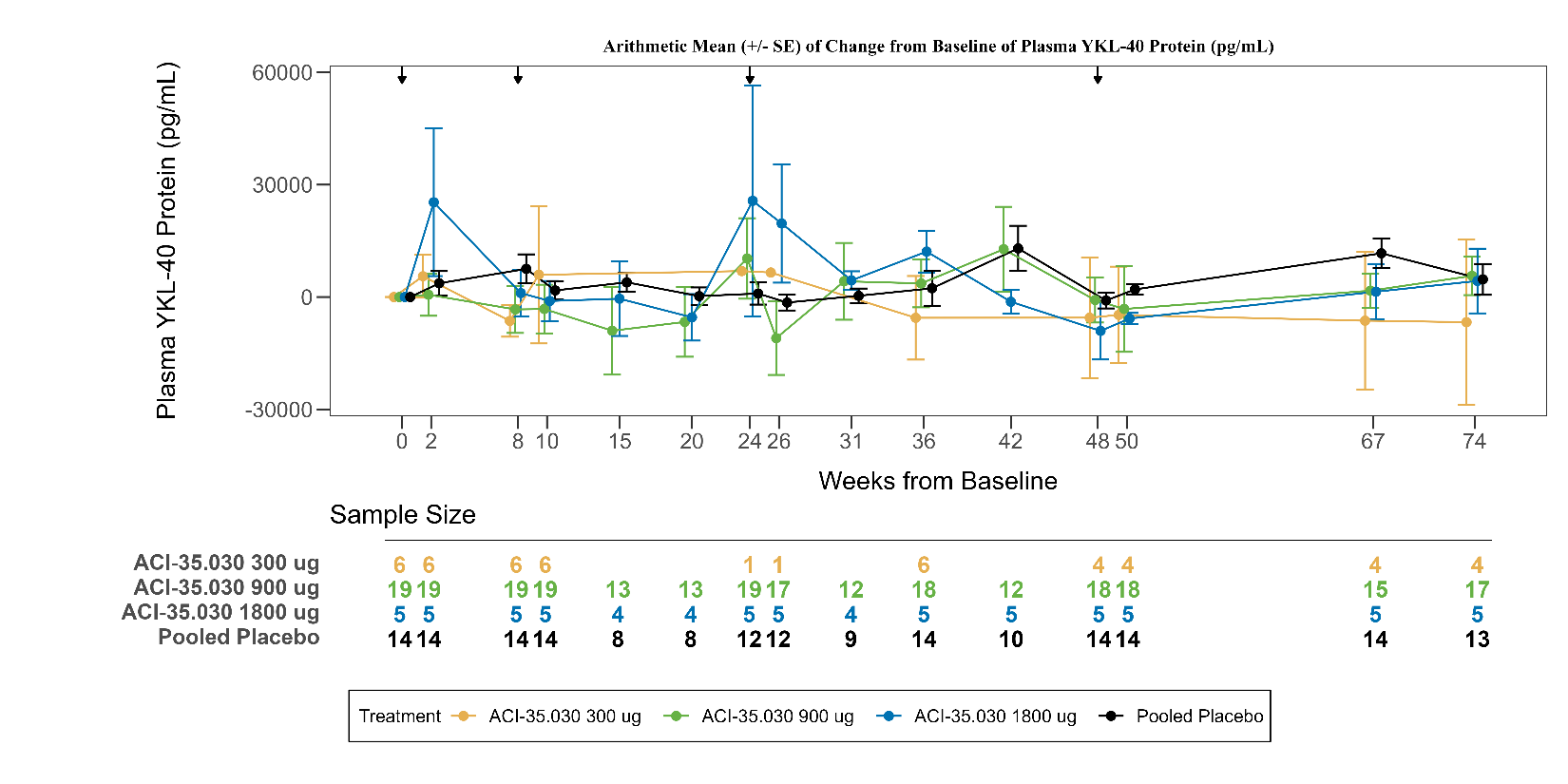
**Figure S19: Arithmetic mean of change from baseline of plasma YKL-40 Protein vs nominal visit time by study treatment arm (Cohort 1).** Error bars denote standard error of the mean. Number of subjects by nominal visit and study treatment arm tabulated below. The four vertical arrows at the top of the graph denote nominal study visit times for administration of ACI-35.030 or placebo.


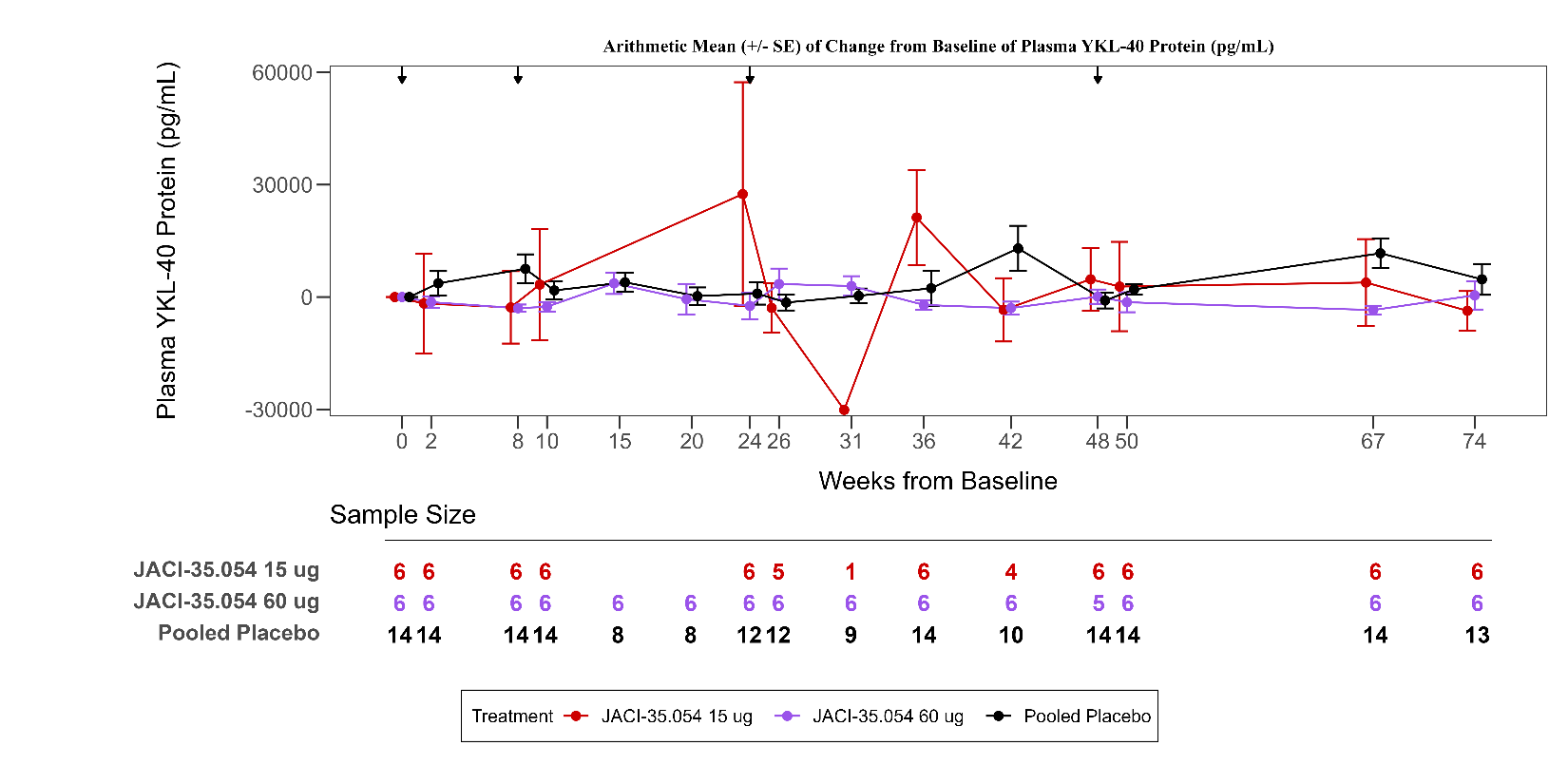
**Figure S20: Arithmetic mean of change from baseline of plasma YKL-40 Protein vs nominal visit time by study treatment arm (Cohort 2).** Error bars denote standard error of the mean. Number of subjects by nominal visit and study treatment arm tabulated below. The four vertical arrows at the top of the graph denote nominal study visit times for administration of JACI-35.054 or placebo.


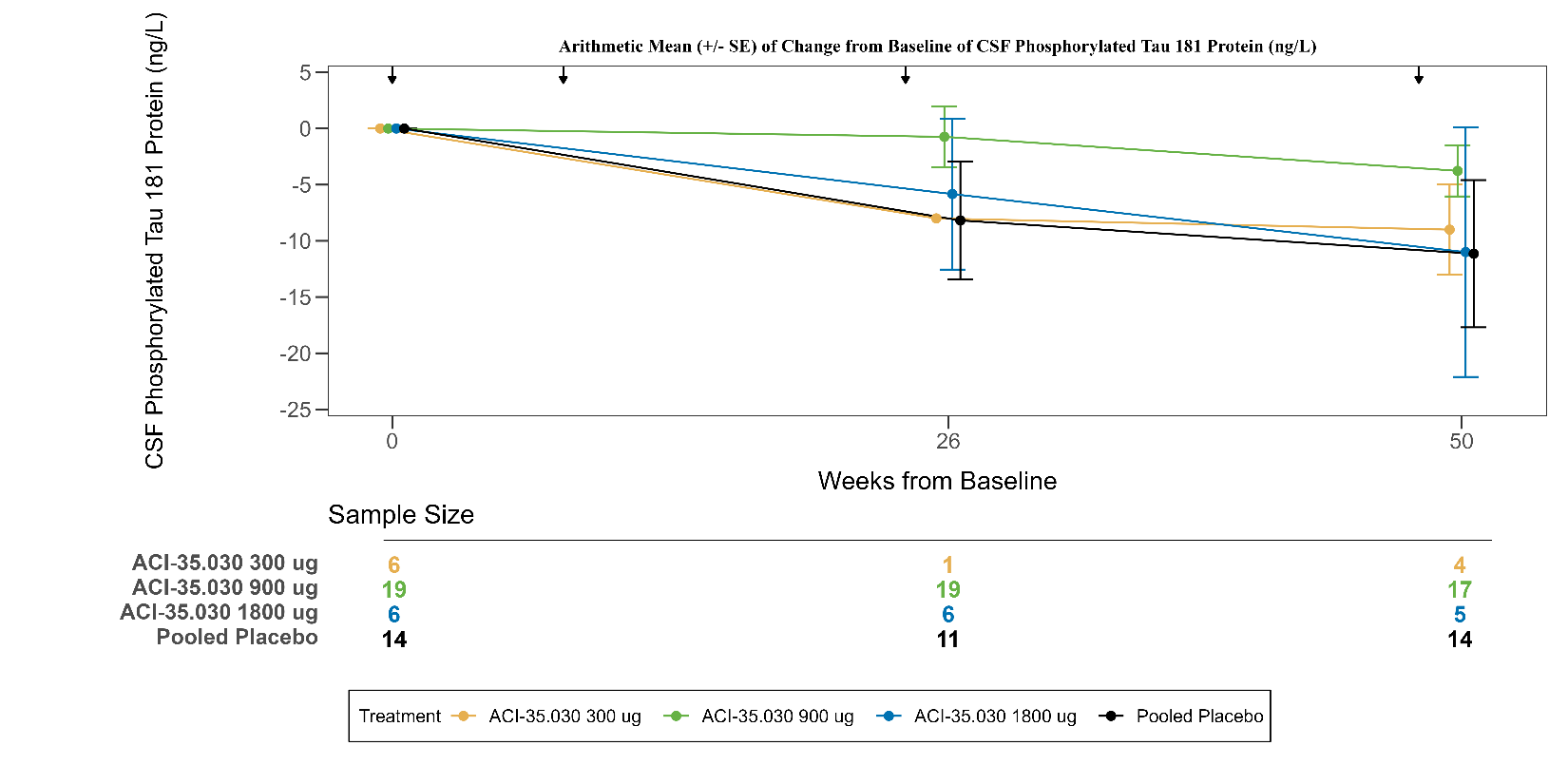
**Figure S21: Arithmetic mean of change from baseline of CSF Phosphorylated Tau 181 Protein vs nominal visit time by study treatment arm (Cohort 1).** Error bars denote standard error of the mean. Number of subjects by nominal visit and study treatment arm tabulated below. The four vertical arrows at the top of the graph denote nominal study visit times for administration of ACI-35.030 or placebo.


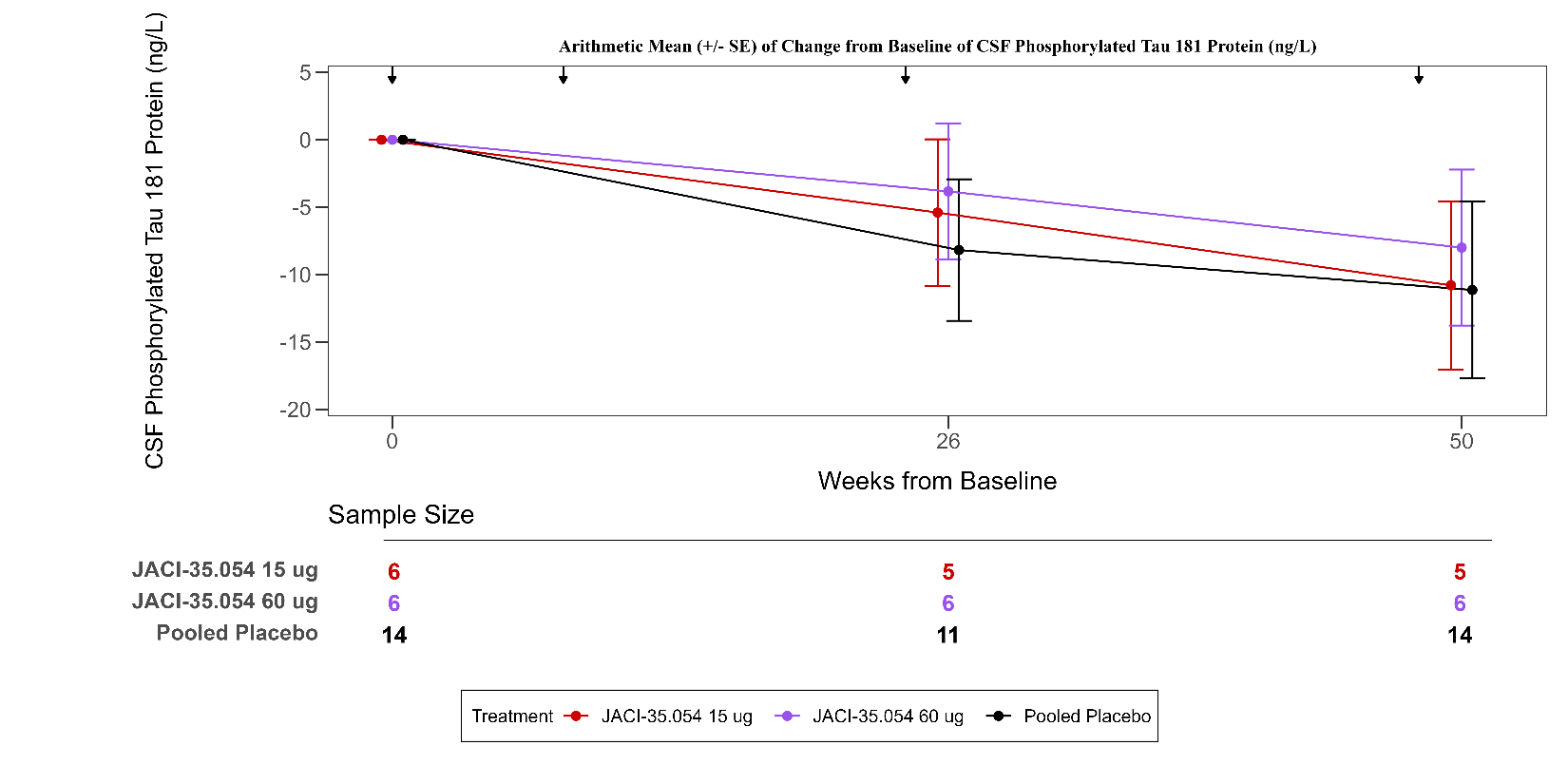
**Figure S22: Arithmetic mean of change from baseline of CSF Phosphorylated Tau 181 Protein vs nominal visit time by study treatment arm (Cohort 2).** Error bars denote standard error of the mean. Number of subjects by nominal visit and study treatment arm tabulated below. The four vertical arrows at the top of the graph denote nominal study visit times for administration of JACI-35.054 or placebo.


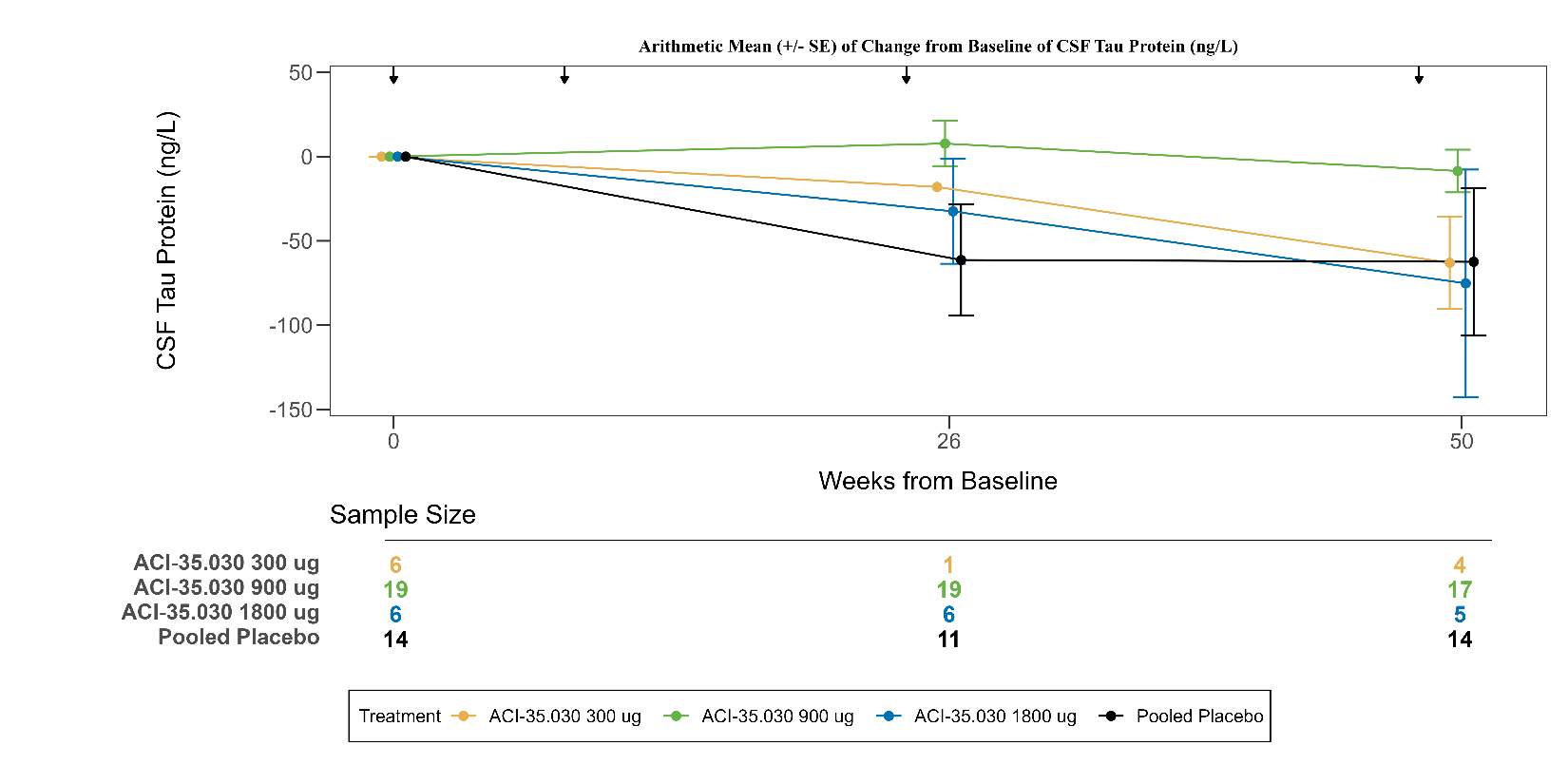
**Figure S23: Arithmetic mean of change from baseline of CSF Total Tau Protein vs nominal visit time by study treatment arm (Cohort 1).** Error bars denote standard error of the mean. Number of subjects by nominal visit and study treatment arm tabulated below. The four vertical arrows at the top of the graph denote nominal study visit times for administration of ACI-35.030 or placebo.


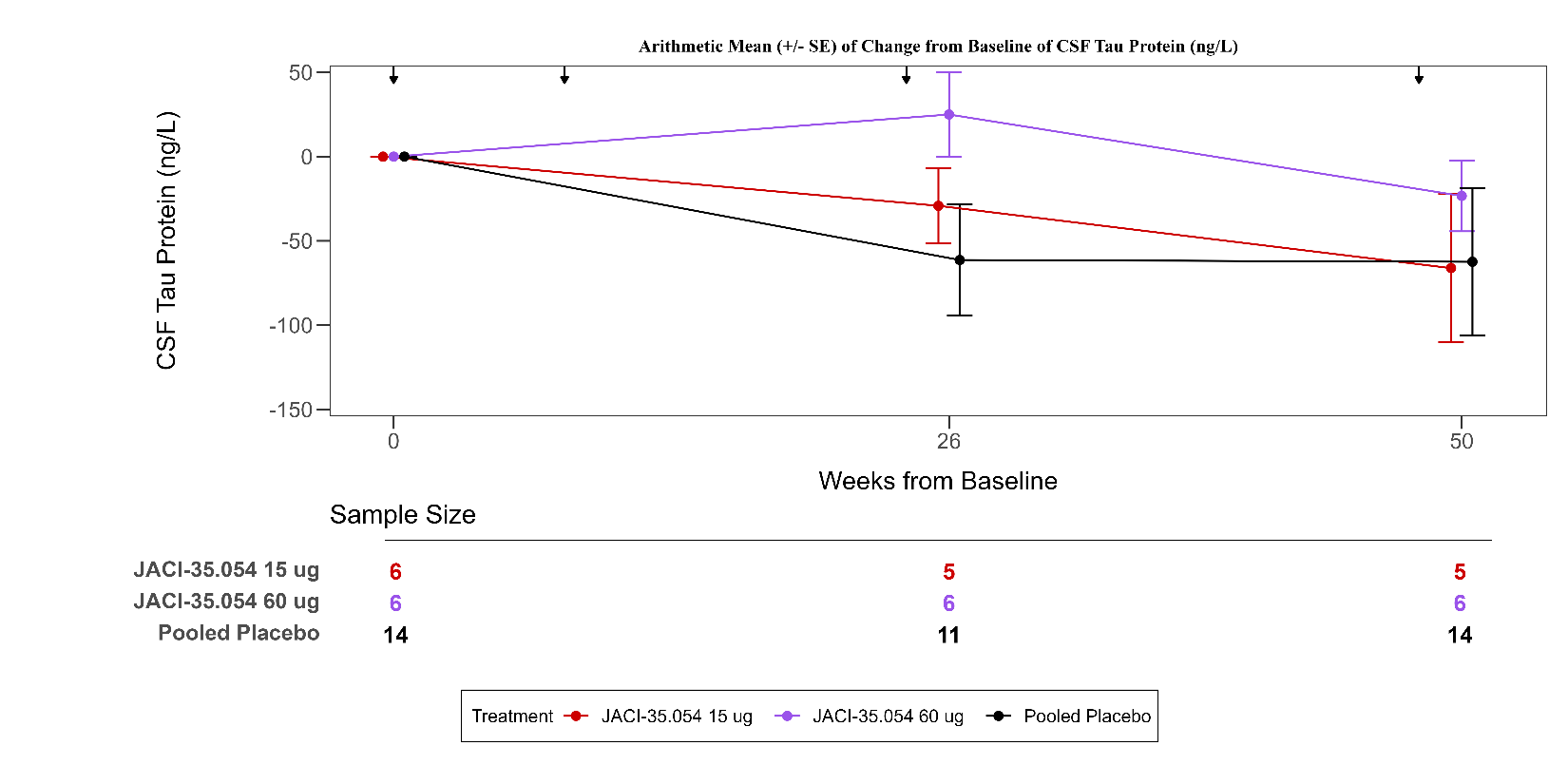
**Figure S24: Arithmetic mean of change from baseline of CSF Total Tau Protein vs nominal visit time by study treatment arm (Cohort 2).** Error bars denote standard error of the mean. Number of subjects by nominal visit and study treatment arm tabulated below. The four vertical arrows at the top of the graph denote nominal study visit times for administration of JACI-35.054 or placebo.


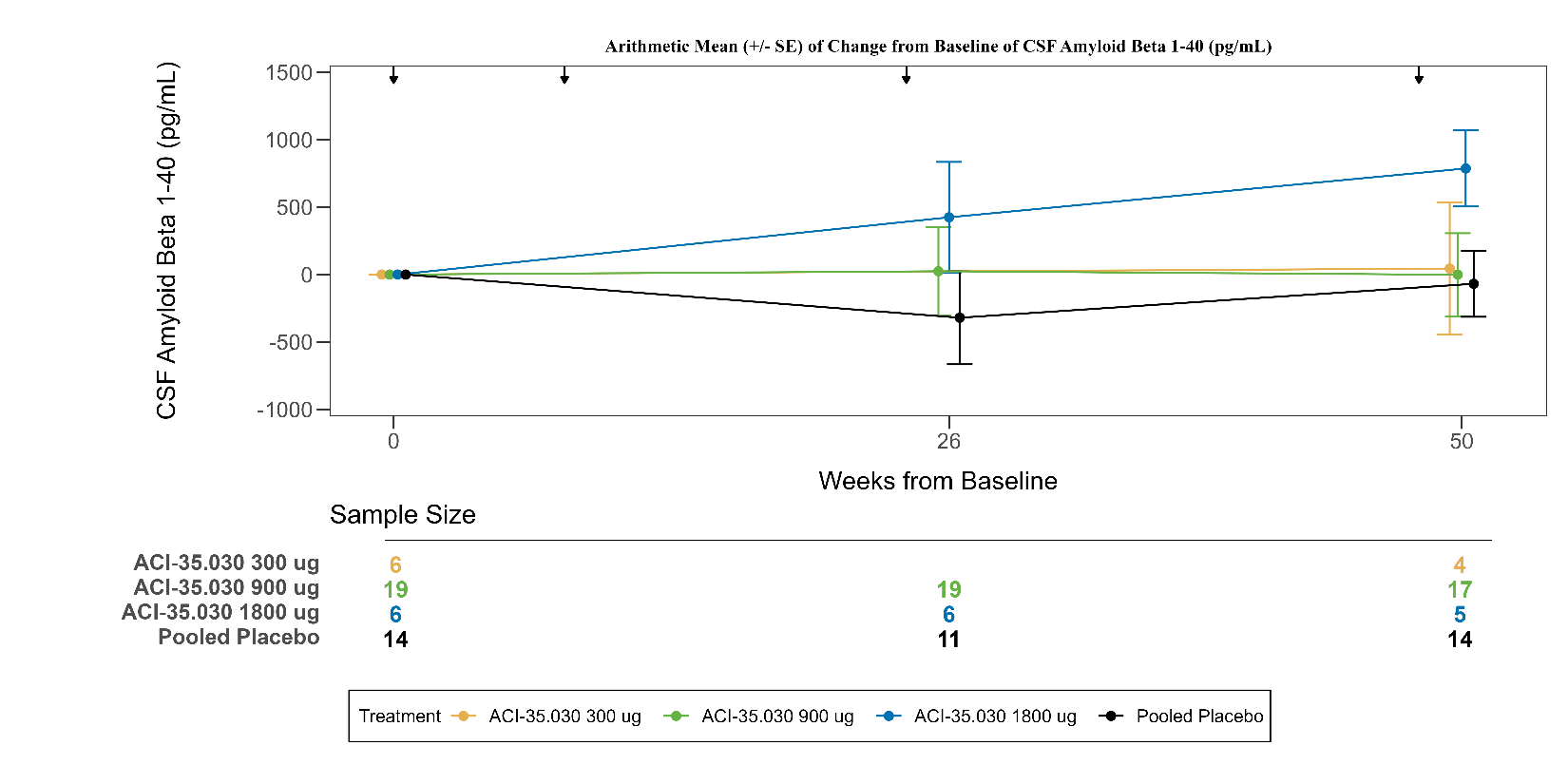
**Figure S25: Arithmetic mean of change from baseline of CSF Amyloid Beta 1-40 vs nominal visit time by study treatment arm (Cohort 1).** Error bars denote standard error of the mean. Number of subjects by nominal visit and study treatment arm tabulated below. The four vertical arrows at the top of the graph denote nominal study visit times for administration of ACI-35.030 or placebo.


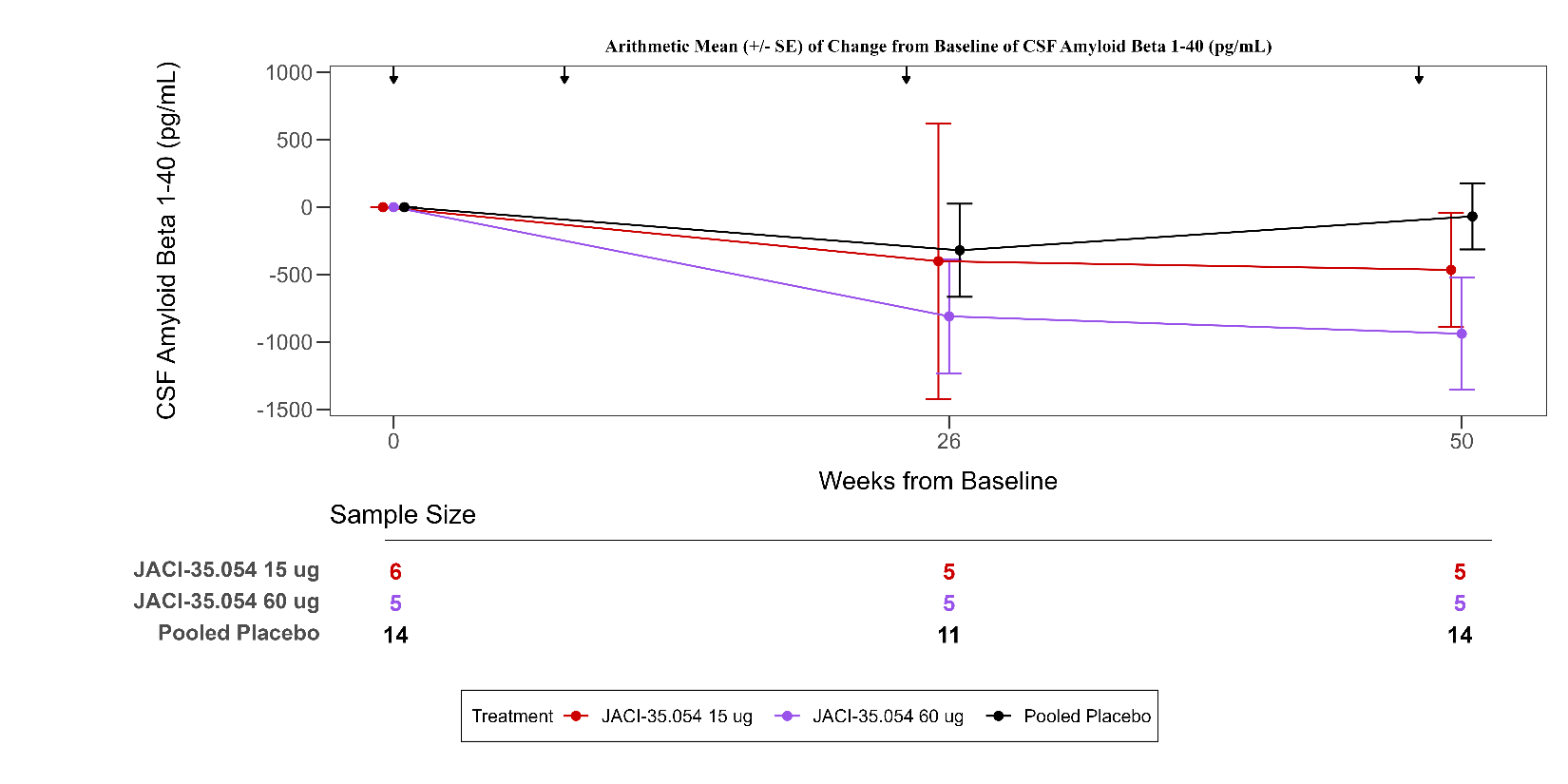
**Figure S26: Arithmetic mean of change from baseline of CSF Amyloid Beta 1-40 vs nominal visit time by study treatment arm (Cohort 2).** Error bars denote standard error of the mean. Number of subjects by nominal visit and study treatment arm tabulated below. The four vertical arrows at the top of the graph denote nominal study visit times for administration of JACI-35.054 or placebo.


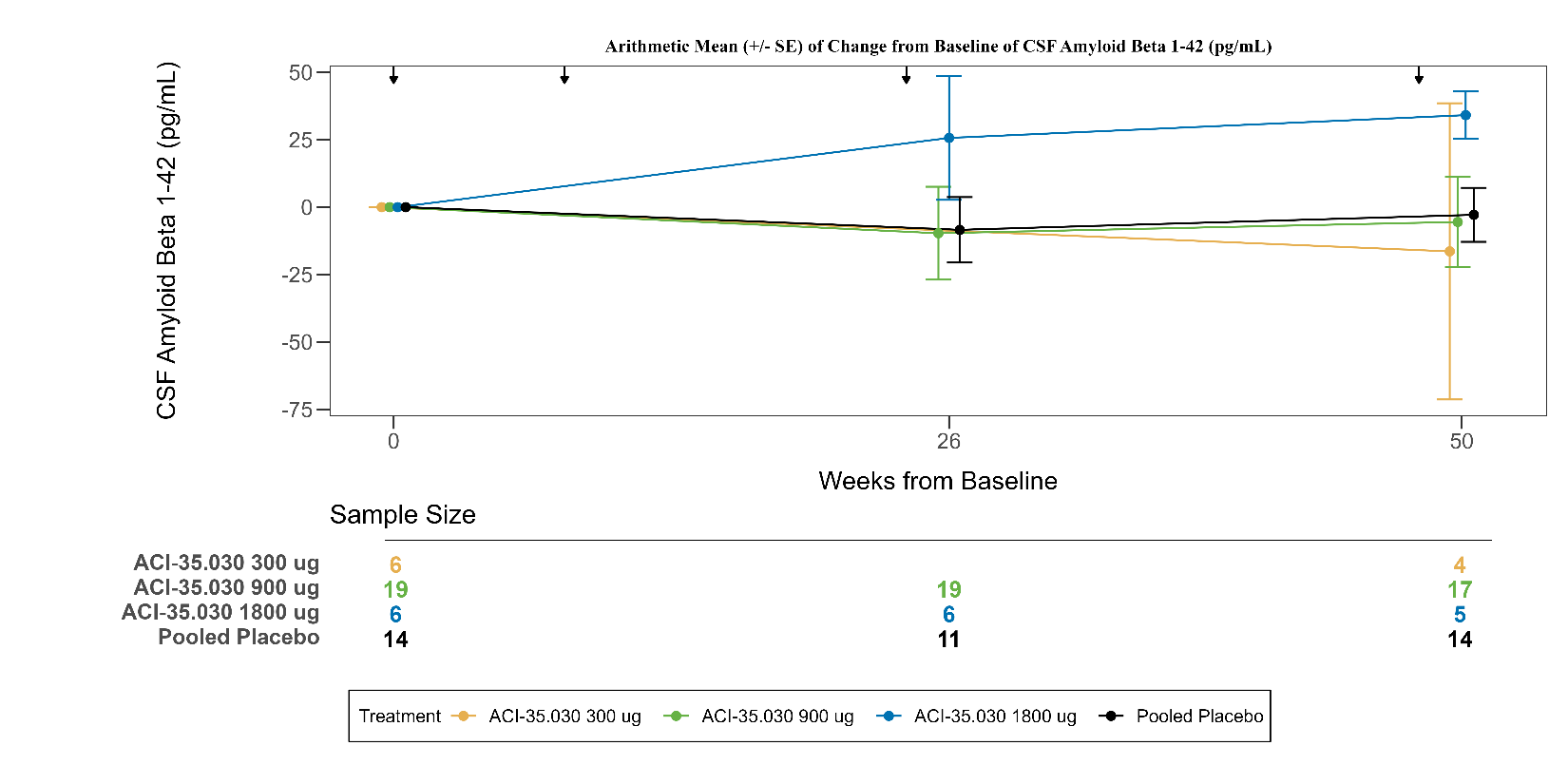
**Figure S27: Arithmetic mean of change from baseline of CSF Amyloid Beta 1-42 vs nominal visit time by study treatment arm (Cohort 1).** Error bars denote standard error of the mean. Number of subjects by nominal visit and study treatment arm tabulated below. The four vertical arrows at the top of the graph denote nominal study visit times for administration of ACI-35.030 or placebo.


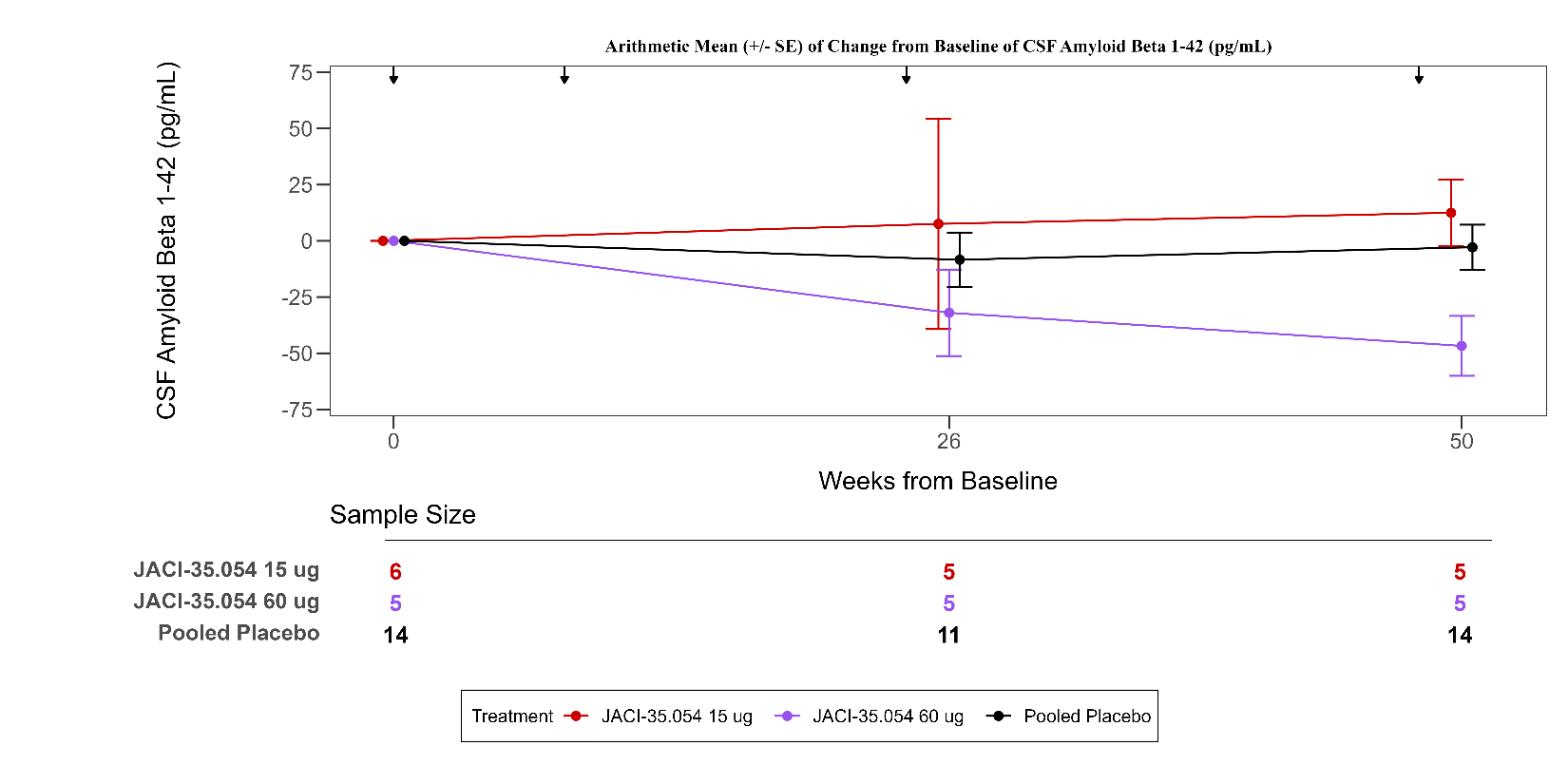
**Figure S28: Arithmetic mean of change from baseline of CSF Amyloid Beta 1-42 vs nominal visit time by study treatment arm (Cohort 2).** Error bars denote standard error of the mean. Number of subjects by nominal visit and study treatment arm tabulated below. The four vertical arrows at the top of the graph denote nominal study visit times for administration of JACI-35.054 or placebo.


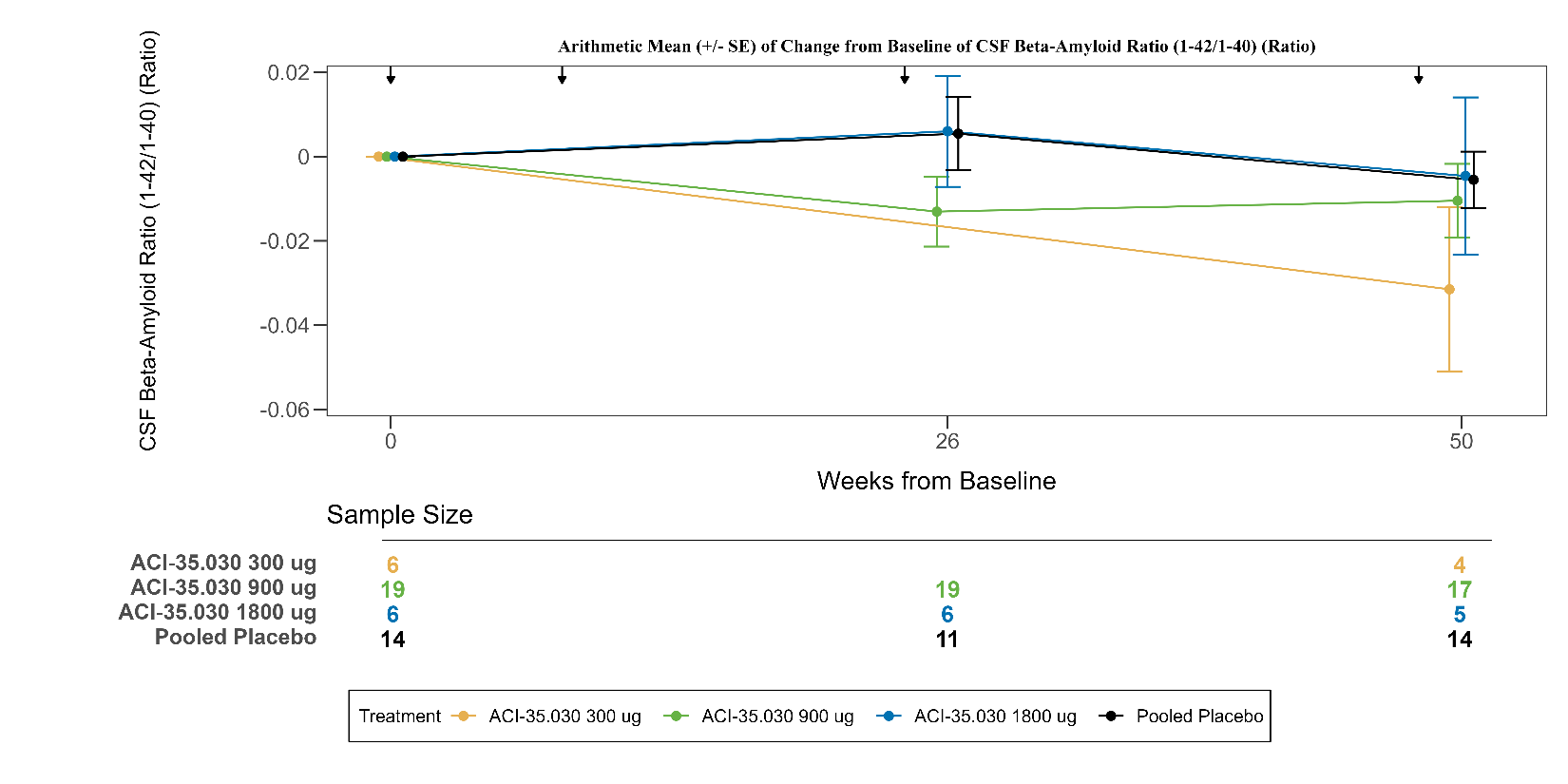
**Figure S29: Arithmetic mean of change from baseline of CSF Ratio of Amyloid Beta (1-42/1-40) vs nominal visit time by study treatment arm (Cohort 1).** Error bars denote standard error of the mean. Number of subjects by nominal visit and study treatment arm tabulated below. The four vertical arrows at the top of the graph denote nominal study visit times for administration of ACI-35.030 or placebo.


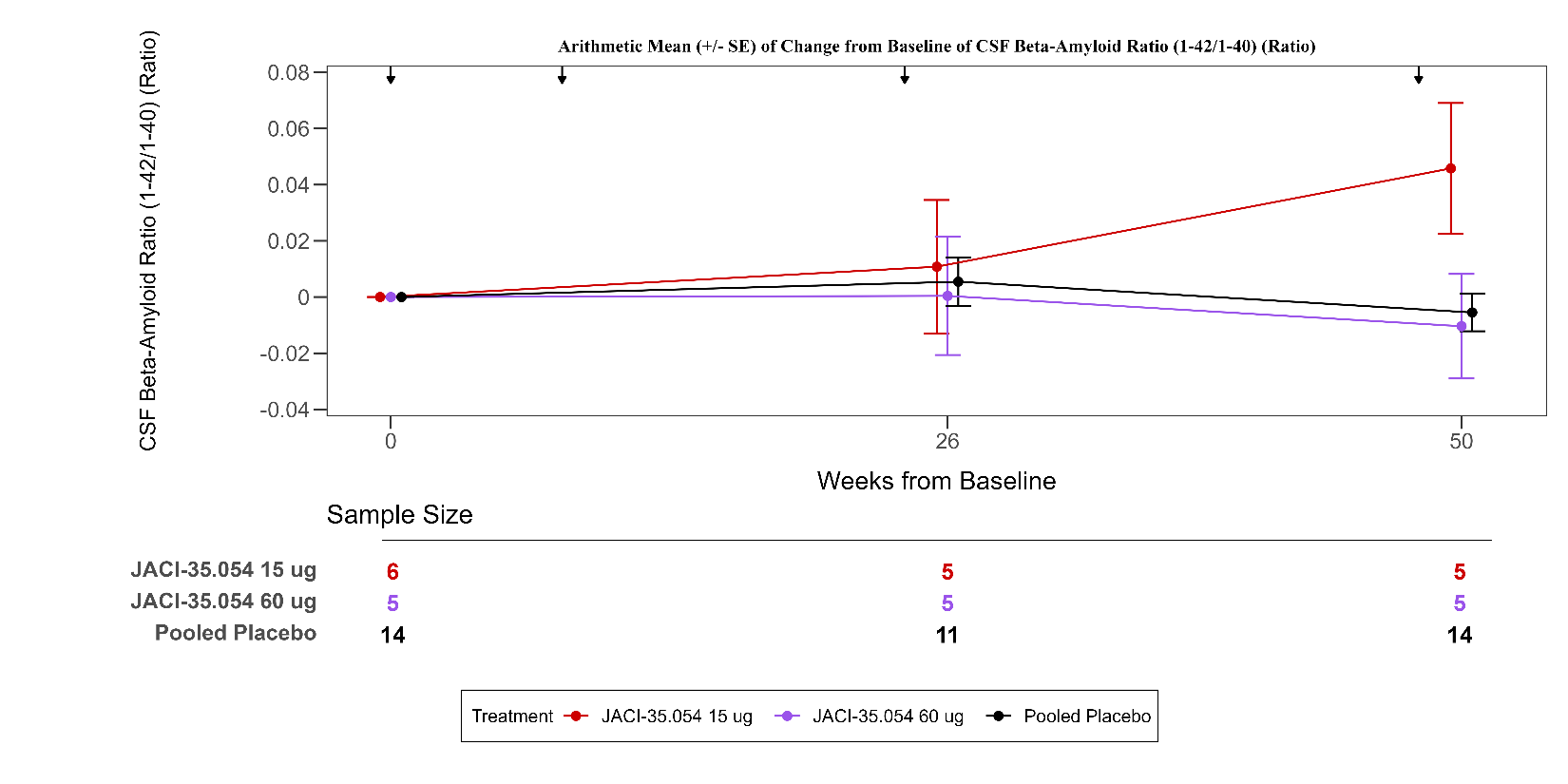
**Figure S30: Arithmetic mean of change from baseline of CSF Ratio of Amyloid Beta (1-42/1-40) vs nominal visit time by study treatment arm (Cohort 2).** Error bars denote standard error of the mean. Number of subjects by nominal visit and study treatment arm tabulated below. The four vertical arrows at the top of the graph denote nominal study visit times for administration of JACI-35.054 or placebo.


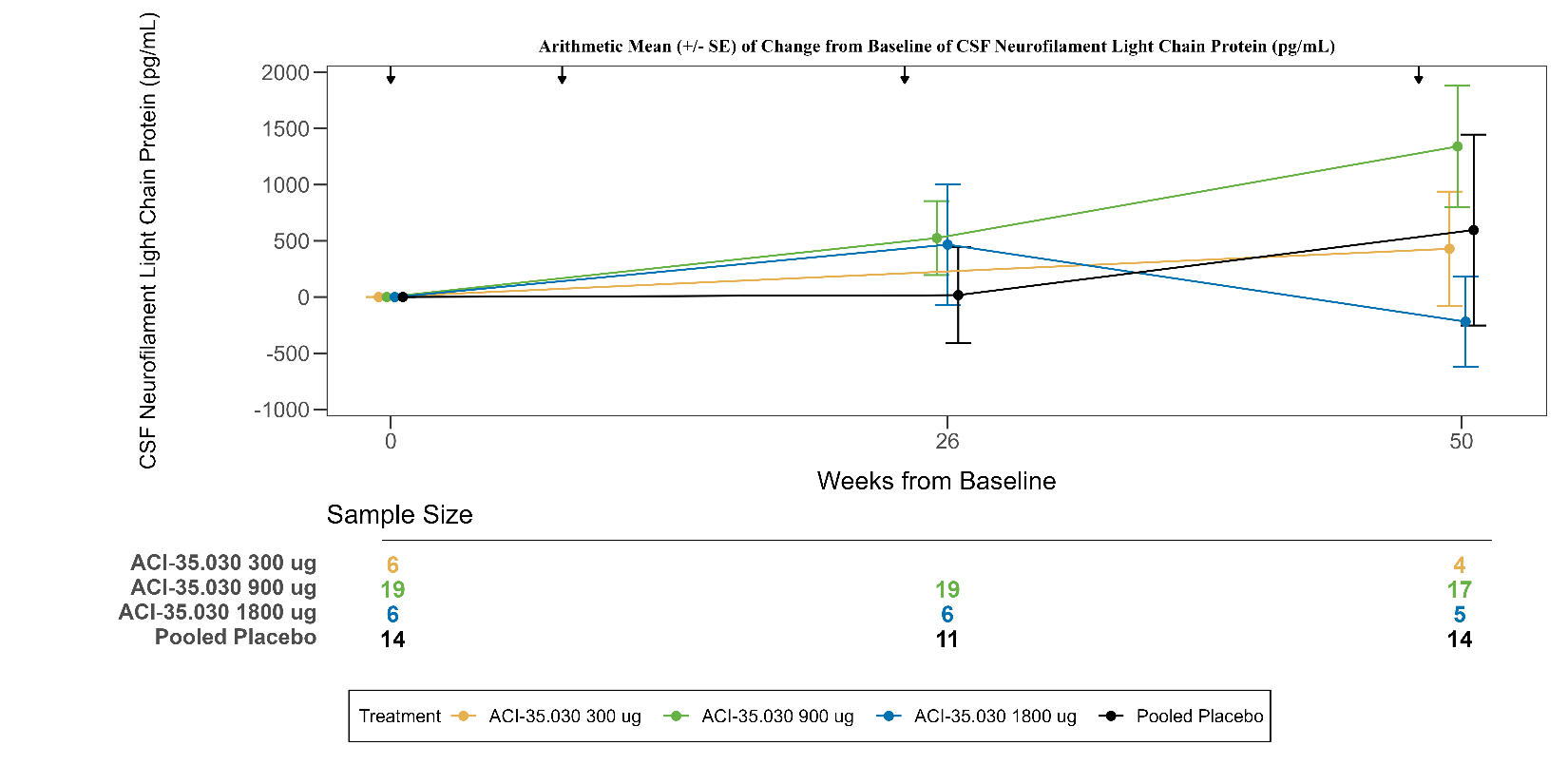
**Figure S31: Arithmetic mean of change from baseline of CSF Neurofilament Light Chain Protein vs nominal visit time by study treatment arm (Cohort 1).** Error bars denote standard error of the mean. Number of subjects by nominal visit and study treatment arm tabulated below. The four vertical arrows at the top of the graph denote nominal study visit times for administration of ACI-35.030 or placebo.


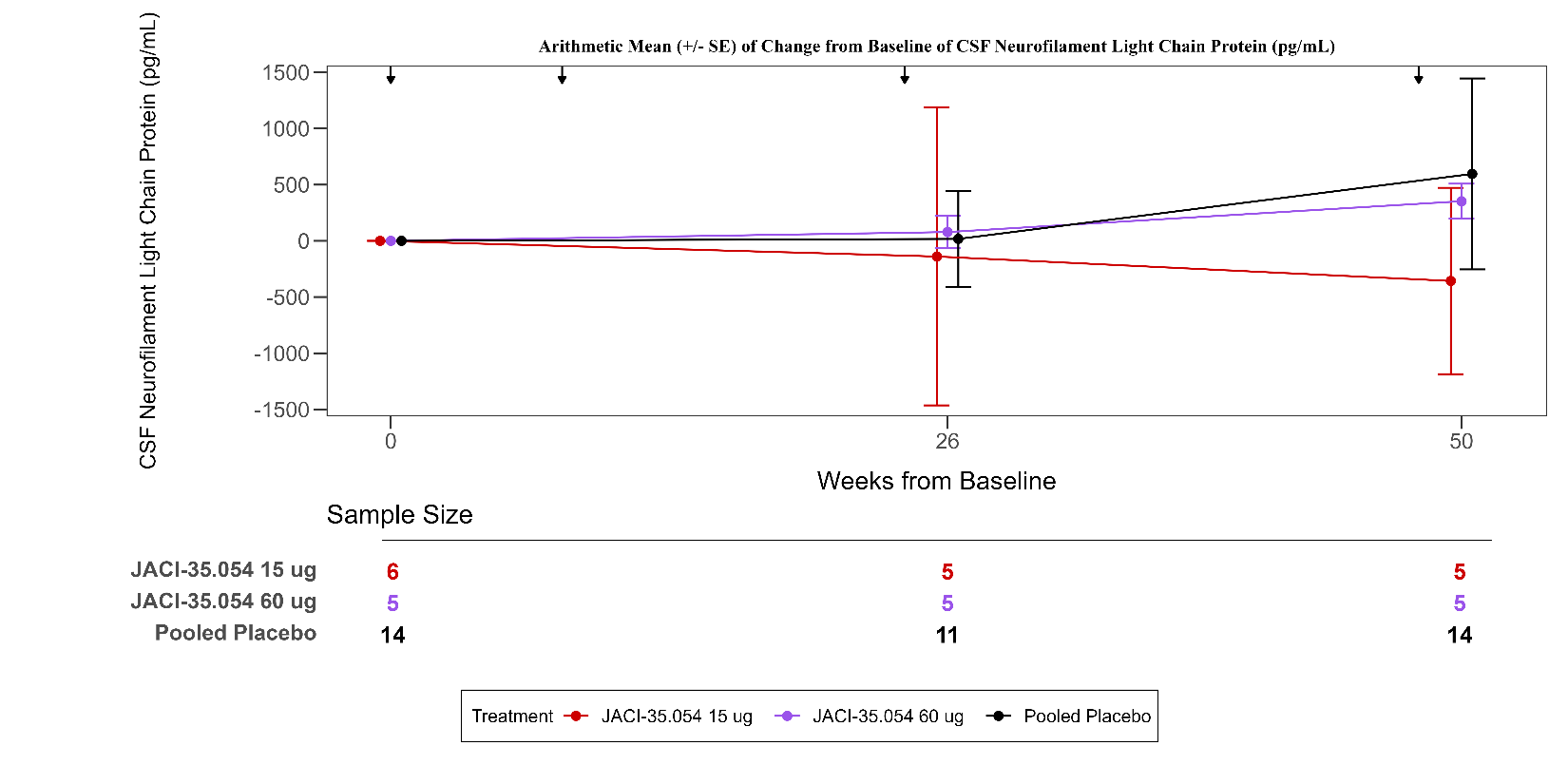
**Figure S32: Arithmetic mean of change from baseline of CSF Neurofilament Light Chain Protein vs nominal visit time by study treatment arm (Cohort 2).** Error bars denote standard error of the mean. Number of subjects by nominal visit and study treatment arm tabulated below. The four vertical arrows at the top of the graph denote nominal study visit times for administration of JACI-35.054 or placebo.


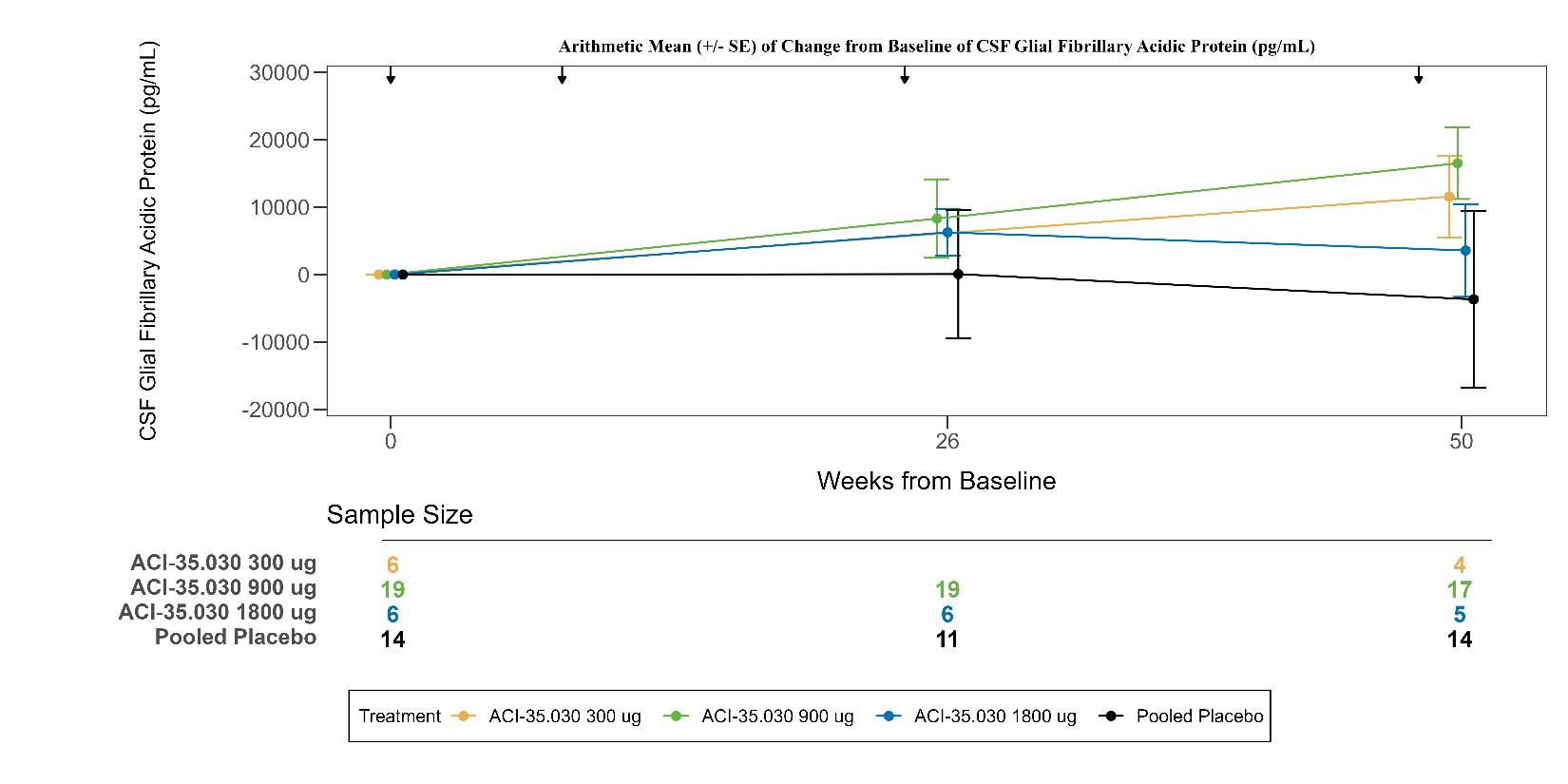
**Figure S33: Arithmetic mean of change from baseline of CSF Glial Fibrillary Acidic Protein vs nominal visit time by study treatment arm (Cohort 1).** Error bars denote standard error of the mean. Number of subjects by nominal visit and study treatment arm tabulated below. The four vertical arrows at the top of the graph denote nominal study visit times for administration of ACI-35.030 or placebo.


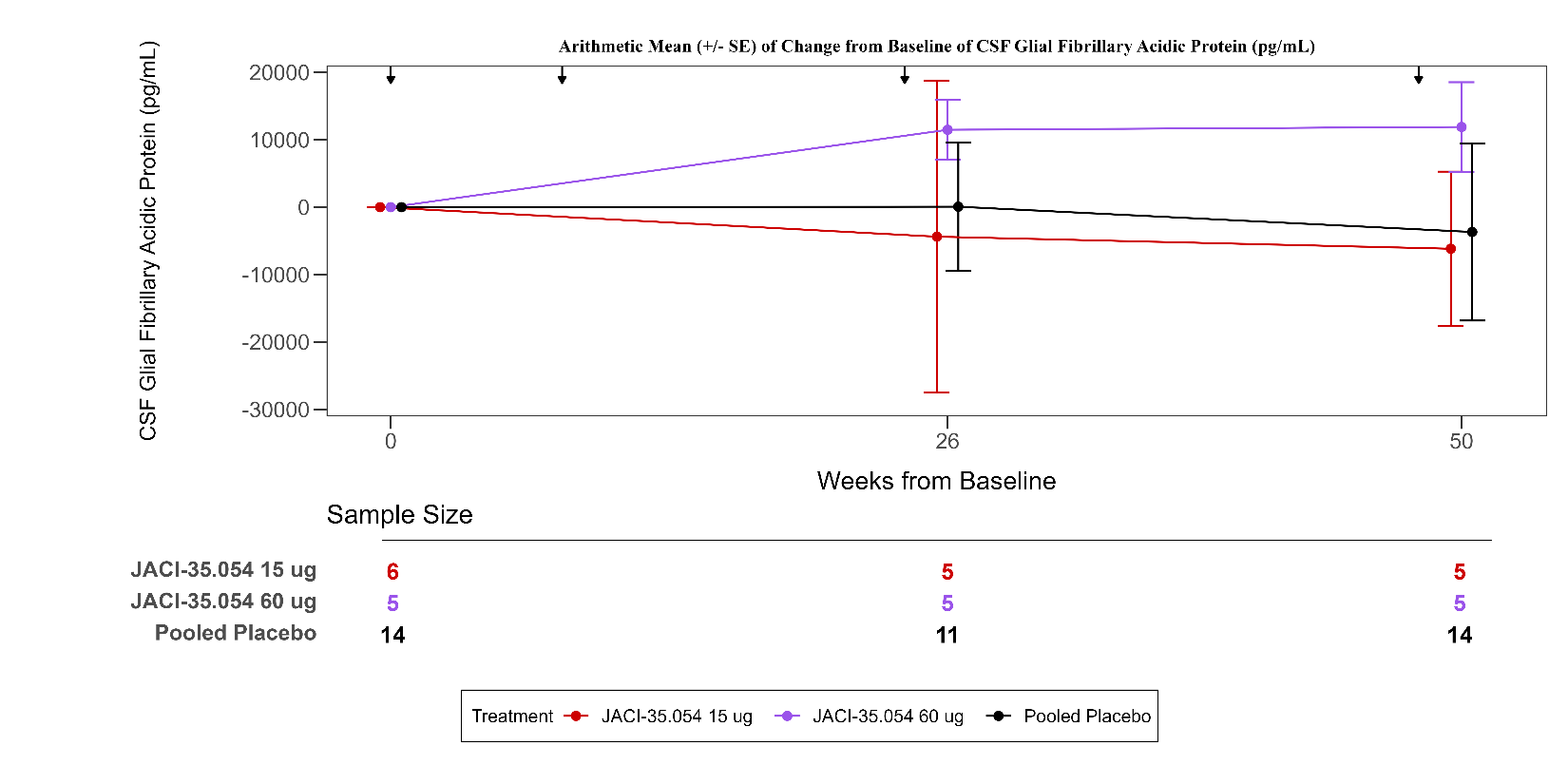
**Figure S34: Arithmetic mean of change from baseline of CSF Glial Fibrillary Acidic Protein vs nominal visit time by study treatment arm (Cohort 2).** Error bars denote standard error of the mean. Number of subjects by nominal visit and study treatment arm tabulated below. The four vertical arrows at the top of the graph denote nominal study visit times for administration of JACI-35.054 or placebo.


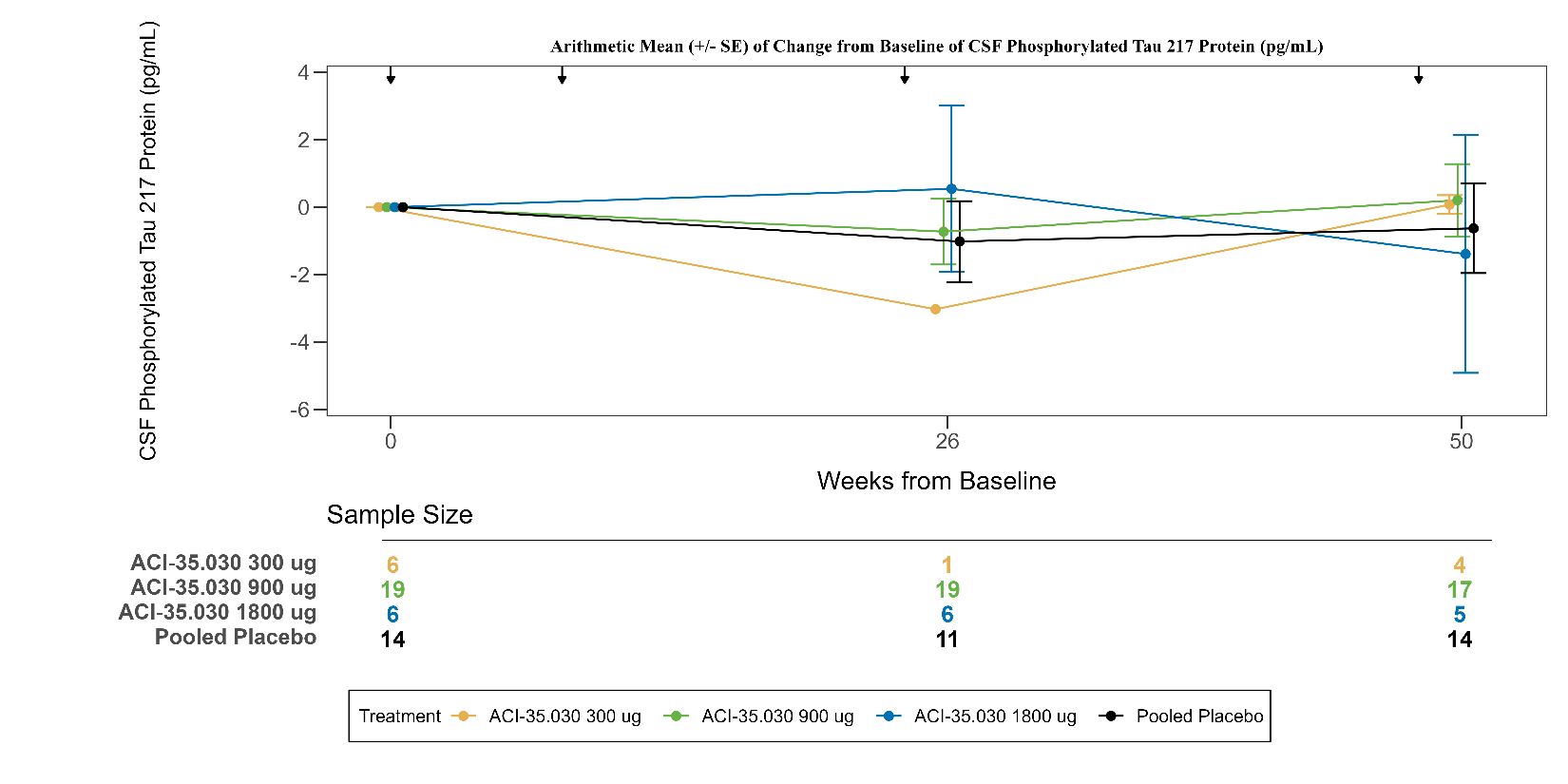
**Figure S35: Arithmetic mean of change from baseline of CSF Phosphorylated Tau 217 Protein vs nominal visit time by study treatment arm (Cohort 1).** Error bars denote standard error of the mean. Number of subjects by nominal visit and study treatment arm tabulated below. The four vertical arrows at the top of the graph denote nominal study visit times for administration of ACI-35.030 or placebo.


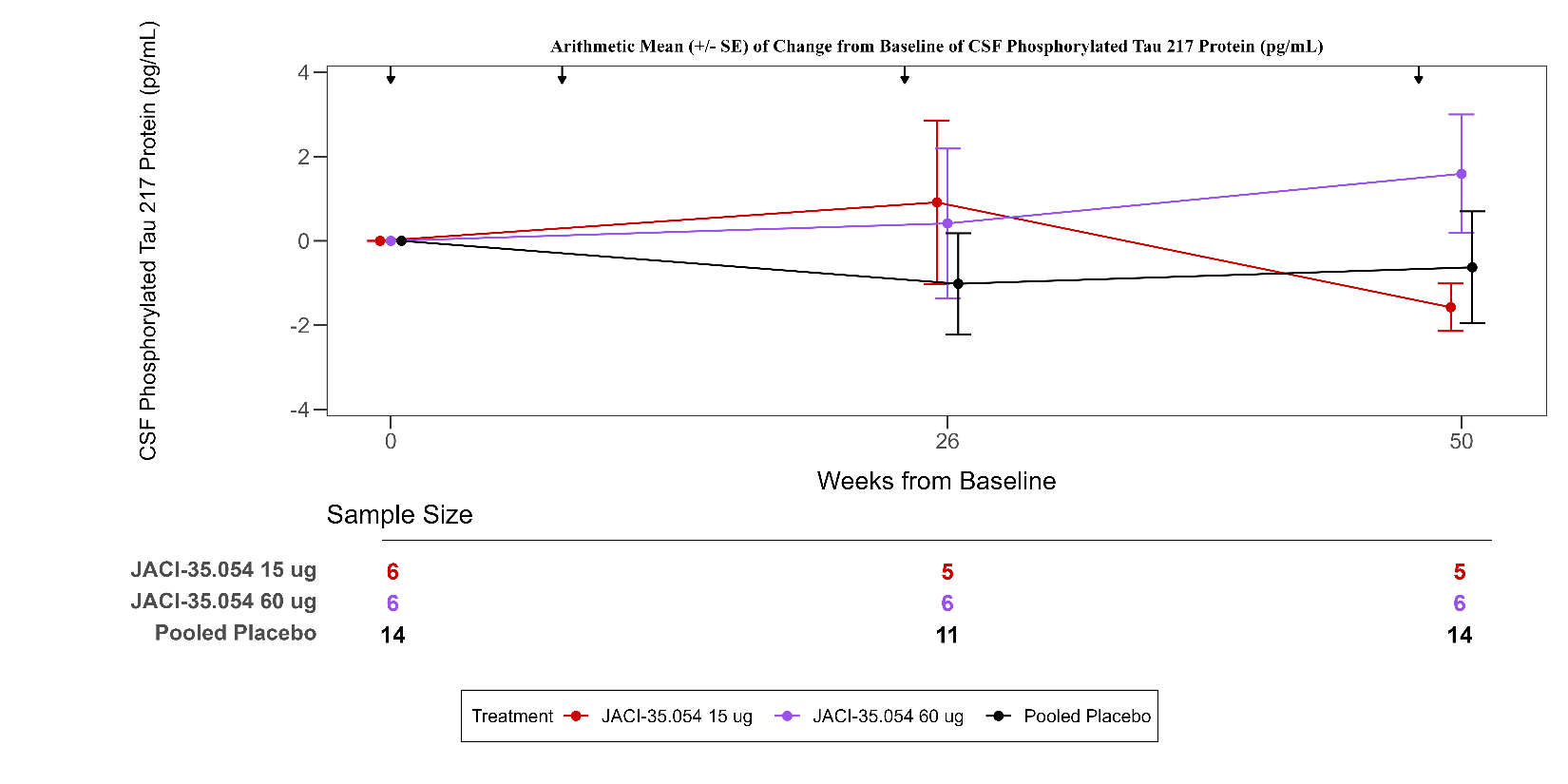
**Figure S36: Arithmetic mean of change from baseline of CSF Phosphorylated Tau 217 Protein vs nominal visit time by study treatment arm (Cohort 2).** Error bars denote standard error of the mean. Number of subjects by nominal visit and study treatment arm tabulated below. The four vertical arrows at the top of the graph denote nominal study visit times for administration of JACI-35.054 or placebo.


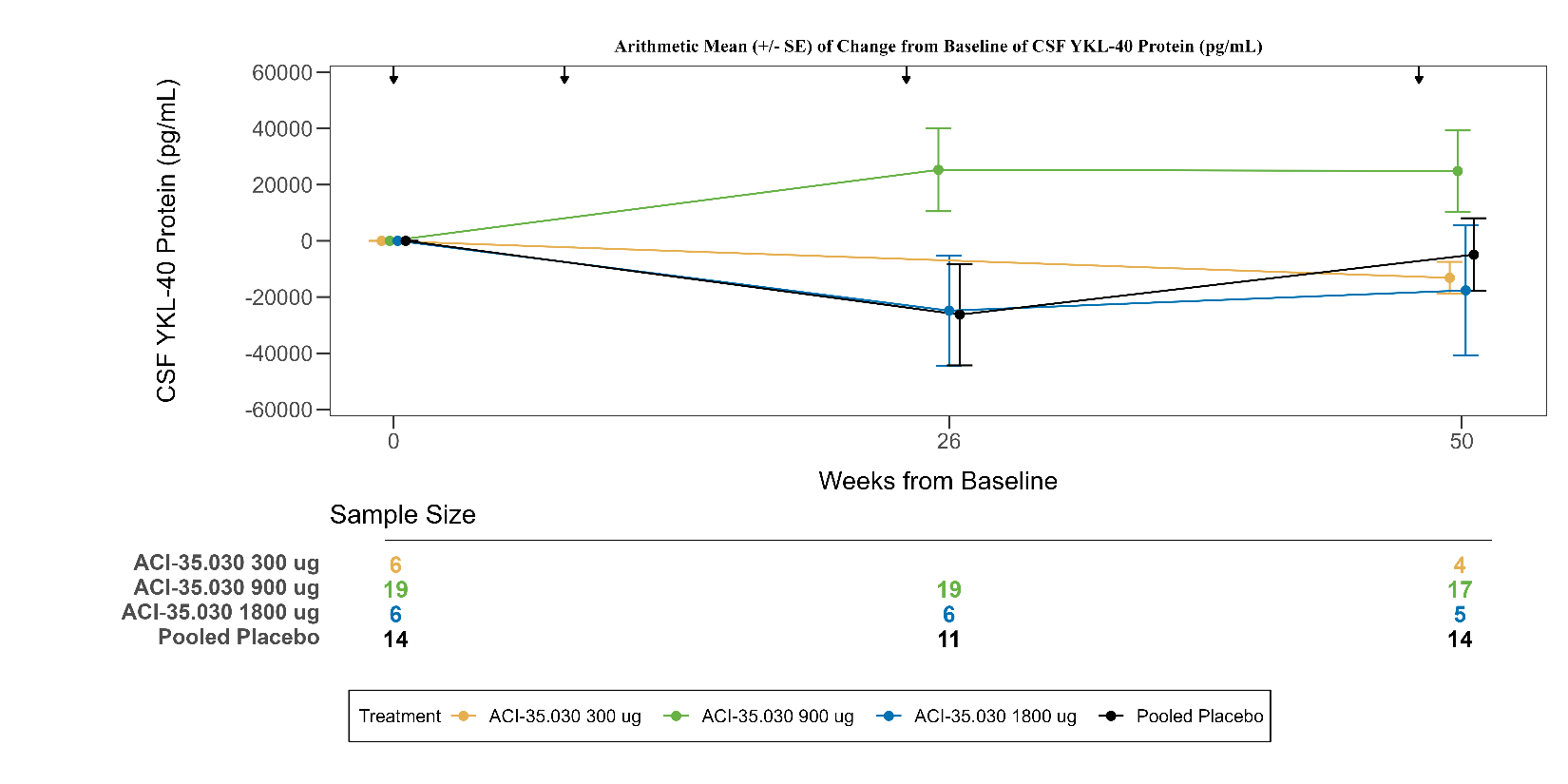
**Figure S37: Arithmetic mean of change from baseline of CSF YKL-40 Protein vs nominal visit time by study treatment arm (Cohort 1).** Error bars denote standard error of the mean. Number of subjects by nominal visit and study treatment arm tabulated below. The four vertical arrows at the top of the graph denote nominal study visit times for administration of ACI-35.030 or placebo.


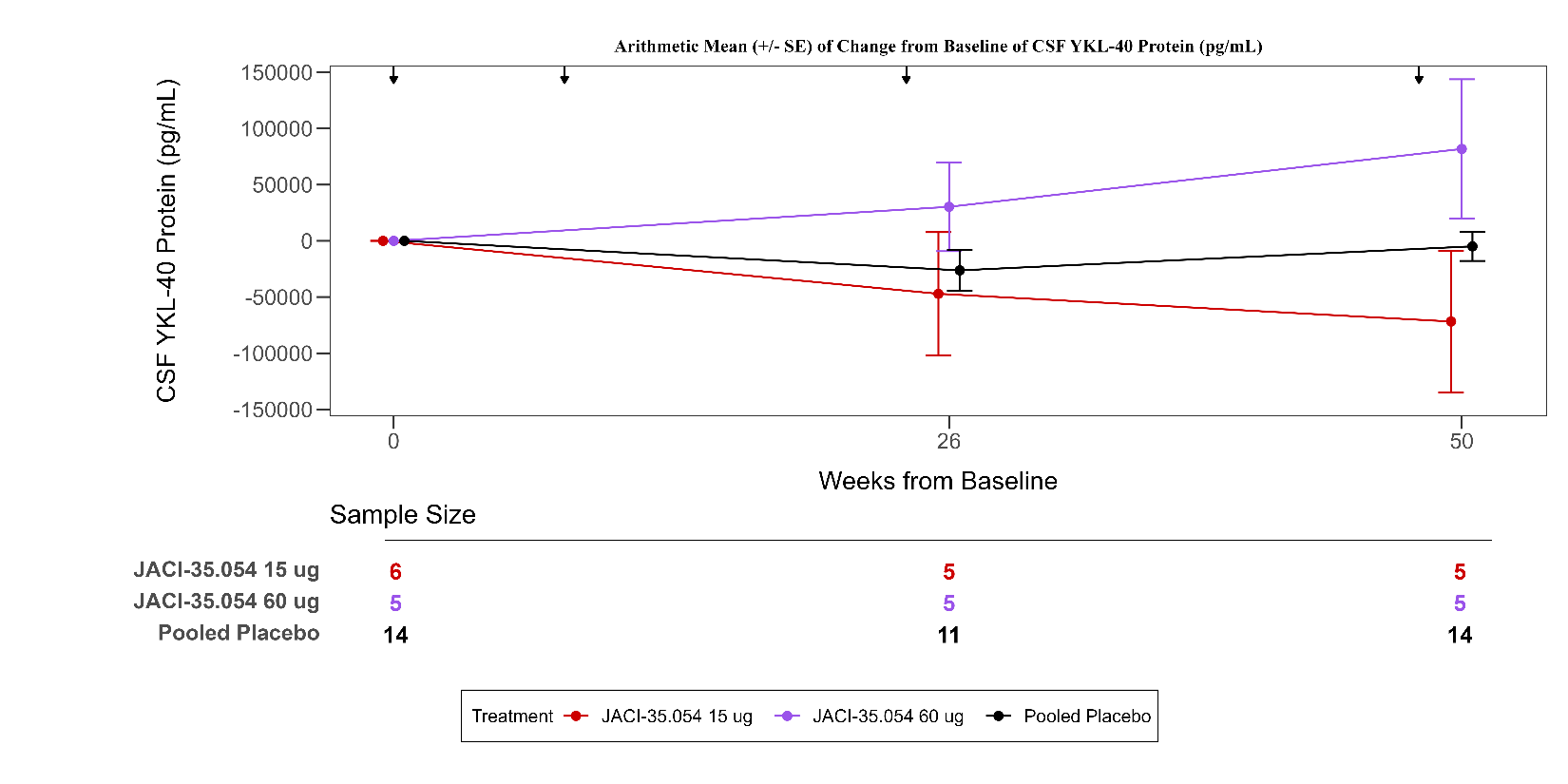
**Figure S38: Arithmetic mean of change from baseline of CSF YKL-40 Protein vs nominal visit time by study treatment arm (Cohort 2).** Error bars denote standard error of the mean. Number of subjects by nominal visit and study treatment arm tabulated below. The four vertical arrows at the top of the graph denote nominal study visit times for administration of JACI-35.054 or placebo.


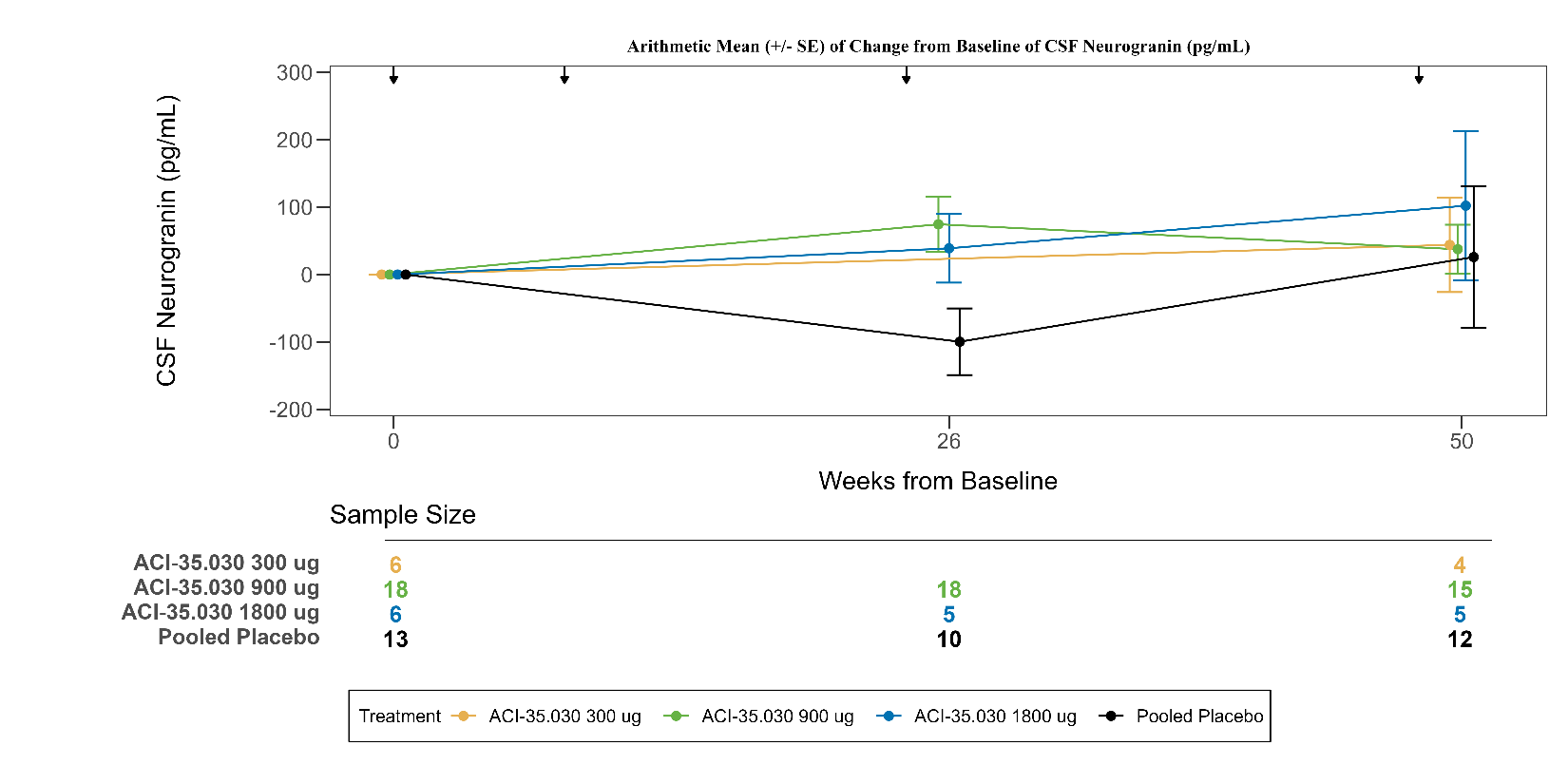
**Figure S39: Arithmetic mean of change from baseline of CSF Neurogranin vs nominal visit time by study treatment arm (Cohort 1).** Error bars denote standard error of the mean. Number of subjects by nominal visit and study treatment arm tabulated below. The four vertical arrows at the top of the graph denote nominal study visit times for administration of ACI-35.030 or placebo.


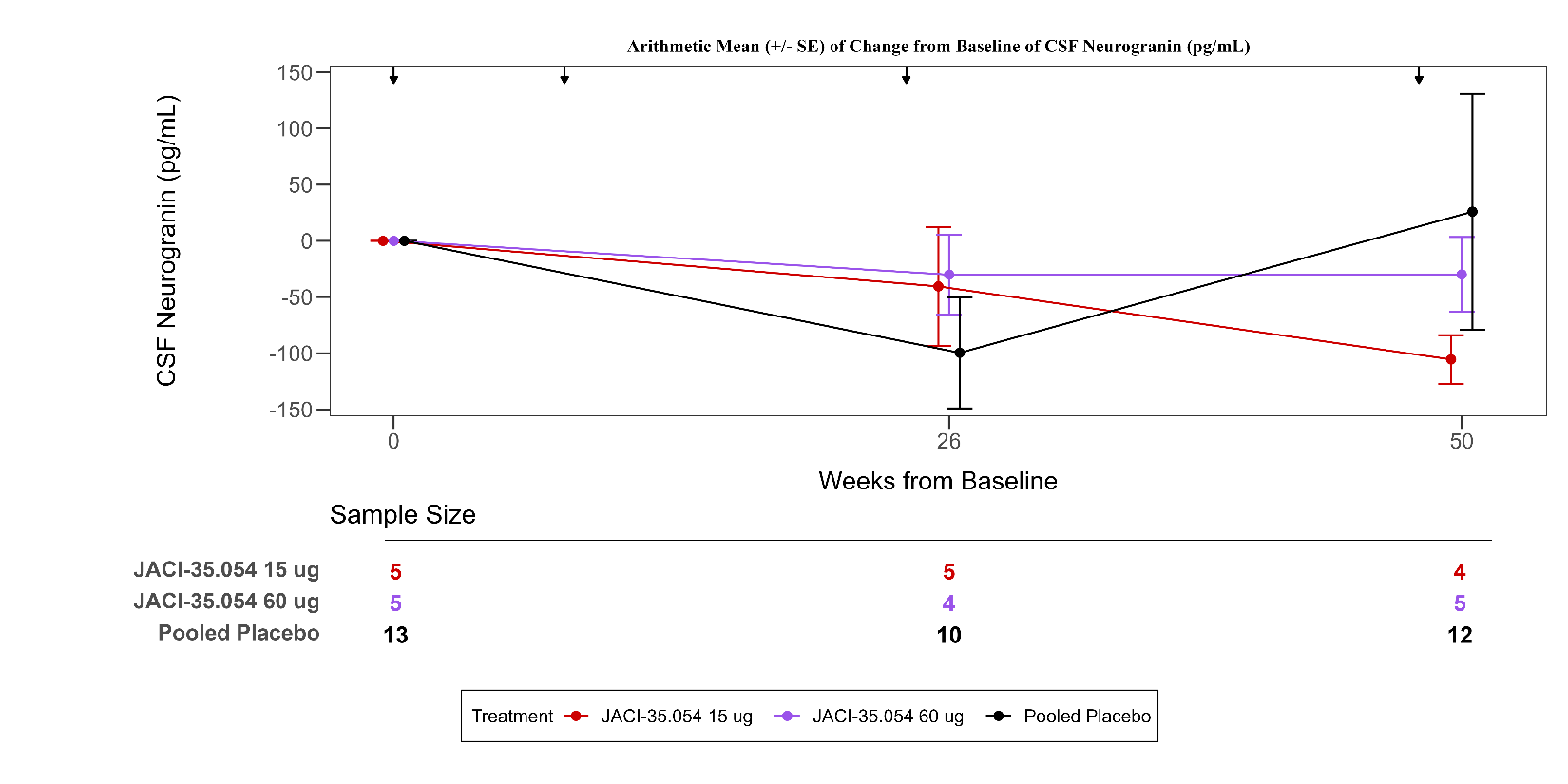
**Figure S40: Arithmetic mean of change from baseline of CSF Neurogranin vs nominal visit time by study treatment arm (Cohort 2).** Error bars denote standard error of the mean. Number of subjects by nominal visit and study treatment arm tabulated below. The four vertical arrows at the top of the graph denote nominal study visit times for administration of JACI-35.054 or placebo.


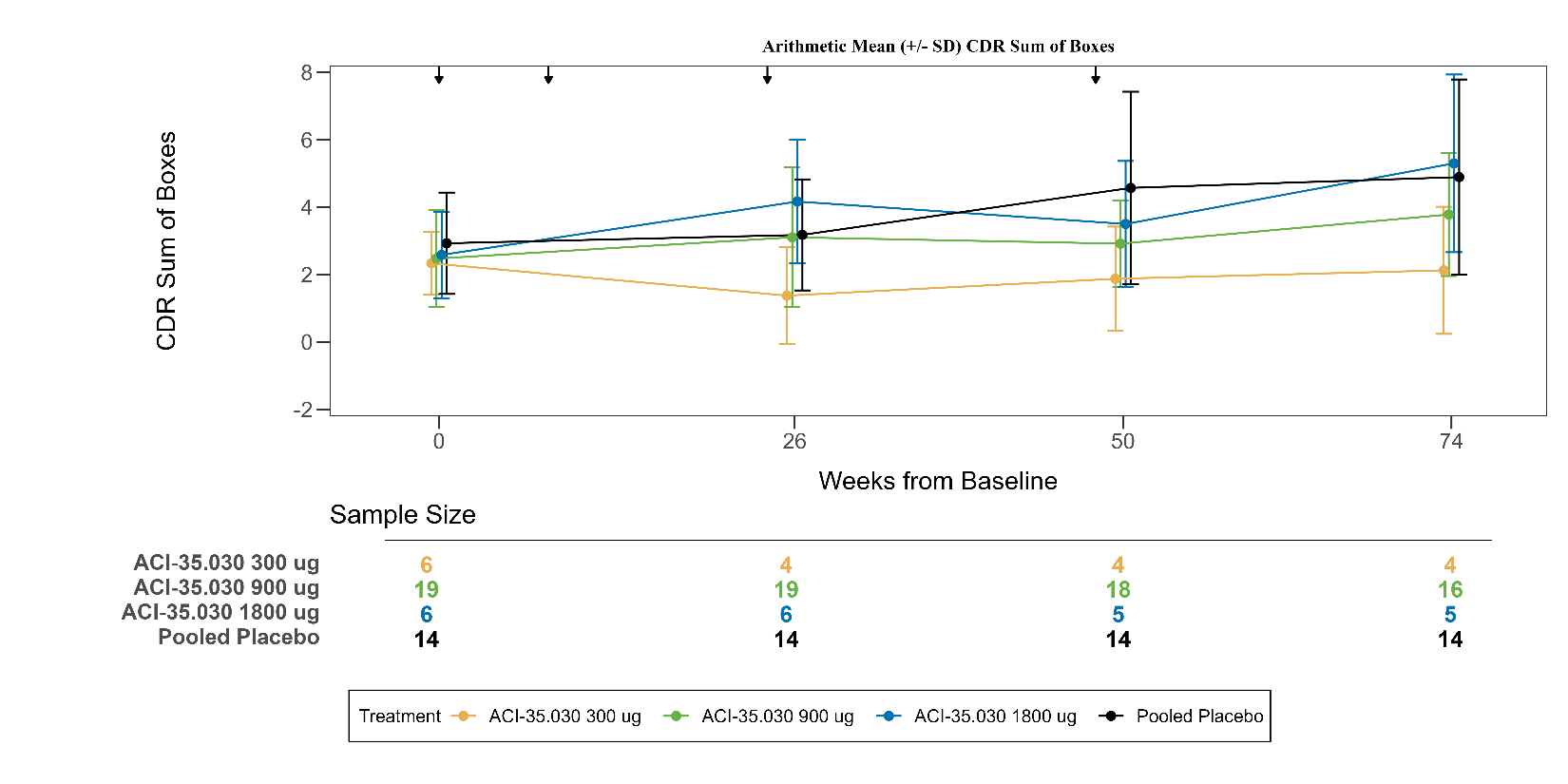
**Figure S41: Arithmetic mean of CDR Sum of Boxes vs nominal visit time by study treatment arm (Cohort 1).** Error bars denote standard deviation. Number of subjects by nominal visit and study treatment arm tabulated below. The four vertical arrows at the top of the graph denote nominal study visit times for administration of ACI-35.030 or placebo.


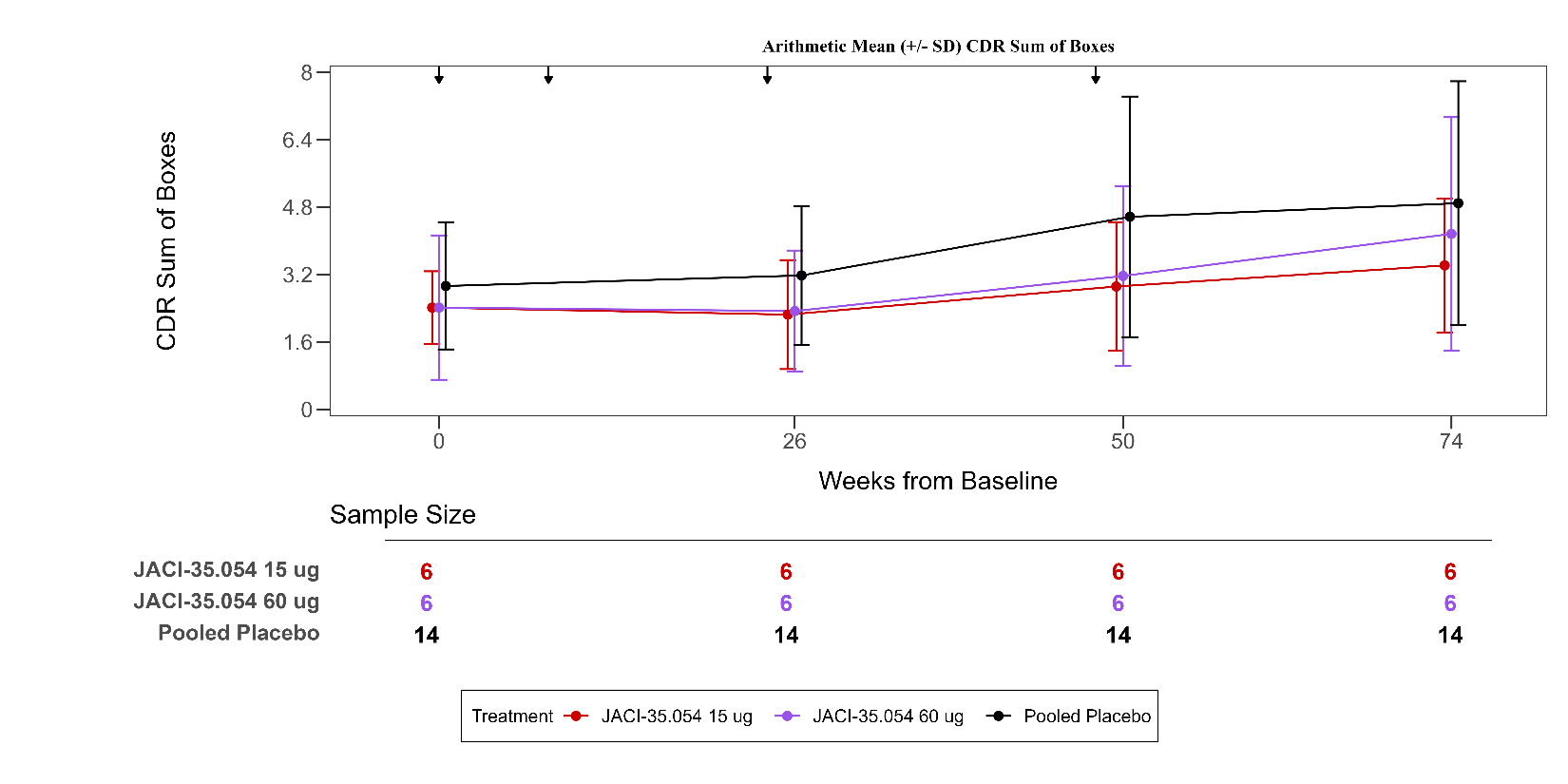
**Figure S42: Arithmetic mean of CDR Sum of Boxes vs nominal visit time by study treatment arm (Cohort 2).** Error bars denote standard deviation. Number of subjects by nominal visit and study treatment arm tabulated below. The four vertical arrows at the top of the graph denote nominal study visit times for administration of JACI-35.054 or placebo.


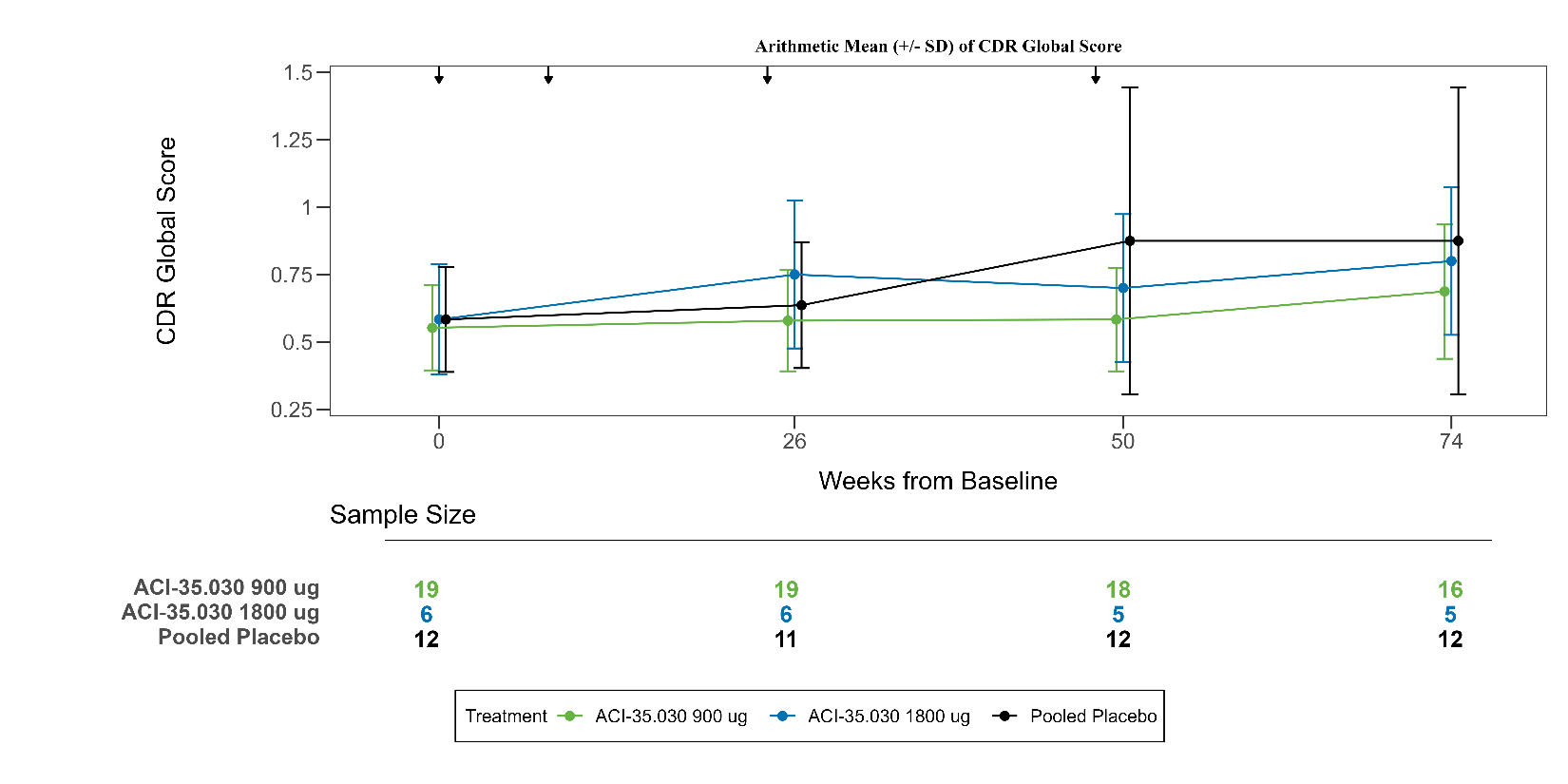
**Figure S43: Arithmetic mean of CDR Global Score vs nominal visit time by study treatment arm (Cohort 1).** Error bars denote standard deviation. Number of subjects by nominal visit and study treatment arm tabulated below. The four vertical arrows at the top of the graph denote nominal study visit times for administration of ACI-35.030 or placebo.


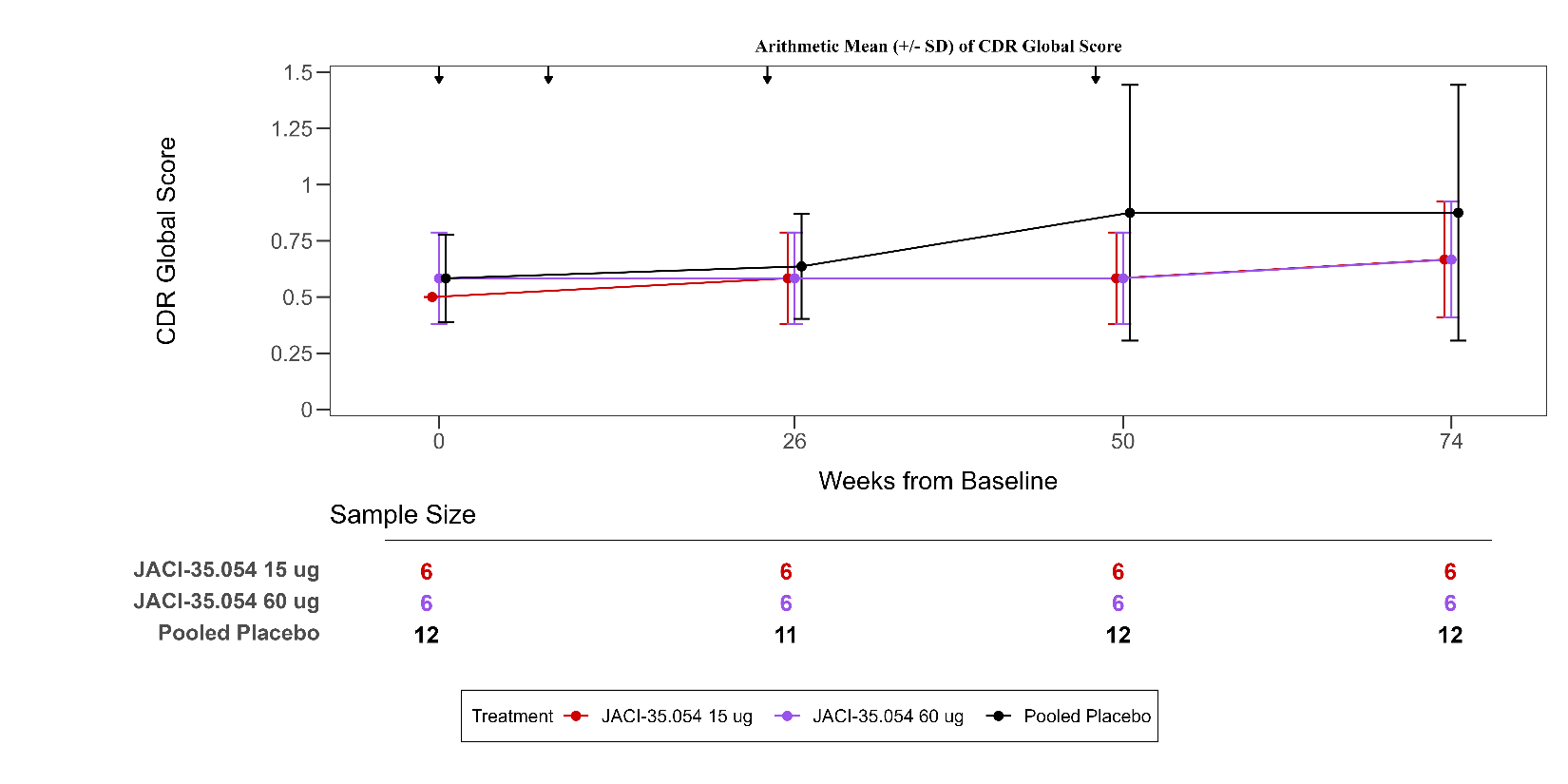
**Figure S44: Arithmetic mean of CDR Global Score vs nominal visit time by study treatment arm (Cohort 2).** Error bars denote standard deviation. Number of subjects by nominal visit and study treatment arm tabulated below. The four vertical arrows at the top of the graph denote nominal study visit times for administration of JACI-35.054 or placebo.


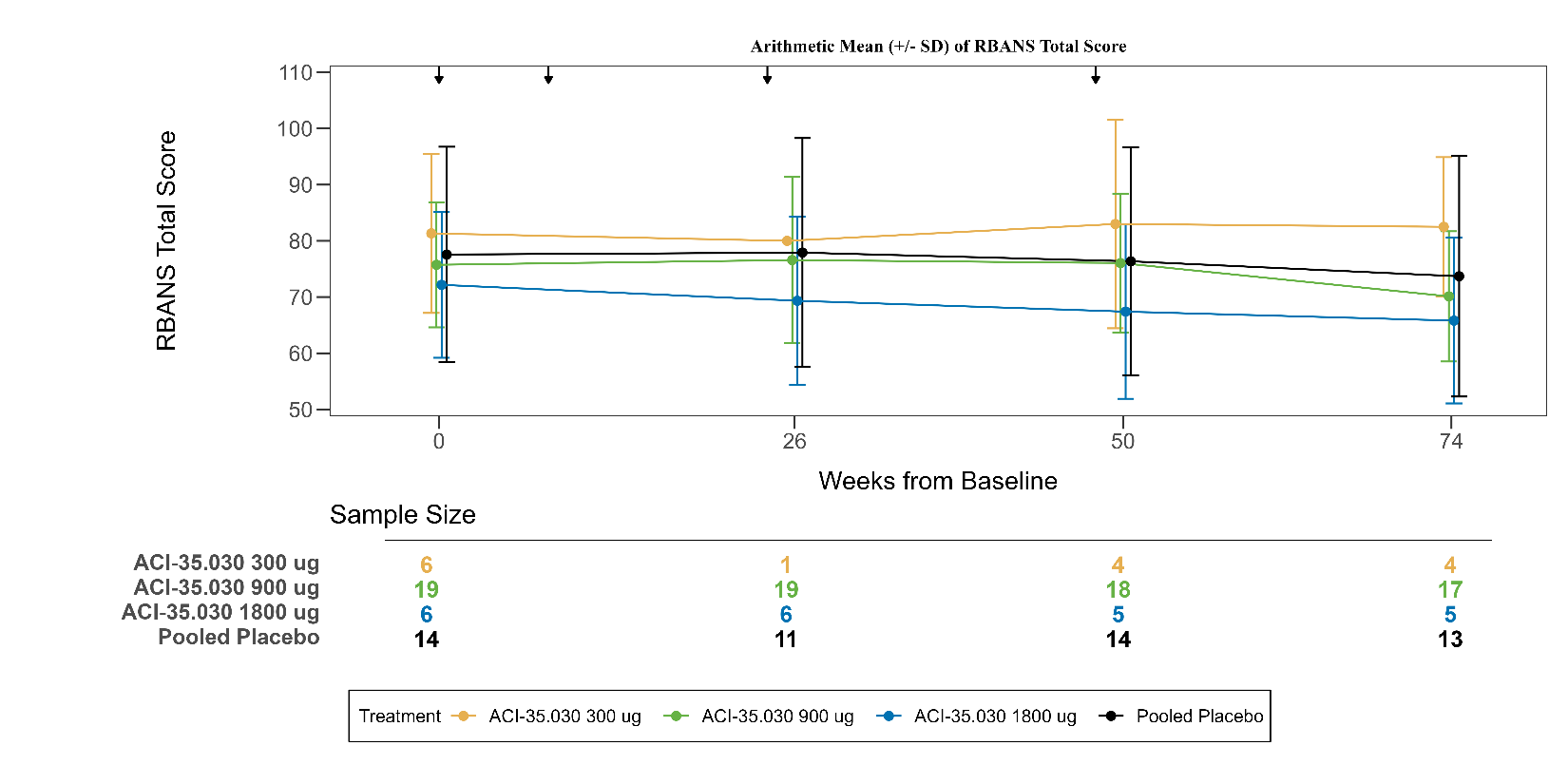
**Figure S45: Arithmetic mean of RBANS Total Score vs nominal visit time by study treatment arm (Cohort 1).** Error bars denote standard deviation. Number of subjects by nominal visit and study treatment arm tabulated below. The four vertical arrows at the top of the graph denote nominal study visit times for administration of ACI-35.030 or placebo.


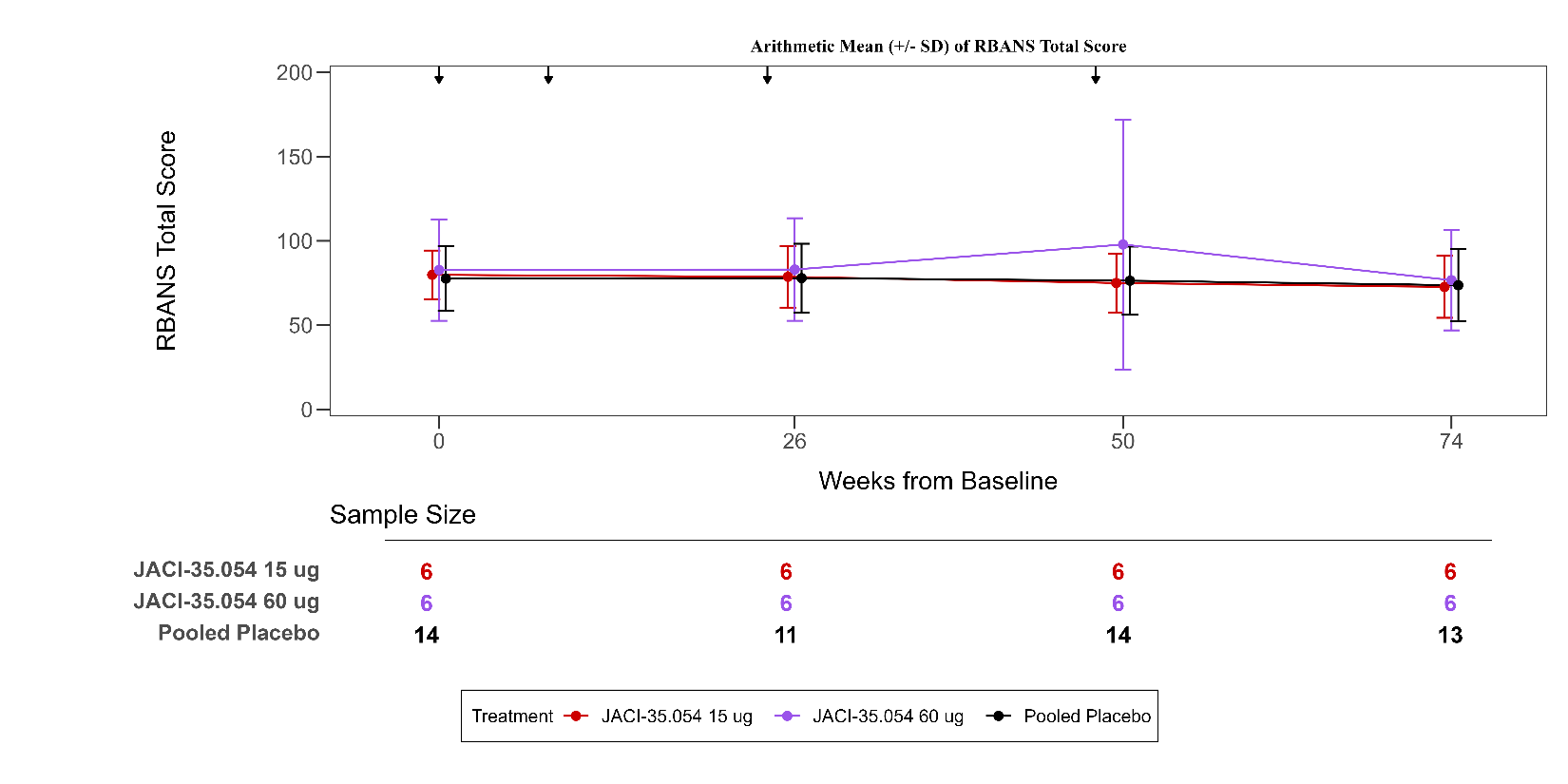
**Figure S46: Arithmetic mean of RBANS Total Score vs nominal visit time by study treatment arm (Cohort 2).** Error bars denote standard deviation. Number of subjects by nominal visit and study treatment arm tabulated below. The four vertical arrows at the top of the graph denote nominal study visit times for administration of JACI-35.054 or placebo.


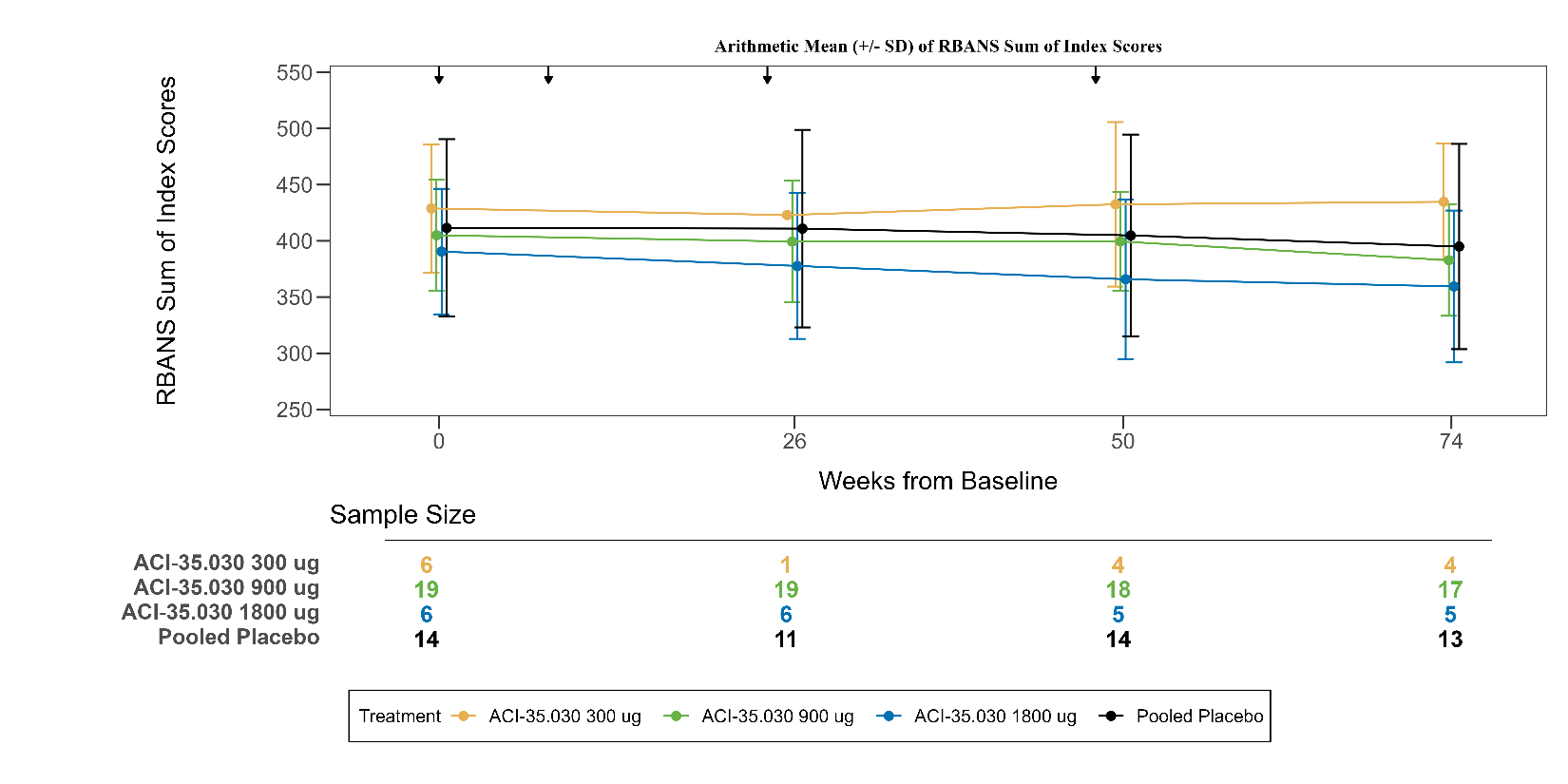
**Figure S47: Arithmetic mean of RBANS Sum of Index Scores vs nominal visit time by study treatment arm (Cohort 1).** Error bars denote standard deviation. Number of subjects by nominal visit and study treatment arm tabulated below. The four vertical arrows at the top of the graph denote nominal study visit times for administration of ACI-35.030 or placebo.


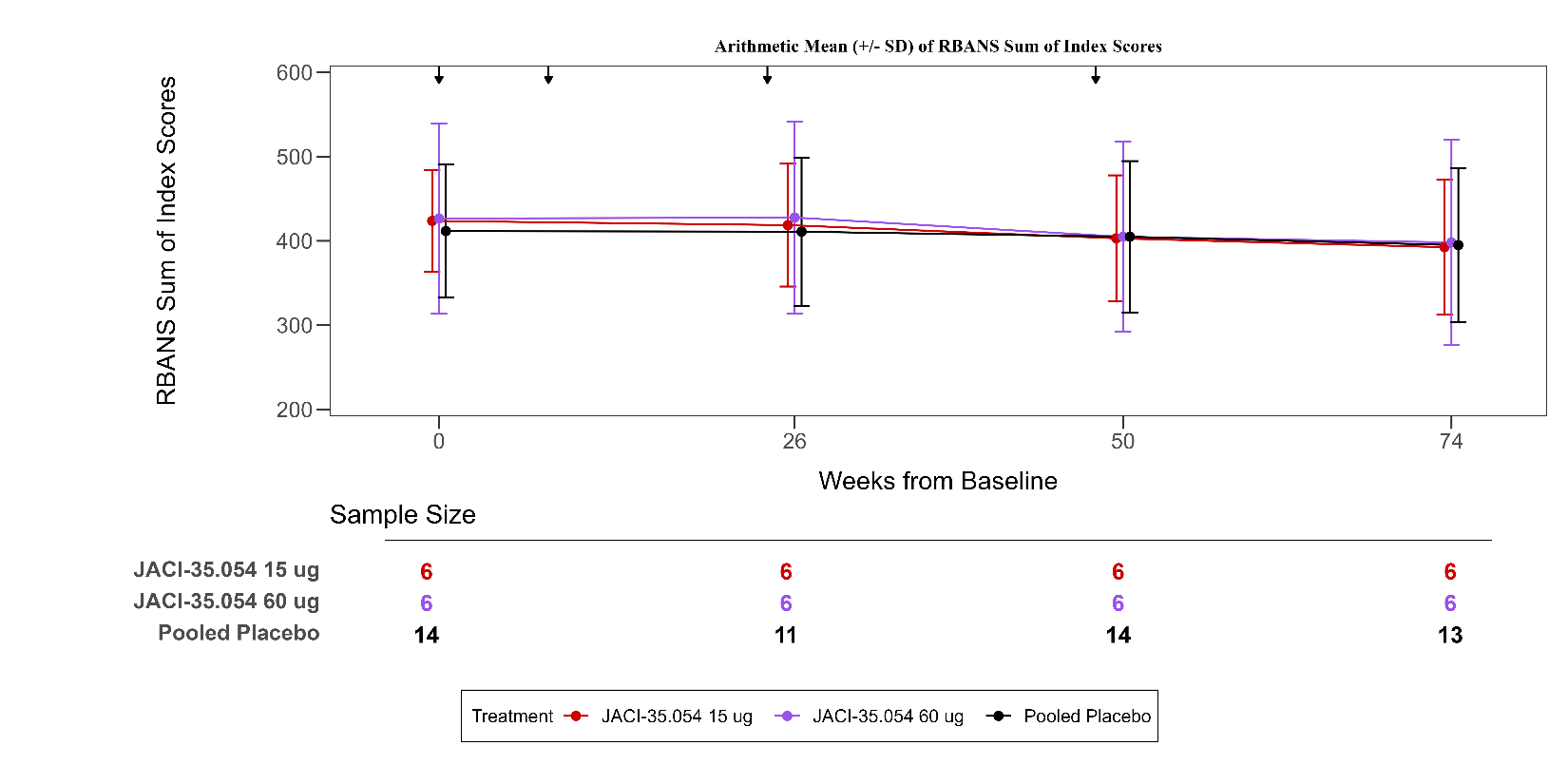
**Figure S48: Arithmetic mean of RBANS Sum of Index Scores vs nominal visit time by study treatment arm (Cohort 2).** Error bars denote standard deviation. Number of subjects by nominal visit and study treatment arm tabulated below. The four vertical arrows at the top of the graph denote nominal study visit times for administration of JACI-35.054 or placebo.


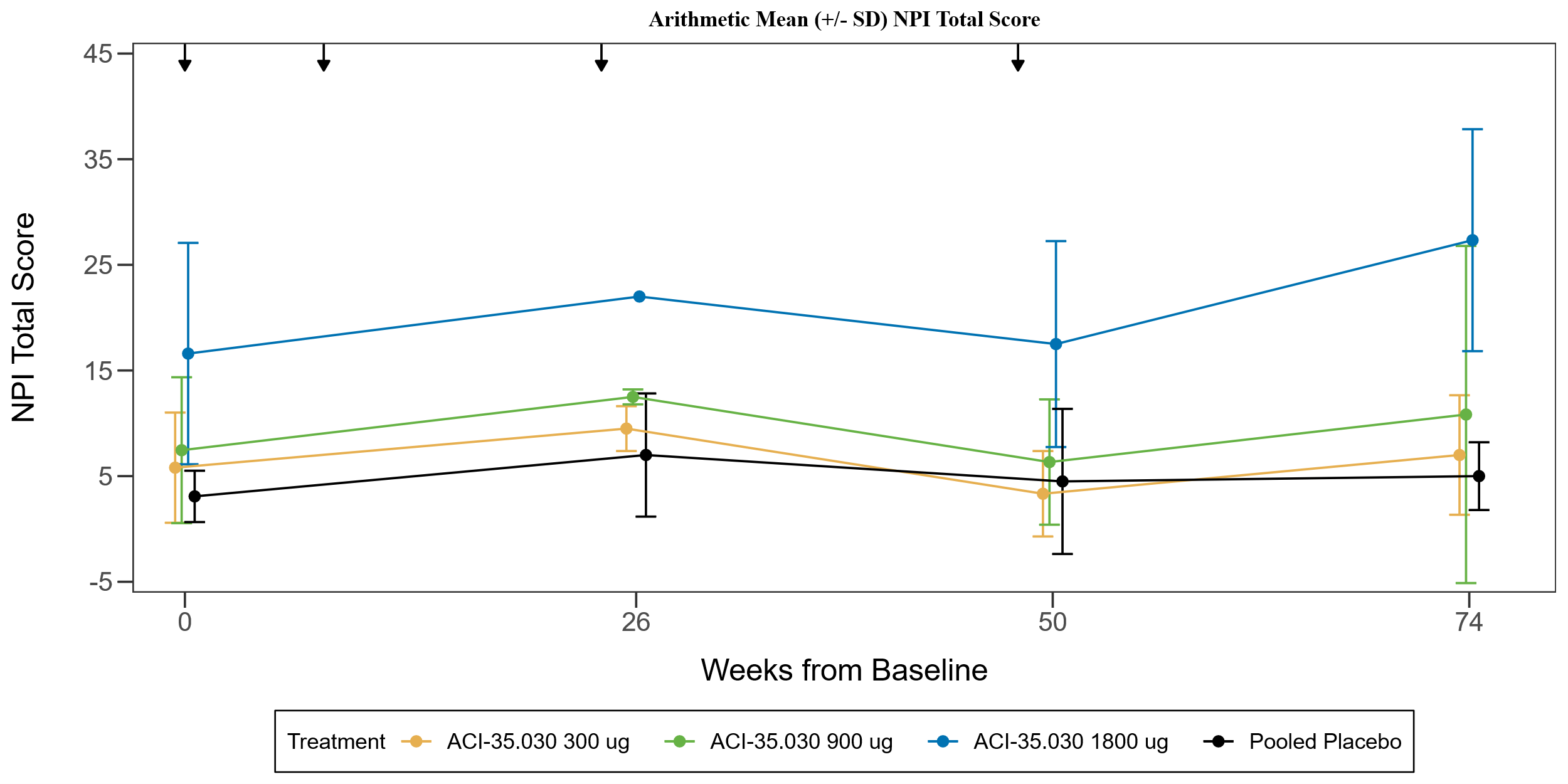


**Figure S49: Arithmetic mean of NPI Total Score vs nominal visit time by study treatment arm (Cohort 1).** Error bars denote standard deviation. Number of subjects by nominal visit and study treatment arm tabulated below. The four vertical arrows at the top of the graph denote nominal study visit times for administration of ACI-35.030 or placebo.


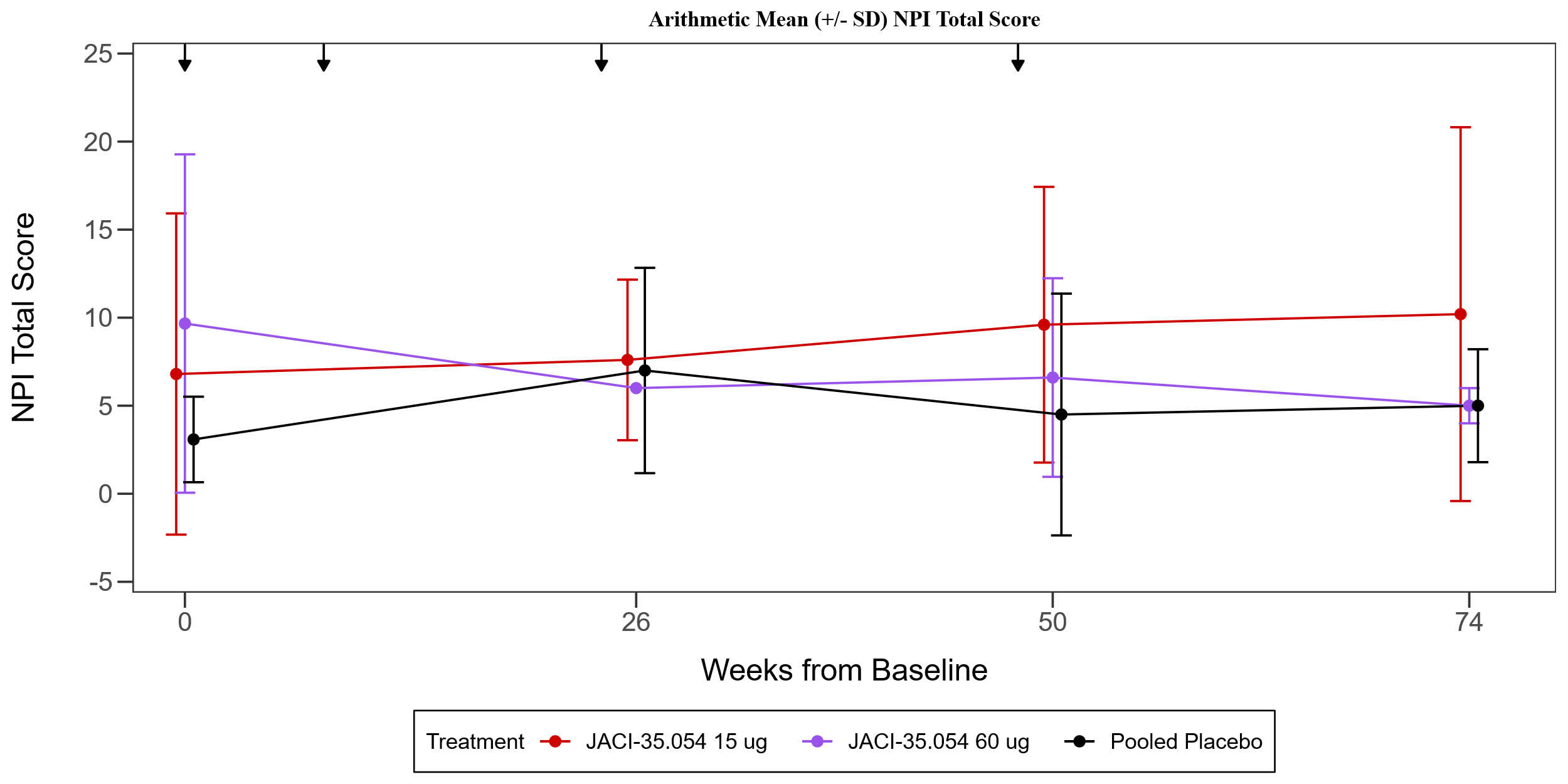


**Figure S50: Arithmetic mean of NPI Total Score vs nominal visit time by study treatment arm (Cohort 2).** Error bars denote standard deviation. Number of subjects by nominal visit and study treatment arm tabulated below. The four vertical arrows at the top of the graph denote nominal study visit times for administration of JACI-35.054 or placebo.


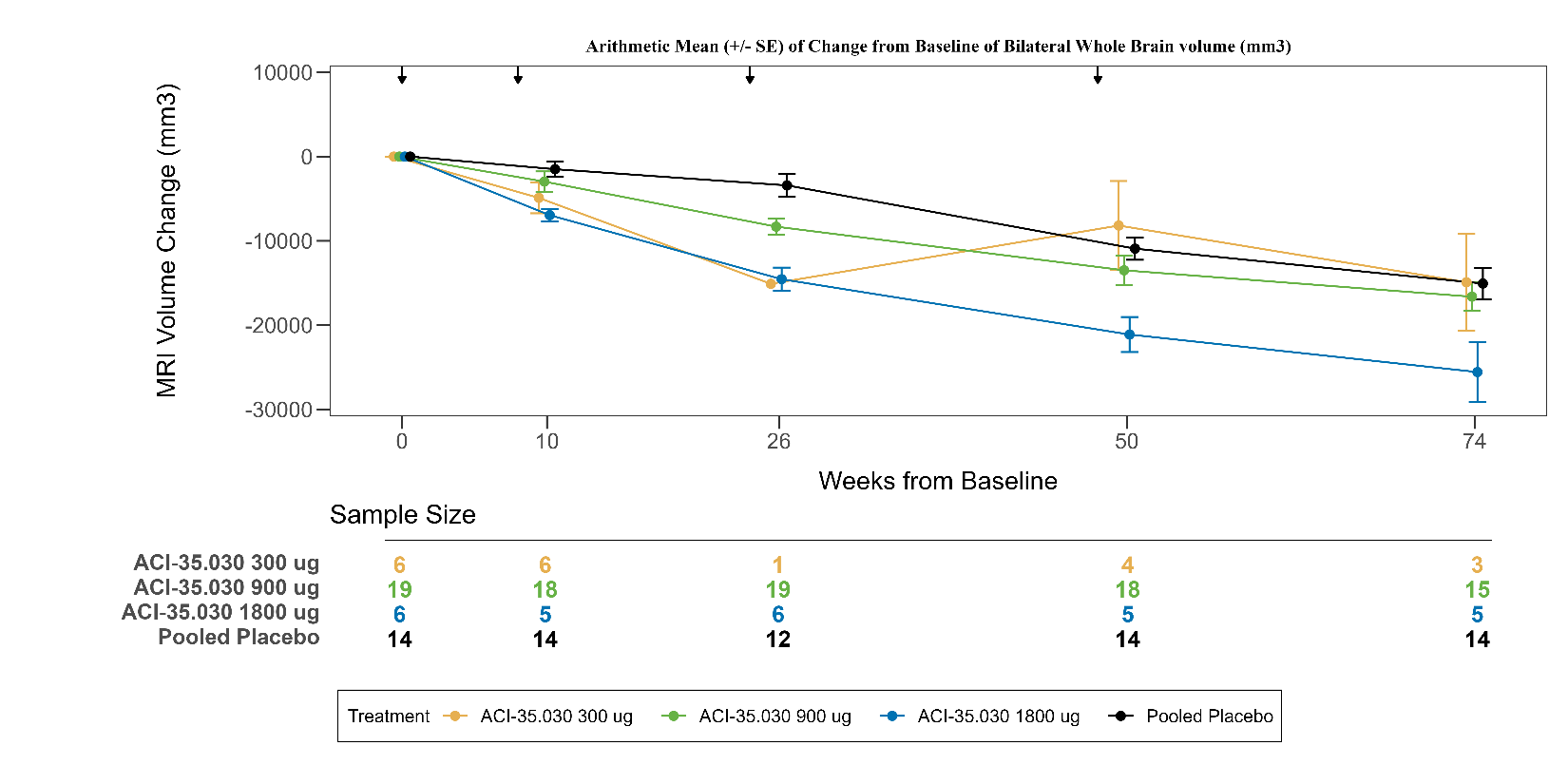
**Figure S51: Arithmetic mean of change from baseline of Whole Brain Volume vs nominal visit time by study treatment arm (Cohort 1).** Error bars denote standard error of the mean. Number of subjects by nominal visit and study treatment arm tabulated below. The four vertical arrows at the top of the graph denote nominal study visit times for administration of ACI-35.030 or placebo.


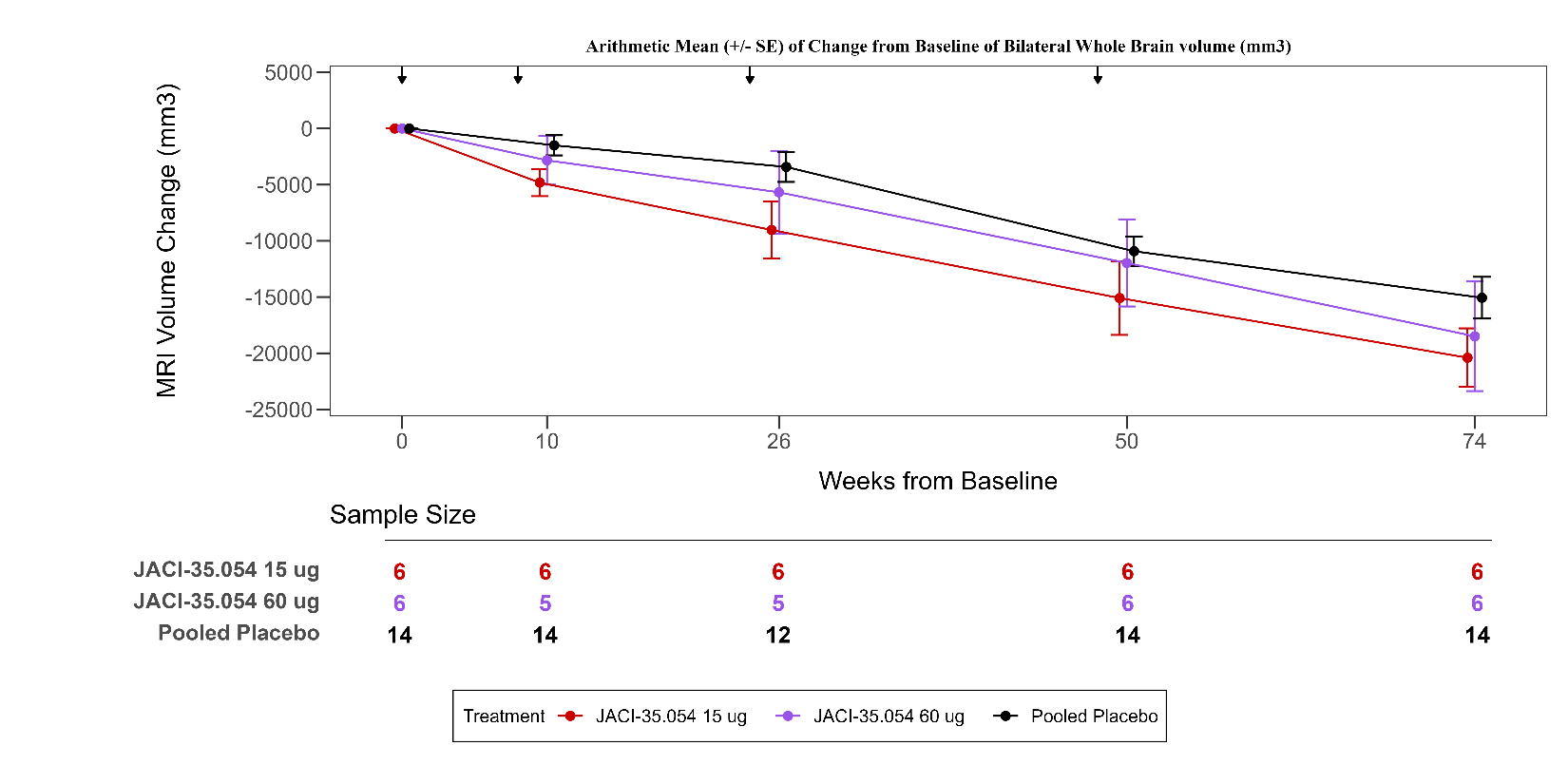
**Figure S52: Arithmetic mean of change from baseline of Whole Brain Volume vs nominal visit time by study treatment arm (Cohort 2).** Error bars denote standard error of the mean. Number of subjects by nominal visit and study treatment arm tabulated below. The four vertical arrows at the top of the graph denote nominal study visit times for administration of JACI-35.054 or placebo.


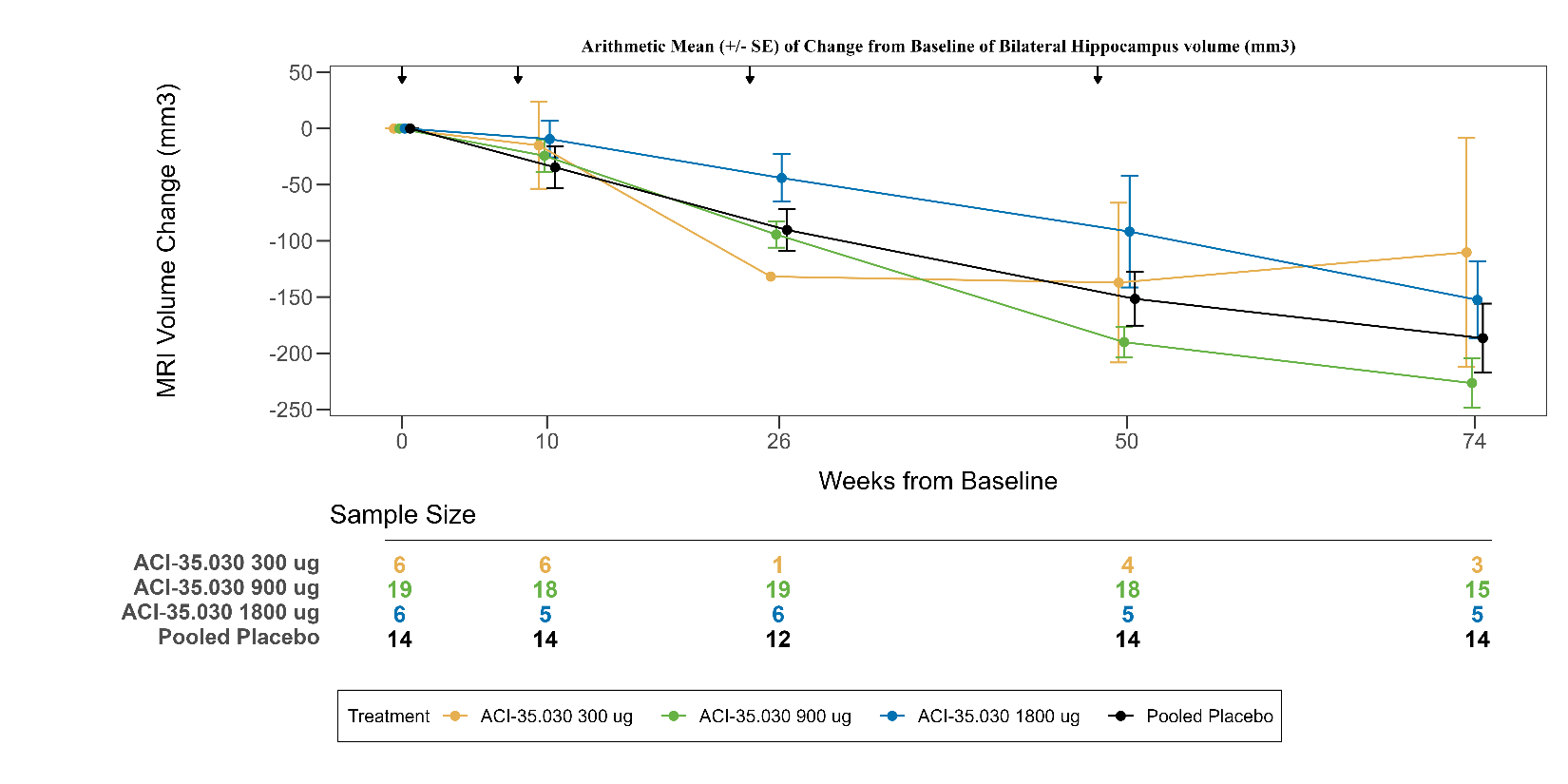
**Figure S53: Arithmetic mean of change from baseline of Bilateral Hippocampus Volume vs nominal visit time by study treatment arm (Cohort 1).** Error bars denote standard error of the mean. Number of subjects by nominal visit and study treatment arm tabulated below. The four vertical arrows at the top of the graph denote nominal study visit times for administration of ACI-35.030 or placebo.


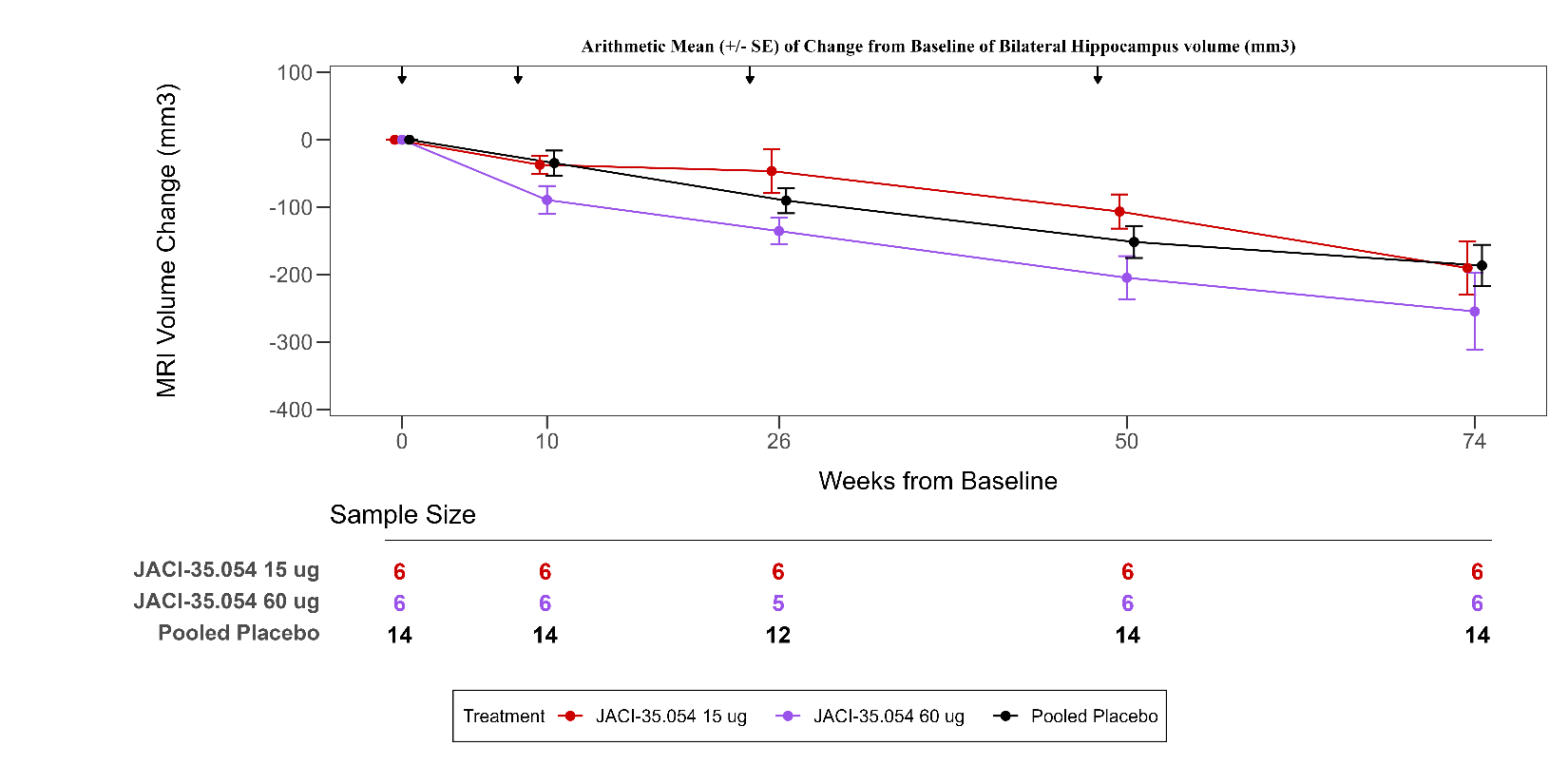
**Figure S54: Arithmetic mean of change from baseline of Bilateral Hippocampus Volume vs nominal visit time by study treatment arm (Cohort 2).** Error bars denote standard error of the mean. Number of subjects by nominal visit and study treatment arm tabulated below. The four vertical arrows at the top of the graph denote nominal study visit times for administration of JACI-35.054 or placebo.


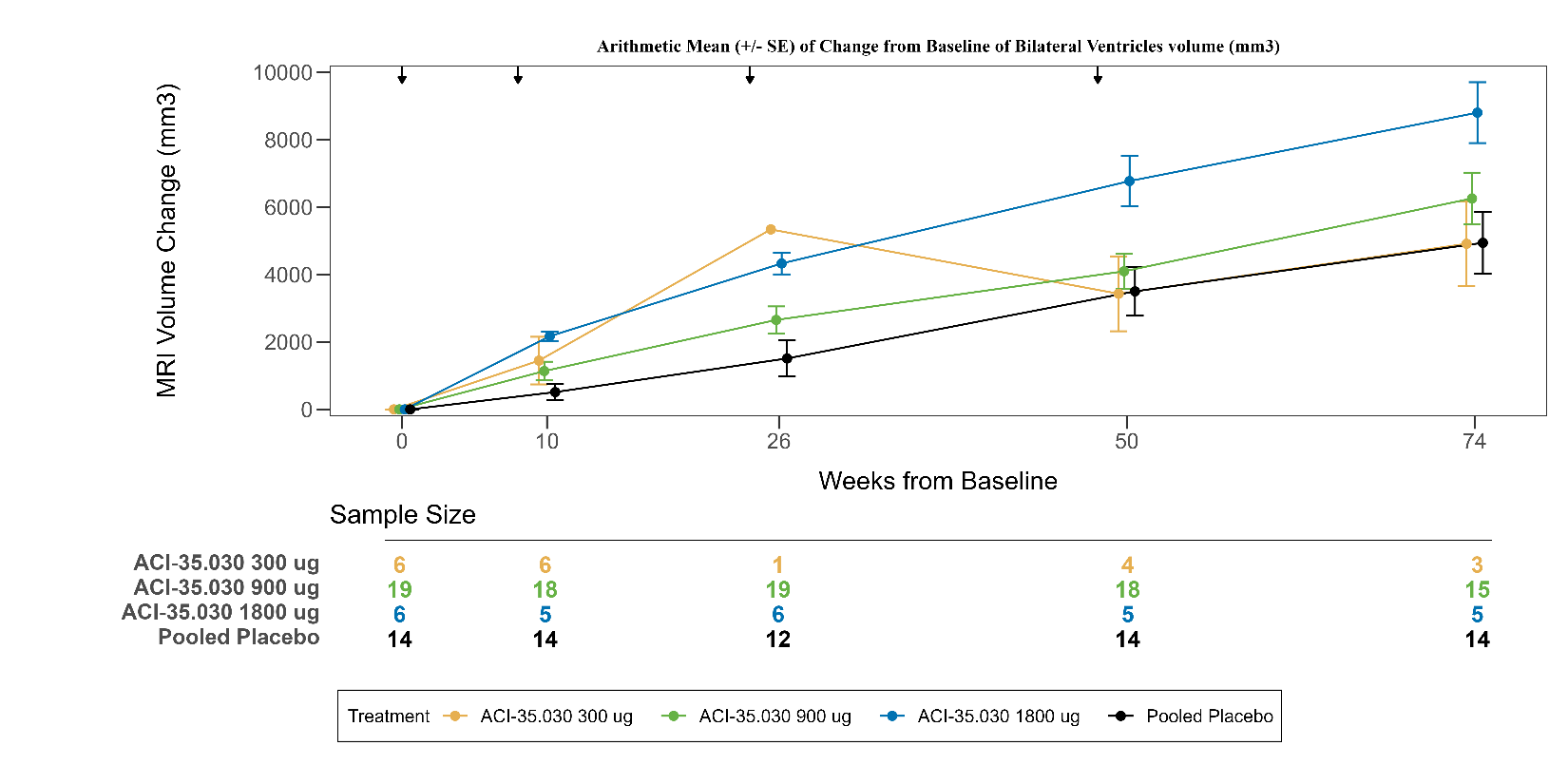
**Figure S55: Arithmetic mean of change from baseline of Bilateral Ventricles Volume vs nominal visit time by study treatment arm (Cohort 1).** Error bars denote standard error of the mean. Number of subjects by nominal visit and study treatment arm tabulated below. The four vertical arrows at the top of the graph denote nominal study visit times for administration of ACI-35.030 or placebo.


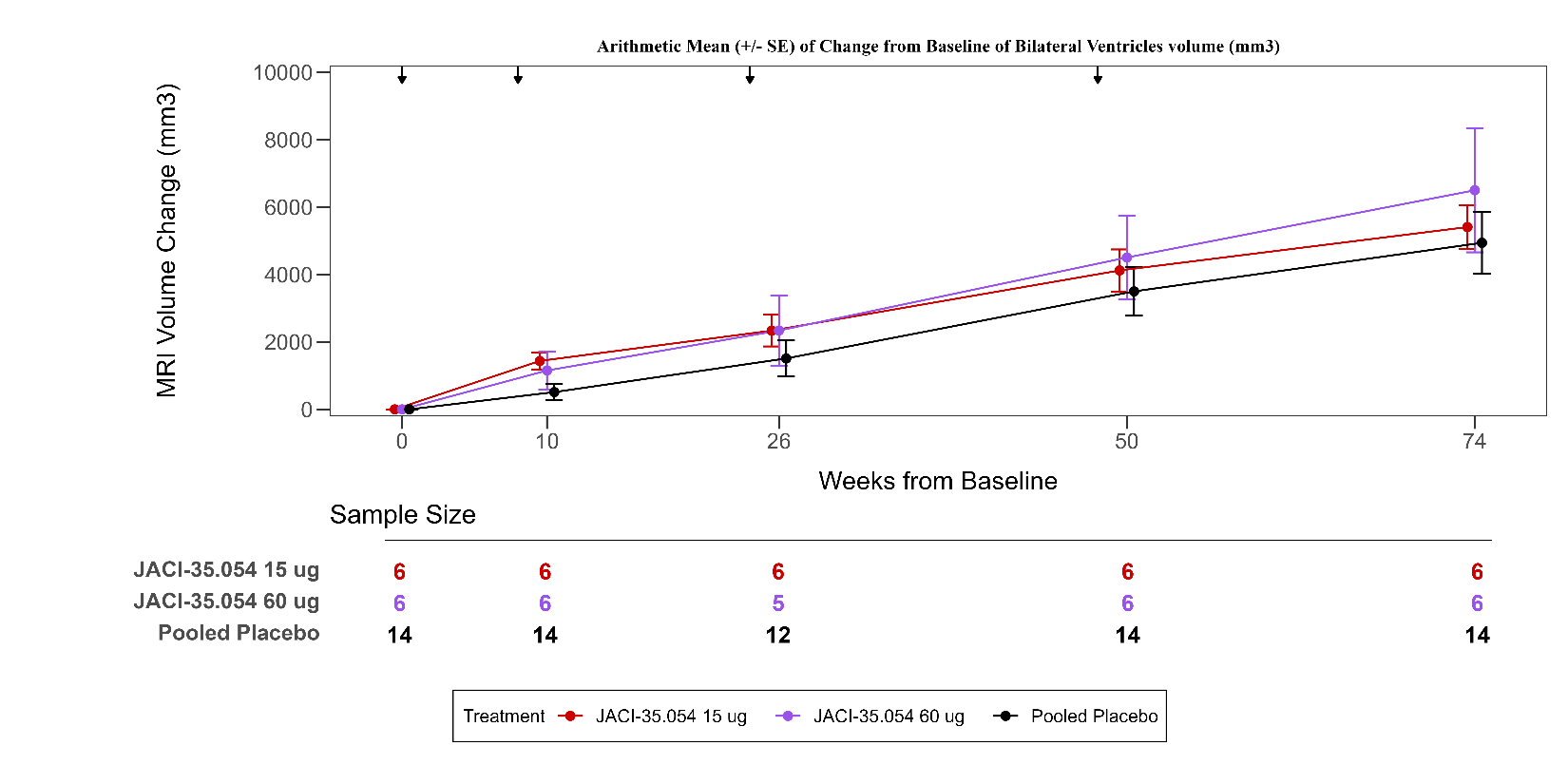
**Figure S56: Arithmetic mean of change from baseline of Bilateral Ventricles Volume vs nominal visit time by study treatment arm (Cohort 2).** Error bars denote standard error of the mean. Number of subjects by nominal visit and study treatment arm tabulated below. The four vertical arrows at the top of the graph denote nominal study visit times for administration of JACI-35.054 or placebo.

**Supplementary Tables**

|  | | | | | | | |
| --- | --- | --- | --- | --- | --- | --- | --- |
| **Visit  Response** | **ACI-35.030 300 µg (N=6)** | **ACI-35.030 900 µg (N=19)** | **ACI-35.030 1800 µg (N=6)** | **JACI-35.054 15 µg (N=6)** | **JACI-35.054 60 µg (N=6)** | **Pooled Placebo (N=14)** |  |
| Visit 2 (Week 2) |  |  |  |  |  |  |  |
| n | 6 | 19 | 6 | 6 | 6 | 14 |  |
| Mean (std) | 59.8 (69.30) | 427.6 (764.92) | 368.4 (252.59) | 1.7 (0.74) | 39.7 (89.39) | 1.0 (0.23) |  |
| Visit 4 (Week 10) |  |  |  |  |  |  |  |
| N | 6 | 19 | 6 | 6 | 6 | 14 |  |
| Mean (std) | 143.7 (199.70) | 347.5 (468.08) | 295.4 (230.33) | 226.2 (173.65) | 111.0 (83.77) | 1.0 (0.15) |  |
| Visit 6 (Week 26) |  |  |  |  |  |  |  |
| N | 1 | 18 | 6 | 6 | 6 | 12 |  |
| Mean (std) | 12.9 (-) | 235.7 (563.34) | 149.9 (131.59) | 553.6 (556.89) | 130.5 (65.11) | 1.3 (0.37) |  |
| Visit 9 (Week 50) |  |  |  |  |  |  |  |
| N | 4 | 18 | 5 | 6 | 6 | 14 |  |
| Mean (std) | 86.2 (133.44) | 369.9 (1250.07) | 107.4 (63.97) | 518.1 (304.34) | 233.4 (229.84) | 1.0 (0.41) |  |
| Visit 11  (FU, Week 74) |  |  |  |  |  |  |  |
| N | 4 | 17 | 5 | 6 | 6 | 14 |  |
| Mean (std) | 5.0 (3.64) | 97.2 (330.06) | 19.4 (18.04) | 146.4 (93.22) | 53.1 (48.57) | 0.9 (0.33) |  |
| **Table S1: Anti-pTau IgG (AU/mL) Titres in Serum – Fold-change from Baseline – Cohort 1 and 2 (Intention-to-Treat Population)**. FU=Follow-up; IgG=immunoglobulin G; pTau=phospho-Tau; std=standard deviation. | | | | | | |  |

|  | | | | | | | |
| --- | --- | --- | --- | --- | --- | --- | --- |
| **Visit  Response** | **ACI-35.030 300 µg (N=6)** | **ACI-35.030 900 µg (N=19)** | **ACI-35.030 1800 µg (N=6)** | **JACI-35.054 15 µg (N=6)** | **JACI-35.054 60 µg (N=6)** | **Pooled Placebo (N=14)** |  |
| Visit 2 (Week 2) |  |  |  |  |  |  |  |
| n | 6 | 19 | 6 | 6 | 6 | 14 |  |
| Mean (std) | 3.6 (3.66) | 3.8 (2.09) | 2.6 (0.85) | 0.9 (0.20) | 1.2 (0.72) | 1.1 (0.20) |  |
| Visit 4 (Week 10) |  |  |  |  |  |  |  |
| N | 6 | 19 | 6 | 6 | 6 | 14 |  |
| Mean (std) | 11.0 (13.04) | 11.9 (16.98) | 10.9 (13.37) | 12.3 (12.32) | 10.8 (11.28) | 1.0 (0.16) |  |
| Visit 6 (Week 26) |  |  |  |  |  |  |  |
| N | 1 | 18 | 6 | 6 | 6 | 12 |  |
| Mean (std) | 4.3 (-) | 12.0 (25.34) | 10.0 (12.81) | 21.2 (21.24) | 10.2 (5.44) | 1.1 (0.38) |  |
| Visit 9 (Week 50) |  |  |  |  |  |  |  |
| N | 4 | 18 | 5 | 6 | 6 | 14 |  |
| Mean (std) | 5.9 (4.42) | 12.6 (29.99) | 8.9 (9.78) | 17.7 (12.99) | 22.9 (20.86) | 1.1 (0.30) |  |
| Visit 11  (FU, Week 74) |  |  |  |  |  |  |  |
| N | 4 | 17 | 5 | 6 | 6 | 14 |  |
| Mean (std) | 2.1 (1.47) | 5.8 (8.06) | 5.1 (5.65) | 5.3 (3.17) | 4.6 (2.62) | 1.0 (0.30) |  |
| **Table S2: Anti-ePHF IgG (AU/mL) Titres in Serum – Fold-change from Baseline – Cohort 1 and 2 (Intention-to-Treat Population).** FU=Follow-up; ePHF=enriched Paired Helical Filaments; IgG=immunoglobulin G; std=standard deviation. | | | | | | |  |

|  | | | | | | | |
| --- | --- | --- | --- | --- | --- | --- | --- |
| **Visit  Response** | **ACI-35.030 300 µg (N=6)** | **ACI-35.030 900 µg (N=19)** | **ACI-35.030 1800 µg (N=6)** | **JACI-35.054 15 µg (N=6)** | **JACI-35.054 60 µg (N=6)** | **Pooled Placebo (N=14)** |  |
| Visit 2 (Week 2) |  |  |  |  |  |  |  |
| n | 6 | 19 | 6 | 6 | 6 | 14 |  |
| Mean (std) | 13.0 (18.57) | 138.9 (409.69) | 37.8 (48.54) | 4.0 (2.53) | 49.6 (107.46) | 0.9 (0.14) |  |
| Visit 4 (Week 10) |  |  |  |  |  |  |  |
| N | 6 | 19 | 6 | 6 | 6 | 14 |  |
| Mean (std) | 7.7 (11.35) | 31.8 (69.14) | 13.0 (11.16) | 809.1 (934.23) | 137.9 (78.29) | 1.0 (0.25) |  |
| Visit 6 (Week 26) |  |  |  |  |  |  |  |
| N | 1 | 18 | 6 | 6 | 6 | 12 |  |
| Mean (std) | 1.1 (-) | 6.3 (6.87) | 7.5 (6.42) | 1390.7 (1855.94) | 241.2 (177.28) | 1.2 (0.38) |  |
| Visit 9 (Week 50) |  |  |  |  |  |  |  |
| N | 4 | 18 | 5 | 6 | 6 | 14 |  |
| Mean (std) | 3.3 (2.84) | 5.8 (7.35) | 9.1 (12.24) | 1413.5 (1572.01) | 268.1 (238.97) | 1.1 (0.53) |  |
| Visit 11  (FU, Week 74) |  |  |  |  |  |  |  |
| N | 4 | 17 | 5 | 6 | 6 | 14 |  |
| Mean (std) | 1.1 (0.22) | 2.2 (2.02) | 3.4 (3.25) | 317.0 (271.20) | 87.8 (59.48) | 1.1 (0.47) |  |
| **Table S3: Anti-Tau IgG (AU/mL) Titres in Serum – Fold-change from Baseline – Cohort 1 and 2 (Intention-to-Treat Population).** FU=Follow-up; IgG=immunoglobulin G; std=standard deviation. | | | | | | |  |

|  | | | | | | | |
| --- | --- | --- | --- | --- | --- | --- | --- |
| **Visit  Response** | **ACI-35.030 300 µg (N=6)** | **ACI-35.030 900 µg (N=19)** | **ACI-35.030 1800 µg (N=6)** | **JACI-35.054 15 µg (N=6)** | **JACI-35.054 60 µg (N=6)** | **Pooled Placebo (N=14)** |  |
| Visit 2 (Week 2) |  |  |  |  |  |  |  |
| n | 6 | 19 | 6 | 6 | 6 | 14 |  |
| Positive (%) | 6 (100) | 19 (100) | 6 (100) | 3 (50.0) | 4 (66.7) | 0 |  |
| Visit 3 (Week 8) |  |  |  |  |  |  |  |
| n | 6 | 19 | 6 | 6 | 6 | 14 |  |
| Positive (%) | 6 (100) | 19 (100) | 6 (100) | 4 (66.7) | 5 (83.3) | 0 |  |
| Visit 4 (Week 10) |  |  |  |  |  |  |  |
| n | 6 | 19 | 6 | 6 | 6 | 14 |  |
| Positive (%) | 6 (100) | 19 (100) | 6 (100) | 6 (100) | 6 (100) | 0 |  |
| Visit 4.1 (Week 15) |  |  |  |  |  |  |  |
| n | 0 | 13 | 4 | 0 | 6 | 8 |  |
| Positive (%) | 0 | 13 (100) | 4 (100) | 0 | 6 (100) | 1 (12.5) |  |
| Visit 4.2 (Week 20) |  |  |  |  |  |  |  |
| n | 0 | 13 | 4 | 0 | 6 | 8 |  |
| Positive (%) | 0 | 13 (100) | 4 (100) | 0 | 6 (100) | 1 (12.5) |  |
| Visit 5 (Week 24) |  |  |  |  |  |  |  |
| n | 1 | 19 | 6 | 6 | 6 | 12 |  |
| Positive (%) | 1 (100) | 19 (100) | 6 (100) | 6 (100) | 6 (100) | 0 |  |
| Visit 6 (Week 26) |  |  |  |  |  |  |  |
| n | 1 | 18 | 6 | 6 | 6 | 12 |  |
| Positive (%) | 1 (100) | 18 (100) | 6 (100) | 6 (100) | 6 (100) | 1 (8.3) |  |
| Visit 6.1 (Week 31) |  |  |  |  |  |  |  |
| n | 0 | 12 | 5 | 1 | 6 | 8 |  |
| Positive (%) | 0 | 12 (100) | 5 (100) | 1 (100) | 6 (100) | 2 (25.0) |  |
| Visit 7 (Week 36) |  |  |  |  |  |  |  |
| n | 6 | 18 | 6 | 6 | 6 | 14 |  |
| Positive (%) | 4 (66.7) | 17 (94.4) | 6 (100) | 6 (100) | 6 (100) | 0 |  |
| Visit 7.1 (Week 42) |  |  |  |  |  |  |  |
| n | 0 | 10 | 6 | 4 | 6 | 10 |  |
| Positive (%) | 0 | 10 (100) | 6 (100) | 4 (100) | 6 (100) | 1 (10.0) |  |
| Visit 8 (Week 48) |  |  |  |  |  |  |  |
| n | 4 | 18 | 5 | 6 | 5 | 14 |  |
| Positive (%) | 2 (50.0) | 17 (94.4) | 5 (100) | 6 (100) | 5 (100) | 1 (7.1) |  |
| Visit 9 (Week 50) |  |  |  |  |  |  |  |
| n | 4 | 18 | 5 | 6 | 6 | 14 |  |
| Positive (%) | 4 (100) | 18 (100) | 5 (100) | 6 (100) | 6 (100) | 1 (7.1) |  |
| Visit 10 (FU, Week 67) |  |  |  |  |  |  |  |
| n | 4 | 17 | 5 | 6 | 6 | 14 |  |
| Positive (%) | 4 (100) | 17 (100) | 5 (100) | 6 (100) | 6 (100) | 1 (7.1) |  |
| Visit 11 (FU, Week 74) |  |  |  |  |  |  |  |
| n | 4 | 17 | 5 | 6 | 6 | 14 |  |
| Positive (%) | 4 (100) | 16 (94.1) | 5 (100) | 6 (100) | 6 (100) | 0 |  |
| **Table S4: Anti-pTau IgG (AU/mL) Titres in Serum – Responder Analysis – Cohort 1 and 2 (Intention-to-Treat Population).** FU=Follow-up; IgG=immunoglobulin G; pTau=phospho-Tau. Note: The determination of antibody response being negative/positive was done using a threshold factor defined from samples from human donors obtained during assay validation. Baseline titres were multiplied with this threshold factor (1.81 for anti-pTau IgG). If the post-baseline result was greater than or equal this value, the antibody response was considered positive; otherwise, it was deemed negative. Percentages were based on the number of participants with data at each visit (n). | | | | | | |  |

|  | | | | | | | |
| --- | --- | --- | --- | --- | --- | --- | --- |
| **Visit  Response** | **ACI-35.030 300 µg (N=6)** | **ACI-35.030 900 µg (N=19)** | **ACI-35.030 1800 µg (N=6)** | **JACI-35.054 15 µg (N=6)** | **JACI-35.054 60 µg (N=6)** | **Pooled Placebo (N=14)** |  |
| Visit 2 (Week 2) |  |  |  |  |  |  |  |
| n | 6 | 19 | 6 | 6 | 6 | 14 |  |
| Positive (%) | 4 (66.7) | 14 (73.7) | 4 (66.7) | 0 | 1 (16.7) | 0 |  |
| Visit 3 (Week 8) |  |  |  |  |  |  |  |
| n | 6 | 19 | 6 | 6 | 6 | 14 |  |
| Positive (%) | 5 (83.3) | 14 (73.7) | 2 (33.3) | 0 | 1 (16.7) | 0 |  |
| Visit 4 (Week 10) |  |  |  |  |  |  |  |
| n | 6 | 19 | 6 | 6 | 6 | 14 |  |
| Positive (%) | 5 (83.3) | 18 (94.7) | 5 (83.3) | 4 (66.7) | 5 (83.3) | 0 |  |
| Visit 4.1 (Week 15) |  |  |  |  |  |  |  |
| n | 0 | 13 | 4 | 0 | 6 | 8 |  |
| Positive (%) | 0 | 11 (84.6) | 3 (75.0) | 0 | 3 (50.0) | 0 |  |
| Visit 4.2 (Week 20) |  |  |  |  |  |  |  |
| n | 0 | 13 | 4 | 0 | 6 | 8 |  |
| Positive (%) | 0 | 11 (84.6) | 3 (75.0) | 0 | 3 (50.0) | 0 |  |
| Visit 5 (Week 24) |  |  |  |  |  |  |  |
| n | 1 | 19 | 6 | 6 | 6 | 12 |  |
| Positive (%) | 1 (100) | 15 (78.9) | 3 (50.0) | 3 (50.0) | 3 (50.0) | 0 |  |
| Visit 6 (Week 26) |  |  |  |  |  |  |  |
| n | 1 | 18 | 6 | 6 | 6 | 12 |  |
| Positive (%) | 1 (100) | 17 (94.4) | 4 (66.7) | 5 (83.3) | 6 (100) | 0 |  |
| Visit 6.1 (Week 31) |  |  |  |  |  |  |  |
| n | 0 | 12 | 5 | 1 | 6 | 8 |  |
| Positive (%) | 0 | 11 (91.7) | 3 (60.0) | 1 (100) | 6 (100) | 0 |  |
| Visit 7 (Week 36) |  |  |  |  |  |  |  |
| n | 6 | 18 | 6 | 6 | 6 | 14 |  |
| Positive (%) | 2 (33.3) | 13 (72.2) | 3 (50.0) | 5 (83.3) | 6 (100) | 0 |  |
| Visit 7.1 (Week 42) |  |  |  |  |  |  |  |
| n | 0 | 10 | 6 | 4 | 6 | 10 |  |
| Positive (%) | 0 | 8 (80.0) | 4 (66.7) | 2 (50.0) | 5 (83.3) | 0 |  |
| Visit 8 (Week 48) |  |  |  |  |  |  |  |
| n | 4 | 18 | 5 | 6 | 5 | 14 |  |
| Positive (%) | 1 (25.0) | 13 (72.2) | 2 (40.0) | 4 (66.7) | 3 (60.0) | 0 |  |
| Visit 9 (Week 50) |  |  |  |  |  |  |  |
| n | 4 | 18 | 5 | 6 | 6 | 14 |  |
| Positive (%) | 3 (75.0) | 16 (88.9) | 4 (80.0) | 5 (83.3) | 6 (100) | 0 |  |
| Visit 10 (FU, Week 67) |  |  |  |  |  |  |  |
| n | 4 | 17 | 5 | 6 | 6 | 14 |  |
| Positive (%) | 2 (50.0) | 14 (82.4) | 2 (40.0) | 4 (66.7) | 5 (83.3) | 0 |  |
| Visit 11 (FU, Week 74) |  |  |  |  |  |  |  |
| n | 4 | 17 | 5 | 6 | 6 | 14 |  |
| Positive (%) | 1 (25.0) | 12 (70.6) | 2 (40.0) | 4 (66.7) | 5 (83.3) | 0 |  |
| **Table S5: Anti-ePHF IgG (AU/mL) Titres in Serum – Responder Analysis – Cohort 1 and 2 (Intention-to-Treat Population).** FU=Follow-up; IgG=immunoglobulin G; ePHF=enriched paired helical filaments. Note: The determination of antibody response being negative/positive was done using a threshold factor defined from samples from human donors obtained during assay validation. Baseline titres were multiplied with this threshold factor (2.21 for anti-ePHF IgG). If the post-baseline result was greater than or equal this value, the antibody response was considered positive; otherwise, it was deemed negative. Percentages were based on the number of participants with data at each visit (n). | | | | | | |  |

|  | | | | | | | |
| --- | --- | --- | --- | --- | --- | --- | --- |
| **Visit  Response** | **ACI-35.030 300 µg (N=6)** | **ACI-35.030 900 µg (N=19)** | **ACI-35.030 1800 µg (N=6)** | **JACI-35.054 15 µg (N=6)** | **JACI-35.054 60 µg (N=6)** | **Pooled Placebo (N=14)** |  |
| Visit 2 (Week 2) |  |  |  |  |  |  |  |
| n | 6 | 19 | 6 | 6 | 6 | 14 |  |
| Positive (%) | 5 (83.3) | 17 (89.5) | 5 (83.3) | 4 (66.7) | 4 (66.7) | 0 |  |
| Visit 3 (Week 8) |  |  |  |  |  |  |  |
| n | 6 | 19 | 6 | 6 | 6 | 14 |  |
| Positive (%) | 2 (33.3) | 12 (63.2) | 3 (50.0) | 5 (83.3) | 4 (66.7) | 0 |  |
| Visit 4 (Week 10) |  |  |  |  |  |  |  |
| n | 6 | 19 | 6 | 6 | 6 | 14 |  |
| Positive (%) | 2 (33.3) | 15 (78.9) | 4 (66.7) | 6 (100) | 6 (100) | 0 |  |
| Visit 4.1 (Week 15) |  |  |  |  |  |  |  |
| n | 0 | 13 | 4 | 0 | 6 | 8 |  |
| Positive (%) | 0 | 8 (61.5) | 2 (50.0) | 0 | 6 (100) | 0 |  |
| Visit 4.2 (Week 20) |  |  |  |  |  |  |  |
| n | 0 | 13 | 4 | 0 | 6 | 8 |  |
| Positive (%) | 0 | 7 (53.8) | 1 (25.0) | 0 | 6 (100) | 0 |  |
| Visit 5 (Week 24) |  |  |  |  |  |  |  |
| n | 1 | 19 | 6 | 6 | 6 | 12 |  |
| Positive (%) | 0 | 8 (42.1) | 1 (16.7) | 6 (100) | 6 (100) | 0 |  |
| Visit 6 (Week 26) |  |  |  |  |  |  |  |
| n | 1 | 18 | 6 | 6 | 6 | 12 |  |
| Positive (%) | 0 | 10 (55.6) | 3 (50.0) | 6 (100) | 6 (100) | 0 |  |
| Visit 6.1 (Week 31) |  |  |  |  |  |  |  |
| n | 0 | 12 | 5 | 1 | 6 | 8 |  |
| Positive (%) | 0 | 7 (58.3) | 2 (40.0) | 1 (100) | 6 (100) | 0 |  |
| Visit 7 (Week 36) |  |  |  |  |  |  |  |
| n | 6 | 18 | 6 | 6 | 6 | 14 |  |
| Positive (%) | 1 (16.7) | 4 (22.2) | 1 (16.7) | 6 (100) | 6 (100) | 0 |  |
| Visit 7.1 (Week 42) |  |  |  |  |  |  |  |
| n | 0 | 10 | 6 | 4 | 6 | 10 |  |
| Positive (%) | 0 | 3 (30.0) | 1 (16.7) | 4 (100) | 6 (100) | 0 |  |
| Visit 8 (Week 48) |  |  |  |  |  |  |  |
| n | 4 | 18 | 5 | 6 | 5 | 14 |  |
| Positive (%) | 0 | 4 (22.2) | 1 (20.0) | 6 (100) | 5 (100) | 0 |  |
| Visit 9 (Week 50) |  |  |  |  |  |  |  |
| n | 4 | 18 | 5 | 6 | 6 | 14 |  |
| Positive (%) | 1 (25.0) | 7 (38.9) | 4 (80.0) | 6 (100) | 6 (100) | 0 |  |
| Visit 10 (FU, Week 67) |  |  |  |  |  |  |  |
| n | 4 | 17 | 5 | 6 | 6 | 14 |  |
| Positive (%) | 0 | 4 (23.5) | 1 (20.0) | 6 (100) | 6 (100) | 0 |  |
| Visit 11 (FU, Week 74) |  |  |  |  |  |  |  |
| n | 4 | 17 | 5 | 6 | 6 | 14 |  |
| Positive (%) | 0 | 3 (17.6) | 1 (20.0) | 6 (100) | 6 (100) | 0 |  |
| **Table S6: Anti-Tau IgG (AU/mL) Titres in Serum – Responder Analysis – Cohort 1 and 2 (Intention-to-Treat Population).** FU=Follow-up; IgG=immunoglobulin G. Note: The determination of antibody response being negative/positive was done using a threshold factor defined from samples from human donors obtained during assay validation. Baseline titres were multiplied with this threshold factor (3.38 for anti-Tau IgG). If the post-baseline result was greater than or equal this value, the antibody response was considered positive; otherwise, it was deemed negative. Percentages were based on the number of participants with data at each visit (n). | | | | | | |  |

| **Visit**  **Response** | **ACI-35.030 300 µg (N=6)** | **ACI-35.030 900 µg (N=19)** | **ACI-35.030 1800 µg (N=6)** | **JACI-35.054 15 µg (N=6)** | **JACI-35.054 60 µg (N=6)** | **Pooled Placebo (N=14)** |
| --- | --- | --- | --- | --- | --- | --- |
| Baseline |  |  |  |  |  |  |
| n | 6 | 19 | 6 | 6 | 6 | 14 |
| Suicidal Ideation |  |  |  |  |  |  |
| Positive (%) | 0 | 0 | 0 | 0 | 0 | 0 |
| Suicidal Behavior |  |  |  |  |  |  |
| Positive (%) | 0 | 0 | 0 | 0 | 0 | 0 |
| Suicidal Ideation or Behavior |  |  |  |  |  |  |
| Positive (%) | 0 | 0 | 0 | 0 | 0 | 0 |
| Visit 6 (Week 26) |  |  |  |  |  |  |
| n | 3 | 19 | 6 | 6 | 6 | 14 |
| Suicidal Ideation |  |  |  |  |  |  |
| Positive (%) | 0 | 0 | 0 | 0 | 0 | 0 |
| Suicidal Behavior |  |  |  |  |  |  |
| Positive (%) | 0 | 0 | 0 | 0 | 0 | 0 |
| Suicidal Ideation or Behavior |  |  |  |  |  |  |
| Positive (%) | 0 | 0 | 0 | 0 | 0 | 0 |
| Visit 9 (Week 50) |  |  |  |  |  |  |
| n | 4 | 18 | 5 | 6 | 6 | 14 |
| Suicidal Ideation |  |  |  |  |  |  |
| Positive (%) | 0 | 0 | 0 | 1 (16.6) | 0 | 0 |
| Suicidal Behavior |  |  |  |  |  |  |
| Positive (%) | 0 | 0 | 0 | 0 | 0 | 0 |
| Suicidal Ideation or Behavior |  |  |  |  |  |  |
| Positive (%) | 0 | 0 | 0 | 1 (16.6) | 0 | 0 |
| Visit 11 (FU,Week 74) |  |  |  |  |  |  |
| n | 4 | 17 | 5 | 6 | 6 | 14 |
| Suicidal Ideation |  |  |  |  |  |  |
| Positive (%) | 0 | 0 | 0 | 0 | 0 | 0 |
| Suicidal Behavior |  |  |  |  |  |  |
| Positive (%) | 0 | 0 | 0 | 0 | 0 | 0 |
| Suicidal Ideation or Behavior |  |  |  |  |  |  |
| Positive (%) | 0 | 0 | 0 | 0 | 0 | 0 |
| **Table S7: Columbia-Suicide Severity Rating Scale (C-SSRS) – Frequency Analysis – Cohort 1 and 2 (Intention to Treat Population).** FU=Follow-up; Percentages were based on the number of participants with data at each visit (n). | | | | | | |
