# Supplementary material for: Safety and immunogenicity of two Tau-targeting active immunotherapies, ACI-35.030 and JACI-35.054, in participants with early Alzheimer's disease: a phase 1b/2a, multicentre, double-blind, randomised, placebo-controlled study
Source: eBioMedicine. 2025 Sep 18;120:105940. doi: 10.1016/j.ebiom.2025.105940 (PMC12481106; doi:10.1016/j.ebiom.2025.105940)
Supplement: Clinical Study Protocol [file mmc2.pdf]

CLINICAL STUDY PROTOCOL

A Phase Ib/IIa Multicenter, Double-Blind, Randomized, Placebo-Controlled Study to Evaluate the Safety, Tolerability and Immunogenicity of Different Doses, Regimens and Combinations of Tau Targeted Vaccines in Subjects with Early Alzheimer's Disease

Study No ACI-35-1802

Protocol version 7.0, 22 October 2021

EudraCT Number 2018-004573-27

-CONFIDENTIAL-

This document and its contents are the property of and confidential to AC Immune SA. Any unauthorized copying or use of this document is prohibited.

SIGNATURE PAGE

Sponsor

[redacted]
AC Immune SA
EPFL Innovation Park - Building G
CH-1015 Lausanne, Switzerland
Tel: [redacted]
Fax: [redacted]
email: [redacted]

____________________ Signature ____________ Date

[redacted]
AC Immune SA
EPFL Innovation Park - Building G
CH-1015 Lausanne, Switzerland
Tel : [redacted]
Fax : [redacted]
email: [redacted]

____________________ Signature ____________ Date

International Coordinating Investigator

[redacted]
Amsterdam UMC
Alzheimer Center
de Boelelaan 1118
1081 HZ Amsterdam
The Netherlands
Tel: [redacted]
Mob: [redacted]
email: [redacted]

________________________ ________________

Signature Date

Principal Investigator

Site Principal Investigator

I agree with the content of this protocol and the nature of the documentation made as part of this study. I have read this protocol, I understand its content and I will work according to this protocol and according to the principles of Good Clinical Practices.

Printed Name	Signature	Date

STUDY SYNOPSIS

**Study Title**	A Phase Ib/IIa multicenter, double blind, randomized, placebo-controlled study to evaluate the safety, tolerability and immunogenicity of different doses, regimens and combinations of Tau targeted vaccines in subjects with early Alzheimer's Disease
**Study number**	ACI-35-1802
**EudraCT number**	2018-004573-27
**Investigators/study Centers**	This study will be conducted in approximately 15 study centers located in Europe.
**Objectives**	**Primary Objectives**<br>To assess the safety and tolerability of study vaccines<br>To assess the immunogenicity of study vaccines (induction of IgG titers against phosphorylated Tau (pTau) in serum)<br>**Secondary Objectives**<br>To further assess the immunogenicity of study vaccines (induction of IgG titers against Tau and IgM titers against pTau and Tau in serum)<br>To assess the avidity of antibodies elicited by immunization<br>**Exploratory Objectives**<br>To explore the effect of study vaccines on putative biomarkers of the progression of Alzheimer's Disease (AD), i.e. blood and/or CSF concentrations of total Tau and pTau proteins, Tau fragments and other related biomarkers<br>To explore the effect of study vaccines on the activation of T-cells in blood<br>To explore the activity of study vaccines on blood inflammatory cytokines (e.g. IL-1B, IL-2, IL-6, IL-8, IL-10, IFN- γ, and TNF- α)<br>To further explore the effect of study vaccines on the immune response (e.g. antibodies against vaccine components, functional capacity of vaccine-induced antibodies)<br>To explore the effect of study vaccines on behavior, cognitive (including, in the Netherlands, the maintenance of the decisional capacity) and functional performance
**Study Endpoints**	**Primary Endpoints**<br>**Safety and tolerability**: Adverse events, immediate and delayed reactogenicity (e.g. anaphylaxis, local and systemic reactogenicity, including immune-complex disease); suicidal ideation (C-SSRS); behavior (NPI); cognitive and functional assessments (RBANS, CDR-SB) to assess safety; vital signs; MRI imaging; electrocardiogram; routine hematology and biochemistry evaluation in blood and urine; evaluation of autoimmune antibodies including anti-dsDNA antibodies in blood; inflammatory markers in blood and CSF<br>**Immune response (i.e. immunogenicity)**: Anti-pTau IgG titers in serum (geometric mean, change from baseline, responder rate, peak and area under the curve)<br>**Secondary Endpoints**<br>**Immune response (i.e. immunogenicity)**: Anti-Tau IgG, anti-pTau and anti-Tau IgM titers in serum (geometric mean, change from baseline, responder rate, peak and area under the curve), determination of the IgG response profile by avidity testing<br>**Exploratory Endpoints**<br>Change from baseline of putative AD biomarker titers in blood and/or CSF (e.g. total Tau, pTau, Tau fragments and other related biomarkers), change from baseline in T-cell activation levels as measured in blood, change from baseline of inflammatory cytokine titers in blood, change from baseline in antibody titers in blood, change from baseline in behavior (NPI), cognitive (including the proportion of subjects maintaining their decisional capacity during the study using the MacCAT-CR interview in the Netherlands) and functional performance (RBANS, CDR-SB) scores

**Design**	This protocol is a master protocol aimed at investigating two different Tau targeted study vaccines.<br>This study is a multicenter prospective placebo-controlled, double-blind and randomized study to assess the safety, tolerability and immunogenicity of different doses, and combinations of Tau targeted vaccines versus placebo over 50 weeks (i.e. 12 months). Immunizations will be performed at months 0 (Week 0), 2 (Week 8), 6 (Week 24)* and 12 (Week 48).<br>***Due to the Covid-19 pandemic, local travel restrictions in Finland effective as of 18 March 2020, and the need to preserve the safety of study participants, modifications are made to the schedule of immunizations of sub-cohort 1.1 subjects not having received the 3rd injection at month 6 already:** immunizations at month 6 (week 24) will not be performed in 7 of 8 sub-cohort 1.1 subjects for whom the planned Visit 5 does not allow all assessments to be done according to the protocol. In addition, Visits 5 (week 24), 6 (week 26) and 7 (week 36) are replaced by remote visits in case these fall during the at-risk period (refer to sections 3.5.2. 10, 3.5.2.11, 3.5.2.12 and 3.5.2.14).<br>Due to the resurgence of the Covid-19 pandemic in multiple participating countries since September 2020, additional adjustments related to the mode of administration of study assessments including study vaccine administration may apply in Finland and other participating countries after the above measures were implemented in Finland.<br><br>**Cohort 1 with ACI-35.030:**<br>Up to 3 dose levels of ACI-35.030 administered by the intramuscular route will be tested. The rationale for dose escalation is to obtain a dose response curve of both safety and immunogenicity in order to select the optimal dose and combination for future studies.<br><br>**Sub-cohort 1.1 (8 subjects):**<br>ACI-35.030 at 300 µg/dose will be administered to 6 subjects and the placebo will be administered to 2 subjects. The safety and tolerability data after all subjects have received the second injection in sub-cohort 1.1 will permit dose escalation after review by the Data Safety Monitoring Board (DSMB).<br><br>**Sub-cohort 1.2 (8 subjects):**<br>ACI-35.030 at 900 µg/dose will be administered to 6 subjects and the placebo will be administered to 2 subjects. The safety and tolerability data after all subjects have received the second injection in sub-cohort 1.2 will permit dose escalation after review by the DSMB.<br><br>**Sub-cohort 1.3 (8 subjects):**<br>ACI-35.030 at 1800 µg/dose will be administered to 6 subjects and the placebo will be administered to 2 subjects.<br><br>**Cohort 2 with JACI-35.054:**<br>The administration schedule, route of administration, duration of treatment and follow-up are identical to cohort 1. Up to 3 dose levels of JACI-35.054 administered by the intramuscular route will be tested in up to 3 sub-cohorts.<br><br>**Sub-cohort 2.1 (8 subjects):**<br>JACI-35.054 at 15 µg/dose will be administered to 6 subjects and the placebo will be administered to 2 subjects. The safety and tolerability data after all subjects have received the second injection in sub-cohort 2.1 will permit dose escalation after review by DSMB.

	**Sub-cohort 2.2 (8 subjects) - Optional:**<br>JACI-35.054 at up to 60 µg/dose will be administered to 6 subjects and the placebo will be administered to 2 subjects. This sub-cohort will be optional and may be conducted based on good safety and tolerability observed in sub-cohort 2.1 and in case the antibody response in this previous sub-cohort is anticipated to be optimized at the dosage of up to 60 µg.<br><br>**Sub-cohort 2.3 (8 subjects) - Optional**<br>JACI-35.054 at up to 150 µg/dose will be administered in 6 subjects and the placebo will be administered in 2 subjects. This sub-cohort will be optional and may be conducted based on good safety and tolerability observed in sub-cohort 2.2 and in case the antibody response in this previous sub-cohort is anticipated to be further optimized at a higher dosage.<br><br>**Cohort 3 with ACI-35.030 and JACI-35.054 in sequential administration**<br>The administration schedule, route of administration, duration of treatment and follow-up are identical to cohorts 1 and 2 but both vaccines ACI-35.030 and JACI-35.054 will be administered in a sequence based on non-human primate immunogenicity data and on data reviewed during the previous sub-cohort interim analyses. In any of the sub-cohorts listed below, the selected dosages of the 2 study vaccines will have been evaluated in cohorts 1 and 2 and safety monitored for at least 10 weeks and safety data of the selected dosages of the 2 study vaccines reviewed by the DSMB.<br>**Sub-cohort 3.1 (8 subjects)**<br>ACI-35.030 and JACI-35.054 at dosages previously evaluated in cohorts 1 and 2 respectively and found to be safe after a study treatment period of at least 10 weeks will be administered sequentially to 6 subjects and placebo will be administered at each dosing occasion to 2 subjects. Immunizations with ACI-35.030 will be performed at months 0 (Week 0) and 2 (Week 8), and with JACI-35.054 at month 6 (Week 24) and 12 (Week 48).<br><br>**Sub-cohort 3.2 (8 subjects) - Optional**<br>This optional sub-cohort may include a different sequence of administration than in sub-cohort 3.1 and/or different dosages of ACI-35.030 and/or JACI-35.054 based on available safety, tolerability and immunogenicity data from previous sub-cohort interim analyses. Dosages of ACI-35.030 and of JACI-35.054 will have been previously evaluated in cohorts 1, 2 and/or 3 without significant safety or tolerability concerns.<br><br>**Sub-cohort 3.3 (8 subjects) - Optional**<br>This optional sub-cohort may include a different sequence of administration than in sub-cohorts 3.1 and 3.2 and/or different dosages of ACI-35.030 and/or JACI-35.054 based on available safety, tolerability and immunogenicity data from previous sub-cohort interim analyses. Dosages of ACI-35.030 and of JACI-35.054 will have been previously evaluated in cohorts 1, 2 and/or 3 without significant safety or tolerability concerns.<br><br>**Sub-cohort expansion:**<br>Optional sub-cohort expansion of up to 16 additional subjects (i.e. up to 12 on active treatment and up to 4 on placebo per sub-cohort) may be considered in sub-cohort(s) with a favorable safety and immunogenicity profile with the objective to confirm the initial sub-cohort(s) immunogenicity results. The number of additional subjects to be enrolled will be determined based on the observed immunogenicity response rate in the sub-cohort(s) and on the potential gain in precision of the response rate estimate in the expanded sub-cohort(s), and will not exceed 16 (i.e., up to 24 subjects overall per sub-cohort). In addition, all other immunogenicity endpoints available at the interim

	timepoint may be considered. The dose-level, sequence of administration and administration regimen of study vaccine(s) will be identical to the initial part of the sub-cohort.<br><br>**Safety follow-up:**<br>All subjects will be kept under clinical observation for 24 hours after the first administration of study vaccine and for 4 hours after subsequent administrations of study vaccine. During that period, the vital signs will be monitored and the injection site will be observed on a regular basis. A physical examination will be performed prior to discharge. The clinical observation may be prolonged in case of safety concerns according to the clinical judgement of the investigator. A subsequent safety assessment will also be performed 48 to 72 hours after each immunization by telephone call for all study subjects. For adverse events considered possibly or probably related to the study vaccine of greater than mild severity according to investigator assessment, subjects will be required to return to the clinic for additional safety assessment. In each sub-cohort, the first dosing of the first 4 subjects should be performed once the safety assessment at 48 to 72 hours of the previous subject has been performed. This will allow the site principal investigator to assess whether there is any clinically relevant safety issue related to study vaccine prior to dosing the subsequent subject. Safety laboratory samples will be collected at baseline, prior to each injection and 2-4 weeks after each injection. Laboratory results will be reviewed by the Medical Monitor on a periodic basis including all out of range values.<br>All treated subjects will have a safety follow-up period of 24 weeks (6 months) after the end of the treatment period. During this period, subjects will be asked to attend a first follow-up visit 19 weeks after the last administration and a last visit at the end of the follow-up period (26 weeks after the last administration). Participants' safety will be monitored throughout the study with regular review of safety data by the DSMB.<br><br>**Post-injection period extension (optional):**<br>Based on immunogenicity data, subjects from sub-cohort(s) may be invited to attend two additional visits (at Week 86 and Week 98) taking place after the end of the safety follow-up period (week 74) in order to evaluate the durability of the immune response and collect additional biomarker data. All subjects participating in this post-injection period extension will have to reconsent. During this additional 24-week (6 month) period, blood will be collected for immunogenicity and biomarker testing. The aim of this optional study extension is to help inform the dosing regimen in subsequent clinical trials of the vaccine(s). AEs/SAEs and concomitant medications (new and/or changes from the previous visit) will also be collected during these 2 additional visits. Upon investigator's medical judgement, other assessments can be performed during this period (e.g., MRI, ECG, etc.) if clinically indicated.<br>In the Netherlands, the subjects' decisional capacity will be re-assessed before consenting for this optional post-injection extension.<br><br>**Interim analyses (IAs)**<br>Interim analyses of safety, tolerability and immunogenicity data may be conducted in each sub-cohort, including expanded sub-cohorts, as follows:<br>- all available subjects in the sub-cohort have completed Visit 4 (Week 10), i.e. 2 to 4 weeks after the second injection<br>- all available subjects in the sub-cohort have completed Visit 6 (Week 26), i.e. 2 to 4 weeks after the third injection<br>- all available subjects in the sub-cohort have completed Visit 9 (Week 50), i.e. 2 to 4 weeks after the last injection at Week 48<br>- all available subjects in the sub-cohort have completed Visit 11 (Week 74), i.e. end of the safety follow-up period

	If the post-injection period is extended in any sub-cohort, IA of immunogenicity data may also be conducted at Visit 13 (Week 98), and optionally at Visit 12 (Week 86).<br>Available biomarker data may also be reviewed during any of these IAs. The IAs described above may also be performed for any expanded sub-cohort.<br>Additional IAs to review the sustainability of immune response data may be conducted between weeks 26 and 50 and between weeks 50 and 74.<br>Independently of the above-mentioned interim analyses, the DSMB will perform regular reviews of the safety and tolerability data throughout the study.<br><br>**Rationale and scope of IAs:**<br>The objectives of the above mentioned IAs in the sub-cohorts (whether expanded or not) is to ensure a regular review of safety, tolerability and immunogenicity with the support of optional biomarker data. A decision to escalate to the next dose in one given sub-cohort may be taken once IA data of the previous dose are available from Visit 4 (Week 10) onwards and pending prior DSMB approval. A decision to expand a sub-cohort may be taken based on satisfactory review of safety data by the DSMB and analysis of immunogenicity IA data of this sub-cohort dose from Visit 4 (Week 10) onwards. The IA at the end of the optional extended post-injection period is intended to monitor the durability of the immune response and any biomarker changes in blood.
**No of subjects**	The number of subjects to be enrolled will be dependent upon the overall number of sub-cohorts as described below, and the number of sub-cohorts in which expansion occurs.<br><br>A minimum of 40 subjects will be enrolled in the three cohorts (8 subjects in each sub-cohort 1.1, 1.2 and 1.3 in Cohort 1, and sub-cohorts 2.1 and 3.1 respectively in Cohorts 2 and 3). The total number of subjects per cohort would be 72 if all 3 sub-cohorts in each cohort are expanded up to 24 subjects. Potential expansion is anticipated only in a limited number of sub-cohorts. However, if all sub-cohorts were to be expanded, this would lead to a total of 216 subjects. The number of subjects in each of the cohorts and potential sub-cohorts is further described below.<br><br>**Cohort 1 (ACI-35.030)**: Up to 72 subjects depending on dose escalation and a decision to expand one or more sub-cohort(s):<br>• 8 subjects in sub-cohort 1.1 with 6 subjects on ACI-35.030 at 300 µg and 2 on placebo;<br>• 8 subjects in sub-cohort 1.2 with 6 subjects on ACI-35.030 at 900 µg and 2 on placebo;<br>• 8 subjects in sub-cohort 1.3 with 6 subjects on ACI-35.030 at 1800 µg and 2 on placebo;<br>• In case of sub-cohort expansion, up to 16 additional subjects in sub-cohort 1.1, 1.2 and/or 1.3 with the same randomization ratio.<br><br>**Cohort 2 (JACI-35.054)**: Up to 72 subjects depending on dose escalation and a decision to expand one or more sub-cohort(s):<br>• 8 subjects in sub-cohort 2.1 with 6 subjects on JACI-35.054 at 15 µg and 2 on placebo;<br>• Optional sub-cohorts:<br>o 8 subjects in sub-cohort 2.2 with 6 subjects on JACI-35.054 up to 60 µg and 2 on placebo;<br>o 8 subjects in sub-cohort 2.3 with 6 subjects on JACI-35.054 up to 150 µg and 2 on placebo;<br>• In case of sub-cohort expansion, up to 16 additional subjects in sub-cohort 2.1, 2.2 and/or 2.3 with the same randomization ratio.

	**Cohort 3 (ACI-35.030 and JACI-35.054 in sequential administration):** Up to 72 subjects depending on the decision to conduct several sub-cohorts and/or to expand one or more sub-cohort(s):<br>• 8 subjects in sub-cohort 3.1 with 6 subjects on ACI-35.030 and JACI-35.054 at dosages previously evaluated in cohorts 1 and 2 respectively and found to be safe after a study treatment period of at least 10 weeks in sequential administration and 2 on placebo.<br>• Optional sub-cohorts:<br>o 8 subjects in sub-cohort 3.2 with 6 subjects on active treatment and 2 on placebo.<br>o 8 subjects in sub-cohort 3.3 with 6 subjects on active treatment and 2 on placebo.<br>• In case of sub-cohort expansion, up to 16 additional subjects in sub-cohorts 3.1, 3.2 and/or 3.3 with the same randomization ratio.<br><br>Any randomized subject withdrawing before the 2nd injection for reasons other than safety may be replaced.
**Study Population**	50-75 years of age (male and female) with a diagnosis of mild AD or MCI due to AD according to National Institute on Aging-Alzheimer's Association (NIA-AA) criteria and a Clinical Dementia Rating Scale (CDR) global score of 0.5 or 1
**Inclusion/Exclusion Criteria**	**Inclusion criteria**<br>1. Male or female with age from 50 and up to 75 years old inclusive.<br>2. Mild Cognitive Impairment (MCI) due to AD or Mild AD according to NIA-AA criteria and a Clinical Dementia Rating scale (CDR) global score of 0.5 or 1 respectively.<br>3. Mini Mental State Examination (MMSE) score of 22 or above.<br>4. Abnormal level of CSF Abeta amyloid 42 (Aß42) consistent with AD pathology at screening.<br>• In borderline cases for CSF Aß42 levels, other results may be considered to help determine amyloid positivity e.g. the Aß42/Aß40 ratio and, on a case by case basis, a history of positive amyloid PET scan or positive CSF Aß42 level.<br>• Results from CSF sampling performed within 6 months prior to screening are acceptable on a case by case basis provided that they are consistent with the presence of amyloid pathology and that the corresponding CSF sample can be used in the study for testing.<br>5. Subjects either not taking any marketed treatment for AD or receiving a stable dose of an acetylcholinesterase inhibitor and/or memantine for at least 3 months prior to baseline.<br>6. Subjects cared for by a reliable informant or caregiver to assure compliance, assist with clinical assessments and report safety issues.<br>7. Women must be post-menopausal for at least one year and/or surgically sterilized. Women of childbearing potential or not post-menopausal must have a negative blood pregnancy test at screening (blood draw between day -14 and day -3 prior to baseline) and be willing to use highly effective methods of contraception from the screening visit until the end of their participation. Urine pregnancy testing will be performed throughout the treatment period to determine if the subject can continue receiving the study vaccine. Male participants in the trial with female partners of child bearing potential are required to use barrier methods of contraception (condoms with spermicide) in addition to contraceptive measures used by female partners during the whole study duration.<br>8. Subjects who in the opinion of the investigator are able to understand and provide written informed consent. In the Netherlands, the subjects' decisional capacity will be also assessed and must be consistent with the ability to provide informed consent using the MacArthur Competency Tool for Clinical Research in order to evaluate their abilities in the areas of understanding, reasoning, appreciation and choice.<br>9. Both subject and informant or caregiver must be fluent in one of the languages of the study and able to comply with all study procedures, including lumbar punctures.

	**Exclusion criteria**<br>1. Participation in previous clinical trials for AD and/or for neurological disorders using active immunization unless there is documented evidence that the subject was treated with placebo only and the placebo vaccine is not expected to induce any specific immune response.<br>2. Participation in previous clinical trials for AD and/or for neurological disorders using any passive immunization within the past 6 months (or 5 half-lives of the investigational antibody, whichever is longer) prior to screening unless there is documented evidence that the subject was treated with placebo only and the placebo is not expected to induce any specific immune response.<br>3. Participation in previous clinical trials for AD and/or for neurological disorders using any small molecule drug including BACE-1 inhibitors within the past 3 months prior to screening.<br>4. Concomitant participation in any other clinical trial using experimental or approved medications or therapies.<br>5. Presence of positive Anti-nuclear Antibody (ANA) titers at a dilution of at least 1:160 in subjects without clinical symptoms of auto-immune disease.<br>6. Current or past history of auto-immune disease, or clinical symptoms consistent with the presence of auto-immune disease.<br>7. Immune suppression including but not limited to the use of immunosuppressive drugs or systemic steroids unless they have been prescribed transiently more than 3 months prior to screening.<br>8. History of severe allergic reaction (e.g., anaphylaxis) including but not limited to severe allergic reaction to previous vaccines and/or medications.<br>9. Prior history of clinically significant hypoglycaemic episodes.<br>10. Drug or alcohol abuse or dependence currently met or within the past five years according to Diagnostic and Statistical Manual of Mental Disorders-V (DSM-V) criteria.<br>11. Any clinically significant medical condition likely to interfere with the evaluation of safety and tolerability of the study treatment and/or the adherence to the full study visit schedule.<br>12. Any clinically significant medical condition likely to impact the immune system (e.g, any history of acquired or innate immune system disorder).<br>13. Use of hydralazine, procainamide, quinidine, isoniazide, TNF-inhibitors, minocycline within the last 12 months prior to screening.<br>14. Use of diltiazem unless on a stable dose for at least 3 months prior to screening.<br>15. Significant risk of suicide defined, using the Columbia-Suicide Severity Rating Scale, as the subject answering: “yes” to suicidal ideation questions 4 or 5 or answering: “yes” to suicidal behavior within the past 12 months.<br>16. Concomitant psychiatric or neurologic disorder other than those considered to be related to AD (e.g. head injury with loss of consciousness, symptomatic stroke, Parkinson’s disease, severe carotid occlusive disease, TIAs).<br>17. History or presence of uncontrolled seizures. If history of seizures, they must be well controlled with no occurrence of seizures within 2 years prior to screening. The use of anti-epileptic medications is permitted if at stable dose for at least 3 months prior to screening.<br>18. History of meningoencephalitis within the past 10 years prior to screening.<br>19. Subjects with a history of hemorrhagic and/or non-hemorrhagic stroke.<br>20. Presence or history of peripheral neuropathy.<br>21. History of inflammatory neurological disorders with potential for CNS involvement.<br>22. Screening MRI scan showing structural evidence of alternative pathology not consistent with AD which could cause the subject's symptoms. Evidence of space occupying lesions other than benign meningioma of less than 1 cm diameter, more than two lacunar infarcts or one single infarct larger than 1 cm in diameter or any single area of superficial siderosis or evidence of a prior macro-hemorrhage ≥10 mm. Microbleeds on T2* MRI are allowed up to a maximum of 10, regardless of the location.

	23. MRI examination cannot be done for any reason, including but not limited to metal implants contraindicated for MRI studies and/or severe claustrophobia.<br>24. Significant hearing or visual impairment or other issues judged relevant by the investigator preventing to comply with the protocol and to perform the outcome measures.<br>25. Clinically significant infections or major surgical operation within 3 months prior to screening. Planned surgery anticipated to occur during participation in the study must be reviewed and approved by the medical monitor at screening.<br>26. Any vaccine received within the past 2 weeks before screening, including influenza vaccine.<br>27. Clinically significant arrhythmias or other clinically significant abnormalities on ECG at screening.<br>28. Myocardial infarction within one year prior to baseline, unstable angina pectoris, or significant coronary artery disease.<br>29. History of cancer within the past 5 years other than treated squamous cell carcinoma, basal cell carcinoma and melanoma in situ, or in-situ prostate cancer or in-situ breast cancer which have been fully removed and are considered cured.<br>30. Clinically significant deviations from normal values for hematologic parameters, liver function tests, and other biochemical measures, that are judged to be clinically significant in the opinion of the investigator.<br>31. Pregnancy confirmed by blood test at screening, or subject planning to be pregnant or lactating.<br>32. Receipt of any anticoagulant drug or antiplatelet drug, except aspirin at doses of 100 mg daily or lower (in order to avoid risk of bleeding during scheduled or unscheduled lumbar puncture).<br>33. Receipt of any antipsychotic drugs unless on stable low doses for the treatment of insomnia.<br>34. Donation of blood or blood products within 30 days prior to screening or plans to donate blood while participating in the study.<br>35. Positive Venereal Disease Research Laboratory (VDRL) consistent with active syphilis at screening.<br>36. Positive HIV test at screening.<br>37. Laboratory or clinical evidence of active hepatitis B and/or C at screening.<br>38. Serum creatinine greater than 1.5x upper limit of normal, abnormal thyroid function tests or clinically significant reduction in serum B12 or folate levels (note: all oral doses of thyroid replacement agents, B12 or folate have to be stable for at least 3 months prior to screening).
**Study Vaccines**	**ACI-35.030 vaccine**<br>**JACI-35.054 vaccine**<br>**Placebo**: Phosphate-buffered saline (PBS) solution
**Dosage, duration of treatment**	In Cohort 1, ACI-35.030 and placebo will be administered by the intramuscular route. The dosage of ACI-35.030 will be 300 µg, 900 µg and 1800 µg in sub-cohorts 1.1, 1.2 and 1.3, respectively.<br>In Cohort 2, JACI-35.054 and placebo will be administered by the intramuscular route. The dosage of JACI-35.054 will be 15 µg in sub-cohort 2.1, up to 60 µg in optional sub-cohort 2.2 and up to 150 µg in optional sub-cohort 2.3.<br>In Cohort 3, ACI-35.030 and JACI-35.054 or placebo will be administered by the intramuscular route with the same administration schedule as in cohorts 1 and 2. In each sub-cohort, ACI-35.030 and JACI-35.054 will be administered sequentially to 6 subjects and placebo will be administered at each dosing occasion to 2 subjects.<br>In sub-cohort 3.1, ACI-35.030 and JACI-35.054 at dosages previously evaluated in cohorts 1 and 2 respectively and found to be safe after a study treatment period of at least 10 weeks will be administered. Immunizations with ACI-35.030 will be performed at months 0 (Week 0) and 2 (Week 8), and with JACI-35.054 at month 6 (Week 24) and 12 (Week 48).<br>In optional sub-cohorts 3.2 and 3.3, the dosages of ACI-35.030 and/or of JACI-35.054 and/or the sequence of administration may differ from sub-cohorts 3.1 and 3.2 and will be based on available safety, tolerability and immunogenicity data from previous study interim analyses. The selected

	dosages will have already been evaluated and found safe and well tolerated in the previously available IAs.<br><br>In all cohorts, the vaccine or placebo will be administered 4 times at 8, 16* and 24 week intervals between each dose. ***Due to the Covid-19 pandemic, local travel restrictions in Finland effective as of 18 March 2020, and the need to preserve the safety of study participants, modifications are made to the schedule of immunizations of sub-cohort 1.1 subjects having not received the 3rd injection at month 6 already**: immunizations at month 6 (week 24) will not be performed in 7 of 8 sub-cohort 1.1 subjects for whom the planned Visit 5 does not allow all assessments to be done according to the protocol.<br>Due to the resurgence of the Covid-19 pandemic in multiple participating countries since September 2020, additional adjustments related to the mode of administration of study assessments including study vaccine administration may apply in Finland and other participating countries after the above measures were implemented in Finland.<br>In all cohorts, the treatment duration is anticipated to be 50 weeks (12 months) and followed by a 24-week (6 months) safety follow-up period. The overall subject participation will be up to around 80 weeks from first screening assessment to last safety follow-up visit. If the follow-up period is extended in a given sub-cohort by another 24 weeks (6 months), the overall duration will be up to around 104 weeks.

TABLE OF CONTENTS

Signature Page **2**

Study Synopsis **5**

Table of Contents **14**

List of Abbreviations and Definition of Terms **17**

Study Contacts **19**

1. Introduction **20**

1.1 Background *20*

1.1.1 Alzheimer's Disease 20

1.1.2 Introduction to ACI-35.030 21

1.1.3 Introduction to JACI-35.054 22

1.1.4 Heterologous immunization; priming with ACI-35.030 vaccine and boosting with JACI-35.054 vaccine 23

1.1.5 Conclusion 24

1.2 Study Rationale *24*

1.3 Dose Rationale *25*

1.4 Study Endpoint Rationale *26*

1.5 Risks and Benefits for Subjects *26*

1.5.1 Risk of Study Intervention 26

1.5.2 Risks of Participation during Covid-19 Pandemic 27

2. Study Objectives and Endpoints **29**

2.1 Study Objectives *29*

2.1.1 Primary Objectives 29

2.1.2 Secondary Objectives 29

2.1.3 Exploratory Objectives 29

2.2 Study Endpoints *29*

2.2.1 Primary Endpoints 29

2.2.2 Secondary Endpoints 30

2.2.3 Exploratory Endpoints 30

3. Investigational Plan **30**

3.1 Overall Study Design and Plan *30*

3.1.1 Cohort 1 with ACI-35.030 30

3.1.2 Cohort 2 with JACI-35.054 31

3.1.3 Cohort 3 with ACI-35.030 and JACI-035.054 in Sequential Administration 31

3.1.4 Sub-cohort expansion 32

3.1.5 Safety follow-up 32

3.1.6 Post-injection period extension (optional) 33

3.1.7 Interim analyses (IA) ... 33
3.1.8 Schematic overview of cohorts design ... 35

3.2 Study Duration ... 36
3.2.1 Screening Period ... 36
3.2.2 Treatment Period ... 36
3.2.3 Follow-up Period ... 36
3.2.4 Post-Injection Period Extension (optional) ... 36
3.2.5 Overall Study Duration ... 36
3.2.6 End of Study Definition ... 36

3.3 Selection of Study Population ... 37
3.3.1 Inclusion Criteria ... 37
3.3.2 Exclusion Criteria ... 37
3.3.3 Removal of Subjects from Therapy or Assessment ... 39

3.4 Treatments ... 40
3.4.1 Details of Study Treatments ... 40
3.4.2 Dosage Schedule ... 42
3.4.3 Treatment Assignment ... 43
3.4.4 Drug Packaging and Blinding ... 43
3.4.5 Drug Inventory and Accountability ... 44
3.4.6 Treatment Compliance ... 44
3.4.7 Prior and Concomitant Illness and Treatments ... 44

3.5 Assessments ... 45
3.5.1 Schedule of Assessments ... 45
3.5.2 Study Procedures ... 51
3.5.3 Assessments for Premature Termination ... 60
3.5.4 Safety/Tolerability Assessments ... 60
3.5.5 Primary and Secondary Immune Response Assessments ... 64
3.5.6 Exploratory assessments ... 64

4. Safety Procedures ... 66

4.1 Definitions and Criteria ... 66
4.1.1 Adverse Events ... 66
4.1.2 Serious Adverse Event ... 66
4.1.3 Unexpected Adverse Drug Reactions ... 67
4.1.4 Abnormal Laboratory Values ... 67
4.1.5 Assessing Intensity and Relationship ... 67
4.1.6 Monitoring and Management of Adverse Events Including Specific Risks ... 68

4.2 Reporting Procedures and Requirements ... 68
4.2.1 Adverse Events ... 68
4.2.2 Serious Adverse Events ... 69
4.2.3 Contraceptive measures and procedures for documenting pregnancy during the study ... 70
4.2.4 Stopping Rules ... 71

5. Data Management and Statistical Analysis ... 72

5.1 Data Management Considerations ... 72

5.2 Electronic CRFs ... 72

5.3 Statistical Considerations ... 73
5.3.1 Sample Size Justification ... 73
5.3.2 Analysis Population ... 73
5.3.3 Protocol Deviations ... 74
5.3.4 Demographic and Baseline Characteristics ... 74
5.3.5 Safety Analyses ... 74
5.3.6 Immune Response Analyses ... 76
5.3.7 Exploratory Analyses ... 76
5.3.8 Interim Analysis ... 78

6. Study Management ... 81

6.1 Ethics and Consent ... 81
6.1.1 Regulations and Guidelines ... 81
6.1.2 Institutional Review Board/Independent Ethics Committees and regulatory authorities ... 81
6.1.3 Informed Consent ... 81
6.1.4 Confidentiality and privacy ... 82
6.1.5 Future Use of Stored Samples ... 82

6.2 Indemnification ... 82

6.3 Discontinuation of the Study by the Sponsor ... 83

6.4 Study Monitoring and Auditing ... 83

6.5 Retention of Records ... 84

6.6 Use of Study Findings ... 84

6.7 Publications ... 84

7. References ... 85

8. Appendices ... 87

Appendix 1 NIA/AA Criteria for probable AD Dementia (McKhann et al., 2011) ... 87

LIST OF ABBREVIATIONS AND DEFINITION OF TERMS

**Aβ**	Amyloid Beta
**AD**	Alzheimer's Disease
**AE**	Adverse Event
**ANA**	Anti-nuclear Antibodies
**Anti-dsDNA**	Anti-double stranded DNA Antibodies
**ApoE**	Apolipoprotein E
**aPTT**	activated Partial Thromboplastin Time
**CDR-SB**	Clinical Dementia Rating scale Sum of Boxes
**CHMP**	Committee for Medicinal Products for Human Use
**cm (unit)**	Centimeter
**CNS**	Central Nervous System
**Covid-19**	Coronavirus disease of 2019
**CRF**	Case Report Form
**CRO**	Contract Research Organization
**CRP**	C-Reactive Protein
**CSF**	Cerebrospinal Fluid
**C-SSRS**	Columbia-Suicide Severity Rating Scale
**DSMB**	Data and Safety Monitoring Board
**DSUR**	Development Safety Update Report
**EC**	Ethics Committee
**ECG**	Electrocardiogram
**EDC**	Electronic Data Capture
**ELISA**	Enzyme-Linked Immunosorbent Assay
**ELISPOT**	Enzyme-Linked Immunospot
**FSH**	Follicle Stimulating Hormone
**GCP**	Good Clinical Practice
**HIV**	Human Immunodeficiency Virus
**IB**	Investigator's Brochure
**ICH**	International Conference on Harmonisation
**IFN-γ**	Interferon-gamma
**IgG**	Immunoglobulin G
**IL-4**	Interleukin-4
**IMP**	Investigational Medicinal Product
**INR**	International Normalized Ratio
**IRB**	Institutional Review Board
**IRT**	Interactive Response Technology
**ITT**	Intention To Treat

**IUD**	Intrauterine Device
**kg (unit)**	Kilogram
**LSLV**	Last Subject Last Visit
**µg (unit)**	Microgram
**MacCAT-CR**	MacArthur Competence Assessment Tool for Clinical Research
**MCI**	Mild Cognitive Impairment
**MMSE**	Mini Mental State Examination
**MPLA**	Monophosphoryl Lipid A
**MRI**	Magnetic Resonance Imaging
**NIA-AA**	National Institute on Aging-Alzheimer's Association
**NPI**	Neuropsychiatric Inventory Scale
**PBMC**	Peripheral Blood Mononuclear Cell
**PBS**	Phosphate Buffered Saline
**PET**	Positron Emission Tomography
**PI**	Principal Investigator
**PP**	Per Protocol
[redacted]	[redacted]
**pTau**	Phospho-Tau
**RBANS**	Repeatable Battery for the Assessment of Neuropsychological Status
**SAE**	Serious Adverse Event
**SAP**	Statistical Analysis Plan
**SDV**	Source Data Verification
**SUSAR**	Suspected Unexpected Serious Adverse Reaction
**T3**	Triiodothyronine
**T4**	Thyroxine
**TEAE**	Treatment Emergent Adverse Event
**TNF-α**	Tumor Necrosis Factor alpha
**TSH**	Thyroid-Stimulating Hormone
**VDRL**	Venereal Disease Research Laboratory
**WOCBP**	Woman Of Child Bearing Potential

STUDY CONTACTS

**Sponsor**<br>AC Immune SA<br>EPFL Innovation Park - Building B<br>CH-1015 Lausanne, Switzerland<br>[redacted]	**Central Reading (MRI and ECG)**<br>[redacted]
**Study Coordination (Monitoring activities, Data Management and Statistics, Pharmacovigilance, etc.)**<br>[redacted]<br><br>**Statistics analysis of data:**<br>[redacted]	**Central Laboratory (Safety and other testing)**<br>[redacted]
**IRT**<br>[redacted]	**Central Laboratory (Primary and Secondary Immunogenicity endpoints)**<br>[redacted]
**Drug Distribution**<br>[redacted]	**Emergency contact details - Safety Officer**<br>[redacted]

1. INTRODUCTION

1.1 Background

1.1.1 Alzheimer’s Disease

Alzheimer’s Disease (AD) is a progressive degenerative disorder of insidious onset characterized by loss of memory and other cognitive function, loss of ability to perform daily living activities and behavioral disturbances. In 2018, an estimated 5.7 million Americans of all ages have been living with Alzheimer's dementia. Worldwide, the number was believed to be close to 50 million people in 2017 (*Alzheimer’s Disease International*).

Currently available treatments are primarily symptomatic in their effects. Hence, there is a need for new therapeutic approaches targeting the pathology of the disease and thereby slowing its progression.

The key pathological hallmarks of the disease are:

- Senile plaques, consisting of abnormal aggregates of the beta-amyloid (Aβ) peptides outside of neurons,
- Neurofibrillary tangles, consisting of abnormally intraneuronal aggregated forms of Tau protein.

Tau protein is a normal component of neurons, and is known as a microtubule-associated protein (MAP) as it interacts with tubulin to stabilize and promote assembly of microtubules in neuronal axons. In AD, Tau protein becomes hyperphosphorylated due to an imbalance in the activity of kinase and phosphatase enzymes. Abnormal phosphorylated Tau aggregates readily into insoluble oligomers which are neurotoxic and contribute to neurodegeneration (Goedert *et al*, 1991). The oligomers progress to tangles of so-called paired helical filaments (Alonso *et al.*, 2001). The degree of neurofibrillary tangle pathology has been consistently shown to be correlated to the degree of dementia in AD subjects (Bierer *et al*, 1995; Braak and Braak, 1991; Delacourte, 2001).

Understanding of the biology of Tau in normal brain and in AD is evolving rapidly. There is new evidence that Tau is released from neurons under normal conditions and taken up by adjacent cells, suggesting that it might have a role in cell signaling (Pooler *et al*, 2013). Furthermore there is evidence that hyperphosphorylated Tau can be released from neurons and taken up by adjacent neurons where it seeds further aggregation and spreading of Tau pathology (Iba *et al*, 2013). The presence of abnormal Tau outside of cells suggests that cell penetration may not be a prerequisite for therapeutic intervention to interfere with the propagation of Tau pathology. Recent data from Positron Emission Tomography (PET) studies suggest that the interaction between beta-amyloid and Tau may be particularly important in the progression of the disease, with Tau spreading occurring more rapidly when amyloid and Tau pathologies coexist in temporal brain areas (Iaccarino *et al*, 2017).

To date, few studies have been conducted with agents specifically targeting Tau pathology. Whereas the formation of senile plaques appears to occur very early in AD, tangle formation, while also present early in the condition, continues to progress as the disease evolves. This suggests that a therapy addressing Tau pathology might be effective both early and later in the disease. Indeed, Axon Neurosciences is developing the active vaccine, AADvac1, directed against pathological Tau proteins, as it has shown to reduce the amount of Tau pathology in the brains of animal models in Alzheimer’s disease, almost completely eliminating a range of AD Tau phospho-species. A phase 1 (Novak *et al*, 2017) and phase 2 (Novak et al, 2021) trials evaluating safety and immunogenicity of AADvac1 in subjects with mild to moderate AD were completed.

Development of therapies aiming at modifying Tau pathology is therefore a rational approach in the continuing search for effective treatments for this condition.

1.1.2 Introduction to ACI-35.030

ACI-35 is a liposomal vaccine consisting of a tetra-palmitoylated pTau T3 peptide (393-408 amino acid (aa) of the longest human Tau isoform (441aa) with phosphorylation on S396 and S404) and monophosphoryl lipid A (MPLA) as adjuvant. Both components are embedded into the bilayer of liposomes. This first generation vaccine has been investigated in a phase 1 clinical trial (ACI-35-1201 study).

ACI-35.030 is a modified version of the ACI-35 vaccine and therefore, referred to as a “second generation vaccine”. A clinical trial assessing ACI-35 demonstrated a favourable safety and tolerability profile when using the antigen (T3 peptide) and the liposomal vehicle composition yet a limited induction of an anti-pTau IgG response at all doses tested. In addition, a boosting effect was not observed with the subsequent injections. Thus, ACI-35.030 is modified to maintain the original antigen (i.e. T3 peptide) and liposomal vehicle composition as ACI-35, while incorporating two additional adjuvants (i.e. 3D-6A-MPLA and CpG7909-cholesterol) and one universal T-cell epitope (i.e. T50 peptide) into the vaccine to potentially improve the anti-pTau IgG response.

1.1.2.1 Pharmacology Studies

One pharmacology study in mice and three studies in Rhesus monkeys showed that the immunization with ACI-35.030 induced:

- a strong and consistent anti-pTau peptide IgG response in C57BL/6 wild type mice
- a strong and consistent anti-pTau peptide IgG response in Rhesus monkeys
- a strong and consistent anti-PHF (paired helical filaments from human Alzheimer’s disease subject) IgG response in Rhesus monkeys

Further details are available in the investigator’s brochure (IB).

1.1.2.2 Toxicology and Safety Studies

One repeated dose GLP toxicology study has been conducted with ACI-35.030 in Rhesus monkey. This study determined that:

- ACI-35.030 immunization was locally well-tolerated and was not associated with organ toxicity.

Additional non-clinical information can be found in the latest version of the IB.

1.1.2.3 Clinical Data

<u>ACI-35.030</u>

At the time of the version of this protocol, 26 subjects have been exposed to ACI-35.030, i.e. the second generation vaccine, or placebo in the current study, in sub-cohorts 1.1, 1.2 and 1.3.

<u>ACI-35</u>

Although ACI-35 differs from the currently tested vaccine, ACI-35.030, in its composition and adjuvants, the safety data generated in the Phase Ib study are included below for information.

The safety, tolerability and immunogenicity of ACI-35 vaccine was evaluated in a clinical phase Ib study conducted in subjects with mild to moderate AD in UK and Finland (ACI-35-1201: A Phase Ib Multicenter, Double-Blind, Randomized, Placebo-Controlled Study of the Safety, Tolerability and Immunogenicity of ACI-35 in Subjects with Mild to Moderate Alzheimer's Disease). The clinical trial was completed on 15 June 2017.

Five cohorts were studied. Each cohort consisted of a different dose (300 µg, 900 µg or 1800 µg) and/or dosing regimen spread over 24 weeks of varying intervals (2, 3 or 5 dose administrations), followed by a final injection that occurred at Week 48 (cohorts 2-5) or between Week 48 and Week 76 (cohort 1).

Twenty-four subjects were randomized and received at least 1 dose of study vaccine. Twenty-two completed the study. Two subjects discontinued the study due to a significant unrelated medical condition which seriously hampered or made the completion of the study impossible.

Overall, ACI-35 was well tolerated in the participating population at all doses tested. Injection site erythema and injection site reactions and fatigue were the most common study vaccine-related Treatment Emergent Adverse Events (TEAE). There was no evidence clinically or from laboratory or radiology findings to suggest the development of CNS inflammation. In general, no pattern of AEs emerged, except injection site reactions on active treatment, which occurred in all subjects at the highest doses tested but were generally mild and self-limiting and did not lead to subject withdrawal.

Treatment with ACI-35 induced IgG and IgM antibodies recognizing both pTau and Tau in most subjects after the first injection. The antibody response in serum was higher for pTau than for Tau confirming the selectivity for an anti-pTau response observed in animal studies. Most subjects receiving the vaccine exhibited a measurable IgM response after the primary and subsequent immunizations. Many subjects also showed a measurable IgG response to the pTau peptide incorporated in the vaccine, but this was variable between individuals and generally did not increase with repeated administrations.

Biomarker analysis in serum or plasma showed inconsistent changes over the course of the study. Furthermore, the ability to draw any conclusion from the biomarker analyses in the samples from the CSF was hampered by the limited number of data obtained. Toll-Like Receptor 4 (TLR4) expression levels in the blood remained stable over the course of the study.

Clinical and cognitive endpoints showed the expected variability over time without evidence for a detrimental effect of the vaccine. Larger studies will be needed to explore effects of the vaccine on these endpoints.

1.1.3 Introduction to JACI-35.054

JACI-35.054 is a vaccine consisting of a conjugate called CRM197-pTau with Aluminum Hydroxide and CpG7909 as adjuvants. CRM197-pTau is a conjugate of a carrier protein called CRM197 and peptide JNJ-66649687. JNJ-6649687 is a chemically synthesized peptide that shares its core 16 amino acid sequence with the T3 peptide described in the ACI-35/ACI-35.030 study vaccines. CRM197 is a well-defined recombinant protein that is a commercially available version of a non-toxic mutant of diphtheria toxin (DT) A chain and that has proven to be a safe carrier protein in commercial prophylactic vaccines and clinical trials for a plethora of different vaccine candidates.

JACI-35.054 is referred to as a "second generation vaccine" because it maintains the original antigen (i.e. T3 peptide) of the ACI-35 vaccine. Pharmacology studies in Rhesus monkeys have indicated that this vaccine induces a slightly different epitope profile of pTau-specific antibodies in the same antigenic sequence as compared to ACI-35.030. This makes JACI-35.054 a good candidate to potentially be used together with ACI-35.030 in order to be able to increase the

quality of immune response. Thus, this second generation vaccine will be tested alone in cohort 2 and in a selected sequence of injection with ACI-35.030 in cohort 3. The goal will be to identify the optimal dosages and regimens for subsequent clinical development.

1.1.3.1 Pharmacology Studies

Two pharmacology studies in Rhesus monkeys showed that the immunization with JACI-35.054 alone or in combination with ACI-35.030 induced:

- a strong and consistent anti-pTau peptide IgG response
- a strong and consistent anti-PHF (paired helical filaments from human Alzheimer's disease subjects) IgG response

Two pharmacology studies in wild type mice showed that the immunization with JACI-35.054 alone induced:

- a strong and consistent anti-pTau peptide IgG response
- optimal anti-pTau peptide IgG response when JACI-35.054 is administered with both Aluminum Hydroxide and CpG7909 adjuvants

Further details are available in the investigator's brochure (IB).

1.1.3.2 Toxicology and Safety Studies

GLP toxicology studies have been conducted with JACI-35.054 in mice and in Rhesus monkey. These studies determined that:

- JACI-35.054 immunization was locally well-tolerated and was not associated with systemic toxicity.

Additional non-clinical information can be found in the latest version of the IB.

1.1.3.3 Clinical Data

At the time of the version of this protocol, 16 subjects have been exposed to JACI-35.054 or placebo in the current study, in sub-cohorts 2.1 and 2.2.

1.1.4 Heterologous immunization; priming with ACI-35.030 vaccine and boosting with JACI-35.054 vaccine

It has been shown over the past decades in non-clinical and clinical studies that a heterologous prime-boost vaccination approach (delivering the same antigen with different vaccine backbones) can be advantageous over a homologous prime-boost regimen (immunizing several times with the same vaccine) (Lu *et al*, 2009; Jalah *et al*, 2014; Khurana *et al*, 2014; Shukarev *et al*, 2017). Heterologous prime-boost vaccination will avoid the re-exposure of the immune system with the "vaccine backbone" and is therefore meant to focus the immune response against the desired antigen(s). This can have two main consequences: (I) avoid a more rapid elimination of the vaccine during a booster vaccination due to pre-existing immunity and memory against several components of the vaccine; (II) induce a more directed immune response against the common antigen (in this specific case the pTau peptide) as only this antigen will be recognized by already primed immune cells. Thus, a heterologous prime-boost approach using the ACI-35.030 liposomal formulation in combination with the JACI-35.054 conjugate vaccine which could improve the immune response elicited towards the pTau peptide will be evaluated in Cohort 3 as described below.

1.1.4.1 *Pharmacology Studies*

Pharmacology studies in Rhesus monkeys showed that priming with ACI-35.030 and boosting with JACI-35.054 induced:

- a strong and consistent anti-pTau peptide IgG response
- a strong and consistent anti-PHF (paired helical filaments from human Alzheimer's disease subjects) IgG response
- Antibodies that bind to the N-terminal and C-terminal regions of the pTau peptide, thus increasing the epitope coverage of the pTau peptide as compared to the ACI-35.030 or JACI-35.054 alone.
- Decreased anti-T50 or anti-CRM197 IgG antibody titers as compared to the homologous immunization regimen.

1.1.4.2 *Toxicology and Safety Studies*

A heterologous combination repeated dose toxicology GLP study was carried out with ACI-35.030 and JACI-35.054 in Rhesus monkey. Rhesus monkeys were treated first with a single dose of ACI-35.030 and subsequently with two doses of JACI-35.054. The study demonstrated:

- ACI-35.030 and JACI-35.054 were both locally and systemically well-tolerated and were not associated with organ toxicity

Additional non-clinical information can be found in the latest version of the IB.

1.1.5 Conclusion

The preceding clinical study ACI-35-1201 demonstrated that ACI-35, i.e. the first generation vaccine, was safe and well tolerated at all doses and treatment regimens tested. However, the IgG response induced in individuals after the first injection did not result in a boosting effect upon subsequent injections and thus, considered of an insufficient magnitude to confer a sustainable clinical benefit. To address this situation, second generation vaccines were developed. ACI-35.030 utilizes properties of ACI-35 (i.e. the liposome and antigen composition). JACI-35.054 incorporates the same pTau peptide antigen, but presents the pTau to the immune system in a different way. In JACI-35.054 the pTau peptide is attached to a large carrier protein (CRM197) instead of being inserted in a liposome. The carrier protein should help the immune system to respond more efficiently to the pTau peptide. At the time of the version of this protocol, ACI-35.030 and JACI-35.054 have been well tolerated in humans in this ongoing clinical study. A total of four serious adverse events in four different subjects have been reported to date (at the time of the version of this protocol) in cohort 1, all considered unlikely related to the study medication.

The strong rationale for testing compounds which have the potential to modify Tau pathology in AD, in conjunction with favorable preclinical safety and efficacy profile, make ACI-35.030 and JACI-35.054 suitable candidates for testing in AD subjects.

1.2 Study Rationale

The preceding clinical trial ACI-35-1201 showed that the original vaccine ACI-35 was safe and well tolerated at all doses and treatment regimens tested. However, the antibody response observed after the first injection in most subjects was not boosted with the subsequent injections thus making it unlikely to confer a potential clinical benefit. To overcome this problem, new "second generation" vaccines were developed, ACI-35.030 and JACI-35.054.

Each second generation vaccine will be tested alone with potential dose escalation and together in a selected sequence of injections (heterologous prime-boost vaccination).

It has been shown that a heterologous prime-boost vaccination strategy can confer superior protection as compared to a homologous prime-boost regimen. However, it has also become clear that the order of administration is crucial to obtain optimal immune responses. This has been extensively observed in the context of infectious diseases, where a DNA vaccine prime followed by a protein vaccine boost was shown to induce higher antibody titers and enhance T cell responses as compared to a protein vaccine prime followed by a DNA vaccine boost (Sin *et al*, 1999; Lu *et al*, 2009). In order to choose the best possible combination between the liposomal formulation ACI-35.030 and the conjugate vaccine JACI-35.054, different heterologous prime-boost regimens have been tested in rhesus monkeys. The resulting data show that a sequence in which priming twice with ACI-35.030 followed by boosting twice with JACI-035.054 induces higher antibody titers against the pTau peptide at the end of the treatment as compared to when priming twice with JACI-35.054 and boosting twice with ACI-35.030. Moreover, a superior capacity to boost the anti-pTau peptide IgG response after each consecutive immunization was observed with the first regimen. In addition, a similar trend was also observed for the antibody response directed against PHF (paired helical filaments isolated from the brain of an AD patient), with higher anti-PHF IgG titers at the end of the treatment when priming with ACI-35.030 and boosting with JACI-35.054.

Based on the above non-clinical data, the selected immunization schedule for the current study in patients is one in which subjects are primed twice with ACI-35.030 followed by two booster immunizations with JACI-35.054.

The results obtained from the use of single second generation vaccines or from heterologous prime-boost vaccination will be compared to select the best strategy for further clinical development.

1.3 Dose Rationale

ACI-35.030 will be tested in up to 3 dose levels in cohort 1, i.e. 300 µg/dose, 900 µg/dose and 1800 µg/dose. The initial dose of 300 µg has been used to evaluate the safety of ACI-35.030 before higher doses are administered to subjects. Both higher doses were determined based on the results of the preceding clinical study and/or on results of pre-clinical studies.

The best performing dose cohort and administration pattern in terms of immunogenicity, safety and tolerability will optionally be expanded to confirm the safety, tolerability and immunogenicity of ACI-35.030.

JACI-35.054 will be tested in cohort 2 at 15 µg/dose and optionally in 2 subsequent cohorts with dosages up to 60 and up to 150 µg/dose (at the time of the version of this protocol, one sub-cohort with a dosage of 60 µg/dose has been initiated following the dose escalation after the 15 µg/dose sub-cohort). The initial dosage of 15 µg will be used to evaluate the safety and immunogenicity of JACI-35.054 before a potential higher dose is administered to subjects. The upper limit of 150 µg/dose is based on non-clinical toxicology data.

ACI-35.030 and JACI-35.054 will be tested in a selected heterologous vaccination regimen in cohort 3. In sub-cohort 3.1, ACI-35.030 and JACI-35.054 will be tested at dosages previously evaluated in cohorts 1 and 2 respectively and found to be safe after a study treatment period of at least 10 weeks. The two optional subsequent sub-cohorts of cohort 3 may include a different sequence of administration than in the previous sub-cohort(s) of cohort 3 and/or different dosages of ACI-35.030 and/or JACI-35.054 based on available safety, tolerability and immunogenicity data from previous sub-cohort interim analyses.

In this study, the administration route will be intramuscular (instead of subcutaneous, as was done in the previous ACI-35-1201 study) in order to improve potentially both local safety at the injection site and immunogenicity. Indeed, better local tolerability and doubling of the area under

the curve for antibody responses to the Aβ vaccine CAD 106 has been reported when switching from the subcutaneous to the intramuscular route of administration (Farlow *et al*, 2015). For vaccines against infectious diseases, intramuscular administration has also been associated with higher immune responses – notably in the case of Hepatitis B vaccine (Wahl *et al*, 1987). The incidence of injection related reactions is often reduced by using this route of administration despite similar or enhanced immune responses to vaccination (Cook, 2008, Diez-Domingo *et al*, 2015).

1.4 Study Endpoint Rationale

The study endpoints were determined from the results of the previous Phase Ib trial and knowledge associated with Alzheimer's Disease.

Primary endpoints will evaluate:

- The safety and tolerability of the study vaccines
- The immunogenicity of the study vaccines (induction of anti-pTau IgG in serum)

These endpoints will form the basis for selection of the vaccine with the optimal profile in terms of safety and immune response.

The secondary endpoints will further evaluate the immune response including:

- The induction of anti-Tau IgG and anti-pTau and anti-Tau IgM in serum as a quantitative response to vaccination.
- The avidity of antibodies elicited by immunization in order to confirm that elicited antibodies have the right profile (qualitative immune response and not only quantitative).

These endpoints will provide further support in selection of the optimal vaccine for further development.

The exploratory endpoints will explore:

- The effect of the vaccines on markers of unwanted inflammation (Induction of tau-specific cytotoxic T cells, inflammatory cytokines)
- The ability of the vaccines to induce antibodies against (p)Tau and against other components of the vaccine (e.g. T50, CRM197, adjuvants)
- The functional capacity of vaccine-induced antibodies as measured by e.g., in vitro Tau uptake assays
- The effect of the vaccines on putative AD biomarker levels in blood and/or CSF (total Tau and pTau) as preliminary evidence of epitope engagement
- The effect of the vaccines on cognitive and clinical endpoints, to assess any unwanted negative effects on these endpoints and obtain preliminary information concerning any positive effects on such endpoints, taking into account that the study is not powered to detect efficacy on such measures.

1.5 Risks and Benefits for Subjects

1.5.1 Risk of Study Intervention

ACI-35.030 and JACI-35.054 include small peptides as antigen encapsulated in liposomes or attached to a recombinant protein, respectively. ACI-35.030 and JACI-35.054 have not shown any systemic adverse reactions in animals. Allergic or autoimmune reactions could, however, theoretically occur. Likewise, a risk of anaphylactic reaction cannot be excluded. Injection site reactions may occur, though to date have been mild for ACI-35.030 and JACI-35.054.

As for other vaccines aimed to act on pathology in the central nervous system, there is a theoretical risk of inducing central nervous system inflammation/meningoencephalitis. Based on the available non-clinical data, the risk of such occurrences is considered to be low. Nevertheless, the possibility of T cell activation leading to such unwanted CNS inflammation cannot be completely excluded.

In addition, although antibodies produced after immunization show specificity for phospho-tau over normal tau, some effect on normal tau might occur. There is no evidence from non-clinical safety studies that the vaccines are associated with abnormalities of neuronal function. Nevertheless, investigators must remain vigilant by carefully evaluating any adverse events reported by the subjects and caregivers. Investigators should also carefully monitor any clinical abnormalities during physical and neurological examinations, especially any possible symptoms or signs of CNS inflammation or neuronal dysfunction and strictly follow and evaluate the safety assessments including MRI scans as per the study protocol. The management of suspected meningoencephalitis cases is described in section 4.1.6.

In the preceding Phase Ib study, the first generation vaccine ACI-35 was well tolerated at all doses tested. Injection site erythema and injection site reactions and fatigue were the most common study vaccine-related AEs. There was no evidence clinically or from laboratory or radiology findings to suggest the development of CNS inflammation. The dose-dependent injection site reactions observed on active treatment were generally mild and self-limiting and did not lead to subject withdrawal.

ACI-35.030 and JACI-35.054 differ from ACI-35. There is thus no guarantee that the good safety profile of the first generation vaccine will be similar for the second generation vaccines. It is also not possible to predict that both second generation vaccines will have the same safety profile based on pre-clinical and clinical results obtained to date. Investigators must therefore remain vigilant and carefully monitor safety according to the study protocol in order to detect early any adverse events.

New safety information arising from the current study which may modify the benefit-risk balance will be provided on an ongoing basis to principal investigators (PIs), ECs and regulatory authorities in the form of SUSAR reports, Development Safety Update Report (DSUR) and/or separate communications or updates to the Investigator's Brochure.

More generally, it is not possible to guarantee that the participating subjects will benefit from being in this study.

Two out of 8 participants will be receiving placebo administrations in each study sub-cohort.

1.5.2 Risks of Participation during Covid-19 Pandemic

Risks of acquiring infection with Covid-19 must be mitigated as much as possible during the ongoing pandemic. Safety of travelling to and attending the site must be evaluated taking into account any local travel restrictions and regulations. Subjects are to be advised to use all possible measures to reduce the risk of infection including social distancing, use of private transportation to the study site, frequent hand washing and use of face masks as appropriate.

Due to the Covid-19 pandemic, health authorities, PIs or the sponsor may consider it is not appropriate for subjects to attend on-site visits. The situation will be monitored on an ongoing basis and the following decisions may be made, before each subject visit, depending on the situation at the local level:

- If the planned screening assessments cannot be completed in safe conditions, all screening assessments will be postponed.

- If a subject is deemed eligible after completing the screening assessments but it is anticipated that randomization (V1) and/or any subsequent visit cannot be completed for logistical or safety reasons, the randomization visit and the related first administration of study vaccine will be postponed.

- Randomization and the related first administration of study vaccine also may be postponed per PI judgment or/and local measures against Covid-19 infection.

- If a subject is already randomized and has already received one dose of study vaccine, it will be assessed whether the subsequent visits can take place safely on site:

  - If yes, the visit and associated on site assessments will take place as planned.
  - If no, on site visits will be replaced by either phone visits or visits at a subject's home including the administration of the study vaccine if permitted according to local regulations (see section 3.5.1.1).

Should home visits be performed, subjects and site staff will be advised to use all possible measures to reduce the risk of infection as per local regulations.

2. STUDY OBJECTIVES AND ENDPOINTS

The overall study objective is to assess the safety, tolerability and immunogenicity of two study vaccines and the best performing administration pattern in subjects with early Alzheimer's Disease (AD), i.e. Mild AD or MCI as diagnosed by the criteria of the National Institute on Aging – Alzheimer's Association (NIA-AA) with a score at initial screening of 22 or above on the Mini-Mental State Examination scale (MMSE).

2.1 Study Objectives

2.1.1 Primary Objectives

- To assess the safety and tolerability of study vaccines.
- To assess the immunogenicity of study vaccines (induction of IgG titers against pTau in serum).

2.1.2 Secondary Objectives

- To further assess the immunogenicity of study vaccines (induction of IgG titers against Tau and of IgM titers against pTau and Tau in serum)
- To assess the avidity of antibodies elicited by immunization

2.1.3 Exploratory Objectives

- To explore the effect of study vaccines on putative biomarkers of the progression of AD, i.e. blood and/or CSF concentrations of total Tau and pTau proteins, Tau fragments and other related biomarkers
- To explore the effect of study vaccines on the activation of T-cells in blood
- To explore the activity of study vaccines on blood inflammatory cytokines (e.g., IL-1B, IL-2, IL-6, IL-8, IL-10, IFN- γ, and TNF- α)
- To further explore the effect of study vaccines on the immune response (e.g. antibodies against vaccine components, functional capacity of vaccine-induced antibodies)
- To explore the effect of study vaccines on behavior, cognitive (including, in the Netherlands, the maintenance of the decisional capacity) and functional performance

2.2 Study Endpoints

2.2.1 Primary Endpoints

Safety and tolerability: Adverse events, immediate and delayed reactogenicity (e.g. anaphylaxis, local and systemic reactogenicity, including immune-complex disease); suicidal ideation (C-SSRS); behavior (NPI); cognitive and functional assessments (RBANS, CDR-SB) to assess safety; vital signs; MRI imaging; electrocardiogram; routine hematology and biochemistry evaluation in blood and urine; evaluation of autoimmune antibodies including anti-dsDNA antibodies in blood; inflammatory markers in blood and CSF.

Immune response (i.e. immunogenicity): Anti-pTau IgG titers in serum (e.g. geometric mean, change from baseline, responder rate, peak and area under the curve).

2.2.2 Secondary Endpoints

Immune response (i.e. immunogenicity): Anti-Tau IgG, anti-pTau and anti-Tau IgM titers in serum (e.g. geometric mean, change from baseline, responder rate, peak and area under the curve), determination of the IgG response profile by avidity testing.

2.2.3 Exploratory Endpoints

Change from baseline of putative AD biomarker titers in blood and/or CSF (e.g. total Tau, pTau, Tau fragments and other related biomarkers), change from baseline in T-cell activation levels as measured in blood, change from baseline of inflammatory cytokine titers in blood, change from baseline in antibody titers in blood, change from baseline in behavior (NPI), cognitive (including the proportion of subjects maintaining their decisional capacity during the study using the MacCAT-CR interview in the Netherlands) and functional performance (RBANS, CDR-SB) scores.

3. INVESTIGATIONAL PLAN

3.1 Overall Study Design and Plan

This protocol is a master protocol aimed at investigating two different Tau targeted vaccines.

This study is a multicenter prospective placebo-controlled, double-blind and randomized study to assess treatment with Tau targeted vaccines versus placebo over 50 weeks (i.e. 12 months) in up to 216 subjects with early Alzheimer's Disease. Immunizations will be performed at months 0 (Week 0), 2 (Week 8), 6 (Week 24)* and 12 (Week 48). Based on the safety and immunogenicity results the protocol may be amended to test additional regimens. The subjects will be enrolled in up to 3 Cohorts, each with up to 72 subjects, the actual number depending on the number of sub-cohorts studied and the number of sub-cohorts in which expansion is performed. The Cohorts and their respective sub-cohorts are described in detail below.

***Due to the Covid-19 pandemic, local travel restrictions in Finland effective as of 18 March 2020, and the need to preserve the safety of study participants, modifications are made to the schedule of immunizations of sub-cohort 1.1 subjects having not received the 3rd injection at month 6 already:** immunizations at month 6 (week 24) will not be performed in 7 of 8 sub-cohort 1.1 subjects for whom the planned Visit 5 does not allow all assessments to be done according to the protocol. In addition, Visits 5 (week 24), 6 (week 26) and 7 (week 36) are replaced by remote visits in case these fall during the at-risk period (refer to sections 3.5.2.10, 3.5.2.11, 3.5.2.12 and 3.5.2.14).

Due to the resurgence of the Covid-19 pandemic in multiple participating countries since September 2020, additional adjustments related to the mode of administration of study assessments including study vaccine administration may apply in Finland and other participating countries after the above measures were implemented in Finland.

3.1.1 Cohort 1 with ACI-35.030

For all cohort 1 subjects, the treatment period will last 50 weeks with the treatment/placebo being administered 4 times at respectively 8, 16 and 24-week intervals between each dose. The treatment period will be followed by a 24-week safety follow-up period starting 2 weeks after the last administration until week 74. Subjects who for any reasons have received less than 4 administrations will be followed at least for the same duration after their last administration.

Up to 3 dose levels of ACI-35.030 administered by the intramuscular route will be tested in 3 sub-cohorts.

Sub-cohort 1.1 (8 subjects):

ACI-35.030 at 300 µg/dose will be administered to 6 subjects and the placebo will be administered to 2 subjects. The safety and tolerability data after all subjects have received the second injection in sub-cohort 1.1 will permit dose escalation after review by the DSMB.

Sub-cohort 1.2 (8 subjects):

ACI-35.030 at 900 µg/dose will be administered to 6 subjects and the placebo will be administered to 2 subjects. The safety and tolerability data after all subjects have received the second injection in sub-cohort 1.2 will permit dose escalation after review by the DSMB.

Sub-cohort 1.3 (8 subjects):

ACI-35.030 at 1800 µg/dose will be administered to 6 subjects and the placebo will be administered to 2 subjects.

For further information on the current study status/progress and relevant safety information, please refer to the IB.

3.1.2 Cohort 2 with JACI-35.054

The administration schedule, route of administration, duration of treatment and follow-up are identical to cohort 1.

Up to 3 dose levels of JACI-35.054 administered by the intramuscular route will be tested in up to 3 sub-cohorts.

Sub-cohort 2.1 (8 subjects):

JACI-35.054 at 15 µg/dose will be administered to 6 subjects and the placebo will be administered to 2 subjects. The safety and tolerability data after all subjects have received the second injection in sub-cohort 2.1 will permit dose escalation after review by the DSMB.

Sub-cohort 2.2 (8 subjects) - Optional:

JACI-35.054 at up to 60 µg/dose will be administered to 6 subjects and the placebo will be administered to 2 subjects. This sub-cohort will be optional and may be conducted based on good safety and tolerability observed in sub-cohort 2.1 and in case the antibody response in this previous sub-cohort is anticipated to be optimized at the dosage of 60 µg. At the time of the version of this protocol, this sub-cohort with a dosage of 60 µg/dose has been initiated following the dose escalation after the 15 µg/dose sub-cohort.

Sub-cohort 2.3 (8 subjects) - Optional:

JACI-35.054 at up to 150 µg/dose will be administered to 6 subjects and the placebo will be administered to 2 subjects. This sub-cohort will be optional and may be conducted based on good safety and tolerability observed in sub-cohort 2.2 and in case the antibody response in this previous sub-cohort is anticipated to be optimized at a higher dosage.

For further information on the current study status/progress and relevant safety information, please refer to the IB.

3.1.3 Cohort 3 with ACI-35.030 and JACI-035.054 in Sequential Administration

The administration schedule, route of administration, duration of treatment and follow-up are identical to cohorts 1 and 2 but both vaccines ACI-35.030 and JACI-35.054 will be administered in a sequence based on non-human primate immunogenicity data and on data reviewed during the previous sub-cohort interim analyses. In any of the sub-cohorts listed below, the selected

dosages of the 2 study vaccines will have been evaluated in cohorts 1 and 2 and safety monitored for at least 10 weeks, and safety data of the 2 study vaccines reviewed by the DSMB.

Sub-cohort 3.1 (8 subjects)

ACI-35.030 and JACI-35.054 at dosages previously evaluated in cohorts 1 and 2 respectively and found to be safe after a study treatment period of at least 10 weeks will be administered sequentially to 6 subjects and placebo will be administered at each dosing occasion to 2 subjects. Immunizations with ACI-35.030 will be performed at months 0 (Week 0) and 2 (Week 8), and with JACI-35.054 at month 6 (Week 24) and 12 (Week 48).

Sub-cohort 3.2 (8 subjects) - Optional

This optional sub-cohort may include a different sequence of administration than in sub-cohort 3.1 and/or different dosages of ACI-35.030 and/or JACI-35.054 based on available safety, tolerability and immunogenicity data from previous sub-cohort interim analyses. Dosages of ACI-35.030 and of JACI-35.054 will have been previously evaluated in cohorts 1, 2 and/or 3 without significant safety or tolerability concerns.

Sub-cohort 3.3 (8 subjects) - Optional

This optional sub-cohort may include a different sequence of administration than in sub-cohorts 3.1 and 3.2 and/or different dosages of ACI-35.030 and/or JACI-35.054 based on available safety, tolerability and immunogenicity data from previous sub-cohort interim analyses. Dosages of ACI-35.030 and of JACI-35.054 will have been previously evaluated in cohorts 1, 2 and/or 3 without significant safety or tolerability concerns.

3.1.4 Sub-cohort expansion

Optional sub-cohort expansion of up to 16 additional subjects (i.e. up to 12 on active treatment and up to 4 on placebo per sub-cohort) may be considered in sub-cohort(s) with a favorable safety and immunogenicity profile with the objective to confirm the initial sub-cohort(s) immunogenicity results. The number of additional subjects to be enrolled will be determined based on the observed immunogenicity response rate in the sub-cohort(s) and on the potential gain in precision of the response rate estimate in the expanded sub-cohort(s), and will not exceed 16 (i.e., up to 24 subjects overall per sub-cohort). In addition, all other immunogenicity endpoints available at the interim timepoint may be considered. The dose-level, sequence of administration and administration regimen of study vaccine(s) will be identical to the initial part of the sub-cohort.

3.1.5 Safety follow-up

All subjects will be kept under clinical observation for 24 hours after the first administration of study vaccine and for 4 hours after subsequent administrations of study vaccine. During that period, the vital signs will be monitored and the injection site will be observed on a regular basis. A physical examination will be performed prior to discharge. The clinical observation may be prolonged in case of safety concerns according to the clinical judgement of the investigator. A subsequent safety assessment will also be performed 48 to 72 hours after each immunization by telephone call for all study subjects. For adverse events considered possibly or probably related to the study vaccine of greater than mild severity according to investigator assessment, subjects will be required to return to the clinic for additional safety assessment. In each sub-cohort, the first dosing of the first 4 subjects should be performed once the safety assessment at 48 to 72 hours of the previous subject has been performed. This will allow the site principal investigator to assess whether there is any clinically relevant safety issue related to study vaccine prior to

dosing the subsequent subject. Safety laboratory samples will be collected at baseline, prior to each injection and 2-4 weeks after each injection. Laboratory results will be reviewed by the Medical Monitor on a periodic basis including all out of range values.

All treated subjects will have a safety follow-up period of 24 weeks (6 months) after the end of the treatment period. During this period, subjects will be asked to attend a first follow-up visit 19 weeks after the last administration and a last visit at the end of the follow-up period (26 weeks after the last administration).

Additionally to the monitoring and recording of the AEs throughout the course of the study and the reporting of SAEs, participant safety will be monitored throughout the study with regular review of safety data by a Data and Safety Monitoring Board (DSMB). The DSMB will consist of specific experts in the field, who will meet on a regular basis to review the safety data of the participants. The outcome of the DSMB meeting will be registered in minutes and will include a recommendation as to whether to continue the study as planned or to modify or stop the study. The DSMB's responsibilities and activities will be described in a charter.

3.1.6 Post-injection period extension (optional)

Based on immunogenicity data, subjects from sub-cohort(s) may be invited to attend two additional visits (at Week 86 and Week 98) taking place after the end of the safety follow-up period (week 74) in order to evaluate the durability of the immune response and collect additional biomarker data. All subjects participating in this post-injection period extension will have to reconsent. During this additional 24-week (6-month) period, blood will be collected for immunogenicity and biomarker testing. The aim of this optional study extension is to help inform the dosing regimen in subsequent clinical trials of the vaccine(s). AEs/SAEs and concomitant medications (new and/or changes from the previous visit) will also be collected during these 2 additional visits. Upon investigator's medical judgement, other assessments can be performed during this period (e.g., MRI, ECG, etc.) if clinically indicated.

In the Netherlands, the subjects' decisional capacity will be re-assessed before consenting for this optional post-injection extension.

3.1.7 Interim analyses (IA)

Interim analyses of safety, tolerability and immunogenicity data may be conducted in each sub-cohort, including expanded sub-cohorts, as follows:

- all available subjects in the sub-cohort have completed Visit 4 (Week 10), i.e. 2 to 4 weeks after the second injection
- all available subjects in the sub-cohort have completed Visit 6 (Week 26), i.e. 2 to 4 weeks after the third injection
- all available subjects in the sub-cohort have completed Visit 9 (Week 50), i.e. 2 to 4 weeks after the last injection at Week 48
- all available subjects in the sub-cohort have completed Visit 11 (Week 74), i.e. end of the safety follow-up period

If the post-injection period is extended in any sub-cohort, IA of immunogenicity data may also be conducted at Visit 13 (Week 98), and optionally at Visit 12 (Week 86).

Available biomarker data may also be reviewed during any of these IAs. The IAs described above may also be performed for any expanded sub-cohort.

Additional IAs to review the sustainability of immune response data may be conducted between weeks 26 and 50 and between weeks 50 and 74.

Independently of the above-mentioned interim analyses, the DSMB will perform regular reviews of the safety and tolerability data throughout the study.

Rationale and Scope of IAs:
The objectives of the above mentioned IAs in the sub-cohorts (whether expanded or not) is to ensure a regular review of safety, tolerability and immunogenicity with the support of optional biomarker data. A decision to escalate to the next dose in one given sub-cohort may be taken once IA data of the previous dose are available from Visit 4 (Week 10) onwards and pending prior DSMB approval. A decision to expand a sub-cohort may be taken based on satisfactory review of safety data by the DSMB and analysis of immunogenicity IA data of this sub-cohort dose from Visit 4 (Week 10) onwards. The IA at the end of the optional extended post-injection period is intended to monitor the durability of the immune response and any biomarker changes in blood.

3.1.8 Schematic overview of cohorts design

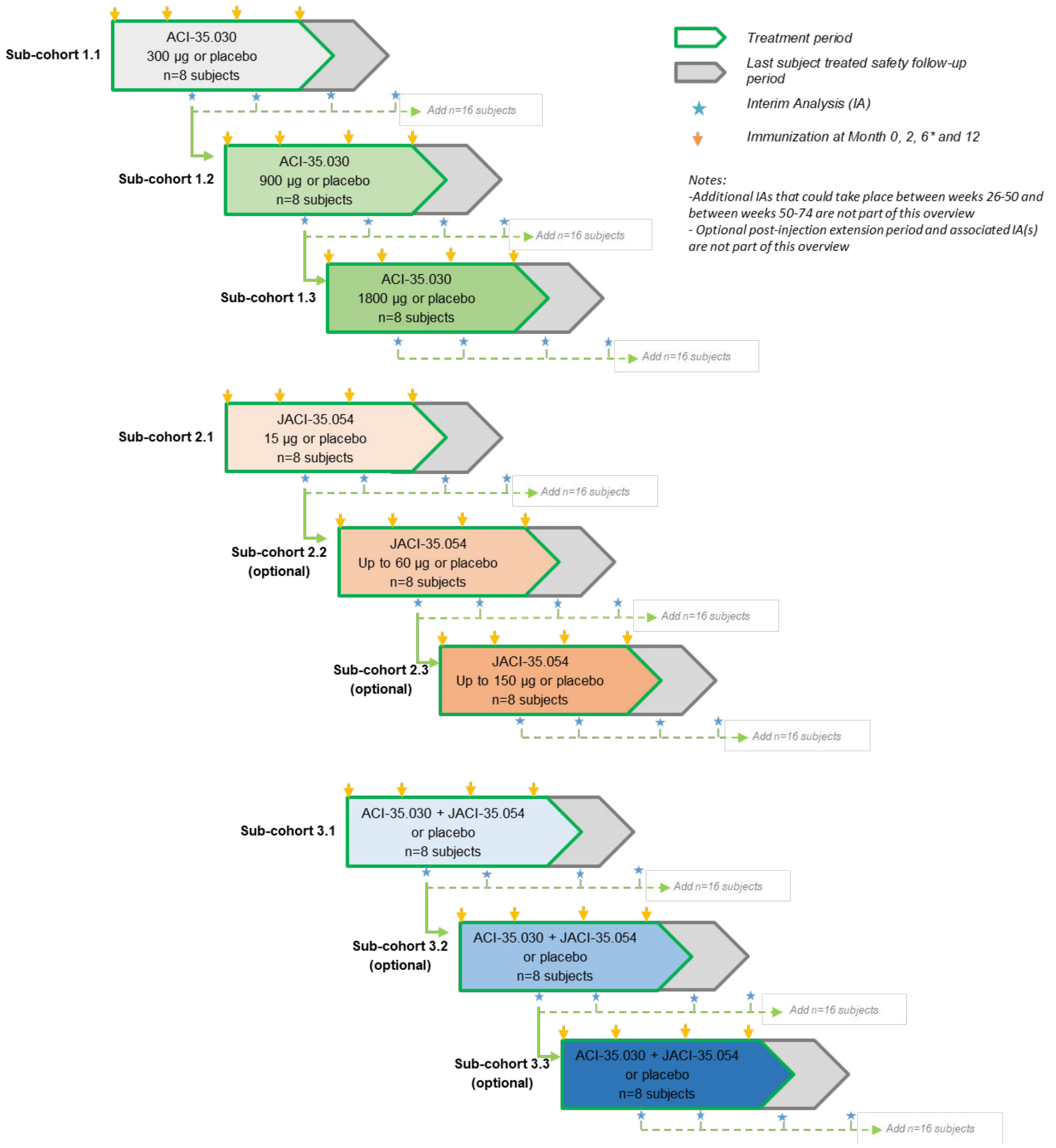

NB: The start of subject screening and treatment in cohort 2 is not linked to any IA performed in cohort 1. It is anticipated that screening in sub-cohort 2.1 will be done in parallel with screening for sub-cohort 1.2. Potential lags in the start of the screening may occur depending on e.g. subject recruitment rate or regulatory approval timelines.

The start of subject screening and treatment in cohort 3 will be based on IA data generated in sub-cohorts 1.1, 1.2, 1.3 and 2.1, and if needed in sub-cohort 2.2, from at least week 10 onwards.

As specified previously, several IAs will be performed in the study to allow for dose escalation, decision on expansion or potential comparison of cohorts.

All IAs planned for cohort 1, 2 and 3 are described also in Section 5.3.8.

Based on immunogenicity data, subjects from sub-cohort(s) may be invited to attend two additional visits (at Week 86 and Week 98) taking place after the end of the safety follow-up period (week 74). In such a case, an IA of immunogenicity data as well as available biomarker data may also be conducted at Visit 13 (Week 98), and optionally at Visit 12 (Week 86), to monitor the durability of the immune response and any biomarker changes in blood.

***Due to the Covid-19 pandemic, local travel restrictions in Finland effective as of 18 March 2020, and the need to preserve the safety of study participants, modifications are made to the schedule of immunizations of sub-cohort 1.1 subjects having not received the 3rd injection at month 6 already**: immunizations at month 6 (week 24) will not be performed in 7 of 8 sub-cohort 1.1 subjects for whom the planned Visit 5 does not allow all assessments to be done according to the protocol.

3.2 Study Duration

3.2.1 Screening Period

The screening period for each subject will be up to 42 days. Screening assessments will be done sequentially over multiple visits as described in the Schedule of Assessment (see section 3.5.1).

3.2.2 Treatment Period

In all cohorts, the treatment duration is anticipated to be 50 weeks (12 months).

3.2.3 Follow-up Period

Subjects participating in the study will be followed up for 24 weeks starting 2 weeks after the last study vaccine administration (treatment completion or early termination). The follow-up period will consist of on-site visits 19 weeks and 26 weeks (6 months) after the last administration.

3.2.4 Post-Injection Period Extension (optional)

If the post-injection period is extended in one or more sub-cohorts as described in section 3.1.6, subjects of the selected sub-cohort(s) will be asked to attend two additional visits over 24 weeks (6 months) after the end of the safety follow-up period and up to week 98.

3.2.5 Overall Study Duration

The duration of participation in the study for subjects completing the study is up to 80 weeks (around 1.5 year) from first screening assessment to the last safety follow-up visit, and up to around 104 weeks (around 2 years) in case the post-injection period is extended by 24 weeks (6 months) at the end of the safety follow-up period (optional).

3.2.6 End of Study Definition

A subject is considered to have completed the study if he or she has completed all phases of the study including the last visit or the last scheduled procedure shown in the Schedule of Assessments, Section 3.5, including the optional post-injection extension period, if applicable.

The end of the study is defined as completion of the last visit of the last participating subject, including the optional post-injection extension period, if applicable.

3.3 Selection of Study Population

Subjects will be 50-75 years old with a clinical diagnosis of mild AD or MCI as assessed by NIA-AA core clinical criteria (McKhann *et al*, 2011, see Appendix 1) and a Clinical Dementia Rating Scale (CDR) global score of 0.5 or 1.

3.3.1 Inclusion Criteria

Subjects meeting all of the following inclusion criteria at screening should be considered as eligible to participate to the study:

1. Male or female with age from 50 and up to 75 years old inclusive.
2. Mild Cognitive Impairment (MCI) due to AD or Mild AD according to NIA-AA criteria and a Clinical Dementia Rating Scale (CDR) global score of 0.5 or 1 respectively.
3. MMSE score of 22 or above.
4. Abnormal level of CSF Abeta amyloid 42 (Aß42) consistent with AD pathology at screening.
    - In borderline cases for CSF Aß42 levels, other results may be considered to help determine amyloid positivity, e.g., the Aß42/Aß40 ratio and, on a case by case basis, a history of positive amyloid PET scan or positive CSF Aß42 level.
    - Results from CSF sampling performed within 6 months prior to screening are acceptable on a case by case basis provided that they are consistent with the presence of amyloid pathology and that the corresponding CSF sample can be used in the study for testing.
5. Subjects either not taking any marketed treatment for AD or receiving a stable dose of an acetylcholinesterase inhibitor and/or memantine for at least 3 months prior to baseline.
6. Subjects cared for by a reliable informant or caregiver to assure compliance, assist with clinical assessments and report safety issues.
7. Women must be post-menopausal for at least one year and/or surgically sterilized. Women of childbearing potential or not post-menopausal must have a negative blood pregnancy test at screening (blood draw between day -14 and day -3 prior to baseline) and be willing to use highly effective methods of contraception from the screening visit until the end of their participation. Urine pregnancy testing will be performed throughout the treatment period to determine if the subject can continue receiving the study vaccine. Male participants in the trial with female partners of child bearing potential are required to use barrier methods of contraception (condoms with spermicide) in addition to contraceptive measures used by female partners during the whole study duration.
8. Subjects who in the opinion of the investigator are able to understand and provide written informed consent. In the Netherlands, the subjects' decisional capacity will be also assessed and must be consistent with the ability to provide informed consent using the MacArthur Competency Tool for Clinical Research in order to evaluate their abilities in the areas of understanding, reasoning, appreciation and choice.
9. Both subject and informant or caregiver must be fluent in one of the languages of the study and able to comply with all study procedures, including lumbar punctures.

3.3.2 Exclusion Criteria

Subjects meeting any of the following exclusion criteria at screening should not be considered as eligible to participate to the study:

1. Participation in previous clinical trials for AD and/or for neurological disorders using active immunization unless there is documented evidence that the subject was treated with placebo only and the placebo vaccine is not expected to induce any specific immune response.

2. Participation in previous clinical trials for AD and/or for neurological disorders using any passive immunization within the past 6 months (or 5 half-lives of the investigational antibody, whichever is longer) prior to screening unless there is documented evidence that the subject was treated with placebo only and the placebo is not expected to induce any specific immune response.
3. Participation in previous clinical trials for AD and/or for neurological disorders using any small molecule drug including BACE-1 inhibitors within the past 3 months prior to screening.
4. Concomitant participation in any other clinical trial using experimental or approved medications or therapies.
5. Presence of positive ANA titers at a dilution of at least 1:160 in subjects without clinical symptoms of auto-immune disease.
6. Current or past history of auto-immune disease, or clinical symptoms consistent with the presence of auto-immune disease.
7. Immune suppression including but not limited to the use of immunosuppressive drugs or systemic steroids unless they have been prescribed transiently more than 3 months prior to screening.
8. History of severe allergic reaction (e.g., anaphylaxis) including but not limited to severe allergic reaction to previous vaccines and/or medications.
9. Prior history of clinically significant hypoglycaemic episodes.
10. Drug or alcohol abuse or dependence currently met or within the past five years according to DSM-V criteria.
11. Any clinically significant medical condition likely to interfere with the evaluation of safety and tolerability of the study treatment and/or the adherence to the full study visit schedule.
12. Any clinically significant medical condition likely to impact the immune system (e.g, any history of acquired or innate immune system disorder).
13. Use of hydralazine, procainamide, quinidine, isoniazide, TNF-inhibitors, minocycline within the last 12 months prior to screening.
14. Use of diltiazem unless on a stable dose for at least 3 months prior to screening.
15. Significant risk of suicide defined, using the Columbia-Suicide Severity Rating Scale, as the subject answering: “yes” to suicidal ideation questions 4 or 5 or answering: “yes” to suicidal behavior within the past 12 months.
16. Concomitant psychiatric or neurologic disorder other than those considered to be related to AD (e.g. head injury with loss of consciousness, symptomatic stroke, Parkinson’s disease, severe carotid occlusive disease, TIAs).
17. History or presence of uncontrolled seizures. If history of seizures, they must be well controlled with no occurrence of seizures within 2 years prior to screening. The use of anti-epileptic medications is permitted if at stable dose for at least 3 months prior to screening.
18. History of meningoencephalitis within the past 10 years prior to screening.
19. Subjects with a history of hemorrhagic and/or non-hemorrhagic stroke.
20. Presence or history of peripheral neuropathy.
21. History of inflammatory neurological disorders with potential for CNS involvement.
22. Screening MRI scan showing structural evidence of alternative pathology not consistent with AD which could cause the subject's symptoms. Evidence of space occupying lesions other than benign meningioma of less than 1 cm diameter, more than two lacunar infarcts or one single infarct larger than 1 cm in diameter or any single area of superficial siderosis or evidence of a prior macro-hemorrhage ≥10 mm. Microbleeds on T2* MRI are allowed up to a maximum of 10, regardless of the location.
23. MRI examination cannot be done for any reason, including but not limited to metal implants contraindicated for MRI studies and/or severe claustrophobia.
24. Significant hearing or visual impairment or other issues judged relevant by the investigator preventing to comply with the protocol and to perform the outcome measures.

25. Clinically significant infections or major surgical operation within 3 months prior to screening. Planned surgery anticipated to occur during participation in the study must be reviewed and approved by the medical monitor at screening.
26. Any vaccine received within the past 2 weeks before screening, including influenza vaccine.
27. Clinically significant arrhythmias or other clinically significant abnormalities on ECG at screening.
28. Myocardial infarction within one year prior to baseline, unstable angina pectoris, or significant coronary artery disease.
29. History of cancer within the past 5 years other than treated squamous cell carcinoma, basal cell carcinoma and melanoma in situ, or in-situ prostate cancer or in-situ breast cancer which have been fully removed and are considered cured.
30. Clinically significant deviations from normal values for hematologic parameters, liver function tests, and other biochemical measures, that are judged to be clinically significant in the opinion of the investigator.
31. Pregnancy confirmed by blood test at screening, or subject planning to be pregnant or lactating.
32. Receipt of any anticoagulant drug or antiplatelet drug, except aspirin at doses of 100 mg daily or lower (in order to avoid risk of bleeding during scheduled or unscheduled lumbar puncture).
33. Receipt of any antipsychotic drugs unless on stable low doses for the treatment of insomnia.
34. Donation of blood or blood products within 30 days prior to screening or plans to donate blood while participating in the study.
35. Positive VDRL consistent with active syphilis at screening.
36. Positive HIV test at screening.
37. Laboratory or clinical evidence of active hepatitis B and/or C at screening.
38. Serum creatinine greater than 1.5x upper limit of normal, abnormal thyroid function tests or clinically significant reduction in serum B12 or folate levels (note: all oral doses of thyroid replacement agents, B12 or folate have to be stable for at least 3 months prior to screening).

3.3.3 Removal of Subjects from Therapy or Assessment

A subject will be considered to have completed the study when he or she completes the Visit 11 at Week 74 (or the Visit 13 at Week 98 in case of optional post-injection period extension). If a subject is discontinued at any time after randomization into the study, the investigator will make every effort to follow the subject and complete the premature discontinuation assessments as shown in Section 3.5.3.
Additional participants may be recruited to replace withdrawn subjects unless the subject withdrew for safety reasons.

A termination electronic case report form (eCRF) page should be completed for every subject who received investigational product, whether or not the subject completes the study. The reason for any early discontinuation should be indicated on this form.

The primary reason for a subject discontinuing early should be selected from the following standard categories of early termination:

- Adverse Event (Adverse Reaction): Clinical or laboratory events occurred that, in the medical judgement of the investigator for the best interest of the subject, are grounds for discontinuation. This includes serious and non-serious adverse events (AEs) regardless of relation to the investigational product.
- Death: The subject died.
- Withdrawal of Consent:

  - The subject or caregiver desired to withdraw from further participation in the study in the absence of an investigator-determined medical need to withdraw. If the subject gave a reason for withdrawing, it should be recorded in the eCRF. In case of caregiver withdrawal, the subject can continue in the study if a new caregiver consents to act in this role.
  - In the Netherlands, subjects who become unable to maintain their decisional capacity during the course of the study as assessed with the MacCAT-CR interview (refer to section 3.5.6 and 6.1.3).
- Protocol Violation: The subject's findings or conduct failed to meet the protocol entry criteria or failed to adhere to the protocol requirements (e.g., drug noncompliance, use of prohibited concomitant treatment (section 3.4.7.2), failure to return for a defined number of visits). The violation is important enough to justify early discontinuation since it would hamper the evaluation of the primary endpoints of the study.
- Lost to Follow-up: The subject stopped coming for subsequent study visits and study personnel were unable to contact the subject.
- Other: The subject was discontinued for a reason other than those listed above, such as termination of study by sponsor.

3.4 Treatments

3.4.1 Details of Study Treatments

Name and Description

Study Active Treatment in cohort 1: ACI-35.030 is a sterile suspension supplied in vials.

Study Active Treatment in cohort 2: JACI-35.054 is a reconstituted solution of the conjugate CRM197-pTau with 2 adjuvants, i.e. Aluminum Hydroxide and CpG7909. Each product is a sterile suspension supplied in vials.

Study Active Treatments in cohort 3: ACI-35.030 and JACI-035.054.

The placebo of the study consists of a PBS solution in vials.

Manufacturing

ACI-35.030 is manufactured by [redacted], a contract manufacturing organization located in [redacted].

The conjugate and adjuvants of JACI-35.054 are manufactured by the following contract manufacturing organizations:

- CRM197-pTau: [redacted]
- Aluminum Hydroxide: [redacted]
- CpG7909: [redacted]

The Placebo is manufactured by [redacted].

Treatment Administration and Preparation

Because the appearance of the liquid in the vial differs between placebo and active treatments (ACI-35.030 or JACI-35.054), the dose preparation will be performed by an unblinded pharmacist or duly trained delegate, independent from the study medical team. An unblinded study nurse will then obtain the study product from the pharmacy and administer the study medication (study vaccine or placebo). JACI-35.054 must be reconstituted before administration. Contact between the unblinded site staff and the blinded site staff or subject must not disclose treatment assignment. Study site staff other than the unblinded pharmacist and the unblinded study nurse must not be allowed to know the study product assigned to any study subject. To maintain the

blinding to subject during study medication administration, the syringes used for injection will be masked.

Treatment administration for subjects in cohorts 1, 2 and 3: To administer doses of study active treatment or corresponding placebo, the following dose preparation should be followed.

**Cohort 1**	
Sub-cohort 1.1: 300 µg antigen or placebo administration	To administer 300 µg of antigen or corresponding placebo, 1 vial of study medication/placebo should be used. [redacted]
Sub-cohort 1.2: 900 µg antigen or placebo administration	To administer 900 µg of antigen or corresponding placebo, 1 vial of study medication/placebo should be used. [redacted]
Sub-cohort 1.3: 1800 µg antigen or placebo administration	To administer 1800 µg of antigen or corresponding placebo, 2 vials of study medication/placebo should be used. [redacted]
**Cohort 2**	
Sub-cohort 2.1: 15 µg JACI-35.054 or placebo administration	To administer 15 µg of JACI-35.054 or corresponding placebo, 1 vial of study medication (obtained after reconstitution)/placebo should be used. [redacted]
Optional Sub-cohorts 2.2 and 2.3: Up to 60 µg and up to 150 µg JACI-35.054 or placebo administration	To administer the required dose of CRM197-pTau or corresponding placebo, 1 vial of study medication (obtained after reconstitution)/placebo should be used. [redacted]
**Cohort 3**	
Sub-cohort 3.1: ACI-35.030 and JACI-035.054 (dosages to be determined) or placebo administration	The same instructions for dose preparation provided for cohort 1 and 2 should be followed depending on the selected dosage and on the study vaccine administered.

Optional Sub-cohort 3.2 and 3.3: ACI-35.030 and JACI-035.054 (respective dosages to be determined) or placebo administration	The same instructions for dose preparation provided for cohort 1 and 2 should be followed depending on the selected dosages and on the study vaccine administered.

A complete product handling instruction manual will be provided to the study site staff responsible for drug handling, reconstitution and administration.

Subjects will be kept under clinical observation for 24 hours after the first administration of study vaccine and for 4 hours after subsequent administrations of study vaccine (second, third and fourth administrations).

In each sub-cohort, the first dosing of the first 4 subjects should be performed once the safety assessment at 48 to 72 hours of the previous subject has been performed. This will allow the site principal investigator to assess there is no clinically relevant safety issue related to study vaccine prior to dosing the subsequent subject.

Treatment administration for the subsequent cohorts: information on the treatment administration will be provided at a later stage in a dedicated substantial protocol amendment.

Distribution and Storage

The vials will be supplied by the appointed Drug Distribution Company to the study sites after the study has been approved by the Ethics Committee and applicable regulatory authorities.

All vials will be supplied to the pharmacist. The person appointed to administer the product will collect the appropriate vials and will proceed to the study vaccine injection. The empty and half-empty vials will be returned to the pharmacist for accountability.

Vials of ACI-35.030 and JACI-35.054 adjuvants (Aluminum hydroxide, CpG7909) should be stored refrigerated at 2-8 °C. Vials of JACI-35.054 conjugate (CRM197-pTau) should be stored frozen at -85 to -65°C. The pharmacist agrees not to distribute the test articles to anyone, except to the appointed person who is responsible for administering the product. This person agrees not to administer the products to anyone else, except to the subjects having given written informed consent to take part in this study.

Unused product remaining at completion of the study should be returned to the Drug Distribution Company or destroyed on site upon approval from Sponsor.

3.4.2 Dosage Schedule

For cohorts 1 2 and 3, immunizations will be performed at months 0 (Week 0), 2 (Week 8), 6 (Week 24)* and 12 (Week 48). Refer to the Schedule of Assessment specified in section 3.5.

***Due to the Covid-19 pandemic, local travel restrictions in Finland effective as of 18 March 2020, and the need to preserve the safety of study participants, modifications are made to the schedule of immunizations of sub-cohort 1.1 subjects having not received the 3rd injection at month 6 already:** immunizations at month 6 (week 24) will not be performed in 7 of 8 sub-cohort 1.1 subjects for whom the planned Visit 5 does not allow all assessments to be done according to the protocol.

3.4.3 Treatment Assignment

A randomization list based on a sequence of numbers, and individual medication codes will be computer generated. Study medication will be labelled with the corresponding medication number. Investigators will use a secure web-based system to randomize eligible subjects to study medication. An Interactive Response Technology (IRT) system will be used for randomization in the study. At screening, participating subjects will be given a subject number that will also correspond to the subject's identification in the study. Assigned numbers will be unique identifiers with 4 digits for the site and 2 last digits for the sequential order of enrolment at a given site.

3.4.4 Drug Packaging and Blinding

Packaging and Labelling

The out-packaging and the labeling of all active treatment and placebo vials are performed by [redacted] according to the EU GMP and local regulations. Information on the subject identity and visit number will have to be added to the boxes and vials assigned to subjects by the site.

Blinding

Study participants, site personnel, CRO, study vendors, sponsor will be blinded to treatment assignment until all subjects in a given sub-cohort have reached the end of the safety follow-up visit (Visit 11, Week 74).

If the follow-up of a specific sub-cohort is extended to allow for the assessment of the durability of the immune response, the study participants and site investigator/personnel will remain blinded to the treatment assignment until the end of the extended period (Visit 13, Week 98). During this extended follow-up period (weeks 74-98), designated sponsor representatives may be unblinded to the treatment assignment to allow for data review in preparation for subsequent trials. Fully unblinded designated sponsor representatives will have no access to the study database and will not be authorized to share any unblinding information with any blinded study staff.

The study is unblinded to the pharmacist and any duly trained and delegated staff responsible for the IMP receipt, accountability and preparation of the syringes, as well as to the study nurse responsible for treatment administration who should not be otherwise involved in the study. In addition, an unblinded monitor from the CRO will control study medication (drug accountability) on site. The Principal Investigator will be provided with the site specific unblinded list after data lock. Database lock will be performed after all subjects have completed the study. It is the Principal investigators' responsibility to decide whether this information should or should not be shared with the subjects.

In order to remain blinded, raters performing the cognitive tests should not have access to any clinical assessment data (e.g. clinical rating scales, adverse events).

Individual immunogenicity or biomarker results will not be shared with any site personnel until after database lock.

Unblinding of individual subjects is restricted to emergency situations and should only be used under circumstances where knowledge of the treatment is considered necessary for the proper management of subject safety. If the treatment code is unblinded, the reason and the date should be documented by the Investigator and the Sponsor should be informed in a promptly manner.

In emergency circumstances where the investigator identifies an urgent clinical need to know whether the subject is receiving active medication or placebo, the IRT allows the immediate unblinding of the subject by the investigator directly. In such cases the rationale must be documented on the subject source data, with immediate notification of the monitor and medical monitor.

The code break will also be reported in the subject specific CRF and events leading to the emergency breaking will be recorded in the serious adverse event (SAE) report form.

Subjects will be given a specific study card containing the information that they are participating in the study and the contact details of the investigator.

3.4.5 Drug Inventory and Accountability

The investigator must keep an accurate accounting of the number of investigational product units delivered to the site, dispensed to subjects, and returned to the sponsor or other disposition during and at the completion of the study. The investigational product must be dispensed to subjects only by an appropriately qualified person. The investigational product is to be used in accordance with the protocol and should be administered to subjects by qualified and pre-identified study site personnel who are under the responsibility of the site principal investigator. Investigators or dedicated study personnel should maintain records that document adequately that the subjects were provided the doses specified by the protocol and reconcile all investigational products received at the site before final disposition. At the end of the study, or as directed, all study vaccines, including unused, partially used, and empty containers, will be returned to the Drug Distribution company or destroyed on site upon approval from Sponsor.

Product accountability and product reconstitution forms will be used by the pharmacist of the study site/the assigned person responsible for the injection. Used vials will be kept for accountability. All study vials (investigational product and placebo) received and dispensed throughout the study period will be inventoried and accounted for the trial period by the pharmacy/site team on the corresponding "product accountability form".

3.4.6 Treatment Compliance

Administration of the investigational products will be performed by pre-identified and qualified study personnel. Any noncompliance to study treatment (e.g. incorrect dose injected, treatment interruption) will be recorded.

3.4.7 Prior and Concomitant Illness and Treatments

3.4.7.1 Prior and Concomitant Illnesses

Investigators should document any prior relevant illnesses (i.e. with an end date before the informed consent is given) in the medical history of the subject. Additional illnesses present at the time informed consent is given are to be regarded as concomitant illnesses. Illnesses first occurring or detected during the study and/or worsening of a concomitant illness during the study are to be documented as AEs on the eCRF.

3.4.7.2 Prior and Concomitant Treatments

Prior treatments, defined as those ongoing or started within 3 months before screening and stopped before the first dosing, should be recorded in the eCRF as prior medications. Concomitant treatments, defined as treatments ongoing at the time of the first dosing or started after the first dosing should be recorded in the eCRF as concomitant medications.

After randomization, subjects may receive the prescribed concomitant medications and treatments as indicated by their clinical condition. Concomitant medication and treatments should be kept unchanged as much as possible during the course of the study. Any change in the nature and/or the dose of the concomitant medication and treatments should be recorded in the appropriate section of the eCRF.

All medications and treatments must be documented in the concomitant medication section of the case report forms.

Subjects receiving acetylcholinesterase inhibitor and/or memantine should have been on a stable dosage for at least 3 months prior to baseline.

The following treatments are not permitted throughout the study:

- Anticoagulants or antiplatelet drugs, except aspirin at doses of 100 mg daily or lower.
- Standard of care immunizations during the period ranging from 2 weeks before or after any administration of study vaccine to avoid potential interference.
- Hydralazine, procainamide, quinidine, isoniazide, TNF-inhibitors, minocycline.
- Diltiazem unless subject is on stable dose 3 months prior to screening.
- Any investigational or non-investigational drugs given as part of another clinical trial.

Wherever possible any change in current medication or introduction of new standard of care medications must be discussed first with the Study Investigator, and if appropriate, the Medical Monitor and Sponsor.

3.5 Assessments

3.5.1 Schedule of Assessments

The study plan proposed is for cohort 1 (ACI-35.030 or placebo), cohort 2 (JACI-35.054 or placebo) and cohort 3 (ACI-35.030 and JACI-35.054, or placebo).

Study Plan	Screening	Treatment period																	Follow-up period		Optional Post-injection period extension ∂ π	
Visit<br>Time (visit or week ± days)	V_S<br>-42d to -3d	V_1<br>0	P_1<br>V_1<br>+2-3d	V_2<br>w2<br>±3d ∞	V_3<br>w8<br>±3d	P_2<br>V_3<br>+2-3d	V_4<br>w10<br>±3d ∞	$V_{4.1}$<br>W15<br>±3d ∂	$V_{4.2}$<br>W20<br>±3d ∂	V_5 Δ<br>w24<br>±7d	P_3 Δ<br>V_5<br>+2-3d	V_6 Δ<br>w26<br>±2d ∞	$V_{6.1}$<br>W31<br>±3d ∂	V_7 Δ<br>w36<br>±3d	$V_{7.1}$<br>W42<br>±3d ∂	V_8<br>w48<br>±7d	P_4<br>V_8<br>+2-3d	V_9<br>w50<br>±2d ∞	V_{10}<br>w67<br>±7d	V_{11}<br>w74<br>±7d	V_{12}<br>W86<br>±7d	V_{13}<br>W98<br>±7d
Treatment (immunization)		•			•					•						•						
Decisional capacity (MacCAT-CR)¤	•									•						•						
Subject Information / Consent	•																					
History (Subject medical history / Family history of AD)	•																					
Inclusion / Exclusion criteria	•	•																				
Adverse Events / Concomitant Medication §	•	•	•	•	•	•	•	•	•	•	•	•	•	•	•	•	•	•	•	•	•	•
Physical and neurological examination	•	•		•	•		•			•		•		•		•		•	•	•		
Vital signs	•	•		•	•		•			•		•		•		•		•	•	•		
Cognitive/Clinical assessments																						
- *C-SSRS, RBANS*	•	•										•						•		•		
- *MMSE*	•																					
- *CDR*	•	•‡										•						•		•		
- *NPI*		•										•						•		•		
Lumbar Puncture (CSF)*	•											•						•				
- *Cell Count (to be performed at site)/Inflammatory markers*	•											•						•				
- *Biomarkers (i.e. levels of amyloid, Total Tau, pTau, Tau fragments and other biomarkers)*	•											•						•				
- *CSF albumin*	•											•						•				
Blood																						
- *Hematology & biochemistry*	•	•		•	•		•			•		•		•		•		•	•	•		
- *Serum albumin*	•											•						•				
- *Serum pregnancy test* †	•																					
- *Screening tests: HIV, Hepatitis B and C, VDRL (syphilis serology), B12, folate, thyroid function tests, ApoE genotype*	•																					
- *Coagulation indices: PT (INR), aPTT*	•									•						•						
- *Anti-Tau IgG/IgM, anti-pTau IgG/IgM*	•	•		•	•		•	•	•	•		•	•	•	•	•		•	•	•	•	•
- *Cytokines*	•	•		•	•		•			•		•		•		•		•	•	•		
- *Biomarkers (i.e. Total Tau and pTau levels)*		•		•	•		•	•	•	•		•	•	•	•	•		•	•	•	•	•
- *T-Cell profile*		•										•						•				
- *ANA and anti-dsDNA*	•	•		•	•		•			•		•		•		•		•	•	•		
Urine																						
- *Routine urine evaluation*	•	•		•	•		•			•		•		•		•		•				
- *Urinary pregnancy test*		•			•					•						•						
ECG	•											•						•		•		
MRI	•						•					•						•		•		

¤ Only in the Netherlands. Initial assessment to be done prior to the informed consent signature. Lumbar punctures are mandatory procedures including at screening visit where the presence of amyloid will be assessed as an inclusion criterion.§ All subjects will be kept under clinical observation for 24 hours after the first administration of study vaccine and for 4 hours after subsequent administrations of study vaccine (second, third and fourth administrations). A subsequent safety assessment will also be performed 48 to 72 hours after each immunization by telephone call (P) for all study subjects. In each sub-cohort, the first dosing of the first 4 subjects should be performed once the safety assessment at 48 to 72 hours of the previous subject is done to confirm there is no clinically relevant safety issue related to study vaccine, according to the site principal investigator. ∞ Visit to be performed preferentially 2 weeks +/- 3 days and exceptionally up to 28 days after immunization. † Blood draw between day -14 and day -3 prior to baseline (V1). ‡For sub-cohort 1.1 only. Δ In sub-cohort 1.1: modification of V_5, P_3, V_6, V_7 procedures and assessments during Covid-19 pandemic. Refer to*

details in sections 3.5.2.10, 3.5.2.11, 3.5.2.12 and 3.5.2.14. ∂ Only applicable for sub-cohorts in which the subject has not yet reached the visit timepoint. If feasible, agreed with subject and locally permitted, these visits may also be performed at subject's home. π Optional extended study period, only applicable if a decision is made by the sponsor. Concerned subjects will consent and in the Netherlands, the subjects' decisional capacity will be assessed before consenting.

3.5.1.1 *Alternative Mode of Administration of Assessments to Reduce Safety Risks due to Participation during Covid-19 pandemic*

The below table applies to subjects who are already randomized (i.e., completed Vs and V1) in the event that the study procedures cannot be performed on-site (O) at the study facility due to the Covid-19 situation but is feasible and locally permitted to be conducted by phone (P) and/or at subject’s home (H). This approach will be implemented on a case-by-case basis after an evaluation of the risk for the participant to attend on-site visits (refer to section 1.5.2 Risks of Participation during Covid-19 Pandemic) or when travel restrictions/site’s policy prevent the on-site visit from being performed. Documentation regarding the mode of administration of study assessments will be maintained on-site in the source documents and/or the investigator site file.

Alternative modes of administration of cognitive tests will be used following specific training of raters and supporting study staff if applicable.

A physician (investigator or delegate) will always be responsible for the 4-hour safety observation post-dosing to ensure that any acute reactions can be addressed. Staff conducting home visits will be informed of pre-identified local acute care facility/system by the site PI.

Study Plan	Treatment period																Follow-up period		Optional Post-injection period extension [7, 9]	
Visit<br>Time (visit or week ± days)	P_1<br>V_1<br>+2-3d	V_2<br>w2<br>±3d	V_3<br>w8<br>±3d	P_2<br>V_3<br>+2-3d	V_4<br>w10<br>±3d	$V_{4.1}$<br>w15[7]<br>±3d	$V_{4.2}$<br>W20[7]<br>±3d	V_5<br>w24<br>±7d	P_3<br>V_5<br>+2-3d	V_6<br>w26<br>±2d	$V_{6.1}$<br>W31[7]<br>±3d	V_7<br>w36<br>±3d	$V_{7.1}$<br>W42[7]<br>±3d	V_8<br>w48<br>±7d	P_4<br>V_8<br>+2-3d	V_9<br>w50<br>±2d	V_{10}<br>w67<br>±7d	V_{11}<br>w74<br>±7d	V_{12}<br>W86<br>±7d	V_{13}<br>W98<br>±7d
Treatment (immunization)			H					H						H						
4-hours medical observation post dosing [1]			H					H						H						
Decisional capacity (MacCAT-CR) [8]								H/P						H/P						
Subject Information / Consent																				
History (Subject medical history / Family history of AD)																				
Inclusion / Exclusion criteria																				
Adverse Events / Concomitant Medication	P	H	H	P	H	H	H	H	P	H	H	H	H	H	P	H	H	H	H	H
Physical and neurological examination [2]		H	H		H			H		H		H		H		H	H	H		
Vital signs		H	H		H			H		H		H		H		H	H	H		
Cognitive/Clinical assessments																				
- *C-SSRS* [3]										H/P						H/P		H/P		
- *RBANS* [4]										H						H		H		
- *MMSE*																				
- *CDR* [3]										H/P						H/P		H/P		
- *NPI* [3]										H/P						H/P		H/P		
Lumbar Puncture (CSF) [5]										O						O				
- *Cell Count (to be performed at site)/Inflammatory markers*										O						O				
- *Biomarkers (i.e. levels of amyloid, Total Tau, pTau, Tau fragments and other biomarkers)*										O						O				
- *CSF albumin*										O						O				
Blood																				
- *Hematology & biochemistry*		H	H		H			H		H		H		H		H	H	H		
- *Serum albumin*										H						H				
- *Serum pregnancy test*																				
- *Screening tests: HIV, Hepatitis B and C, VDRL (syphilis serology), B12, folate, thyroid function tests, ApoE genotype*																				
- *Coagulation indices: PT (INR), aPTT*								H						H						
- *Anti-Tau IgG/IgM, anti-pTau IgG/IgM*		H	H		H	H	H	H		H	H	H	H	H		H	H	H	H	H
- *Cytokines*		H	H		H			H		H		H		H		H	H	H		
- *Biomarkers (i.e. Total Tau and pTau levels)*		H	H		H	H	H	H		H	H	H	H	H		H	H	H	H	H
- *T-Cell profile*										H						H				
- *ANA and anti-dsDNA*		H	H		H			H		H		H		H		H	H	H		
Urine																				
- *Routine urine evaluation*		H	H		H			H		H		H		H		H				
- *Urinary pregnancy test*			H					H						H						
ECG										H						H		H		
MRI [6]					O					O						O		O		

1. *At a minimum, a physician (investigator or delegate) will be present for the entire observation period. A resuscitation wallet will be brought to subject's home (anaphylactic treatment, adrenaline and portable automatic external defibrillator).*
2. *Procedure must be performed by a physician (investigator or delegate).*
3. *Phone rating should be favored over home rating if feasible to reduce the number of staff at subject's home.*
4. *Home rating will be performed. If feasible, virtual rating may replace home rating (i.e. rating by videoconference supported by home visit of an investigator or delegate).*

5. *At V6, the lumbar puncture will be performed either at the study facility or if locally feasible at an alternate (external) facility according to the visit window. If facilities are unavailable, the procedure will be postponed until the earliest possible timeslot. Should the lumbar puncture be delayed at V6, the timing of the last lumbar puncture at V9 will be adjusted or cancelled on a case-by-case basis to avoid repeated procedures within a short interval of time.*
6. *At V4, V6, V9 and V11, MRI scans will be performed either at the qualified imaging facility or if locally feasible at an alternate (non-qualified) facility according to the visit window. If facilities are unavailable, the procedure will be postponed until the earliest possible timeslot. Should a MRI scan be delayed, the timing of the subsequent scan will be adjusted or cancelled on a case-by-case basis to avoid repeated procedures within a short interval of time.*
7. *Only applicable for sub-cohorts in which the subject of the sub-cohort has not yet reached the visit timepoint.*
8. *Only in the Netherlands.*
9. *Optional extended study period, only applicable if a decision is made by the sponsor. Concerned subject will consent and in the Netherlands, the subjects' decisional capacity will be assessed before consenting.*

3.5.2 Study Procedures

During their participation in the study, subjects will have to undergo a certain number of procedures as summarized in the Schedule of Assessment (section 3.5.1). A list of the different procedures, samplings and type of data collected at each visit is available below for cohorts 1, 2 and 3. A more detailed description of the procedures the subject will undergo is available in sections 3.5.4 to 3.5.6.

Due to the resurgence of the Covid-19 pandemic in multiple participating countries since September 2020, an alternative mode of administration of study assessments will apply to randomized subjects who have previously received the first immunization if the study procedure cannot be performed on-site at the study facility and if the procedure is feasible and locally permitted to be administered by phone and/or at subject's home (see section 3.5.1.1 Alternative Mode of Administration of Assessments to Reduce Safety Risks due to Participation during Covid-19 pandemic).

3.5.2.1 Vs-Screening [day -42 to -3]

The procedures listed below will be performed sequentially over a period of 42 to 3 days prior to the baseline visit (Week 0). The sequence is specified in the Schedule of Assessments (section 3.5.1).

- Subject's decisional capacity to consent: MacCAT-CR interview (in the Netherlands). To be performed prior to the informed consent signature.
- Subject information and consent discussion
- Written informed consent from the subject
- Written informed consent from the caregiver
- Year of birth
- Gender
- Ethnic origin
- Women of child bearing potential: discussion on reliable contraceptive measures (refer to section 4.2.3)
- Report former brain MRI assessment (<1 year)
- Year of onset of disease
- Familial history for AD
- Previous medications for AD (describe)
- Relevant medical and surgical history for other diseases
- Prior medications
- AEs
- Review NIA-AA diagnostic criteria
- Cognition: MMSE, RBANS (form C)
- Clinical status: C-SSRS, CDR
- Physical and neurological examination
- Vital signs
- Blood sample collected for:
  - routine hematology and biochemistry evaluation, including CRP
  - antibody response components including for example anti-pTau and anti-Tau IgG and IgM titers
  - Inflammatory cytokines
  - coagulation indices assessment
  - screening tests: B12, folate, thyroid function tests, HIV, Hepatitis B and C and VDRL (syphilis serology), ApoE genotyping, serum pregnancy testing
  - ANA and anti-dsDNA testing

- Routine urine evaluation
- ECG
- Brain MRI
- Lumbar puncture for cerebrospinal fluid sample (12 mL) for cell count, inflammatory markers, exploratory biomarker testing and for confirming amyloid and Tau positivity according to inclusion criteria (note: blood for serum albumin should be taken and measured concomitantly).
- Review inclusion and exclusion criteria

3.5.2.1.1 *Re-screening:*

If a subject has entered a screening period and is considered eligible for the study but cannot be randomized in one sub-cohort for logistical reasons (e.g., due to completion of randomization in current sub-cohort(s)), the subject may be re-screened at a later time for potential randomization in subsequent sub-cohorts in the study.
In such cases, the following screening assessments should be re-done over a period of 42 to 3 days prior to the baseline visit (Week 0) to re-confirm eligibility, i.e.:

- Subject's decisional capacity to consent: MacCAT-CR interview (in the Netherlands). To be performed prior to the informed consent signature.
- Subject information and consent discussion
- Written informed consent from the subject
- Written informed consent from the caregiver
- Women of child bearing potential: discussion on reliable contraceptive measures (refer to section 4.2.3)
- Report former brain MRI assessment (<1 year)
- Previous medications for AD (describe)
- Relevant medical and surgical history for other diseases
- Prior medications
- AEs
- Review NIA-AA diagnostic criteria
- Cognition: MMSE, RBANS (form C)
- Clinical status: C-SSRS, CDR
- Physical and neurological examination
- Vital signs
- Blood sample collected for all lab tests applicable to Screening visit except ApoE genotyping
- Routine urine evaluation
- ECG
- Brain MRI should be repeated if clinically indicated (e.g. abnormality on neurological exam) or if the MRI scan from the preceding screening period is more than 6 months prior to re-screening
- Lumbar puncture for cerebrospinal fluid sample (12 mL) should be repeated if the procedure from the preceding screening period is more than 6 months before re-screening
- Review inclusion and exclusion criteria

3.5.2.2 Visit 1/ Baseline [Week 0]

- Check that inclusion/exclusion criteria are still applicable
- Subject and caregiver training to report any AE and SAE
- AEs
- Concomitant medications (describe new and/or changes from the previous visit)

- Physical and neurological examination
- Vital signs before the administration of study medication
- Cognition: RBANS (form A)
- Clinical status: CDR (only applicable to sub-cohort 1.1), NPI, C-SSRS
- Blood sample collected prior to administration of study medication for:
  - routine hematology and biochemistry evaluation, including CRP
  - antibody response components including for example anti-pTau and anti-Tau IgG and IgM titers
  - Inflammatory cytokines
  - AD biomarker assessment
  - T-Cell profile assessment
  - ANA and anti-dsDNA testing
- Routine urine evaluation and urinary pregnancy test
- Administration of study medication
- 24-hour clinical observation:
  - Vital signs and observation of injection site 1, 2, 4, 8, 12 and 24 hours after the administration of study medication
  - Physical examination before discharge

3.5.2.3 *Phone call 1 [Week 0]*

- AEs
- Concomitant medications (describe new and/or changes from the previous visit)
- In case of safety concerns, as per investigator assessment, an additional visit will be scheduled in order to further assess the event(s).

3.5.2.4 *Visit 2 [Week 2]*

- AEs
- Concomitant medications (describe new and/or changes from the previous visit)
- Physical and neurological examination
- Vital signs
- Blood sample collected for:
  - routine hematology and biochemistry evaluation, including CRP
  - antibody response components including for example anti-pTau and anti-Tau IgG and IgM titers
  - Inflammatory cytokines
  - AD biomarker assessment
  - ANA and anti-dsDNA testing
- Routine urine evaluation

3.5.2.5 *Visit 3 [Week 8]*

- AEs
- Concomitant medications (describe new and/or changes from the previous visit)
- Physical and neurological examination
- Vital signs before the administration of study medication
- Blood sample collected prior to administration of study medication for:
  - routine hematology and biochemistry evaluation, including CRP
  - antibody response components including for example anti-pTau and anti-Tau IgG and IgM titers
  - Inflammatory cytokines

AD biomarker assessment
ANA and anti-dsDNA testing

- Routine urine evaluation and urinary pregnancy test
- Administration of study medication
- 4-hour clinical observation:
  - Vital signs and observation of the injection site 1, 2 and 4 hours after the administration of study medication
  - Physical examination before discharge

3.5.2.6 Phone call 2 [week 8]

- AEs
- Concomitant medications (describe new and/or changes from the previous visit)
- In case of safety concerns, as per investigator assessment, an additional visit will be scheduled in order to further assess the event(s).

3.5.2.7 Visit 4 [Week 10]

This visit can take place within a period of up to 2 days.

- AEs
- Concomitant medications (describe new and/or changes from the previous visit)
- Physical and neurological examination
- Vital signs
- Blood sample collected for:

  routine hematology and biochemistry evaluation, including CRP
  antibody response components including for example anti-pTau and anti-Tau IgG and IgM titers
  Inflammatory cytokines
  AD biomarker assessment
  ANA and anti-dsDNA testing
- Routine urine evaluation
- Brain MRI

3.5.2.8 Visit 4.1 [Week 15]

This visit is only applicable in future sub-cohorts or in ongoing sub-cohorts, in subjects not having yet reached this visit timepoint. If feasible, agreed with subject and locally permitted, these visits may also be performed at subject's home.

- AEs
- Concomitant medications (describe new and/or changes from the previous visit)
- Blood samples collected for:

  antibody response components including for example anti-pTau and anti-Tau IgG and IgM titers
  AD biomarker assessment

3.5.2.9 Visit 4.2 [Week 20]

This visit is only applicable in future sub-cohorts or in ongoing sub-cohorts, in subjects not having yet reached this visit timepoint. If feasible, agreed with subject and locally permitted, these visits may also be performed at subject's home.

- AEs
- Concomitant medications (describe new and/or changes from the previous visit)
- Blood samples collected for:

antibody response components including for example anti-pTau and anti-Tau IgG and IgM titers
AD biomarker assessment

3.5.2.10 Visit 5 [Week 24]

- Assessment of the subject's decisional capacity: MacCAT-CR interview (in the Netherlands)
- AEs
- Concomitant medications (describe new and/or changes from the previous visit)
- Physical and neurological examination
- Vital signs before the administration of study medication
- Blood sample collected prior to administration of study medication for:
  routine hematology and biochemistry evaluation, including CRP
  antibody response components including for example anti-pTau and anti-Tau IgG and IgM titers
  coagulation indices assessment
  Inflammatory cytokines
  AD biomarker assessment
  ANA and anti-dsDNA testing
- Routine urine evaluation and urinary pregnancy test
- Administration of study medication
- 4-hour clinical observation:
  - Vital signs and observation of the injection site 1, 2 and 4 hours after the administration of study medication
  - Physical examination before discharge

Due to the Covid-19 pandemic, local travel restrictions in Finland effective as of 18 March 2020, and the need to preserve the safety of study participants, changes are made to the schedule of visits and procedures of sub-cohort 1.1 subjects:

- Given that the situation is not anticipated to be resolved to allow all procedures to be performed according to the protocol, Visit 5 will not be performed for any sub-cohort 1.1 subjects for whom the planned Visit 5 falls during the at-risk period.
- Instead, a remote visit will be performed in which the subject and caregiver are interviewed by telephone concerning any adverse events or changes in concomitant medications. Where possible, the NPI and C-SSRS scales will be administered during this interview. The remote visit will be performed at the initially planned Visit 5 date and according to the time-window defined in the Schedule of Assessments, i.e. at week 24 ± 7 days.
- This change is not applicable to the subject who had Visit 5 (week 24) procedures according to the protocol on 18 February 2020.

3.5.2.11 Phone call 3 [Week 24]

- AEs
- Concomitant medications (describe new and/or changes from the previous visit)
- In case of safety concerns, as per investigator assessment, an additional visit will be scheduled in order to further assess the event(s).

Due to the Covid-19 pandemic, local travel restrictions in Finland effective as of 18 March 2020, and the need to preserve the safety of study participants, changes are made to the schedule of visits and procedures of sub-cohort 1.1 subjects: Phone call 3 is not applicable in case Visit 5 and the associated immunization have not been performed.

3.5.2.12 Visit 6 [Week 26]

This visit can take place within a period of up to 2 days.

- AEs
- Concomitant medications (describe new and/or changes from the previous visit)
- Physical and neurological examination
- Vital signs
- Cognition: RBANS (form B)
- Clinical status: CDR, NPI, C-SSRS
- Blood sample collected for:
    - routine hematology and biochemistry evaluation, including CRP
    - antibody response components including for example anti-pTau and anti-Tau IgG and IgM titers
    - Inflammatory cytokines
    - AD biomarker assessment
    - T-Cell profile assessment
    - ANA and anti-dsDNA testing
- Lumbar puncture for cerebrospinal fluid sample (12 mL) for cell count, inflammatory markers and exploratory biomarker testing (note: blood for serum albumin should be taken and measured concomitantly)
- Routine urine evaluation
- Brain MRI
- ECG

Due to the Covid-19 pandemic, local travel restrictions in Finland effective as of 18 March 2020, and the need to preserve the safety of study participants, changes are made to the schedule of visits and procedures of sub-cohort 1.1 subjects:

- Given that the situation is not anticipated to be resolved to allow all procedures to be performed according to the protocol, Visit 6 will not be performed for any sub-cohort 1.1 subjects for whom the planned Visit 6 falls during the at-risk period.
- Instead, a remote visit will be performed in which the subject and caregiver are interviewed by telephone concerning any adverse events or changes in concomitant medications. Where possible, the CDR, NPI and C-SSRS scales will be administered during this interview. The remote visit will be performed at the initially planned Visit 6 date and according to the time-window defined in the Schedule of Assessments, i.e. at week 26 ± 2 days.
- This change is not applicable to the subject who had Visit 5 (week 24) procedures according to the protocol on 18 February 2020.

3.5.2.13 Visit 6.1 [Week 31]

This visit is only applicable in future sub-cohorts or in ongoing sub-cohorts, in subjects not having yet reached this visit timepoint. If feasible, agreed with subject and locally permitted, these visits may also be performed at subject’s home.

- AEs
- Concomitant medications (describe new and/or changes from the previous visit)
- Blood samples collected for:
    - antibody response components including for example anti-pTau and anti-Tau IgG and IgM titers
    - AD biomarker assessment

3.5.2.14 Visit 7 [Week 36]

- AEs
- Concomitant medications (describe new and/or changes from the previous visit)
- Physical and neurological examination
- Vital signs
- Blood sample collected for:
  - routine hematology and biochemistry evaluation, including CRP
  - antibody response components including for example anti-pTau and anti-Tau IgG and IgM titers
  - Inflammatory cytokines
  - AD biomarker assessment
  - ANA and anti-dsDNA testing
- Routine urine evaluation

Due to the Covid-19 pandemic, local travel restrictions in Finland effective as of 18 March 2020, and the need to preserve the safety of study participants, changes are made to the schedule of visits and procedures of sub-cohort 1.1 subjects:

- In case the situation is anticipated to not be resolved to allow all procedures to be performed according to the protocol, Visit 7 will not be performed. The decision will be taken on a case-by-case basis depending whether the visit for a particular subject is falling during the at-risk period.
- Instead, a remote visit will be performed in which the subject and caregiver are interviewed by telephone concerning any adverse events or changes in concomitant medications. Where possible, the NPI and C-SSRS scales will be administered during this interview. The remote visit will be performed at the initially planned Visit 7 date and according to the time-window defined in the Schedule of Assessments, i.e. at week 36 ± 3 days.

3.5.2.15 Visit 7.1 [Week 42]

This visit is only applicable in future sub-cohorts or in ongoing sub-cohorts, in subjects not having yet reached this visit timepoint. If feasible, agreed with subject and locally permitted, these visits may also be performed at subject's home.

- AEs
- Concomitant medications (describe new and/or changes from the previous visit)
- Blood samples collected for:
  - antibody response components including for example anti-pTau and anti-Tau IgG and IgM titers
  - AD biomarker assessment

3.5.2.16 Visit 8 [Week 48]

- Assessment of the subject's decisional capacity: MacCAT-CR interview (in the Netherlands)
- AEs
- Concomitant medications (describe new and/or changes from the previous visit)
- Physical and neurological examination
- Vital signs before the administration of study medication
- Blood sample collected prior to administration of study medication for:
  - routine hematology and biochemistry evaluation, including CRP
  - antibody response components including for example anti-pTau and anti-Tau IgG and IgM titers
  - coagulation indices assessment

Inflammatory cytokines
AD biomarker assessment
ANA and anti-dsDNA testing

- Routine urine evaluation and urinary pregnancy test
- Administration of study medication
- 4-hour clinical observation:
    - Vital signs and observation of the injection site 1, 2 and 4 hours after the administration of study medication
    - Physical examination before discharge

3.5.2.17 Phone call 4 [Week 48]

- AEs
- Concomitant medications (describe new and/or changes from the previous visit)
- In case of safety concerns, as per investigator assessment, an additional visit will be scheduled in order to further assess the event(s).

3.5.2.18 Visit 9 [Week 50]

This visit can take place within a period of up to 2 days.

- AEs
- Concomitant medications (describe new and/or changes from the previous visit)
- Physical and neurological examination
- Vital signs
- Cognition: RBANS (form A)
- Clinical status: CDR, NPI, C-SSRS
- Blood sample collected for:
    - routine hematology and biochemistry evaluation, including CRP
    - antibody response components including for example anti-pTau and anti-Tau IgG and IgM titers
    - Inflammatory cytokines
    - AD biomarker assessment
    - T-Cell profile assessment
    - ANA and anti-dsDNA testing
- Lumbar puncture for cerebrospinal fluid sample (12 mL) for cell count, inflammatory markers and exploratory biomarker testing (note: blood for serum albumin should be taken and measured concomitantly)
- Routine urine evaluation
- ECG
- Brain MRI

3.5.2.19 Visit 10 [Week 67]

- AEs
- Concomitant medications (describe new and/or changes from the previous visit)
- Physical and neurological examination
- Vital signs
- Blood sample collected for:
    - routine hematology and biochemistry evaluation, including CRP
    - antibody response components including for example anti-pTau and anti-Tau IgG and IgM titers
    - Inflammatory cytokines

AD biomarker assessment
ANA and anti-dsDNA testing

3.5.2.20 Visit 11 [Week 74]

This visit can take place within a period of up to 2 days.

- AEs
- Concomitant medications (describe new and/or changes from the previous visit)
- Physical and neurological examination
- Vital signs
- Cognition: RBANS (form B)
- Clinical status: CDR, NPI, C-SSRS
- Blood sample collected for:
  - routine hematology and biochemistry evaluation, including CRP
  - antibody response components including for example anti-pTau and anti-Tau IgG and IgM titers
  - Inflammatory cytokines
  - AD biomarker assessment
  - ANA and anti-dsDNA testing
- ECG
- Brain MRI

3.5.2.21 Visit 12 [Week 86] (Optional)

This visit would be applicable for future or ongoing sub-cohorts where the sponsor is interested in extending the post-injection follow-up period and where subjects have not yet passed study visit 12 (week 86). Prior to any assessment described below, written consent of subjects will be obtained, and in the Netherlands, the subjects' decisional capacity will be re-assessed before consenting.

If feasible, after subject consent and if locally permitted, this visit may also be performed at subject's home.

- AEs
- Concomitant medications (describe new and/or changes from the previous visit)
- Blood samples collected for:
  - antibody response components including for example anti-pTau and anti-Tau IgG and IgM titers
  - AD biomarker assessments

3.5.2.22 Visit 13 [Week 98] (Optional)

This visit would be applicable for sub-cohorts where the post-injection follow-up period has been extended. Prior to any assessment described below, written consent of subjects will have been obtained, and in the Netherlands, the subjects' decisional capacity will have been re-assessed before consenting.

If feasible, after subject consent and if locally permitted, these visits may also be performed at subject's home.

- AEs
- Concomitant medications (describe new and/or changes from the previous visit)
- Blood samples collected for:
  - antibody response components including for example anti-pTau and anti-Tau IgG and IgM titers
  - AD biomarker assessments

3.5.2.23 Unscheduled Visit (UV)

At any time during the study, subjects might be invited to the site for additional visits (e.g. further evaluation needed following a phone call with the subject). In case of safety concerns, as per investigator assessment during a scheduled “Phone call” or a visit of the post-injection period extension (if applicable) with the subject, an additional visit will be scheduled in order to further assess the event(s). Any subjects attending an unscheduled visit should at a minimum complete the following safety related assessments:

- Provide information on new AEs/SAEs since last visit.
- Provide information on new concomitant medication since last visit.
- Be examined for the Vital signs
- Go through physical and neurological examination
- Have blood withdrawn for standard safety evaluation

Upon investigator’s medical judgement, other assessments considered as needed can be performed during those visits (MRI, ECG, etc.).

Due to the resurgence of the pandemic in multiple participating countries since September 2020 and in case the subject and caregiver cannot travel to the site due to Covid-19 restriction, the Unscheduled Visit and related assessments will be performed at subject’s home if feasible and locally permitted (see details under 3.5.1.1 Alternative Mode of Administration of Assessments to Reduce Safety Risks due to Participation during Covid-19 pandemic).

3.5.3 Assessments for Premature Termination

Any subject, who withdraws from the study prior to completion (see standard categories of early termination under section 3.3.3), will be encouraged to at least complete the safety assessment evaluation (see detail under 3.5.2.21 Unscheduled Visit) by attending a last visit.

If this is not possible, subject will be asked to at a minimum, provide the reason for withdrawal and provide information on any adverse events that might have arisen since the last visit and the outcome of any previously recorded AEs.

3.5.4 Safety/Tolerability Assessments

Safety monitoring after administration of study medication

All subjects will be kept under clinical observation for 24 hours after the first administration of study vaccine and for 4 hours after subsequent administrations of study vaccine (second, third and fourth administrations). During that period, the vital signs will be monitored and the injection site will be observed on regular basis. A physical examination will be performed prior to discharge. The clinical observation may be prolonged in case of safety concerns according to the clinical judgement of the investigator.

A subsequent safety assessment will also be performed 48 to 72 hours after each immunization by telephone call for all study subjects. An unscheduled visit should be proposed in case of moderate or severe suspected adverse reactions reported by telephone assessment, according to clinical judgement.

Physical and Neurological Examination

Physical examination will include assessment of general appearance, the head, eyes, ears, nose, throat, heart, chest, lungs, abdomen, lymph nodes, extremities, peripheral pulses, skin and any other physical conditions of note. Weight will be recorded in kg and height (first attempt) in cm. The neurological examination will include examination of the cranial nerves, upper and lower

extremities for muscle strength, reflexes, sensation and cerebellar function. Examinations will be performed at each visit at the center.

Vital Signs

Vital signs will be measured at each visit. During dosing visits, vital signs (blood pressure, heart rate, respiratory rate and temperature) will be measured before and after the administration of study vaccine. At V1 (baseline), measurements will be made just before the first injection (time 0) and at the following time after the injection at 1, 2, 4, 8, 12, and 24 hours. On subsequent dosing visits (V3, V5*, V8), measurements will be done at time 0, 1, 2, and 4 hours. *The vital signs assessment does not apply in case V5 is performed remotely in sub-cohort 1.1 subjects (refer to section 3.5.2.10).

Blood pressure and heart rate will be measured in the sitting and standing positions at the screening visit and at time 0 of V1 (baseline). The sitting blood pressure should be measured first and should be measured after the subject has been sitting down for at least 5 minutes. The subject should then be asked to stand and the standing blood pressure should be measured after the subject has been standing for 2 minutes. At all other time points for V1 or for all other visits, blood pressure and heart rate will be measured in the sitting position only.

Blood pressures will be measured with a standardized mercury manometer; alternative validated methods of measurement may also be used. The point of disappearance of Korotkoff sounds (phase V) will be recorded as the diastolic blood pressure where a sphygmomanometer is used.

Heart rate will be determined over 60 seconds following the recording of blood pressure in the corresponding position.

Body temperature will be measured and recorded.

Pregnancy Testing

In women of child bearing potential, blood pregnancy tests will be performed at the screening visit (blood draw between day -14 and day -3 prior to baseline). Subsequently, pregnancy tests from urine will be performed prior to the dosing. The test strips will be provided by the Central Laboratory.

Electrocardiogram (ECG)

12-lead ECG recording machines will be provided by the central reading company. Printouts of each ECG must be kept at the site for archiving. The central reading company will manage ECG data through their Web-based platform. The system allows the acquisition, storage and display of ECG recordings from the ECG machines so that cardiologists can review them. A manual describing all methods for collecting ECG data will be provided to the Investigator.

Magnetic Resonance Imaging (MRI)

Brain MRI scans will be conducted according to the schedule of assessments and examined for evidence of encephalitis and other brain pathology. Scans will be systematically analyzed e.g. for the presence of micro hemorrhages or vasogenic edema by the Central reading company. A manual describing all methods for collecting MRI data will be provided to the site.

Safety Blood and Urine Analysis

Tests for safety blood and urine analysis will be performed at the central laboratory. Details regarding handling, storage and shipment of the safety blood and urine samples will be provided to the study centers in writing (see Laboratory Investigator Manual).

Hematology

Safety hematological analysis will be performed at all visits. Analyses will include: red blood cell count, hemoglobin, hematocrit, red cell indices, white blood cell count (including differential), platelet count.

Coagulation indices (PT (INR)/aPTT) will be measured at the Screening Visit and at visits preceding the visits where the lumbar punctures take place.

Biochemistry

Safety biochemistry will be performed at all visits. Analyses will include:

Sodium, potassium, chloride, urea, creatinine, calcium, inorganic phosphate, glucose, total bilirubin, total protein, albumin, aspartate aminotransferase, alanine aminotransferase, alkaline phosphatase, Gamma GT, creatine kinase, cholesterol, triglycerides and uric acid and CRP.

Diagnosis of auto-immune diseases

Anti-Nuclear Antibody (ANA) and anti-double stranded DNA (anti-dsDNA) antibody titers will be tested at all visits. At dosing visits, the blood sample will be collected prior to injection of the study vaccine. A detailed assessment for lupus or other rheumatologic disease will be performed if the anti-ds DNA antibody test is positive. Subjects who have a positive anti-dsDNA antibody result and associated clinical symptoms will be discontinued from further immunizations but will be followed for safety. A DSMB review will be held to determine further immunizations on the study.

Safety Urine Analysis

Safety urine analysis will be performed at visits indicated in the schedule of assessments (section 3.5.1). Analyses will include: pH, protein, glucose, ketones and blood.

Inflammatory Markers in CSF

CSF samples will be obtained (as per the schedule of assessments of section 3.5.1) using standard lumbar puncture procedures performed by a physician experienced in the procedure who will ensure that the subject receives appropriate medical care after the procedure has been performed. Materials for and details regarding collecting and processing CSF samples will be provided to the investigator. CSF samples will be sent to Central Laboratory according to provided instructions.

The lumbar puncture which is conducted at screening should be performed at least 3 days prior to the administration of the study medication. This is to reduce the chance that any post-lumbar puncture headache might interfere with the assessment of adverse events occurring after administration of the study medication. The CSF samples will be examined for evidence of inflammation including:

- Protein
- CSF/serum albumin ratio
- IgG + IgM index
- Oligoclonal bands
- Glucose
- Cell Count and differential count when applicable (to be performed at the local site laboratory)

Screening Tests

The following tests will be performed on serum at screening visit only as part of work-up to rule out other causes of cognitive impairment: VDRL (Syphilis serology), Vitamin B12 and folate, Thyroid function tests (T4, T3, TSH), HIV. In addition Hepatitis B and C testing will be conducted.

Adverse Events

All AEs occurring after the subject signs the ICF and up to the last safety follow-up visit (V11 Week 74) or up to Visit 13 (Week 98) in case the post-injection period is extended, will be recorded.

See Section 4 for additional information.

Cognitive tests

The cognitive tests are used either to verify eligibility criteria or as part of the safety assessments to verify there is no impairment of subject cognitive status that could be related to the study vaccine. The tests have been selected as suitable for the degree of cognitive impairment in eligible subjects. Tests should be performed by a trained rater, who except for the cognitive rating activities is not involved in any other study-related activities. The rater should be a psychologist or other site personnel, experienced on psychometric tests, who should neither perform nor have access to any other subject clinical assessments during the course of the study. An experienced and adequately trained back-up rater should exist. Training on cognitive tests will be provided by [redacted].

Whenever possible the cognitive tests should be performed at the same time of day throughout the study for each individual subject, i.e. ideally within a ± 2 hours-time window.

MMSE

The Mini Mental State Examination (MMSE) (Folstein 1975) is a widely used test of overall cognitive function, assessing memory, orientation and praxis in a short series of tests. The score is from 0 to 30 with 30 being the best possible and 0 being the worst possible score. The test will only be performed at the screening visit to confirm subject's eligibility to participate.

RBANS

The Repeatable Battery for the Assessment of Neuropsychological Status (RBANS) (Randolph, 1998) is a test designed to measure cognitive decline or improvement in a short series of 12 subtests that measure attention, language, visuospatial/constructional abilities, and immediate and delayed memory. The test yields 5 Index scores and a Total Scale score. Normative information from the test manual is used to calculate the Index and Total scores. Three versions of the scale are used during the study: form A (at V1 and V9), form B (at V6 and V11) and form C (at Screening).

Clinical rating scales

The clinical rating tests are used as part of the safety assessments to verify there is no impairment of subject clinical status that could be related to the study vaccine. The clinical rating scales should be performed by a trained clinical rater (experienced site personnel). The clinical rater should not perform the cognitive tests. Training on clinical tests will be provided by [redacted].

C-SSRS

Recent guidance from the FDA has indicated the importance of collecting data on potential induction of suicidal behavior with all new agents acting on the central nervous system. Although no such effects are anticipated with the study vaccines investigated, this will be directly evaluated with the Columbia-Suicide Severity Rating Scale (C-SSRS) at several

time points during the study. Questions related to suicidal behavior or ideation are directly asked by the rater to the subject. Two versions of the questionnaire are used during the study: Baseline/Screening version (at Screening) and Since Last Visit Version (at V1).

CDR

The Clinical Dementia Rating Scale (Hughes et al. 1982) is a global rating of the function of AD subjects assessed in six categories: memory, orientation, judgement and problem solving, community affairs, home and hobbies and personal care. It is based on a semi-structured interview conducted with the subject and caregiver, by a rater without access to the results of the cognitive tests described above. Each category has scores from 0 (no symptoms) to 3 (severe) and the sum of these items (sum of boxes) may therefore range from 0 to 18 points. The CDR global score is derived from scores of each individual category and will be used as part of the selection criteria (score of 0.5 or 1 at Screening).

NPI

The Neuropsychiatric Inventory (NPI) is a 12-item scale which assesses behavioral disturbances commonly occurring in dementia subjects. Twelve behavioral areas are included in the NPI: delusions, hallucinations, agitation/aggression, depression/dysphoria, anxiety, elation/euphoria, apathy/indifference, disinhibition, irritability/lability, aberrant motor behavior, night-time behaviors, and appetite/eating changes. Through a structured interview with the caregiver, each of these 12 domains will be assessed. Both the frequency (0-4) and severity (0-3) of each domain will be determined and multiplied together and summed across domains for a total score of 0-144; a higher score indicates more severe psychopathology.

3.5.5 Primary and Secondary Immune Response Assessments

At all visits (including visits in the extension of the post-injection period, if applicable), blood will be collected for determination of the immune response in serum, i.e. anti-pTau and anti-Tau IgG and IgM titers.

Serum samples after conduct of the per-protocol performed analyses will be stored appropriately for up to 10 years after the end of the study and if necessary used for additional analyses with other immunogenicity assays.

3.5.6 Exploratory assessments

T cell activation

Samples of baseline V1, V6 and V9 will be assessed by ELISPOT analysis.
Remaining unused aliquots of PBMC samples after conduct of the per-protocol performed analyses will be stored appropriately for up to 10 years after the end of the study and if necessary used for additional analyses with other immunogenicity assays.

Inflammatory cytokines

At all visits, blood will be collected for assessment of the inflammatory cytokines (e.g. IL-1β, IL-2, IL-6; IL-8, IL-10, IFN-γ and TNF-α).

Biomarkers in CSF and Plasma

The following putative AD biomarkers will be assessed in CSF and/or in plasma:

- Total tau, pTau, Tau fragments
- Other biomarkers of relevance

Blood samples for AD biomarker testing will be collected at all visits except screening (including visits in the extension of the post-injection period, if applicable).

CSF samples will be collected at Screening, V6 and V9.

CSF and plasma samples after conduct of the per-protocol performed analyses will be stored appropriately for up to 10 years after the end of the study and if necessary used for additional analyses with other related biomarker assays.

Immune response

At all visits (including visits in the extension of the post-injection period, if applicable), blood will be collected for further exploring the immune response in serum. e.g. antibodies against vaccine components, functional capacity of vaccine-induced antibodies).

Serum samples after conduct of the per-protocol performed analyses will be stored appropriately for up to 10 years after the end of the study and if necessary used for additional analyses with other immunogenicity assays.

Clinical rating scales and cognitive tests

The cognitive and clinical assessments described in section 3.5.4 as safety parameters will also be examined for any preliminary evidence of efficacy, taking into account that the study is not powered for statistical comparisons.

Subject capacity to consent: MacCAT-CR

In the Netherlands, the subject's decisional capacity will be assessed by using the MacArthur Competence Assessment Tool for Clinical Research (MacCAT-CR). The MacCAT-CR assesses the subject capacities to consent to research participation (Appelbaum and Grisso, 2001). It is a 21-item structured interview with four subscales assessing the following four main dimensions of decision making: understanding, appreciation, reasoning, and expressing a choice. Questions are tailored to the research context defined in this protocol. The determination that the subject is able to give informed consent and is able to maintain his/her decisional capabilities during the study will be based on the subject's responses to the questions in conjunction with the Investigator's clinical judgement. The rater must be an investigator or delegate adequately trained.

The MacCAT-CR interview will be performed at study start prior to signing the informed consent form and subsequently at V5 and V8, and before consenting to the extension of the post-injection period. In case it is determined that the subject is no longer able to maintain his/her decisional capacity, participation should be discontinued.

4. SAFETY PROCEDURES

Throughout the course of the study, all AEs will be monitored and recorded on an AE eCRF, including the AE's description, start and end date, seriousness, severity, action taken, and relationship to the investigational product. If AEs occur, the first concern will be the safety of the study subjects. All AEs will be followed until resolved or stable and the outcome documented on the eCRFs. While not considered an AE per se, pregnancies will also be reported in an expedited manner like AEs.

For the purpose of safety reporting, the trial period is defined as the interval between the signature of the informed consent by the subject and the end of the designated follow-up period, or the end of the post-injection period extension, if applicable (or last visit/assessment in case of early termination).

4.1 Definitions and Criteria

4.1.1 Adverse Events

An adverse event (AE) is any untoward medical occurrence in a subject or clinical investigation subject administered a pharmaceutical product and which does not necessarily have a causal relationship with this treatment.

An AE can therefore be any unfavorable and unintended sign (including an abnormal laboratory finding), symptom or disease temporally associated with the use of a medicinal (investigational) product, whether or not related to the medicinal (investigational) product.

Pre-existing conditions which worsen during the study, exacerbation of a pre-existing illness or increase in frequency or intensity of a pre-existing episodic event or condition are to be reported as AEs. A condition detected or diagnosed after the informed consent signature even though it may have been present prior to the start of the study should also be reported as an AE.

4.1.2 Serious Adverse Event

A Serious Adverse Event (SAE) is any AE occurring at any dose that results in any of the following outcomes:

- Death (Note: Death is an outcome, not an event)
- Life-threatening (Note: life-threatening refers to an event in which the subject was at risk of death at the time of the event; it does not refer to an event which could hypothetically have caused death had it been more severe)
- Inpatient hospitalization or prolongation of an existing hospitalization (Note: "inpatient hospitalization" refers to an unplanned, overnight hospitalization)
- Persistent or significant disability / incapacity
- Congenital anomaly / birth defect
- Important medical event (an event not meeting the above criteria but deemed by the investigator) to be one that may jeopardize the subject or may require intervention to prevent one of the other outcomes listed above (e.g. intensive treatment in an emergency room or at home for allergic bronchospasm or blood dyscrasias or convulsions that do not result in hospitalization).

4.1.2.1 Clarifications

Hospitalization for elective treatment of a pre-existing condition that did not worsen during the study is not considered an SAE.

Complications that occur during hospitalization are AEs. If a complication prolongs hospitalization, or meets any of the other SAE criteria, then the event is an SAE.

4.1.3 Unexpected Adverse Drug Reactions

An unexpected adverse drug reaction (ADR) is a reaction for which the nature or severity is not consistent with the applicable reference safety information (e.g. Investigator's Brochure).

All noxious and unintended responses to a medicinal product related to any dose should be considered an adverse drug reaction. The phrase "responses to a medicinal product" means that a causal relationship between a medicinal product and an AE is at least a reasonable probability, i.e. the relationship or probability cannot be ruled out.

4.1.4 Abnormal Laboratory Values

Abnormal laboratory findings and other abnormal assessments that are judged by the investigator as clinically significant must be recorded as AEs or SAEs if they meet the definition of an AE, as defined in section 4.1.1, or an SAE, as defined in section 4.1.2. Clinically significant abnormal laboratory findings or other abnormal assessments that are detected after study drug administration or that are present at baseline and worsen following the start of the study are included as AEs or SAEs. The investigator should exercise medical and scientific judgement in deciding whether an abnormal laboratory finding or other abnormal assessment is clinically significant.

4.1.5 Assessing Intensity and Relationship

Intensity

All AEs must be rated on a 3-point scale of increasing severity using the following definitions:

MILD	An adverse event that is easily tolerated by the subject causes minimal discomfort and does not interfere with everyday activities.
MODERATE	An adverse event that is sufficiently discomforting to interfere with normal everyday activities; intervention may be needed. Event is not hazardous to the subject's health.
SEVERE	An adverse event that prevents normal everyday activities; treatment or other intervention usually needed. Event is a definite hazard to the subject's health.

Relationship

The investigator should make an assessment of whether the AE is likely to be related to the IMP according to the following definitions

UNRELATED	A relationship to the study vaccine can be definitely ruled out (reasonable explanation must be given, e.g. involved in traffic accident while in back seat of car).
UNLIKELY RELATED	A relationship to the study vaccine is considered unlikely: the time relationship to the administration of study vaccine does not suggest a causal relationship and/or the underlying disease, other concomitant illnesses or medications appear more likely explanations according to present knowledge.
POSSIBLY RELATED	There is a reasonable possibility that the adverse event may have been caused by the study vaccine: there is a reasonable time relationship to the administration of study vaccine but the nature of the event, the underlying disease, and/or concomitant medication or concomitant illnesses suggest that other explanations are a significant possibility.

PROBABLY RELATED	The study vaccine is considered to be the most likely cause of the adverse event: there is a reasonable time relationship to the administration of the study vaccine and the event is considered unlikely to be attributed to concurrent disease or concomitant medications.

All AEs assessed as having a reasonable suspected causal relationship to the study medication (i.e., possibly, probably) will be considered as related to the study medication for regulatory reporting purposes.

4.1.6 Monitoring and Management of Adverse Events Including Specific Risks

Adverse events should be managed using adequate measures and symptomatic treatment as needed as there is no specific antidote to ACI-35.030 and JACI-35.054.

Skin reactions at the injection site should be managed symptomatically. In case of local reaction, it is recommended to use the alternate arm for the subsequent injection.

Allergic and anaphylactic reactions should be treated according to standard medical practice.

Signs and symptoms of neurological adverse events (e.g. headache, confusion, motor and/or sensitive deficits) must be assessed in every clinical visit. As per the study protocol, MRI scans should be reviewed carefully for abnormalities. Subjects should be regularly reminded to contact the site without delay if they experience significant adverse events. An unscheduled visit should be proposed in case of moderate or severe suspected adverse reactions reported by telephone assessment, according to clinical judgement.

In suspected cases of meningoencephalitis, patients should be investigated as deemed clinically appropriate, e.g. by neurological/psychiatric examination as well as CSF sampling, MRI scanning and electroencephalography (EEG). In confirmed cases related to the study vaccine, further injections of study vaccine should be withheld, and appropriate treatment such as intravenous corticosteroids should be administered according to clinical judgement. If appropriate, consideration may be given to the use of plasmapheresis which was reported to be effective in one of 2 subjects with meningoencephalitis secondary to the anti-amyloid vaccine AN1792 not responding to steroids (Orgogozo et al, 2003). Complications such as convulsions should be managed according to standard medical practice.

4.2 Reporting Procedures and Requirements

4.2.1 Adverse Events

If several signs, symptoms or diagnostic abnormalities are clearly related to a medically defined diagnosis or syndrome, the diagnosis should be reported on the AE pages in the eCRF. All clearly related signs, symptoms and abnormal diagnostic procedures should be grouped together as a single diagnosis. Grouping into a medical diagnosis should only be done if every component sign and symptom is a medically and clearly acknowledged component of the diagnosis by standard textbooks of medicine. If any aspect of the signs or symptoms does not fit into a classic pattern of the diagnosis or syndrome, a separate AE should be reported for each such sign or symptom.

A diary will be given to subjects as a memory aid for recording adverse events and concomitant medication changes occurring during the study conduct. It will be kept with the help of the caregiver. Subject diaries should be reviewed by site personnel at each study visit.

All AEs during the trial will be recorded in the eCRF. For each sign, symptom or diagnosis, the Investigator will provide the following information: Type of event, date initiated/observed, severity,

action taken, seriousness, date stopped, outcome and relation to investigational medicinal product.

4.2.2 Serious Adverse Events

The Sponsor delegates to [redacted] the reporting of pharmacovigilance information and data entry into the EudraVigilance database. The investigators report to the CRO's Safety Officer who will keep the Sponsor informed. This reporting routine will be described in a written procedure prior to start of screening.

The SAE must be reported immediately by the investigator, within one calendar day (24 hours) from time of awareness by telephone or fax to the Safety Officer. Any follow-up data will be detailed in a subsequent SAE form, which also must be reported to the Safety Officer within one calendar day (24 hours) from time of awareness. Study medication errors, including overdoses must be reported in an expedited manner like SAEs, even in the absence of associated clinical symptoms.

All safety related information will be collected and processed promptly, to comply with regulatory requirements.

All AEs that meet the criteria for SAEs, study medication errors, including overdoses require a completion of a SAE Report Form. All SAEs must also be recorded on the AE pages in the eCRF. AEs (including SAEs) will be collected and recorded from the time the subject signs the Informed Consent Form until the last follow-up visit.

Follow-up:

a) The investigator should take all appropriate measures to ensure the safety of the subjects, notably he/she should follow up the outcome of any SAEs (clinical signs, laboratory values or other etc.) until the return to normal or until consolidation of the subject's condition.

b) In case of any SAE, the subject must be followed up until clinical recovery is complete and laboratory results have returned to normal, or until progression has stabilized. This may imply that follow-up will continue after the subject has left the trial and that additional investigations may be requested by the Sponsor.

c) In case of any SAE brought to the attention of the investigator at any time after cessation of investigational medicinal product and considered by him/her caused by the trial treatment with a reasonable possibility, this should be reported to the Sponsor.

d) The treatment code must be broken only in exceptional circumstances when knowledge of the investigational medicinal product is essential for treating the subject.

e) The Safety Officer identified below will report SAEs to the Competent Authorities and Ethical Committees on behalf of the Sponsor according to the applicable regulatory requirements.

Suspected Unexpected Serious Adverse Reactions (SUSARs) will be notified by the Safety Officer to the Competent Authorities and Ethics Committee (EC) in an expedited manner as follows: fatal or life- threatening SUSARs – as soon as possible but no later than 7 calendar days after receipt of the minimum criteria for expedited reporting; non-fatal and non-life-threatening SUSARs - as soon as possible but no later than 15 calendar days after receipt of the minimum criteria for expedited reporting. Relevant follow- up information for fatal or life-threatening SUSARs will be provided to the Competent Authorities and EC(s) within an additional 8 calendar days. The IB will be used as the reference safety information for the determination of the expectedness of the SAE.

Safety Officer:

[redacted]

4.2.3 Contraceptive measures and procedures for documenting pregnancy during the study

4.2.3.1 Definitions

The following definitions are derived from the Head of Medicine Agencies Clinical Trial Facilitation Group (Recommendations related to contraception and pregnancy testing in clinical trials, 2014).

A woman is considered of childbearing potential (WOCBP) if fertile, following menarche and until becoming post-menopausal unless permanently sterile. Permanent sterilization methods include hysterectomy, bilateral salpingectomy and bilateral oophorectomy. A postmenopausal state is defined as no menses for 12 months without an alternative medical cause. A high follicle stimulating hormone (FSH) level in the postmenopausal range may be used to confirm a post-menopausal state in women not using hormonal contraception or hormonal replacement therapy. However in the absence of 12 months of amenorrhea, a single FSH measurement is insufficient.

A man is considered fertile after puberty unless permanently sterile by bilateral orchidectomy.

An effective contraceptive is a method of birth control which can achieve a low failure rate (i.e., less than 1 % per year) when used consistently and correctly. Such methods include:

- Oral, intravaginal or transdermal combined (estrogen and progestogen containing) hormonal contraception associated with inhibition of ovulation
- Oral, injectable or implantable progestogen-only hormonal contraception associated with inhibition of ovulation
- Intrauterine device (IUD)
- Intrauterine hormone-releasing system (IUS)
- Bilateral tubal occlusion
- Vasectomized partner
- Sexual abstinence

Vasectomized partner is a highly effective birth control method provided that partner is the sole sexual partner of the WOCBP trial participant and that the vasectomized partner has received medical assessment of the surgical success.

True sexual abstinence i.e. refraining from heterosexual intercourse during the entire study duration will only be considered as a highly effective method when this is in line with the preferred and usual lifestyle of the subject. Periodic abstinence (e.g., calendar, ovulation, symptothermal, post-ovulation methods), declaration of abstinence for the duration of exposure to IMP, and withdrawal are not acceptable methods of contraception.

4.2.3.2 Procedures

Pregnant women, women who plan to become pregnant, or women currently nursing an infant may not take part in this study. WOCBP must be willing to use highly effective methods of contraception from the screening visit until the end of their participation. WOCBP will be asked to have a blood pregnancy test at screening (blood draw between day -14 and day -3 prior to baseline), and a confirmation of the non-pregnancy status will be reassessed via urine pregnancy

testing before the dosing to exclude the possibility of pregnancy. Women who could become pregnant must use an effective contraceptive during the course of this study.

Any woman who finds that she has become pregnant while taking part in the study including during the SAE follow-up period should immediately contact the Investigator who should notify the Safety Officer without delay. Women who become pregnant during the study will be withdrawn from the study and no further immunizations given. While not a SAE, pregnancy must be reported as an SAE for monitoring and follow-up. Pregnancy should be followed up until delivery, and the Safety Officer informed of the outcome.

Male participants in the trial with female partner of child bearing potential will be informed about the potential risk of the treatment on the partner's pregnancy and are required to use barrier methods of contraception (condoms with spermicide) in addition to contraceptive measures used by female partners during the whole study duration. In case a female partner becomes pregnant during the subject's participation in the study, pregnancy will be reported and followed up until delivery, and the Safety Officer informed of the outcome.

4.2.4 Stopping Rules

4.2.4.1 Stopping at the Individual Subject Level

Dosing will be suspended at the individual level if a SAE considered related to study medication is observed. Dosing will also be suspended at the individual level in the case of adverse reactions of moderate intensity persisting for more than 2 weeks or of severe intensity until further evaluation by the DSMB. Dosing may also be suspended in the case of adverse reactions of shorter duration or lower intensity if the investigator considers this clinically indicated.

Dosing may also be suspended in the case safety assessments cannot be performed during the Covid-19 pandemic and/or are exposing subjects to an increased risk of infection with Covid-19 (refer to section 1.5.2. Risks of Participation during Covid-19 Pandemic).

4.2.4.2 Stopping or Suspension of Dosing

Dosing will be suspended in all subjects if meningoencephalitis is observed in any subject, until further evaluation by the DSMB. The Competent Authority in each participating country will be informed of such a temporary halt to dosing following a case of meningoencephalitis. Dosing will not recommence until a protocol substantial amendment is submitted to the Competent Authorities requesting trial restart.

All SAEs will be reported to the DSMB who will evaluate them and give recommendations whether dosing in the cohort as a whole can continue. Where two or more clinically relevant non-serious adverse reactions of moderate intensity persisting for more than 2 weeks or severe intensity are observed, this will be drawn to the attention of the DSMB who will evaluate the cases and determine whether dosing can continue in the cohort as a whole.

5. DATA MANAGEMENT AND STATISTICAL ANALYSIS

5.1 Data Management Considerations

The database is set up and managed by [redacted] and hosted in a secure web environment by software vendor Perceptive Informatics. The database system used is DataLabs which is a software designed by Perceptive Informatics and programmed by [redacted] to provide applications for data management, data collection, data validation and storage of clinical trial data. The system is in compliance with industry regulations, including FDA 21 Code of Federal Regulations (CFR) Part 11.

The study is run as an EDC-study (Electronic Data Capture), i.e. all relevant data are entered by the sites directly into the clinical database under the supervision of the site investigator.

The subjects enrolled into the trial will be identified in the database by a unique subject number.

Subject data should be entered into the eCRF by authorized site staff in a timely manner. After data entry systematic data validation will be performed and any data discrepancies will be presented electronically to the site staff through the DataLabs.

Queries for discrepant data may be generated automatically by the software upon entry or generated manually by the monitor or the trial data manager. All queries, whether generated by the system or by trial staff, will be in electronic format.

The systematic data validation will provide a clean and consistent database prior to the statistical analysis. Data will be processed in accordance with the general terms and conditions of the EU's General Data Protection Regulation and any applicable national legislation.

Handling of data entered into the eCRF and other external data such as laboratory data will be specified in the Data Management Plan.

Data Management will be performed according to current [redacted] standard operating procedures. The investigator is responsible for ensuring the accuracy, completeness, legibility, and timeliness of the data reported. All source documents should be completed in a neat, legible manner to ensure accurate interpretation of data. Data recorded in the electronic case report form (eCRF) derived from source documents should be consistent with the data recorded on the source documents.

5.2 Electronic CRFs

DataLabs will be used for data collection. Following training, trial site staff will be given access to the software. Access to the software is restricted to trial staff participating in the trial and the extent of access will depend on the staff members' user role in the trial.

Data recorded in the eCRFs will be accessible to trial staff through a secure internet connection immediately after entry.

The investigator or staff authorized by the investigator will enter subject data into electronic eCRFs. The eCRFs must be maintained in an up-to-date condition at all times by the investigator or designee. The investigator, or co-investigator(s) authorized by the investigator, will sign electronically all sections of eCRFs used. This signature information (including date of signature) will be kept in the audit trail and is unalterable. Only medically qualified (co)investigators can sign data on clinical assessments/safety.

Any correction(s) made by the investigator, or authorized site staff, to the eCRF after original entry will be documented in the audit trail. Changes to data already approved require the re-

signature of investigator or authorized staff. The audit trail will identify the person making the change and the date, time and reason for the change.

The trial monitor will check the eCRFs for accuracy and completion and perform source data verification (SDV). The trial monitor will document electronically SDV of all sections of eCRFs used.

For archiving purposes, each investigator will be supplied with a copy of the eCRFs, for all subjects enrolled at the site, via an electronic medium at completion of the trial. Audit trail information will be included.

The eCRFs will be available for inspection by authorized representatives from Sponsor and from Regulatory Authorities and/or EC.

5.3 Statistical Considerations

Statistical analysis will be performed by [redacted] and by [redacted]. The presentation and analysis of data will be detailed in a Statistical Analysis Plan (SAP) that will be approved by AC Immune.

5.3.1 Sample Size Justification

Antibody titer assessment: The preliminary assessment of the antibody response and of the safety/tolerability (comparison between sub-cohorts) will be performed based on the data of all randomized subjects (6 on ACI-35.030/JACI-35.054 and 2 on placebo per sub-cohort) based on the timepoints related to the different IAs that will be conducted during the study. The initial number of subjects in each sub-cohort is consistent with the customary sample size employed in this type of studies. It is expected to allow clinical judgement of safety and tolerability, and to get preliminary evidence of an acceptable immune response. This sample size is also anticipated to provide sufficient evidence to support the possible enrollment of up to 16 additional subjects in the sub-cohort(s) showing the most favorable antibody response profile(s) with the objective to confirm the initial results. The number of additional subjects to be enrolled will be determined based on the preliminary observed immunogenicity response rate in the sub-cohort(s) and on the potential gain in precision of the response rate estimate in the expanded sub-cohort(s), and will not exceed 16 (12 on ACI-35.030/JACI-35.054 and 4 on placebo per sub-cohort). In addition, all other immunogenicity endpoints available at the interim timepoint may be considered in the sample size re-estimation. This may include but is not limited to the observed variability of titers such as the Anti-pTau IgG and the Anti-e-PHF IgG.

In total, up to 24 subjects can be randomized to cohort 1, cohort 2 and cohort 3, respectively (not taking into account sub-cohort(s) expansion). Any randomized subject withdrawing before the 2nd injection for reasons other than safety may be replaced in the cohort.

For safety, if an AE occurs at the rate of 1% or 10%, then the chance of observing such an AE among 6 subjects receiving that dose of active treatment will be 6% or 47%, respectively. If no AE of a given type is observed in any of the 6 subjects at a given dose of active treatment, then with 80% (90%) confidence the true incidence of that type of AE at that dose is at most 24% (32%). An optional recruitment expansion of up to 16 additional subjects may be considered in any sub-cohort to allow further assessment of safety at that dose.

5.3.2 Analysis Population

The intention-to-treat (ITT) analysis set is defined as all randomized subjects who received at least one dose of the drug during the study.

The ITT analysis set will be used for presentation of primary, secondary and exploratory endpoints and for description of baseline characteristics.

The per-protocol analysis set (PP) is defined as all subjects from the ITT set who do not have any major protocol deviations.

Major protocol deviations will be defined prior to database lock. In any case, withdrawals from the study prior to completion either during the treatment period or during the safety follow-up period are considered major protocol deviations.

The PP analysis set will be used as a supportive analysis of the primary endpoints.

The definition of the safety analysis set, which will be used for analysis of safety data, is identical to the ITT analysis set.

5.3.3 Protocol Deviations

Protocol deviations should be collected by site and grouped into different categories, such as:

- Subjects who enter the study even though they did not satisfy the entry criteria
- Those who developed withdrawal criteria during the study but were not withdrawn
- Those who received the wrong treatment or incorrect dose
- Those who received any excluded concomitant treatment

Major protocol violations will lead to exclusion from the PP analysis set.

5.3.4 Demographic and Baseline Characteristics

Dose groups will be compared with respect to subject demographics and baseline characteristics will be summarized using descriptive statistics. No formal statistical analysis tests will be performed.

Placebo subjects can be pooled over different (sub-)cohorts for presentation, where appropriate.

5.3.5 Safety Analyses

The primary safety endpoints are type, frequency and intensity of AEs evaluated until the end of treatment and change from baseline in the parameters of physical and neurological examination, vital signs, MRI imaging, ECG, routine hematology and biochemistry evaluation in blood and urine, evaluation of autoimmune antibodies in blood and inflammatory markers in blood and CSF. Other cognitive and clinical variables related to safety are described below.

5.3.5.1 Cognitive/Clinical variables

The following variables will be included in the analyses:

1) Global function (change from baseline in CDR-SB (Clinical Dementia Rating Scale-Sum of Boxes)
2) Other cognitive tests (change from baseline in RBANS)
3) Behavior (change from baseline in NPI)
4) Columbia-Suicide Severity Rating Scale (C-SSRS) scores

Data will be presented per measurement time and dose group.
For endpoint 1), the baseline value is the score obtained at Screening except for subjects of sub-cohort 1.1 for which the baseline value is the score obtained at V1 [Week 0]. For other endpoints, the baseline value is the score obtained at V1 [Week 0]. Change from baseline [Week 0 or

Screening] at Visit 6 [Week 10], Visit 9 [Week 50] and Visit 11 [Week 74] will be summarized per dose group.
For endpoint 4), individual narratives will be presented for all subjects who report serious suicidal intent or any suicidal acts during the study.

Where data on RBANS, CDR-SB and NPI have been collected remotely by telephone interview, at subjects' homes or by videoconference, specific sub-analyses will be conducted in order to explore the potential impact of the remote mode of administration on the related results.

5.3.5.2 Adverse Events

All adverse events (AEs) will be coded using the most up-to-date version of the Medical Dictionary for Regulatory Affairs (MedDRA). As AEs are collected starting from the moment of the ICF signature, AEs and treatment-emergent adverse events (TEAEs) will be discriminated. A TEAE is defined as any AE that has an onset date/time on or after the first dose of investigational product, or any pre-existing condition that has worsened on or after the first dose of investigational product.

Overview of AEs will be given in terms of number and percentage of subjects per dose group experiencing:

- AEs
- SAEs
- Severe adverse events
- Adverse drug reactions
- AEs leading to withdrawal
- Deaths

AEs will also be summarized by System Organ Class and Preferred Term, according to severity (mild, moderate, severe) and according to relationship to drug.

Most frequent AEs will be presented per dose group in bar charts and time to first SAE will be presented graphically.

5.3.5.3 Other Variables Related to Safety

The following variables will be included in the analyses:

1) Routine hematology and biochemistry evaluations in blood
2) Routine biochemistry evaluations in urine
3) Inflammatory markers in blood and CSF
4) Autoimmune antibodies in blood
5) Physical examination results
6) Neurological examination results
7) Vital signs
8) Brain imaging (MRI) results
9) Electrocardiograms

Endpoints 1-4:

All laboratory parameters will be summarized per dose group and measurement time point. Change from baseline will be presented per dose group at Visit 11 [Week 74]. Shift tables will present shift from baseline [Week 0] to Visit 9 [Week 50].

A box plot will present laboratory values per measurement time and dose group.

Endpoints 5-6:

Data will be summarized per measurement time and dose group.

Endpoint 7:

Vital signs will be presented as described for laboratory parameters.

Endpoints 8-9:

Data will be summarized per measurement time and dose group.

5.3.6 Immune Response Analyses

5.3.6.1 Primary Endpoint

Anti-pTau IgG titers in serum (geometric mean, change from baseline, responder rate, peak and area under the curve).

5.3.6.2 Analysis of Primary Endpoint

Geometric mean and change from baseline at Visit 9 [Week 50] in Anti-pTau IgG titer will be calculated. The response will be defined using the parameters given in the SAP. Time-dependent development of the titers will be displayed graphically per dose group and per subject.

The number and proportion of subjects with a positive response will be summarized per visit and per dose group. The determination of negative/positive response will be done using a threshold defined during assay validation. Response rates will be displayed graphically in a response rate vs. dose plot to investigate whether there is any dose response relationship.

In addition, the peak and area under the curve of antibody response will be described. The results will be summarized per dose group.

No statistical testing has been planned for antibody responses.

During interim reviews, similar analyses will be performed up to the relevant visit as specified in section 5.3.8.

5.3.6.3 Secondary Endpoint

Anti-Tau IgG, anti-pTau and anti-Tau IgM titers in serum (geometric mean, change from baseline, responder rate, peak and area under the curve), determination of the IgG response profile by avidity testing.

5.3.6.4 Analysis of Secondary Endpoint

The analyses specified under 5.3.5.1 will be done to evaluate the induction of anti-Tau IgG, anti-pTau IgM and anti-Tau IgM titers in serum.

The response profile by avidity testing will also be described.

5.3.7 Exploratory Analyses

5.3.7.1 Exploratory Endpoints

1) AD biomarkers in blood and CSF
    a. antibody response components including for example total Tau, pTau and Tau fragments

   b. other putative AD biomarkers as relevant
2) T-cell activation (ELISPOT)
3) Inflammatory cytokines in blood (e.g. IL-1B, IL-2, IL-6, IL-8, IL-10, IFN- γ, and TNF- α)
4) Immune response e.g. antibodies against vaccine components, functional capacity of vaccine-induced antibodies
5) Behavior (NPI), cognitive (including the assessment of the decisional capacity using the MacCAT-CR interview in the Netherlands) and functional performance (RBANS, CDR-SB) scores

5.3.7.2 Analysis of Exploratory Endpoints

Endpoint 1:

Data will be summarized per measurement time and dose group. Change from baseline [Week 0] at Visit 4 [Week 10] (blood biomarkers only), Visit 6 [Week 26] and Visit 9 [Week 50] will also be analyzed.

Data will be presented graphically in a time series plot, presenting mean values per dose group, and in a box plot per measurement time and dose group.

Endpoint 2:

T-cell activation will be summarized by cohort, dose group and measurement time point. Change from baseline [week 0] at Visit 6 [Week 26] and Visit 9 [Week 50] will be presented per dose group.

Endpoint 3:

Data will be summarized per measurement time and dose group. Change from baseline [week 0] at Visit 4 [Week 10], Visit 6 [Week 26] and Visit 9 [Week 50].

Data will be presented graphically in a time series plot, presenting mean values per dose group, and in a box plot per measurement time and dose group.

Endpoint 4:

Geometric mean and change from baseline at Visit 9 [Week 50] in antibody titers will be calculated. The response will be defined using the parameters included in the SAP. Time-dependent development of the titers will be displayed graphically per dose group and per subject.

Endpoint 5:

Data will be presented per measurement time and dose group.

For the CDR-SB score, the baseline value is the score obtained at Screening except for subjects of sub-cohort 1.1 for which the baseline value is the score obtained at V1 [Week 0]. For the NPI and RBANS scores, the baseline value is the score obtained at V1 [Week 0]. Change from baseline [week 0 or Screening] at Visit 6 [Week 26], Visit 9 [Week 50] and Visit 11 [Week 74] will be summarized per dose group. For the MacCAT-CR assessment, the baseline value is the score obtained at Screening. The proportion of subjects in the Netherlands who are maintaining their decision-making capacity at Visit 5 [Week 24] and Visit 8 [Week 48] using the MacCAT-CR assessment will also be presented per dose group.

Where data on RBANS, CDR-SB and NPI have been collected remotely by telephone interview, at subjects' homes or by videoconference, specific sub-analyses will be conducted in order to explore the potential impact of the remote mode of administration on the related results.

5.3.8 Interim Analysis

The following interim analyses may be conducted in this study (also refer to Figure 1 for schematic overview under section 3.1.7).

Cohort 1

Timepoint	Sub-cohorts	Scope/objective
Visit 4 [Week 10], i.e. 2 to 4 weeks after the second injection	Sub-cohort 1.1, 1.2 and 1.3	Evaluate the safety, tolerability and immunogenicity of 300 µg, 900 µg and 1800 µg ACI-35.030 to dose escalate the sub-cohorts 1.2 and 1.3 and/or potentially expand the recruitment in the sub-cohort 1.1, 1.2 and/or 1.3. Biomarker results may also be reviewed
Visit 6 [Week 26], i.e. 2 to 4 weeks after the third injection	Sub-cohort 1.1, 1.2 and 1.3	Evaluate the safety, tolerability and immunogenicity of 300 µg, 900 µg and 1800 µg ACI-35.030 at this timepoint and potentially expand the recruitment in the sub-cohort 1.1, 1.2 and/or 1.3. Biomarker results may also be reviewed.
Visit 9 [Week 50], i.e. 2 to 4 weeks after the last injection at Week 48	Sub-cohort 1.1, 1.2 and 1.3	Evaluate the safety, tolerability and immunogenicity of 300 µg, 900 µg and 1800 µg ACI-35.030 at this timepoint, and potentially expand the recruitment in the sub-cohort 1.1, 1.2 and/or 1.3. Biomarker results may also be reviewed.
Visit 11 [Week 74]	Sub-cohort 1.1, 1.2 and 1.3	Evaluate the safety, tolerability and immunogenicity of 300 µg, 900 µg and 1800 µg ACI-35.030 at this timepoint and potentially expand the recruitment in the sub-cohort 1.1, 1.2 and/or 1.3. Biomarker results may also be reviewed.

Cohort 2

Timepoint	Sub-cohorts	Scope/objective
Visit 4 [Week 10], i.e. 2 to 4 weeks after the second injection	Sub-cohort 2.1, 2.2 (optional) and 2.3 (optional)	Evaluate the safety, tolerability and immunogenicity of 15 µg, 60 µg and 150 µg JACI-35.054 to dose escalate the sub-cohorts 2.2 and 2.3 and/or potentially expand the recruitment in the sub-cohort 2.1 and/or 2.2 and/or 2.3. Biomarker results may also be reviewed.
Visit 6 [Week 26], i.e. 2 to 4 weeks after the third injection	Sub-cohort 2.1, 2.2 (optional) and 2.3 (optional)	Evaluate the safety, tolerability and immunogenicity of 15 µg, 60 µg and 150 µg JACI-35.054 at this timepoint and potentially expand the recruitment in the sub-cohort 2.1, 2.2 and/or 2.3. Biomarker results may also be reviewed.
Visit 9 [Week 50], i.e. 2 to 4 weeks after the last injection at Week 48	Sub-cohort 2.1, 2.2 (optional) and 2.3 (optional)	Evaluate the safety, tolerability and immunogenicity of 15 µg, 60 µg and 150 µg JACI-35.054 at this timepoint, and potentially expand the recruitment in the sub-cohort 2.1, 2.2 and/or 2.3. Biomarker results may also be reviewed.

Visit 11 [Week 74]	Sub-cohort 2.1, 2.2 (optional) and 2.3 (optional)	Evaluate the safety, tolerability and immunogenicity of 15 µg, 60 µg and 150 µg JACI-35.054 at this timepoint and potentially expand the recruitment in the sub-cohort 2.1, 2.2 and/or 2.3. Biomarker results may also be reviewed.

Cohort 3 with both vaccines in sequential administration

Timepoint	Sub-cohorts	Scope/objective
Visit 4 [Week 10], i.e. 2 to 4 weeks after the second injection	Sub-cohort 3.1, 3.2 (optional) and 3.3 (optional)	Evaluate the safety, tolerability and immunogenicity of ACI-35.030 and JACI-35.054 (dosages to be determined) to dose escalate the sub-cohorts 3.2 and 3.3 and/or potentially expand the recruitment in the sub-cohort 3.1, 3.2 and/or 3.3. Biomarker results may also be reviewed.
Visit 6 [Week 26], i.e. 2 to 4 weeks after the third injection	Sub-cohort 3.1, 3.2 (optional) and 3.3 (optional)	Evaluate the safety, tolerability and immunogenicity of ACI-35.030 and JACI-35.054 (dosages to be determined) at this timepoint and potentially expand the recruitment in the sub-cohort 3.1, 3.2 and/or 3.3. Biomarker results may also be reviewed.
Visit 9 [Week 50], i.e. 2 to 4 weeks after the last injection at Week 48	Sub-cohort 3.1, 3.2 (optional) and 3.3 (optional)	Evaluate the safety, tolerability and immunogenicity of ACI-35.030 and JACI-35.054 (dosages to be determined) at this timepoint, and potentially expand the recruitment in the sub-cohort 3.1, 3.2 and/or 3.3. Biomarker results may also be reviewed.
Visit 11 [Week 74]	Sub-cohort 3.1, 3.2 (optional) and 3.3 (optional)	Evaluate the safety, tolerability and immunogenicity of ACI-35.030 and JACI-35.054 (dosages to be determined) at this timepoint and potentially expand the recruitment in the sub-cohort 3.1, 3.2 and/or 3.3. Biomarker results may also be reviewed.

The administration schedule of ACI-35.030 and JACI-35.054 may be subject to modification in case the magnitude of immunogenicity response observed during planned interim analysis will not be considered satisfactory by the Sponsor. If deemed necessary, such modification will be implemented in a protocol amendment.

The IAs described above for the 3 cohorts may also be performed in case of any sub-cohort expansion. Additional IAs to review the sustainability of immune response data may be conducted between weeks 26 and 50 and between weeks 50 and 74. If the post-injection period is extended in any sub-cohort, IA of immunogenicity data may also be conducted at Visit 13 (Week 98), and optionally at Visit 12 (Week 86).

In case data cut points occur within a short interval of time, interim analysis may be combined as deemed appropriate.

Safety assessment by the DSMB

Additionally to the above interim analyses, the safety data will be reviewed on a quarterly basis by the DSMB as described in a DSMB statistical analysis plan. DSMB members will receive unblinded packages containing all the safety/tolerability data prior to the meeting. Scope and frequency of the meetings are detailed in the DSMB charter.

Blinding

For all data analyses, the study treatment assignment will remain blinded for study participants, site personnel, CRO, study vendors and sponsor until all subjects in a given sub-cohort have reached the end of the safety follow-up visit (Visit 11, Week 74). The DSMB members may be unblinded to individual subjects. Designated sponsor representatives who have no access to the study database may be semi-unblinded to individual data, i.e. only after systematic re-coding of the subject number in order to prevent the identification of the associated treatment assignment.

If the follow-up of a specific sub-cohort is extended to allow for the assessment of the durability of the immune response, the study participants and site investigator/personnel will remain blinded to the treatment assignment until the end of the extended period (Visit 13, Week 98). During this extended follow-up period (weeks 74-98), designated sponsor representatives may be unblinded to the treatment assignment to allow for data review in preparation for later stage trials. Fully unblinded designated sponsor representatives will have no access to the study database and will not be authorized to share any unblinding information with any blinded study staff.

Unblinding at an individual level can be performed by site PIs or delegated site staff only for emergency reasons. Further information about the blinding of treatment assignment at the site level can be found in section 3.4.4.

Blind management will be outlined in a blinding/unblinding study plan.

6. STUDY MANAGEMENT

6.1 Ethics and Consent

6.1.1 Regulations and Guidelines

The trial will be conducted according to the principles and rules laid down in the Declaration of Helsinki and its subsequent amendments and will be conducted following the principles of ICH (International Conference on Harmonization) guideline for Good Clinical Practice and adherence with the applicable regulatory or legal requirements.

6.1.2 Institutional Review Board/Independent Ethics Committees and regulatory authorities

The study protocol will be submitted for examination to Institutional Review Board (IRB) / Ethics Committee (EC) and all applicable regulatory authorities. Commencement of the clinical trial is not permitted without written approval of the ethics committee and all applicable regulatory authorities.

The IRB/EC(s) must be notified of and approve all subsequent additions or changes in the study protocol. The regulatory authorities must be notified of and approve any substantial protocol amendments according to the requirements of applicable regulations.

6.1.3 Informed Consent

The written informed consent procedure should comply with any applicable regulatory requirement(s) and should adhere to GCP/ICH guidelines and to the ethical principles that have origin in the Declaration of Helsinki, as defined below but not limited to:

- The written informed consent must be obtained before any study specific procedures have been undertaken.
    - The investigator (or a study site staff designated by the investigator), must obtain a written informed consent signed and personally dated by the subject.
    - In addition, the Investigator (or a study site staff designated by the investigator) must obtain the consent from the subject’s caregiver to conduct his or her study related tasks, including supervision of concomitant medications, and contribution to assessment of global function, activities of daily living and behavior.
- One original of the informed consent form should be kept in the Investigator’s file and one original (or a copy if accepted according to local regulation) provided to the subject/caregiver.

Subjects and/or caregivers may withdraw consent throughout the study, whenever they consider appropriate to do so. Country-specific regulatory requirements regarding written informed consent procedures will be observed, in addition to the general principles outlined above.

In the Netherlands, the decisional capacity of subjects will be assessed at study start prior to signing the Informed Consent Form to evaluate their capacity to consent and the decisional capacity will be also assessed subsequently at V5 and V8, and before consenting for the optional post-injection extension.

Details on this assessment are available under section 3.5.6.

6.1.4 Confidentiality and privacy

Participant confidentiality and privacy is strictly held in trust by the participating investigators, their staff, and the Sponsor and their contracted vendors. This confidentiality is extended to cover testing of biological samples and genetic tests in addition to the clinical information relating to participants. Therefore, the study protocol, documentation, data, and all other information generated will be held in strict confidence. No information concerning the study or the data will be released to any unauthorized third party without prior written approval of the Sponsor.

All research activities will be conducted in as private a setting as possible.

The study monitor, other authorized representatives of the sponsor, representatives of the ECs, regulatory agencies or pharmaceutical company supplying the IMP may inspect all documents and records required to be maintained by the investigator, including but not limited to, medical records (office, clinic, or hospital) and pharmacy records for the participants in this study. The clinical study site will permit access to such records.

The study participant's contact information will be securely stored at each clinical site for internal use during the study. At the end of the study, all records will continue to be kept in a secure location for as long a period as dictated by the ECs, regulatory agencies, or Sponsor requirements.

Study participant research data, which is for purposes of statistical analysis and scientific reporting, will be transmitted to and stored at [redacted]. This will not include the participant's contact or identifying information. Rather, individual participants and their research data will be identified by a unique study identification number. The study data entry and study management systems used by clinical sites and by [redacted] research staff will be secured and password protected. At the end of the study, all de-identified study databases will be archived at the Sponsor.

6.1.5 Future Use of Stored Samples

Available biological samples (serum, cells and CSF) will be stored for potential exploratory assessments, e.g. potential biomarkers (CSF neurogranin, CSF VILIP-1, plasma NFL, Aβ1-42 and Aβ1-40), antibodies against vaccine components, functional capacity of vaccine-induced antibodies, binding of vaccine-induced antibodies to human paired filament tau, anti Tau B cell response, T cell activation in CSF, B cell activation in blood. Future use will be limited to research related to the study vaccines and/or Alzheimer's disease.

With the participant's approval and as approved by ECs, de-identified leftover blood and CSF samples will be stored at a Biosample Repository for up to 10 years after the end of the study (i.e. last subject completing the last follow-up visit). Genetic analyses will not be conducted with the leftover samples. Permission to transmit and store biological samples to long-term biosample repository will be included in the informed consent.

An individual participant can choose to withdraw consent to have biological specimens stored for future research anytime during the storage period.

6.2 Indemnification

The sponsor's indemnification of the investigator and institution during the conduct of this study is addressed in a letter of indemnification provided as a separate document. Other indemnification or insurance will be provided as necessary under EU and local regulations.

6.3 Discontinuation of the Study by the Sponsor

Should it prove necessary to discontinue the study permanently prior to completion (e.g. for compromised safety), subjects will be examined as per the final visit and appropriate early study completion CRFs completed.

In case it is decided to terminate the study prematurely, the sponsor will immediately notify investigators, relevant authorities and additional contacts. Investigators will promptly notify their relevant Ethics Committees and the study participants. The study products and materials will be returned.

In the Netherlands, based on Article 3 of the Revised CCMO Directive on the Assessment of Clinical Trial Agreements dated 1 November 2011, premature termination is only possible:

a. if the judgement of the competent medical research ethics committee that has assessed the study is irrevocably revoked;
b. if a reasonable case can be made for terminating the study in the interests of the health of the research subjects;
c. if it transpires that continuation of the study cannot serve any scientific purpose, and this is confirmed by the medical research ethics committee that has issued a positive decision on the study;
d. if one of the parties or the funder has been declared insolvent or a bankruptcy/winding-up petition has been filed in respect of one of the parties or the financier, or one of the parties or the financier is dissolved as a legal entity;
e. if the principal investigator is no longer capable of performing the tasks of the principal investigator, and no replacement agreeable to both parties can be found;
f. if one of the two parties fails to comply with the obligations arising from the agreement and, provided compliance is not permanently impossible, this compliance has not taken place within thirty days of the defaulting party receiving a written request to comply, unless failure to comply is not in reasonable proportion to the premature termination of the study;
g. if circumstances beyond the control of the sponsor, investigator or funder make it unreasonable to require the study's continuation.

6.4 Study Monitoring and Auditing

This study will be monitored for quality assurance at all stages of its development and conduct by the clinical research personnel employed by the sponsor or its representative. Monitoring will include personal visits and telephone communication to assure that the investigation is conducted according to the protocol, standard operating procedures, Guidelines of Good Clinical Practice, and applicable regulatory requirements. Quality control procedures will be applied to each stage of data handling to ensure that all data are reliable and have been processed correctly. On-site review of eCRFs will include a review of forms for completeness and clarity, and consistency with source documents available for each subject. A variety of original documents, data and records will be considered as source documents in this trial. The eCRF itself is not to be used as a source document under any circumstances.

Medical advisors and CRAs or assistants may request to witness subject evaluations occurring as part of this protocol. The investigator and appropriate personnel will be periodically requested to attend meetings organized by the sponsor to assure acceptable protocol execution. The study may be subject to audit by the sponsor or by regulatory authorities. If such an audit occurs, the investigator must agree to allow access to required subject records. By signing this protocol, the investigator grants permission to personnel from the sponsor, its representatives, and appropriate regulatory authorities, for on-site monitoring of all appropriate study documentation,

as well as on-site review of the procedures employed in eCRF generation, where clinically appropriate.

6.5 Retention of Records

The investigational sites will maintain all trial documentation and take measures to prevent accidental or premature destruction of these documents.

The investigator agrees to keep records, including the identity of all participating subjects, all original signed Informed Consent Forms, a copy of all eCRFs, and detailed records of drug disposition. These should be kept in an Investigator’s File that should be set-up by the Sponsor and kept regularly updated by the Investigator during the study.

To comply with international regulations, the records should be retained by the Investigator following the duration of the most stringent authority. The investigational sites must notify the sponsor before destroying any data or records.

6.6 Use of Study Findings

By signing the study protocol, the investigator agrees to the use of results of the study for the purposes of national and international registration. If necessary, the authorities will be notified of the investigator’s name, address, qualification, and extent of involvement. Reports covering clinical and biometric aspects of the study will be prepared by the sponsor or its representative.

6.7 Publications

As a multicenter trial, the sponsor intends to publish clinical data from all centers participating in the investigation. A publication committee selected by the sponsor will submit draft manuscripts to all participating investigators for their comments. In conformity with the Uniform Requirements for Manuscripts Submitted to Biomedical Journals published by the International Committee of Medical Journal Editors, investigators whose contribution consists solely in the collection of data will not be named individually as authors. Rather, those investigators will receive a collective authorship and will be identified in a note.

Individual investigators and/or their associates subsequently may publish additional finding of the study in scientific journals or present them at scientific meetings, provided that the sponsor is given ample opportunity to review any proposed abstract, manuscript, or slide presentation before its submission. This review is required to ensure that the sponsor is aware of all written and oral presentations of the data and does not imply any editorial review or restriction of the contents of the presentation or use.

7. REFERENCES

Alonso A, Zaidi T, Novak M, Grundke/Iqbal K (2001) Hyperphosphorylation induces self-assembly of tau into tangles of paired helical filaments/straight filaments. Proc Natl Acad Sci USA 98(12):6923-8

Appelbaum PS, Grisso T (2001). MacArthur competence assessment tool for clinical research (MacCAT-CR). Sarasota, FL, US: Professional Resource Press/Professional Resource Exchange.

Bierer LM, Hof PR, et al. (1995) Neocortical neurofibrillary tangles correlate with dementia severity in Alzheimer's disease. Arch Neurol 52: 81-88

Braak H, Braak E (1991) Neuropathological stageing of Alzheimer-related changes. Acta Neuropathol 82: 239-259

Cook IF (2008) Evidence based route of administration of vaccines. Human Vaccines 4(1): 67-73

Delacourte A (2001) The molecular parameters of tau pathology. Tau as a killer and a witness. Adv Exp Med Biol 487: 5-19

Diez-Domingo J, et al. (2015) Comparison of intramuscular and subcutaneous administration of a herpes zoster live-attenuated vaccine in adults aged ≥ 50 years: A randomized non-inferiority clinical trial. Vaccine 33: 789-795

Farlow MR, et al. (2015) Long-term treatment with active Aβ immunotherapy with CAD106 in mild Alzheimer’s disease. Alzheimers Res Ther 7(1): 23

Folstein MF, Folstein SE, McHugh PR (1975) “Mini-Mental State”: a practical method for grading the cognitive state of patients for the clinician. J Psychiatr Res 12:189-198

Goedert M, Spillantini MG, Crowther RA (1991) Tau proteins and neurofibrillary degeneration. Brain Pathol 1(4):279-286

Hughes CP, et al (1982) A new clinical scale for the staging of dementia. Am J Psychiatry 140: 566-572

Iaccarino L,et at. (2018) Local and distant relationships between amyloid, tau and neurodegeneration in Alzheimer's Disease. NeuroImage: Clinical 17 (2018) 452–464

Iba M, et al. (2013) Synthetic tau fibrils mediate transmission of neurofibrillary tangles in a transgenic mouse model of Alzheimer's-like tauopathy. J Neurosci 33: 1024-1037

Jalah R, et al. (2014) DNA and Protein Co-Immunization Improves the Magnitude and Longevity of Humoral Immune Responses in Macaques. PLoS ONE 9(3): e91550

Khurana S, et al. (2014) Heterologous Prime-Boost Vaccination with MF59-Adjuvanted H5 Vaccines Promotes Antibody Affinity Maturation towards the Hemagglutinin HA1 Domain and Broad H5N1 Cross-Clade Neutralization. PLoS ONE 9(4): e95496

Lu S, et al. (2009) Heterologous Prime-Boost Vaccination. Current Opinion in Immunology 21(3): 346–351

McKhann GM, et al. (2011) The diagnosis of dementia due to Alzheimer's disease: Recommendations from the National Institute on Aging – Alzheimer's Association workgroups on diagnostic guidelines for Alzheimer's disease. Alzheimer's & Dementia: The Journal of the Alzheimer's Association 7(3): 263-269

Novak P, et al. (2017) Safety and immunogenicity of the tau vaccine AADvac1 in subjects with Alzheimer's disease: a randomised, double-blind, placebo-controlled, phase 1 trial. Lancet Neuro 16(2): 123-134

Novak P, et al. (2021) ADAMANT: a placebo-controlled randomized phase 2 study of AADvac1, an active immunotherapy against pathological tau in Alzheimer's disease. Nature Aging volume 1, pages 521–534.

Orgogozo,J.M., et al, (2003). Subacute meningoencephalitis in a subset of patients with AD after Abeta42 immunization. Neurology 61, 46-54.

Pooler AM, et al. (2013) Physiological release of endogenous tau is stimulated by neuronal activity. EMBO Rep 14: 389-394

Randolph C, et al. (1998) The Repeatable Battery for the Assessment of Neuropsychological Status (RBANS): preliminary clinical validity. J Clin Exp Neuropsychol 20(3): 310-9

Sin JI, et al. (1999) DNA Priming–Protein Boosting Enhances Both Antigen-Specific Antibody and Th1-Type Cellular Immune Responses in a Murine Herpes Simplex Virus-2 gD Vaccine Model. DNA and cell biology, Volume 18, Number 10

Shukarev G, et al. (2017) A two-dose heterologous prime-boost vaccine regimen eliciting sustained immune responses to Ebola Zaire could support a preventive strategy for future outbreaks. Human Vaccines & Immunotherapeutics, vol. 13, no. 2, 266–270

Wahl M, Hermodsson S (1987) Intradermal, subcutaneous or intramuscular administration of hepatitis B vaccine: side effects and antibody response. Scand J Infect Dis 19(6): 617-21

8. APPENDICES

Appendix 1 NIA/AA Criteria for probable AD Dementia (McKhann *et al.*, 2011)

I. <u>Core Clinical criteria</u>

Dementia is diagnosed when there are cognitive or behavioral (neuropsychiatric) symptoms that:

- Interfere with the ability to function at work or at usual activities; and
- Represent a decline from previous levels of functioning and performing; and
- Are not explained by delirium or major psychiatric disorder;
- Cognitive impairment is detected and diagnosed through a combination of (1) history-taking from the patients and knowledgeable information and (2) an objective cognitive assessment, either a “bedside” mental status examination or neuropsychological testing.
- The cognitive or behavioral impairment involves a minimum of two of the following domains:
  - Impaired ability to acquire and remember new information
  - Impaired reasoning and handling of complex tasks, poor judgement
  - Impaired visuospatial abilities
  - Impaired language functions
  - Changes in personality, behavior, or comportment

II. <u>Criteria for the clinical diagnosis of PROBABLE Alzheimer’s disease</u>

Probable AD dementia is diagnosed when the patient:

- Meets criteria for dementia as described above and has the following characteristics:
  - Insidious onset;
  - Clear-cut history of worsening of cognition by report or observation; and
  - The initial and most prominent cognitive deficits are evident on history and examination in one of the following category.
    - Amnestic presentation
    - Nonamnestic presentations

III. <u>Other parameters increasing the certainty of AD dementia diagnosis</u>

- Evidence of causative mutation
- Presence of AD biomarkers
  - Low CSF $A\beta_{42}$
  - Positive PET amyloid imaging
  - Elevated CSF tau (total tau and phosphorylated tau)
  - Decreased fluorodeoxyglucose uptake on PET in temporo-parietal cortex
  - Disproportionate atrophy on structural magnetic resonance imaging in medial, basal and lateral temporal lobe and medial parietal cortex.
