# Supplementary material for: Safety and immunogenicity of two Tau-targeting active immunotherapies, ACI-35.030 and JACI-35.054, in participants with early Alzheimer's disease: a phase 1b/2a, multicentre, double-blind, randomised, placebo-controlled study
Source: eBioMedicine. 2025 Sep 18;120:105940. doi: 10.1016/j.ebiom.2025.105940 (PMC12481106; doi:10.1016/j.ebiom.2025.105940)
Supplement: Statistical Analysis Plan [file mmc3.pdf]

Statistical Analysis Plan

**Sponsor:**	AC Immune SA
**Protocol No:**	ACI-35-1802
**[redacted] Project ID:**	[redacted]
**Protocol Version No. / Date:**	V7.0 / 04-Nov-2021 (UK specific version)<br>V7.0 / 22-Oct-2021 (Global version)
**CRF Version No. / Date:**	V5.0 / 15-Dec-2022

**Title:**	A Phase Ib/IIa Multicenter, Double-Blind, Randomized, Placebo-Controlled Study to Evaluate the Safety, Tolerability and Immunogenicity of Different Doses, Regimens and Combinations of Tau Targeted Vaccines in Subjects with Early Alzheimer's Disease
**SAP No. / Date:**	V4.0 / 05-Dec-2023

Strictly Confidential. © 2020, [redacted] All rights reserved.

1.0 Approvals

**Sponsor**	
**Sponsor Name:**	AC Immune SA
**Representative / Title:**	[redacted]
**Signature / Date:**	
**Representative / Title:**	[redacted]
**Signature / Date:**	
[redacted]	
**Project Manager / Title:**	[redacted]
**Signature / Date:**	
**Biostatistician / Title:**	[redacted]
**Signature / Date:**	

Strictly Confidential. © 2020, [redacted] All rights reserved.

Table of Contents

1.0 Approvals 2
Table of Contents 3
2.0 Purpose 6
3.0 Scope 6
4.0 Introduction 6
5.0 Study Objectives 6
5.1 Primary Objective 6
5.2 Secondary Objectives 6
5.3 Exploratory Objectives 6
6.0 Study Design 7
6.1 Sample Size Considerations 12
6.2 Randomization 12
6.3 Blinding / Data Recoding of Immune Response and Biomarkers Results 12
7.0 Study Endpoints 14
7.1 Primary Endpoints 14
7.2 Secondary Endpoints 14
7.3 Exploratory Endpoints 14
8.0 Definitions 15
8.1 Study Day 15
8.2 Baseline 15
8.3 Change from Baseline 16
8.4 Past and Current Medical Histories 16
8.5 Prior and Concomitant Treatments 16
8.6 Treatment Compliance 17
8.7 Treatment Exposure 17
8.8 Treatment Emergent Adverse Event 18
8.9 Adverse Event Relatedness 18
8.10 Abnormal Laboratory Values 18
8.11 Handling of Partial or Missing Dates 18
8.12 Handling of Unscheduled Visits Data and Unscheduled Assessments 20
8.13 Handling of Missing Adverse Event Severity and Relationship 20

Strictly Confidential. © 2020, [redacted] All rights reserved.

8.14 Imputation of Titers Below the Limit of Quantification (BLQ) 20
8.15 Imputation of Biomarker Values Below the Limit of Quantification (BLQ) 21
8.16 Geometric Mean Titer 21
8.17 Normal Approximation Confidence Interval (CI) for the Geometric Mean Titer 21
8.18 Antibody Response 21
8.18.1 Anti-pTau IgG, anti-Tau IgG, anti-pTau IgM, anti-Tau IgM, anti-ePHF IgG and anti-CRM IgG 21
8.18.2 Anti-CpG IgG and anti-T50 IgG 22
8.19 Avidity index 22
8.20 Area Under the Curve (AUC) 22
8.21 Peak Concentration 22
9.0 Analysis Populations 22
9.1 Enrolled 22
9.2 Intention-to-Treat 22
9.3 Per Protocol 23
9.4 Safety Population 23
10.0 Interim Analyses 24
10.1 Cohort 1 with ACI-35.030 24
10.2 Cohort 2 with JACI-35.054 25
10.3 Cohort 3 with ACI-35.030 and JACI-35.054 25
10.4 Interim Analysis Procedures 26
10.5 Additional Clarifications Preventing Unblinding During IAs 27
10.6 Interim Analysis Conduct 27
11.0 Statistical Methods 28
11.1 Subject Disposition 28
11.2 Protocol Deviations and Violations 28
11.3 Demographic and Baseline Characteristics 29
11.4 Treatments 29
11.4.1 Study Drug Exposure 29
11.4.2 Prior and Concomitant Medications 29
11.5 Safety Analyses 29
11.5.1 Adverse Events 29
11.5.2 Laboratory Data 30

Strictly Confidential. © 2020, [redacted] All rights reserved.

11.5.3 Vital Signs 31
11.5.4 Electrocardiogram (ECG) 32
11.5.5 Physical Examinations 33
11.5.6 Neurological Examinations 33
11.5.7 Magnetic Resonance Imaging (MRI) 33
11.5.8 Cognitive/Clinical Variables 34
11.6 Immune Response Analyses 37
11.6.1 Immune Response for Primary Endpoints 37
11.6.2 Immune Response for Secondary Endpoints 37
11.6.3 Immune Response for Exploratory Endpoints 38
11.7 Other Exploratory Analyses 38
11.7.1 AD biomarkers in blood and CSF 38
11.7.2 T-cell activation (ELISPOT) 39
11.7.3 Inflammatory cytokines in blood 39
11.7.4 Volumetric MRI 39
12.0 Changes from the Analysis Planned in the Protocol 40
13.0 Validation 40
Appendix 1 Glossary of Abbreviations 41
Appendix 2 Tables, Figures, Listings 43
Appendix 3 Immunogenicity Testing for Cohort 1 and 2 55
Document History 57

Strictly Confidential. © 2020, [redacted] All rights reserved.

2.0 **Purpose**

The statistical analysis plan (SAP) describes the statistical methods to be used during the reporting and analyses of data collected under AC Immune Protocol ACI-35-1802.

3.0 **Scope**

The SAP outlines the following:

- Study objectives
- Study design
- Variables analyzed and analysis sets
- Applicable study definitions
- Statistical methods

Any major updates to the SAP after finalization of the current version will be added to the SAP document history. If any non-administrative changes are required, the SAP will be updated by [redacted] and approved again by the sponsor.

4.0 **Introduction**

This SAP should be read in conjunction with the study protocol and Case Report Form (CRF).

This version of the plan has been developed using the protocol(s) and CRF version specified in the title page.

Any further changes to the protocol and/or CRF may necessitate updates to the SAP.

5.0 **Study Objectives**

5.1 Primary Objective

The primary study objectives are to assess:

- The safety and tolerability of study vaccines
- The immunogenicity of study vaccines (induction of Immunoglobulin G [IgG] titers against phospho-Tau [pTau] in serum)

5.2 Secondary Objectives

The secondary study objectives are to further assess the immunogenicity of study vaccines including:

- To further assess the immunogenicity of study vaccines (induction of IgG titers against Tau and of Immunoglobulin M [IgM] titers against pTau and Tau in serum)
- To assess the avidity of antibodies elicited by immunization

5.3 Exploratory Objectives

- To explore the effect of study vaccines on putative biomarkers of the progression of AD, i.e. blood and/or Cerebrospinal Fluid (CSF) concentrations of total Tau and pTau proteins, Tau fragments and other related biomarkers
- To explore the effect of study vaccines on the activation of T-cells in blood
- To explore the activity of study vaccines on blood inflammatory cytokines (e.g., Interleukin-1B [IL-1β], IL-2, IL-6, IL-8, IL-10, Interferon-Gamma [IFN- γ], and Tumor Necrosis Factor alpha [TNF-α])

Strictly Confidential. © 2020, [redacted] All rights reserved.

- To further explore the effect of study vaccines on the immune response (e.g. antibodies against vaccine components, functional capacity of vaccine-induced antibodies)
- To explore the effect of study vaccines on behavior, cognitive (including, in the Netherlands, the maintenance of the decisional capacity) and functional performance

6.0 **Study Design**

This section reflects the language from Global Protocol Version 7.0 dated 22-Oct-2021. Country-specific changes to the design of the study and the schedule of assessments have been included in the respective protocol versions (V7.0 dated 04-Nov-2021 (UK specific version)).

This is a multicenter prospective placebo-controlled, double-blind and randomized study to assess treatment with Tau targeted vaccines versus placebo over 50 weeks (i.e. 12 months) in up to 216 subjects with early Alzheimer's Disease. For both cohorts 1 and 2, immunizations will be performed at months 0 (Week 0), 2 (Week 8), 6 (Week 24)* and 12 (Week 48). Based on the safety and immunogenicity results the protocol may be amended to test additional regimens. The subjects will be enrolled in up to 3 Cohorts, each with up to 72 subjects, the actual number depending on the number of sub-cohorts studied and the number of sub-cohorts in which expansion is performed.

* Due to the Covid-19 pandemic, local travel restrictions in Finland effective as of 18 March 2020, and the need to preserve the safety of study participants, modifications were made to the schedule of immunizations of sub-cohort 1.1 subjects who had not received the 3rd injection at month 6 already: immunizations at month 6 (week 24) were not performed in 7 of 8 sub-cohort 1.1 subjects for whom the planned Visit 5 did not allow all assessments to be done according to the protocol. In addition, Visits 5 (Week 24), 6 (Week 26) and 7 (Week 36) were replaced by remote visits in case these fell during the at-risk period (refer to protocol sections 3.5.2. 10, 3.5.2.11, 3.5.2.12 and 3.5.2.14).

Cohort 1 with ACI-35.030
For all cohort 1 subjects, the treatment period will last 50 weeks with the treatment/placebo being administered 4 times as outlined in the previous paragraph. The treatment period is 50 weeks and will be followed by a 24-week safety follow-up period starting 2 weeks after the last administration. Subjects who for any reasons have received less than 4 administrations will be followed at least for the same duration after their last administration until week 74. The overall subject participation will be up to around 80 weeks from first screening (screening visit is estimated as 6 weeks; 50 weeks of treatment and 24 weeks of safety follow-up) to last safety follow-up visit.

In this cohort, the ACI-35.030 vaccine will be assessed. The placebo used will be a phosphate-buffered saline (PBS) solution. The administration route of treatment will be intramuscular. ACI-35.030 will be tested in up to 3 dose levels in cohort 1, within 3 sub-cohorts. Up to 32 subjects, depending on dose escalation and a decision to expand one sub-cohort, will be treated as outlined below.

Sub-cohort 1.1 (8 subjects)
ACI-35.030 at 300 µg/dose will be administered in 6 subjects and the placebo will be administered in 2 subjects. The safety and tolerability data after all subjects have received the second injection in sub-cohort 1.1 permit dose escalation after review by Data Safety Monitoring Board (DSMB).

Sub-cohort 1.2 (8 subjects)
ACI-35.030 at 900 µg/dose will be administered in 6 subjects and the placebo will be administered in 2 subjects. The safety and tolerability data after all subjects have received the second injection in sub-cohort 1.2 permit dose escalation after review by DSMB.

Sub-cohort 1.3 (8 subjects)
ACI-35.030 at 1800 µg/dose will be administered in 6 subjects and the placebo will be administered in 2 subjects.

Strictly Confidential. © 2020, [redacted] All rights reserved.

Cohort 2 with ACI-35.054
All cohort 2 subjects will receive a second generation ACI-35 vaccine (JACI-35.054) administered alone. Administration schedule, route of administration, treatment duration and follow-up duration are identical to cohort 1. Up to 3 dose levels of JACI-35.054 administered by the intramuscular route will be tested in up to 3 sub-cohorts. The placebo used will be a phosphate-buffered saline (PBS) solution.

Sub-cohort 2.1 (8 subjects)
JACI-35.054 at 15 µg/dose will be administered in 6 subjects and the placebo will be administered in 2 subjects. The safety and tolerability data after all subjects have received the second injection in sub-cohort 2.1 permit dose escalation after review by DSMB.

Sub-cohort 2.2 (8 subjects) – Optional
JACI-35.054 at up to 60 µg/dose will be administered in 6 subjects and the placebo will be administered in 2 subjects. This sub-cohort will be optional and may be conducted based on good safety and tolerability observed in sub-cohort 2.1 and in case the antibody response in this previous sub-cohort is anticipated to be optimized at the dosage of up to 60 µg. At the time of the release of protocol version 7.0 dated 22 Oct 2021, this sub-cohort with a dosage of 60 µg/dose has been initiated following the dose escalation after the 15 µg/dose sub-cohort.

Sub-cohort 2.3 (8 subjects) – Optional
JACI-35.054 at up to 150 µg/dose will be administered in 6 subjects and the placebo will be administered in 2 subjects. This sub-cohort will be optional and may be conducted based on good safety and tolerability observed in sub-cohort 2.2 and in case the antibody response in this previous sub-cohort is anticipated to be further optimized at a higher dosage.

Cohort 3 with ACI-35.030 and JACI-35.054 in sequential administration
The administration schedule, route of administration, duration of treatment and follow-up are identical to cohorts 1 and 2 but both vaccines ACI-35.030 and JACI-35.054 will be administered in a sequence based on non-human primate immunogenicity data and on data reviewed during the previous sub-cohort interim analyses (IA). In any of the sub-cohorts listed below, the selected dosages of the 2 study vaccines will have been evaluated in cohorts 1 and 2 and safety monitored for at least 10 weeks and safety data of the selected dosages of the 2 study vaccines reviewed by the DSMB.

Note: Planned as optional in the protocol, Sub-cohort 2.3 and all Cohort 3 have not been initiated since the data obtained during the study from other sub-cohorts was considered to be sufficient to inform the subsequent clinical development steps. Therefore, no analyses will be conducted.

Sub-cohort expansion
Optional sub-cohort expansion of up to 16 additional subjects (i.e. up to 12 on active treatment and up to 4 on placebo per sub-cohort) may be considered in sub-cohort(s) with a favorable safety and immunogenicity profile with the objective to confirm the initial sub-cohort(s) immunogenicity results. The number of additional subjects to be enrolled will be determined based on the observed immunogenicity response rate in the sub-cohort(s) and on the potential gain in precision of the response rate estimate in the expanded sub-cohort(s), and will not exceed 16 (i.e., up to 24 subjects overall per sub-cohort). In addition, all other immunogenicity endpoints available at the interim timepoint may be considered. The dose-level, sequence of administration and administration regimen of study vaccine(s) will be identical to the initial part of the sub-cohort.

Note: An expansion of 17 additional subjects was done in sub-cohort 1.2 (for a total of 25 subjects in that sub-cohort) to allow further assessment of safety and immunogenicity at that dose. It was considered that the recruitment of 17 (instead of up to 16 as planned per protocol) subjects is acceptable by the Sponsor.

Strictly Confidential. © 2020, [redacted] All rights reserved.

Safety follow-up

All subjects will be kept under clinical observation for 24 hours after the first administration of study vaccine and for 4 hours after subsequent administrations of study vaccine. During that period, the vital signs will be monitored and the injection site will be observed on a regular basis. A physical examination will be performed prior to discharge. The clinical observation may be prolonged in case of safety concerns according to the clinical judgement of the investigator. A subsequent safety assessment will also be performed 48 to 72 hours after each immunization by telephone call for all study subjects. For adverse events considered possibly or probably related to the study vaccine of greater than mild severity according to investigator assessment, subjects will be required to return to the clinic for additional safety assessment. In each sub-cohort, the first dosing of the first 4 subjects should be performed once the safety assessment at 48 to 72 hours of the previous subject has been performed. This will allow the site principal investigator to assess whether there is any clinically relevant safety issue related to study vaccine prior to dosing the subsequent subject. Safety laboratory samples will be collected at baseline, prior to each injection and 2-4 weeks after each injection.

Laboratory results will be reviewed by the Medical Monitor on a periodic basis including all out of range values.

All treated subjects will have a safety follow-up period of 24 weeks (6 months) after the end of the treatment period. During this period, subjects will be asked to attend a first follow-up visit 19 weeks after the last administration and a last visit at the end of the follow-up period (26 weeks after the last administration). Participants' safety will be monitored throughout the study with regular review of safety data by the DSMB.

Post-injection period extension (optional)

Based on immunogenicity data, subjects from sub-cohort(s) may be invited to attend two additional visits (at Week 86 and Week 98) taking place after the end of the safety follow-up period (week 74). In such a case, an IA of immunogenicity data as well as available biomarker data may also be conducted at Visit 13 (Week 98), and optionally at Visit 12 (Week 86), to monitor the durability of the immune response and any biomarker changes in blood. All subjects participating in this post-injection period extension will have to reconsent. During this additional 24-week (6 month) period, blood will be collected for immunogenicity and biomarker testing. The aim of this optional study extension is to help inform the dosing regimen in subsequent clinical trials of the vaccine(s). AEs/SAEs and concomitant medications (new and/or changes from the previous visit) will also be collected during these 2 additional visits. Upon investigator's medical judgement, other assessments can be performed during this period (e.g., MRI, ECG, etc.) if clinically indicated.

In the Netherlands, the subjects' decisional capacity will be re-assessed before consenting for this optional post-injection extension.

Note: Planned as optional per the protocol, post-injection period extension was not conducted as deemed unnecessary by the sponsor.

Any randomized subject withdrawing before the 2nd injection for reasons other than safety may be replaced.

A schematic overview of the cohorts is given below in Figure 1:

Strictly Confidential. © 2020, [redacted] All rights reserved.

Figure 1 Schematic Overview of Cohorts Design

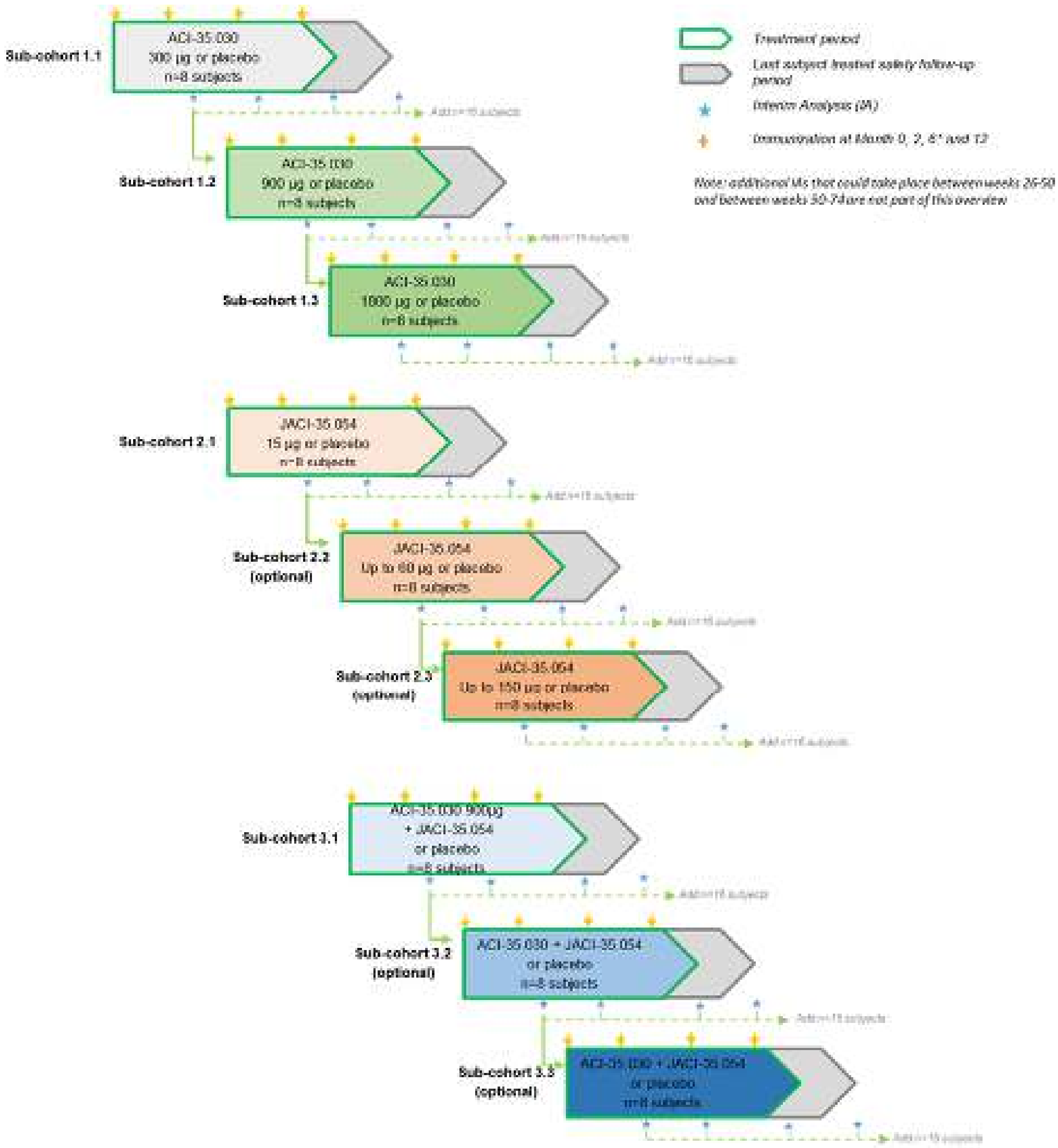

Important: The start of subject screening and treatment in cohort 2 is not linked to any IA performed in cohort 1. It is anticipated that screening in sub-cohort 2.1 will be done in parallel with screening for sub-cohort 1.2. Potential lags in the start of the screening may occur depending on e.g. subject recruitment rate or regulatory approval timelines.

The start of subject screening and treatment in cohort 3 will be based on IA data generated in sub-cohorts 1.1, 1.2 and 2.1, and if needed in sub-cohort 2.2, from at least week 10 onwards.

Strictly Confidential. © 2020, [redacted] All rights reserved.

Figure 2 Schedule of Assessments (specific to Protocol V7.0 dated 22-Oct-2021 (Global Version))

Study Plan	Screening	Treatment period																	Follow-up period		Optional Post-injection period extension ∂ π	
Visit	V_S	V_1	P_1	V_2	V_3	P_2	V_4	$V_{4.1}$	$V_{4.2}$	V_5 Δ	P_3 Δ	V_6 Δ	$V_{6.1}$	V_7 Δ	$V_{7.1}$	V_8	P_4	V_9	V_{10}	V_{11}	V_{12}	V_{13}
Time (visit or week ± days)	-42d to -3d	0	V_1 +2-3d	w2 ±3d ∞	w8 ±3d	V_3 +2-3d	w10 ±3d ∞	W15 ±3d ∂	W20 ±3d ∂	w24 ±7d	V_5 +2-3d	w26 ±2d ∞	W31 ±3d ∂	w36 ±3d	W42 ±3d ∂	w48 ±7d	V_8 +2-3d	w50 ±2d ∞	w67 ±7d	w74 ±7d	w86 ±7d	w98 ±7d
Treatment (immunization)		•			•					•						•						
Decisional capacity (MacCAT-CR)□	•									•						•						
Subject Information / Consent	•																					
History (Subject medical history / Family history of AD)	•																					
Inclusion / Exclusion criteria	•	•																				
Adverse Events / Concomitant Medication §	•	•	•	•	•	•	•	•	•	•	•	•	•	•	•	•	•	•	•	•	•	•
Physical and neurological examination	•	•		•	•		•			•		•		•		•		•	•	•		
Vital signs	•	•		•	•		•			•		•		•		•		•	•	•		
Cognitive/Clinical assessments																						
- C-SSRS, RBANS	•	•										•						•		•		
- MMSE	•																					
- CDR	•	•‡										•						•		•		
- NPI		•										•						•		•		
Lumbar Puncture (CSF)*	•											•						•				
- Cell Count (to be performed at site)/Inflammatory markers	•											•						•				
- Biomarkers (i.e. levels of amyloid, Total Tau pTau, Tau fragments and other biomarkers)	•											•						•				
- CSF albumin	•											•						•				
Blood																						
- Hematology & biochemistry	•	•		•	•		•			•		•		•		•		•	•	•		
- Serum albumin	•											•						•				
- Serum pregnancy test †	•																					
- Screening tests: HIV, Hepatitis B and C, VDRL (syphilis serology), B12, folate, thyroid function tests, ApoE genotype	•																					
- Coagulation indices: PT (INR), aPTT	•									•						•						
- Anti-Tau IgG/IgM, anti-pTau IgG/IgM	•	•		•	•		•	•	•	•		•	•	•	•	•		•	•	•	•	•
- Cytokines	•	•		•	•		•			•		•		•		•		•	•	•		
- Biomarkers (i.e. Total Tau and pTau levels)		•		•	•		•	•	•	•		•	•	•	•	•		•	•	•	•	•
- T-Cell profile		•										•						•				
- ANA and anti-dsDNA	•	•		•	•		•			•		•		•		•		•	•	•		
Urine																						
- Routine urine evaluation	•	•		•	•		•			•		•		•		•		•				
- Urinary pregnancy test		•			•					•						•						
ECG	•											•						•		•		
MRI	•						•					•						•		•		

□ *Only in the Netherlands. Initial assessment to be done prior to the informed consent signature. * Lumbar punctures are mandatory procedures including at screening visit where the presence of amyloid will be assessed as an inclusion criterion.§ All subjects will be kept under clinical observation for 24 hours after the first administration of study vaccine and for 4 hours after subsequent administrations of study vaccine (second, third and fourth administrations). A subsequent safety assessment will also be performed 48 to 72 hours after each immunization by telephone call (P) for all study subjects. In each sub-cohort, the first dosing of the first 4 subjects should be performed once the safety assessment at 48 to 72 hours of the previous subject is done to confirm there is no clinically relevant safety issue related to study vaccine, according to the site principal investigator. ∞ Visit to be performed preferentially 2 weeks +/- 3 days and exceptionally up to 28 days after immunization. † Blood draw between day -14 and day -3 prior to baseline (V1). ‡For sub-cohort 1.1 only. Δ In sub-cohort 1.1: modification of V_5, P_3, V_6, V_7 procedures and assessments during Covid-19 pandemic. Refer to details in sections 3.5.2.10, 3.5.2.11, 3.5.2.12 and 3.5.2.14.*
∂ Only applicable for sub-cohorts in which the subject has not yet reached the visit timepoint. If feasible, agreed with subject and locally permitted, these visits may also be performed at subject's home. π *Optional extended study period, only applicable if a decision is made by the sponsor. Concerned subjects will consent and in the Netherlands, the subjects' decisional capacity will be assessed before consenting.*

Strictly Confidential. © 2020, [redacted] All rights reserved.

6.1 Sample Size Considerations

Antibody titer assessment: The preliminary assessment of the antibody response and of the safety/tolerability (comparison between sub-cohorts) will be performed based on the data of all randomized subjects (6 on ACI-35.030/JACI-35.054 and 2 on placebo per sub-cohort) based on the timepoints related to the different IAs that will be conducted during the study. The initial number of subjects in each sub-cohort is consistent with the customary sample size employed in this type of studies. It is expected to allow clinical judgement of safety and tolerability, and to get preliminary evidence of an acceptable immune response. This sample size is also anticipated to provide sufficient evidence to support the possible enrollment of up to 16 additional subjects in the sub-cohort(s) showing the most favorable antibody response profile(s) with the objective to confirm the initial results. The number of additional subjects to be enrolled will be determined based on the preliminary observed immunogenicity response rate in the subcohort(s) and on the potential gain in precision of the response rate estimate in the expanded sub-cohort(s), and will not exceed 16 (12 on ACI-35.030/JACI-35.054 and 4 on placebo per sub-cohort). In addition, all other immunogenicity endpoints available at the interim timepoint may be considered in the sample size re-estimation. This may include but is not limited to the observed variability of titers such as the Anti-pTau IgG and the Anti-e-PHF IgG.

In total, up to 24 subjects can be randomized to cohort 1, cohort 2 and cohort 3, respectively (not taking into account sub-cohort(s) expansion). Any randomized subject withdrawing before the 2nd injection for reasons other than safety may be replaced in the cohort.

For safety, if an AE occurs at the rate of 1% or 10%, then the chance of observing such an AE among 6 subjects receiving that dose of active treatment will be 6% or 47%, respectively. If no AE of a given type is observed in any of the 6 subjects at a given dose of active treatment, then with 80% (90%) confidence the true incidence of that type of AE at that dose is at most 24% (32%).

An expansion was done in sub-cohort 1.2 in which 17 additional subjects were recruited (for a total of 25 subject in the sub-cohort) to allow further assessment of safety and immunogenicity at that dose.

6.2 Randomization

A randomization list based on a sequence of numbers has been computer generated by Suvoda. The individual medication codes are provided by the IP supplier ([redacted]). A dummy randomization list was created by Suvoda and then reviewed and approved by the blinded ST and AC Immune. Following this approval, the live randomization list was created and approved by an unblinded ST ahead of implementation into the Interactive Response Technology (IRT) system.

Study medication will be labelled with the corresponding medication number. Investigators will use a secure web-based system to randomize eligible subjects to study medication. The Suvoda IRT system will be used for randomization in the study. At screening, participating subjects will be given a subject number that will also correspond to the subject’s identification in the study. Assigned numbers will be unique identifiers with 4 digits for the site and 2 last digits for the sequential order of enrolment at a given site.

6.3 Blinding / Data Recoding of Immune Response and Biomarkers Results

For all data analyses, the study treatment assignment will remain blinded for study participants, site personnel, CRO, study vendors and sponsor until all subjects in a given sub-cohort have reached the end of the safety follow-up visit (Visit 11, Week 74). The DSMB members may be unblinded to individual subjects. Designated sponsor representatives who have no access to the study database may be semi-

Strictly Confidential. © 2020, [redacted] All rights reserved.

unblinded to individual data, i.e. only after systematic re-coding of the subject number in order to prevent the identification of the associated treatment assignment.

It was planned as per protocol that if the follow-up of a specific sub-cohort is extended to allow for the assessment of the durability of the immune response, the study participants and site investigator/personnel will remain blinded to the treatment assignment until the end of the extended period (Visit 13, Week 98). During this extended follow-up period (weeks 74-98), designated sponsor representatives may be unblinded to the treatment assignment to allow for data review in preparation for later stage trials. Fully unblinded designated sponsor representatives will have no access to the study database and will not be authorized to share any unblinding information with any blinded study staff. Eventually, the follow-up period of specific sub-cohorts was not extended,

Since the presence of antibody titers in serum and the variations in biomarker levels in plasma or CSF samples may unblind anyone seeing these data, a recoding (blinding) procedure will be followed prior to transmitting these data to the Sponsor Data Review Committee / Unblinded Team in order to maintain the study blind on an individual subject basis. Study team members having access to immune response and biomarkers data associated with the original patient ID (i.e. the patient ID as assigned by IRT and captured in eCRF) will be considered unblinded, i.e. only the Unblinded Reporting Team (statistical programmers, statistician) should have access to these.

New patient IDs will be programmatically generated and assigned to patients using SAS® by an unblinded statistical programmer at [redacted] The “RANUNI” function will be used to recode patient IDs and ensure that there is no discernible sequence or pattern, with a seed number that is known to the unblinded statistical programmer and a second unblinded person at the CRO, but unknown to the Sponsor/Blinded Team. Analysis output of antibody titer and biomarker results will be displayed using recoded patient IDs. Hence, the Sponsor will not be able to draw conclusions as to the patients’ original IDs.

A list with original patient IDs and associated recoded patient IDs will be generated via SAS®. The list will be maintained by the unblinded statistical programmer and made available to the Sponsor/Blinded Team after study unblinding (following database lock) and filed in the appropriate unblinded section the electronic Trial Master Files (eTMF).

DATA MUST NEVER BE SENT FROM THE ANALYZING LABORATORIES TO AC IMMUNE SA, THE SITE, [redacted] BLINDED TEAM MEMBERS, PATIENTS AND/OR CAREGIVERS TO AVOID ANY UNBLINDING!

All electronic files and related emails provided by the analyzing laboratories will be maintained by the unblinded team and filed in the unblinded section of the eTMF. Sponsor should not have access to these files before study unblinding (following database lock).

In case there is missing data at one or more visit timepoints for a patient (e.g. in case of early withdrawal from treatment due to safety), the results pertaining to that patient will only be included in the TFLs summarizing the results per dose group and will not be presented individually. This is applicable when producing IA.

All SAS programs created for the study for data recording purposes will be validated according to [redacted] Standard Operation Procedures. No recoding will be done for safety, tolerability and data related to cognitive and clinical effects, as data will be extracted directly from the eCRF.

Additionally, anti-double stranded DNA results (corresponding to the anti-dsDNA ELIA) are blinded to the site investigator due to risk of unblinding, but they must not be blinded to the DSMB and sponsor. Both should receive the results for both anti-dsDNA testing (ELIA and FARR) to allow safety monitoring of the subjects.

Strictly Confidential. © 2020, [redacted] All rights reserved.

7.0 **Study Endpoints**

7.1 Primary Endpoints

The primary focus of this study is the assessment of safety and tolerability of ACI-35.030 and JACI-35.054. Safety and tolerability will be assessed by examination of a number of endpoints:

- Adverse events
- Immediate and delayed reactogenicity (e.g. anaphylaxis, local and systemic reactogenicity, including immune-complex disease)
- Suicidal ideation as assessed by Columbia-Suicide Severity Rating Scale (C-SSRS)
- Behavior as assessed by Neuropsychiatric Inventory Scale (NPI)
- Cognitive and functional assessments (RBANS, CDR-SB)
- Vital signs
- Magnetic Resonance Imaging (MRI) imaging
- Electrocardiogram
- Routine hematology and biochemistry evaluation in blood and urine
- Evaluation of autoimmune antibodies including anti-DNA antibodies in blood
- Inflammatory markers in blood and CSF

The immune response (i.e. immunogenicity) will be assessed by:

- Anti-pTau IgG and Anti-ePHF IgG titers in serum (e.g. geometric mean, change from baseline, responder rate, peak and area under the curve)

Note: anti-pTau IgG means the antibody response generated against pathological Tau species. This umbrella denomination includes anti-pTau IgG and anti-ePHF IgG responses.

7.2 Secondary Endpoints

Immunogenicity of study vaccine will be assessed by:

- Examination of the immune response: anti-Tau IgG, anti-pTau IgM and anti-Tau IgM titers in serum (e.g. geometric mean, change from baseline, responder rate, peak and area under the curve)
- Determination of the IgG response profile by avidity testing

7.3 Exploratory Endpoints

Activity of study vaccine on exploratory endpoints will be assessed by the examination of:

- Change from baseline of putative AD biomarker titers in blood and/or CSF (e.g. total Tau, pTau, Tau fragments and other related biomarkers)
- Change from baseline in T-cell activation levels as measured in blood
- Change from baseline of inflammatory cytokine titers in blood
- Change from baseline in antibody titers (e.g. antibodies against other vaccine components) in blood
- Change from baseline in Behavior (NPI)

Strictly Confidential. © 2020, [redacted] All rights reserved.

- Change from baseline in cognitive (including the proportion of subjects maintaining their decisional capacity during the study using the MacCAT-CR interview in the Netherlands) and functional performance assessed through RBANS and CDR-SB scores
- Volumetric MRI

8.0 **Definitions**

8.1 Study Day

All study days on or after the first administration of study vaccine (Week 0/Visit 1) will be calculated as date of assessment minus date of first administration of study vaccine + 1. Study days before the first administration of study vaccine will be calculated as date of assessment minus date of first administration of study vaccine. Every effort will be made to avoid missing and/or incomplete dates. In case of missing and/or incomplete dates no study days will be calculated.

8.2 Baseline

In most cases, except for antibody titers, the baseline value is defined as the last non-missing value prior to the first immunization at Week 0 (Visit 1). For some variables this will be the value from Visit 1 provided that the assessment occurs before administration of the first study vaccine and for other variables this will be from the screening period.

For applicable variables that include a date and time, both date and time should be used in the calculations.

Example 1

Subject	Datetime of First Injection	Visit ID	Assessment Date	Baseline Flag
100101	16-Oct-2019: 10:41	Screening	14-Oct-2019: 10:35	
		Visit 1	16-Oct-2019: 10:00	Yes

Example 2

Subject	Datetime of First Injection	Visit ID	Assessment Date	Baseline Flag
100101	16-Oct-2019: 10:41	Screening	14-Oct-2019: 10:35	Yes
		Visit 1	16-Oct-2019: 11:00	

Example 3

Subject	Datetime of First Injection	Visit ID	Assessment Date	Baseline Flag
100101	16-Oct-2019: 10:41	Screening	14-Oct-2019: 10:35	
		Visit 1	16-Oct-2019: 10:00	
		UNS* Visit 1	16-Oct-2019: 10:30	Yes

* UNS=Unscheduled.

For antibody titers the baseline will be calculated as the mean of the titers measured at the screening and visit 1, including unscheduled visits, provided that they occur prior the date and time of the first injection. All blood samples withdrawn for immunogenicity parameters assessment at Day 0 (Visit 1) are always pre-dose (taken BEFORE the administration of study vaccine), therefore these will be included in the baseline titers calculation. In case the time of blood collection is after the time of the injection at Day 0 then only the screening measurement will be considered as baseline.

Strictly Confidential. © 2020, [redacted] All rights reserved.

Example 4

Subject	Datetime of First Injection	Visit ID	Assessment Date	Titer
100101	16-Oct-2019: 10:41	Visit 1	16-Oct-2019: 11:00	513

The mean baseline value will be 513. That is, the titer from Visit 1 being the only one available assessment and there are no other assessments up to and including the date the first injection is administered (only if the time of the titer collection is after the first injection time) will be used for the baseline titer.

Example 5

Subject	Datetime of First Injection	Visit ID	Assessment Date	Titer
100101	16-Oct-2019: 10:41	Screening	14-Oct-2019: 10:35	532
		Visit 1	16-Oct-2019: 10:15	513

The mean baseline value will be (532+513)/2, i.e. 522.5. That is, titers from screening and assessments up to and including the date when the first injection is administered (only if the time of the titer collection is before the first injection time) will be used for the baseline titer.

Example 6

Subject	Datetime of First Injection	Visit ID	Assessment Date	Titer
100101	16-Oct-2019: 10:41	Screening	14-Oct-2019: 10:35	532
		Visit 1	16-Oct-2019: 10:00	513
		UNS* Visit 1	16-Oct-2019: 10:30	527

* UNS=Unscheduled.

The mean baseline value will be (532+513+527)/3, i.e. 524. That is, titers from screening and assessments up to and including the date when the first injection is administered (only if the time of the titer collection is before the first injection time) will be used for the baseline titer.

8.3 Change from Baseline

The change from baseline value is defined as the value at any given post-baseline timepoint minus the baseline value, i.e. post-baseline value – baseline value.

Percent change from baseline is defined as follows: ((post-baseline value – baseline value) / baseline value) * 100.

Fold-change from baseline is defined as the ratio calculated by the post-baseline value divided by the baseline value. A value of 1 indicates a post-baseline value equal to the baseline value. A value above 1 indicates an increase from baseline; a value below 1 indicates a decrease from baseline.

8.4 Past and Current Medical Histories

Past medical histories are those with a stop date prior to the informed consent date.

Current medical histories are those which started prior to inclusion in the study but which are ongoing or with a stop date on or after the informed consent date.

8.5 Prior and Concomitant Treatments

Prior treatments are defined as those with a stop date prior to the first administration of study vaccine.

Strictly Confidential. © 2020, [redacted] All rights reserved.

Concomitant treatments are defined as treatments that are ongoing or with a stop date on or after the first administration of study vaccine.

Example 1

		Prior/Concomitant Medication		
Subject	Date of First Injection	Start Date	Stop Date	Prior/Concomitant
100101	16-Oct-2019	14-Sep-2019	15-Oct-2019	Prior

NB. Stopped prior to date of first vaccine so prior medication.

Example 2

		Prior/Concomitant Medication		
Subject	Date of First Injection	Start Date	Stop Date	Prior/Concomitant
100101	16-Oct-2019	14-Sep-2019	16-Oct-2019	Concomitant

NB. Stopped on the same date as the date of first vaccine so concomitant medication.

Example 3

		Prior/Concomitant Medication		
Subject	Date of First Injection	Start Date	Stop Date	Prior/Concomitant
100101	16-Oct-2019	14-Sep-2019	ONGOING	Concomitant

NB. Started prior to date of first vaccine and ongoing, so concomitant.

Example 4

		Prior/Concomitant Medication		
Subject	Date of First Injection	Start Date	Stop Date	Prior/Concomitant
100101	16-Oct-2019	16-Oct-2019	ONGOING	Concomitant

NB. Started on the date of first vaccine and ongoing, so concomitant.

Example 5

		Prior/Concomitant Medication		
Subject	Date of First Injection	Start Date	Stop Date	Prior/Concomitant
100101	16-Oct-2019	20-Oct-2019	21-Oct-2019	Concomitant

NB. Started and stopped after the date of first vaccine, so concomitant.

Please refer to section 8.11 below for how to handle partial or missing dates in the assessment of whether or not a medication was taken prior to or concomitantly with study treatment.

8.6 Treatment Compliance

Compliance (%) will be calculated as:

Compliance = (Number of Immunizations Received/ Expected Total Immunizations) * 100.

Example 1
If a subject received 3 of the 4 injections: Compliance = (3/4)*100 = 75%.

8.7 Treatment Exposure

Duration of Exposure (days) will be calculated as:

Duration of Exposure = Date of Last Administration of Study Vaccine – Date of First Administration of Study Vaccine + 1.

Strictly Confidential. © 2020, [redacted] All rights reserved.

8.8 Treatment Emergent Adverse Event

A treatment emergent adverse event (TEAE) is defined as:

- any AE that has an onset on or after the first dose of investigational product
- or any pre-existing condition that has worsened on or after the first dose of investigational product
- or any AE that starts prior to the end of the designated follow-up period, i.e. 24 weeks (6 months) after the end of the treatment period.

If applicable, the assessment of whether or not an AE is treatment emergent should include both the date and time relative to the first administration of study vaccine.

Example 1

Subject	Date of First Injection	AE Start Date	AE Stop Date	TEAE?
100101	16-Oct-2019	14-Sep-2019	15-Oct-2019	No

NB. AE stopped prior to the first administration of vaccine, not TEAE.

Example 2

Subject	Date of First Injection	AE Start Date	AE Stop Date	TEAE?
100101	16-Oct-2019	18-Oct-2019	21-Oct-2019	Yes

NB. AE started after the first administration of vaccine, TEAE.

Please refer to SAP Section 8.11, below for how to handle partial or missing dates in the assessment of whether or not an event is a TEAE.

8.9 Adverse Event Relatedness

Relationship to study treatment is recorded as unrelated, unlikely related, possibly related and probably related on the eCRF. All relationships reported as unrelated or unlikely related will be categorized as “Not Related”, while possibly related and probably related will be categorized as “Related” in the AE tables. AE listings will present relationship to study treatment as available in the raw data / eCRF.

8.10 Abnormal Laboratory Values

Clinically significant abnormal laboratory findings or other abnormal assessments that are detected after the first administration of study vaccine or that are present at baseline and worsen following the first administration of study vaccine are included as AEs or SAEs. The investigator should exercise medical and scientific judgement in deciding whether an abnormal laboratory finding is clinically significant.

8.11 Handling of Partial or Missing Dates

For the purpose of calculating durations there will be no imputations made for partial or missing dates. Where duration of an event is calculated based on actual dates and any of the dates used in the computational algorithm are partial or missing, the duration will also be missing.

For the purpose of assigning medical histories as ‘past or current’, AEs as ‘treatment-emergent’ and therapies as ‘prior or concomitant’ (partially) missing start and stop dates will not be imputed.

Strictly Confidential. © 2020, [redacted] All rights reserved.

In general, medical histories will be considered current, unless there is evidence in the (partial) dates available that the medical condition stopped prior to the first administration of study vaccine. The same rules will be applied to partial or missing AD diagnosis dates.

Example 1

		Medical History		
Subject	Date of First Injection	Start Date	Stop Date	Past/Current
100101	16-Oct-2019	14-Sep-2019	xx-Oct-2019	Current

NB. Month of stop date is the same as the month of the first injection so medical history is assumed to be current.

Example 2

		Medical History		
Subject	Date of First Injection	Start Date	Stop Date	Past/Current
100101	16-Oct-2019	xx-Sep-2019	xx-xxx-2019	Current

NB. Year of stop date is the same as the year of the first injection so medical history is assumed to be current.

Example 3

		Medical History		
Subject	Date of First Injection	Start Date	Stop Date	Past/Current
100101	16-Oct-2019	14-Sep-2019	xx-xxx-xxxx	Current

NB. Stop Date missing and medication not checked as ongoing, so medical history is assumed to be current.

Example 4

		Medical History		
Subject	Date of First Injection	Start Date	Stop Date	Past/Current
100101	16-Oct-2019	xx-Oct-2019	15-Oct-2019	Prior

NB. Although start date partial, stop date is prior to the date of the first injection, so medical history is assumed to be past.

In general, an AE will be considered treatment-emergent, unless there is evidence in the (partial) dates available that it was not treatment-emergent. In particular, in case of missing start dates of AEs, these will be considered treatment-emergent, unless the stop date of the AE is prior to the first administration of study vaccine. In the case of partially missing start dates, the AE will be considered treatment-emergent, unless the information from the partial dates clearly shows that the AE was not treatment-emergent.

Example 1

Subject	Date of First Injection	AE Start Date	AE Stop Date	TEAE?
100101	16-Oct-2019	14-Sep-2019	xx-Sep-2019	No

NB. Month AE stopped is prior to the month of the first injection so AE is not TEAE.

Example 2

Subject	Date of First Injection	AE Start Date	AE Stop Date	TEAE?
100101	16-Oct-2019	xx-Oct-2019	xx-Oct-2019	Yes

NB. Month of AE start and stop is the same as the month of the first injection so AE assumed to be TEAE.

In general, medications will be considered concomitant, unless there is evidence in the (partial) dates available that it was stopped prior to the first administration of study vaccine.

Example 1

		Prior/Concomitant Medication		
Subject	Date of First Injection	Start Date	Stop Date	Prior/Concomitant
100101	16-Oct-2019	14-Sep-2019	xx-Oct-2019	Concomitant

NB. Month of stop date is the same as the month of the first injection so medication is assumed to be concomitant.

Example 2

Prior/Concomitant Medication

Strictly Confidential. © 2020, [redacted] All rights reserved.

Subject	Date of First Injection	Start Date	Stop Date	Prior/Concomitant
100101	16-Oct-2019	xx-Sep-2019	xx-xxx-2019	Concomitant

NB. Year of stop date is the same as the year of the first injection so medication is assumed to be concomitant.

Example 3

		Prior/Concomitant Medication		
Subject	Date of First Injection	Start Date	Stop Date	Prior/Concomitant
100101	16-Oct-2019	14-Sep-2019	xx-xxx-xxxx	Concomitant

NB. Stop Date missing and medication not checked as ongoing, so medication is assumed to be concomitant.

Example 4

		Prior/Concomitant Medication		
Subject	Date of First Injection	Start Date	Stop Date	Prior/Concomitant
100101	16-Oct-2019	xx-Oct-2019	15-Oct-2019	Prior

NB. Although start date partial, stop date is prior to the date of the first injection, so medication is assumed to be prior.

8.12 Handling of Unscheduled Visits Data and Unscheduled Assessments

If scheduled and unscheduled assessments are available for the same parameter within a single visit, then only the last scheduled assessment will be used in the analysis. In case only unscheduled visit and no planned visit is conducted for particular parameters, the unscheduled visit may be mapped to a scheduled visit, only if it is within the visit window as defined in the protocol, and then summarized. This applies, for example, to visits performed by telephone rather than onsite due to Covid-19 lockdown / travel restriction. Scheduled (planned) and unscheduled visits data will be included in listings and the one used in the analysis will be flagged.

The visit window allowed for each particular parameter is specified in the Schedule of Assessments included in the protocol.

In the ECG external data transfer set, ECG repetitions are referenced as unscheduled visits. In such cases, the following rules will be applied:

- The last ECG measurement will be taken for analysis presented in summary tables.
- The listing will show all ECG repetitions at the respective assigned visit.

In case the ECG was done at a scheduled visit where an ECG was not planned, the following rules will be applied in the analysis:

- The ECG results will not be taken into consideration for summary tables.
- The corresponding listing will show these evaluations and flag them with a respective footnote.

8.13 Handling of Missing Adverse Event Severity and Relationship

If Severity is missing the event will be assumed to be severe. If relationship is missing the event will be assumed to be the most related, i.e. probably related.

8.14 Imputation of Titers Below the Limit of Quantification (BLQ)

The Lower Limit of Quantification (LLOQ) will be provided in the immunogenicity vendor data transfer. For titers BLQ the reported result of the LLOQ will be replaced with an imputed value of half the LLOQ, i.e. 0.5*LLOQ. Listings will include the original result as provided by the vendor.

Strictly Confidential. © 2020, [redacted] All rights reserved.

8.15 Imputation of Biomarker Values Below the Limit of Quantification (BLQ)

The Lower Limit of Quantification (LLOQ) will be provided in the biomarker vendor data transfers. For values BLQ the reported result will be replaced with an imputed value of the LLOQ. Listings will include the original result as provided by the vendor.

8.16 Geometric Mean Titer

The Geometric Mean Titer (GMT) is calculated by taking the antilogarithm of the mean of the log10 transformed titers. Antibody titers BLQ will be imputed as outlined in Section 8.14.

8.17 Normal Approximation Confidence Interval (CI) for the Geometric Mean Titer

Lower Limit=antilogarithm of LL, where LL = Mean of log values – 1.96 x Standard Error of log values.

Upper Limit=antilogarithm of UL, where UL = Mean of log values + 1.96 x Standard Error of log values,

where the Standard Error of log transformed values is the Standard Deviation (calculated on log transformed data) divided by the square root of the number of observations.

8.18 Antibody Response

8.18.1 Anti-pTau IgG, anti-Tau IgG, anti-pTau IgM, anti-Tau IgM, anti-ePHF IgG and anti-CRM IgG

The determination of antibody response being negative/positive will be done using a threshold factor defined from samples from human donors (obtained during the validation of each assay) by [redacted] (see the table below). The baseline titer, as defined in section 8.2, will be multiplied with this threshold factor. The corresponding value will then be compared to post-baseline results. If the post-baseline result is greater than or equal to (≥) this value, the antibody response will be considered to be positive, otherwise it will be termed negative. For example, if the baseline titer is 524 and the threshold factor for a response is 2 we have the following: If a post-baseline visit titer is ≥ 524*2=1048 then the antibody response at this visit will be considered positive; if the post-baseline visit titer is <1048 then the antibody response at this visit will be considered negative.

ASSAY	ANALYTE	RESULT	Threshold Factor	UNIT
[redacted]	Anti-pTau IgG	Rounded value. Rounded as per protocol (e.g. 3 significant digits).	1.81	AU/mL
[redacted]	Anti-Tau IgG	Rounded value. Rounded as per protocol (e.g. 3 significant digits)	3.38	AU/mL
[redacted]	Anti-pTau IgM	Rounded value. Rounded as per protocol (e.g. 3 significant digits)	2.11	AU/mL
[redacted]	Anti-Tau IgM	Rounded value. Rounded as per protocol (e.g. 3 significant digits)	3.20	AU/mL
[redacted]	Anti-ePHF IgG	Rounded value. Rounded as per protocol (e.g. 3 significant digits)	2.21	AU/mL
[redacted]	Anti-ePHF Avidity	Rounded value. Rounded as per protocol (e.g. 3 significant digits) $, *	N.Ap.	N.Ap.#
[redacted]	Anti-CpG IgG	Rounded value. Rounded as per protocol (e.g. 3 significant digits)	Cut-point is applicable &	AU/mL
[redacted]	Anti-T50 IgG	Rounded value. Rounded as per protocol (e.g. 3 significant digits)	Cut-point is applicable &	AU/mL
[redacted]	Anti-CRM IgG	Rounded value. Rounded as per protocol (e.g. 3 significant digits)	2.23	AU/mL

Avidity will be calculated as a ratio and hence no unit is applicable
$ Only Post treatment results are reported for anti-ePHF avidity (i.e. Screening and Visit 1 are not applicable) of samples that are positive for anti-ePHF IgG antibodies.
* ePHF avidity will not be reported if ePHF IgG is negative or if ePHF IgG average of multiple runs is used and ePHF avidity is considered unreportable by [redacted]/the sponsor. In both cases blank result value will be populated, “NVR” (no valid result) will be

Strictly Confidential. © 2020, [redacted] All rights reserved.

included in the Status column and a reason will be added in the comments ("ePHF IgG negative" or "ePHF IgG average of multiple runs").
& determination of positive or negative samples is made by [redacted]. Numeric values are only reported on samples positive for antibodies. In case the concentrations are undetectable or samples are evaluated as negative, "BLQ" (Below Limit of Quantification) is reported.

For layout/reading purpose, shorter adjuvant names have been used, i.e. CRM in place of CRM197.

8.18.2 Anti-CpG IgG and anti-T50 IgG

A numeric value of antibody titer will only be reported to [redacted] on samples termed positive by [redacted] based on the plate specific cut-point. If a sample has signal below the plate-specific cut-point, it is considered negative and [redacted] will report it as "BLQ" (Below Limit of Quantification). To be considered positive, a sample should have a signal equal or greater than the plate specific cut-point.

For layout/reading purpose, shorter adjuvant names have been used, i.e. CpG in place of CpG7909.

8.19 Avidity index

The avidity index will be reported by [redacted] based on the ratio between the concentration of anti-enriched Paired Helical Filaments [anti-ePHF] IgG measured in saturated (high coating) and unsaturated (low coating) conditions. The avidity index will only be reported for post baseline visits and if the anti-ePHF IgG antibody response measured on high coating is positive (see definition of positive/negative response in section 8.17.1).

8.20 Area Under the Curve (AUC)

Area Under the Curve (AUC) (AU/mL*h) of anti-pTau and anti-Tau in serum will be calculated by using the trapezoidal rule up to Week 50 as well as up to Week 74.

AUC will also be provided for all other immune response parameters including anti-ePHF IgG (high coating).

8.21 Peak Concentration

Peak concentration (AU/mL) of immune response parameters is defined as the maximum concentration observed post dose.

9.0 **Analysis Populations**

In the event that a subject randomized to receive ACI-35.030 within Cohort 1 received any Placebo at any of the 4 injections they will still be presented in the ACI-35.030 arm unless they received only Placebo in which case they will be presented in the Placebo arm. In the event that a subject randomized to receive Placebo received one or more doses of ACI-35.030, the subject will be presented in the ACI-35.030 treatment arm. Same consideration will be applied to subjects from Cohort 2 with JACI-35.054.

9.1 Enrolled

The enrolled population is defined as all subjects who signed informed consent.

9.2 Intention-to-Treat

The intention-to-treat (ITT) population is defined as all randomized subjects who received at least one dose of the drug during the study, and will be used for presentation of primary, secondary and exploratory endpoints and for description of baseline characteristics.

Strictly Confidential. © 2020, [redacted] All rights reserved.

The ITT population will be analyzed and presented according to the treatment arm in which the subject was randomized regardless of the number of injections that the subject actually received.

9.3 Per Protocol

The per-protocol (PP) population is defined as all subjects from the ITT population who do not have any important protocol deviations which might significantly affect the completeness, accuracy, and/or reliability of the study data or that may have an impact on the immune response assessment (a subset of all important protocol deviations which will be considered critical), and will be used as a supportive analysis of the primary and secondary efficacy endpoints for the final analysis.

The PP population will be defined during the course of the study ahead of each interim analysis but semi-unblinded IA TFLs based on the PP analysis set will not be prepared and delivered to the Sponsor Data Review Committee in case there is a risk of unblinding. Such instances could be for example if high number of subjects missed visits and/or treatment (vaccine) administration even though this might not be considered a major protocol deviation.

Analysis based on the PP population per timepoint might also be presented. This will allow to include all the participants in the summary tables and figures who do not have any major protocol deviations that are expected to impact the immunogenicity outcomes up to a corresponding timepoint.

All the protocol deviations will be identified and recorded in the Predictivv Study Operation (PSO) [redacted] system. The subset of important protocol deviations leading to exclusion from the PP set will be identified prior to the unblinding of the study for the final analysis and will include at least the following:

- Subjects who entered the study even though they did not satisfy the eligibility criteria
- Those who developed withdrawal criteria during the study but were not withdrawn
- Those who received the wrong treatment or incorrect dose
- Those who received any excluded concomitant treatment, see further details below

Subjects receiving acetylcholinesterase inhibitor and/or memantine should have been on a stable dosage for at least 3 months prior to baseline.

The following treatments are not permitted throughout the study:

- Anticoagulants or antiplatelet drugs, except aspirin at doses of 100 mg daily or lower
- Standard of care immunizations during the period ranging from 2 weeks before or after any administration of study vaccine to avoid potential interference
- Hydralazine, procainamide, quinidine, isoniazide, TNF-inhibitors, minocycline
- Diltiazem unless subject is on stable dose 3 months prior to screening
- Any investigational or non-investigational drugs given as part of another clinical trial

The PP analysis population will be re-defined at the end of the study, incorporating important PDs identified during the whole study duration including those listed above following review of the PDs by the study team, prior to unblinding for the final analysis. The study team ([redacted] and AC Immune) may decide to re-evaluate the above listed PDs on a case by case basis with regards to the exclusion of subjects who reported such PDs from the PP population.

In the case that the ITT population is identical to the PP population, only the ITT analysis population will be reported.

9.4 Safety Population

The Safety Population is defined as all randomized subjects who received at least one dose of IMP. Subjects will be analyzed based on the study treatment actually taken. In the event that no subject

Strictly Confidential. © 2020, [redacted] All rights reserved.

received incorrect medication the ITT and Safety populations will be the same. Consequently, all safety TFLs will be presented for the ITT population only noting that the populations are the same.

In the event that a subject randomized to receive ACI-35.030 within Cohort 1 received any Placebo at any of the 4 injections they will still be presented in the ACI-35.030 arm unless they received only Placebo in which case they will be presented in the Placebo arm. In the event that a subject randomized to receive Placebo received one or more doses of ACI-35.030, the subject will be presented in the ACI-35.030 treatment arm. If this occurs the TFL shells will be amended to present efficacy related parameters by the ITT population and Safety related parameters by the Safety population.

10.0 **Interim Analyses**

The following interim analyses may be conducted in this study (also refer to Figure 1 for schematic overview under Section 6.0). Interim analyses of safety, tolerability and immunogenicity data may be conducted in each sub-cohort, including expanded sub-cohorts as given below.

10.1 Cohort 1 with ACI-35.030

The following interim analyses may be conducted in this study for cohort 1:

Time point	Sub-cohorts	Scope / Objective
Visit 4 [Week 10], i.e. 2 to 4 weeks after the second injection	All available subjects in Sub-cohort 1.1, 1.2 and 1.3	Evaluate the safety, tolerability and immunogenicity of 300 µg, 900 µg and 1800 µg ACI-35.030 to dose escalate the sub-cohorts 1.2 and 1.3 and/or potentially expand the recruitment in the sub-cohort 1.1, 1.2 and/or 1.3. Biomarker results may also be reviewed.
Visit 6 [Week 26]*, i.e. 2 to 4 weeks after the third injection	All available subjects in Sub-cohort 1.1, 1.2 and 1.3	Evaluate the safety, tolerability and immunogenicity of 300 µg, 900 µg and 1800 µg ACI-35.030 at this time point and potentially expand the recruitment in the sub-cohort 1.1, 1.2 and/or 1.3. Biomarker results may also be reviewed.
Visit 9 [Week 50], i.e. 2 to 4 weeks after the last injection at Week 48	All available subjects in Sub-cohort 1.1, 1.2 and 1.3	Evaluate the safety, tolerability and immunogenicity of 300 µg, 900 µg and 1800 µg ACI-35.030 at this time point, and potentially expand the recruitment in the sub-cohort 1.1, 1.2 and/or 1.3. Biomarker results may also be reviewed.
Visit 11 [Week 74]	All available subjects in Sub-cohort 1.1, 1.2 and 1.3	Evaluate the safety, tolerability and immunogenicity of 300 µg, 900 µg and 1800 µg ACI-35.030 at this time point and potentially expand the recruitment in the sub-cohort 1.1, 1.2 and/or 1.3. Biomarker results may also be reviewed.

* Note that this analysis was not conducted for sub-cohort 1.1 since the time point was reached in this sub-cohort before this analysis was implemented in protocol version 5.

Strictly Confidential. © 2020, [redacted] All rights reserved.

10.2 Cohort 2 with JACI-35.054

The following interim analyses may be conducted in this study for cohort 2:

Time point	Sub-cohorts	Scope / Objective
Visit 4 [Week 10], i.e. 2 to 4 weeks after the second injection	All available subjects in Sub-cohort 2.1, 2.2 (optional) and 2.3 (optional)	Evaluate the safety, tolerability and immunogenicity of 15 µg, 60 µg and 150 µg JACI-35.054 to dose escalate the sub-cohorts 2.2 and 2.3 and/or potentially expand the recruitment in the sub-cohort 2.1 and/or 2.2 and/or 2.3. Biomarker results may also be reviewed.
Visit 6 [Week 26], i.e. 2 to 4 weeks after the third injection	All available subjects in Sub-cohort 2.1, 2.2 (optional) and 2.3 (optional)	Evaluate the safety, tolerability and immunogenicity of 15 µg, 60 µg and 150 µg JACI-35.054 at this time point and potentially expand the recruitment in the sub-cohort 2.1, 2.2 and/or 2.3. Biomarker results may also be reviewed.
Visit 9 [Week 50], i.e. 2 to 4 weeks after the last injection at Week 48	All available subjects in Sub-cohort 2.1, 2.2 (optional) and 2.3 (optional)	Evaluate the safety, tolerability and immunogenicity of 15 µg, 60 µg and 150 µg JACI-35.054 at this time point, and potentially expand the recruitment in the sub-cohort 2.1, 2.2 and/or 2.3. Biomarker results may also be reviewed.
Visit 11 [Week 74]	All available subjects in Sub-cohort 2.1, 2.2 (optional) and 2.3 (optional)	Evaluate the safety, tolerability and immunogenicity of 15 µg, 60 µg and 150 µg JACI-35.054 at this time point and potentially expand the recruitment in the sub-cohort 2.1, 2.2 and/or 2.3. Biomarker results may also be reviewed.

10.3 Cohort 3 with ACI-35.030 and JACI-35.054

The following interim analyses may be conducted in this study for cohort 3:

Time point	Sub-cohorts	Scope / Objective
Visit 4 [Week 10], i.e. 2 to 4 weeks after the second injection	All available subjects in Sub-cohort 3.1, 3.2 (optional) and 3.3 (optional)	Evaluate the safety, tolerability and immunogenicity of ACI-35.030 and JACI-35.054 (dosages to be determined) to dose escalate the sub-cohorts 3.2 and 3.3 and/or potentially expand the recruitment in the sub-cohort 3.1, 3.2 and/or 3.3. Biomarker results may also be reviewed.
Visit 6 [Week 26], i.e. 2 to 4 weeks after the third injection	All available subjects in Sub-cohort 3.1, 3.2 (optional) and 3.3 (optional)	Evaluate the safety, tolerability and immunogenicity of ACI-35.030 and JACI-35.054 (dosages to be determined) at this time point and potentially expand the recruitment in the sub-cohort 3.1, 3.2 and/or 3.3. Biomarker results may also be reviewed.

Strictly Confidential. © 2020, [redacted] All rights reserved.

Time point	Sub-cohorts	Scope / Objective
Visit 9 [Week 50], i.e. 2 to 4 weeks after the last injection at Week 48	All available subjects in Sub-cohort 3.1, 3.2 (optional) and 3.3 (optional)	Evaluate the safety, tolerability and immunogenicity of ACI-35.030 and JACI-35.054 (dosages to be determined) at this time point, and potentially expand the recruitment in the sub-cohort 3.1, 3.2 and/or 3.3. Biomarker results may also be reviewed.
Visit 11 [Week 74]	All available subjects in Sub-cohort 3.1, 3.2 (optional) and 3.3 (optional)	Evaluate the safety, tolerability and immunogenicity of ACI-35.030 and JACI-35.054 (dosages to be determined) at this time point and potentially expand the recruitment in the sub-cohort 3.1, 3.2 and/or 3.3. Biomarker results may also be reviewed.

10.4 Interim Analysis Procedures

For each IA a data lock point (DLP) will be employed. Data cuts will be performed based on the DLP for each IA including all data collected until this date. Every effort will be made to ensure that all data for each IA for the cohorts of interest, prior to and including the DLP are complete, clean (all data clarifications have been made) and coded. Data Review Meetings will be planned before each IA in order to verify that no important data issues which require resolution remain ahead of conducting an IA.

In addition to the above IAs, safety data will be reviewed on a quarterly basis by a Data Safety Monitoring Board (DSMB). The scope and frequency of the meetings are detailed in the DSMB charter. The materials to be provided for each meeting will be outlined in the DSMB SAP and TFL Shells and Appendix 2 Tables, Figures, Listings of this document.

For the first DSMB the committee will receive J-review objects for review as programmatic TFLs will not be available at this time due to limited data availability.

For each IA, outlined above, the DSMB will receive the same safety/tolerability data as for a quarterly DSMB meeting. A separate semi-unblinded Sponsor Data Review Committee (SDRC) will receive the semi-unblinded (i.e. recoded) Immunogenicity Data. Please refer to Section 6.3 for further details. The scope and responsibilities of this committee will be outlined in the SDRC charter. The list of TFLs to be provided to the Sponsor Data Review Committee for each IA are included in Appendix 2 Tables, Figures, Listings of this document.

Provision of these unblinded outputs will be handled by a separate unblinded team within [redacted] The outputs for each DSMB and IA will be stored in a secure access-controlled area at [redacted] accessible only by the Independent Reporting Team. These unblinded outputs will be provided to named individuals only within the DSMB and Sponsor Data Review Committee via a highly secure collaborative workspace.

Available biomarker data may also be reviewed during any of these IAs. The IAs described above for the 3 cohorts may also be performed for any expanded sub-cohort.

Additional IAs to review the sustainability of immune response data may be conducted between weeks 26 and 50 and between weeks 50 and 74. If the post-injection period is extended in any sub-cohort, IA of immunogenicity data may also be conducted at Visit 13 (Week 98), and optionally at Visit 12 (Week 86).

Independently of the above-mentioned interim analyses, the DSMB will perform regular reviews of the safety and tolerability data throughout the study.

Strictly Confidential. © 2020, [redacted] All rights reserved.

Rationale and Scope of IAs

The objectives of the above mentioned IAs in the sub-cohorts (whether expanded or not) is to ensure a regular review of safety, tolerability and immunogenicity with the support of optional biomarker data. A decision to escalate to the next dose in one given sub-cohort may be taken once IA data of the previous dose are available from Visit 4 (Week 10) onwards and pending prior DSMB approval. A decision to expand a sub-cohort may be taken based on satisfactory review of safety data by the DSMB and analysis of immunogenicity IA data of this sub-cohort dose from Visit 4 (Week 10) onwards. The IA at the end of the optional extended post-injection period is intended to monitor the durability of the immune response and any biomarker changes in blood.

In case data cut points occur within a short interval of time, interim analysis may be combined as deemed appropriate.

Stopping Rules

Stopping at the Individual Subject Level
Dosing will be suspended at the individual level if a Serious Adverse Event (SAE) considered related to study medication is observed. Dosing will also be suspended at the individual level in the case of adverse reactions of moderate intensity persisting for more than 2 weeks or of severe intensity until further evaluation by the DSMB. Dosing may also be suspended in the case of adverse reactions of shorter duration or lower intensity if the investigator considers this clinically indicated.

Stopping or Suspension of Dosing
Dosing will be suspended in all subjects if meningoencephalitis is observed in any subject, until further evaluation by the DSMB. The Competent Authority in each participating country will be informed of such a temporary halt to dosing following a case of meningoencephalitis. Dosing will not recommence until a protocol substantial amendment is submitted to the Competent Authorities requesting trial restart.

All SAEs will be reported to the DSMB who will evaluate them and give recommendations whether dosing in the cohort as a whole can continue. Where two or more clinically relevant non-serious adverse reactions of moderate intensity persisting for more than 2 weeks or severe intensity are observed, this will be drawn to the attention of the DSMB who will evaluate the cases and determine whether dosing can continue in the cohort as a whole.

10.5 Additional Clarifications Preventing Unblinding During IAs

In case of missing values (e.g. drop-out) or a protocol deviation that is expected to impact the immunogenicity outcome, the complete subject-level profiles and data might not be provided to limit the risk of potential unblinding but rather to provide individual data up to any missing value and aggregate statistics thereafter. It may be discussed and agreed with AC Immune on a case by case basis using a risk-based approach.

The definitions of the analysis populations used will remain as specified in Section 9.0, but footnotes will be included to clarify the selection criteria and justify inclusion of subjects in each particular output.

10.6 Interim Analysis Conduct

Interim analyses will be performed by [redacted] and by [redacted] working in collaboration. Additional specific tasks associated with the joint effort in this partnership will be included in a separate document (Transition Report).

[redacted] will follow a separate SAP prepared by their internal team, i.e. “SAP for semi-unblinded interim analyses of the immunogenicity data” and [redacted] will provide [redacted] with the ADaM datasets which will be used for the production of each interim analysis of immunogenicity data. [redacted] and [redacted] SAPs are complementary and refer to one another as needed for the interim analyses deliveries.

Strictly Confidential. © 2020, [redacted] All rights reserved.

11.0 **Statistical Methods**

All analyses will use SAS® version 9.4 or higher. Descriptive summaries will be tabulated by treatment group (ACI 300µg, ACI 900µg, ACI 1800µg, JACI 15µg, JACI 60µg). Where applicable, all subjects from each main cohort will be summarized together (excluding immunogenicity and biomarker TFLs).

Categorical data will be presented using counts and percentages, with the number of subjects in each category as the denominator for percentages. Percentages will be rounded to one decimal place except 100% will be displayed without any decimal places and percentages will not be displayed for zero counts.

Continuous data will be summarized using the number of observations (n), mean, standard deviation (SD), median, first quartile, third quartile, minimum, and maximum. Minimum and maximum will be rounded to the precision of the original data. Mean and median will be rounded to 1 decimal place greater than the precision of the original data. The SD will be rounded to 2 decimal places greater than the precision of the original data, up to a maximum of 3 decimal places.

A sensitivity analysis may be carried out at the end of the study if the number of patients impacted by the COVID-19 pandemic is large (e.g. >10% missed injections due to COVID-19) and thought to be impacting the efficacy and safety endpoints. This sensitivity analysis may exclude the patients affected by the pandemic.

11.1 Subject Disposition

The number of subjects screened; the number and percentage of subjects screen failed, randomized, treated, randomized and not treated and enrolled into each analysis population (ITT and PP) will be summarized by treatment group and overall for subjects in the enrolled population [**TFL Shell Table 14.1.1.1**].

For the ITT population the number and percentage of subjects having completed or prematurely discontinued the treatment and/or the study will be presented by treatment arm and overall. Primary reason for premature discontinuation (Adverse event, Death, Lost to Follow-up, Protocol Deviation, Study Subject Withdrawal by Caregiver, Withdrawal by Subject and Other) will be summarized [**TFL Shell Table 14.1.1.2**].

The number and percentage of subjects providing data at each of the study visits will be presented by treatment arm and overall for the ITT population [**TFL Shell Table 14.1.1.4**].

Randomization assignments, including date of informed consent, details pertaining to which treatment a subject was assigned to and re-consenting details will be listed [**TFL Shell Listing 16.1.7**]. Date of completion of the study and IMP and reasons for discontinuation of the study or IMP will be listed [**TFL Shell Listing 16.2.1.1, 16.2.1.2**]. Details of the assignments of subjects to study populations (Safety/ITT and Per Protocol) will be listed for all enrolled subjects [**TFL Shell Listing 16.2.1.1, 16.2.1.2**]. Details of the visits (conducted and missed) provided by each subject will be listed for the Intention-to Treat Population [**TFL Shell Listing 16.2.3.2**].

11.2 Protocol Deviations and Violations

The number and percentage of subjects with important protocol deviations will be presented by treatment group and overall for the ITT population [**TFL Shell Table 14.1.1.3.1**].

All protocol deviations will be listed for the Intention-to Treat Population [**TFL Shell Listing 16.2.2.1**].

Any COVID-19-specific protocol deviations will be summarized [**TFL Shell Table 14.1.1.3.2**] and listed separately [**TFL Shell Listing 16.2.2.2**]. The number of patients missing visits or visits with alternative contact due to the COVID-19 pandemic will be summarized [**TFL Shell Table 14.1.1.3.3**].

Strictly Confidential. © 2020, [redacted] All rights reserved.

11.3 Demographic and Baseline Characteristics

The following demographic and baseline variables will be summarized by treatment group (and overall) for the ITT population: age (years), height (cm), weight (kg), BMI(kg/m^2), Mini Mental State Examination (MMSE) (total score) and time since initial diagnosis of Alzheimer's Disease (AD) will be summarized as continuous data. Sex, race, ethnicity, child-bearing potential (yes, no) and number of family members with AD diagnosis will be summarized as categorical data [**TFL Shell Table 14.1.2**].

Demographic data will also be listed [**TFL Shell Listing 16.2.4.1**].

Past and current medical histories, as defined in section 8.4, by Medical Dictionary for Regulatory Authorities (MedDRA) latest version System Organ Class (SOC) and Preferred Term (PT) will be summarized by treatment group and overall for the ITT population [**TFL Shell Table 14.1.3.1, 14.1.3.2**].

Medical history data will also be listed [**TFL Shell Listing 16.2.4.2**].

11.4 Treatments

11.4.1 Study Drug Exposure

The following details will be summarized with regards to study drug compliance and exposure:

- The number and percentage of subjects receiving each of 4 planned injections (including the visits at which injections were received)
- Compliance (%), as defined in section 8.6
- The duration of exposure (days) as defined in section 8.7

All the above will be summarized by treatment group and overall for the Intention-to Treat Population [**TFL Shell Table 14.1.6**].

Administration information of the IMP at the scheduled visits will be listed, including information of any noncompliance to study treatment (e.g. incorrect dose injected, treatment interruption) [**TFL Shell Listing 16.2.5.1**] and treatment compliance [**TFL Shell Listing 16.2.5.2**].

11.4.2 Prior and Concomitant Medications

Prior and concomitant medications will be coded using the World Health Organization dictionary (WHO) Drug Dictionary (WHODD) latest version. Each medication will be assigned a Preferred Term (PT). Please refer to the Coding Convention document for further details on the coding of medications.

The number and percentage of subjects with prior and concomitant medication WHO Drug Dictionary PTs will be summarized by treatment group and overall for the Intention-to Treat Population [**TFL Shell Table 14.1.4, 14.1.5**].

All prior and concomitant medications and procedures will be listed [**TFL Shell Listing 16.2.4.3, 16.2.4.4**].

11.5 Safety Analyses

The primary safety endpoints are type, frequency, and intensity of AEs evaluated until the end of treatment and change from baseline in the parameters of physical and neurological examination, vital signs, MRI imaging, Electrocardiogram (ECG), routine hematology and biochemistry evaluation in blood and urine, evaluation of autoimmune antibodies in blood and inflammatory markers in blood and CSF. Other cognitive and clinical variables related to safety are provided in Section 5.3.5.1 and Section 5.3.5.3 of the study protocol. Continuous and categorical laboratory data will be summarized separately.

11.5.1 Adverse Events

All adverse events (AEs) will be coded using the most up-to-date version of the MedDRA dictionary. As AEs are collected starting from the moment of the Informed Consent Form (ICF) signature, AEs and

Strictly Confidential. © 2020, [redacted] All rights reserved.

treatment emergent adverse events (TEAEs) will be discriminated. Please refer to Section 8.8 for the definition of TEAEs.

An overview of AEs and TEAEs will be given in terms of the number and percentage of subjects and number of AEs and TEAEs per treatment group (and overall) for the Safety Population within each of the following categories:

- AEs
- TEAEs
- Serious AEs (SAEs)
- Serious TEAEs
- Severe adverse events
- Severe TEAEs
- Adverse drug reactions
- Serious adverse drug reactions
- AEs leading to discontinuation of study treatment
- AEs leading to discontinuation from the study
- TEAEs leading to discontinuation from the study
- AEs with an outcome of death
- TEAEs with an outcome of death

[**TFL Shell Table 14.3.2.1**]

Selected TEAE categories from above will also be summarized by MedDRA SOC and PT [**TFL Shell Table 14.3.2.2, 14.3.2.3, 14.3.2.4, 14.3.2.5, 14.3.2.6, 14.3.2.7, 14.3.2.8**], according to severity (mild, moderate, severe) [**TFL Shell Table 14.3.2.9**] and according to relationship to drug [**TFL Shell Table 14.3.2.10**].

Most frequent TEAEs will be presented by Treatment Group in a Table [**TFL Shell Table 14.3.2.11**] and a bar chart [**TFL Shell Figure 14.3.1**], where most frequent TEAEs are defined events which occur in at least 10% of subjects in any treatment group. Time to first SAE since first injection (in days) will be presented graphically as a bar chart per cohort with subjects as individual points [**TFL Shell Figure 14.3.2**].

All AEs [Non-Injection Site Reactions (ISR) and ISRs] will be listed [**TFL Shell Listing 16.2.7.1 to 16.2.7.6.3**].

11.5.2 Laboratory Data

The following safety laboratory data will be collected for this study:

- Routine hematology [red blood cell count, hemoglobin, hematocrit, red cell indices, white blood cell count (including differential), platelet count], coagulation [prothrombin time (PT), international normalized ratio (INR), activated partial thromboplastin time (APTT)] and biochemistry evaluations [Sodium, potassium, chloride, urea, creatinine, calcium, inorganic phosphate, glucose, total bilirubin, total protein, albumin, aspartate aminotransferase, alanine aminotransferase, alkaline phosphatase, Gamma-glutamyl Transferase (Gamma GT), creatine kinase, cholesterol, triglycerides and uric acid and C-Reactive Protein (CRP)] in blood
- Routine biochemistry evaluations in urine [pH, protein, glucose, ketones and blood]
- Inflammatory markers in blood [CRP] and Cerebrospinal Fluid (CSF) [protein, CSF/serum albumin ratio, IgG + IgM Index, Oligoclonal bands, glucose, cell count and differential count]
- Autoimmune antibodies in blood [Anti-nuclear Antibodies (ANA), Anti-double stranded DNA Antibodies (Anti-dsDNA)]
- Serology (HIV-1/2 Antibody, Hepatitis B Virus Surface Antigen, Hepatitis C Virus Antibody, Rapid Plasma Reagin (syphilis))

Strictly Confidential. © 2020, [redacted] All rights reserved.

- Other laboratory tests (U1-nRNP Ab, Anti-Ro (SS-A Ab), Anti-La (SS-B Ab), Complement C1q Antibody, C3c, Complement C4)

The actual values and change from baseline, see Sections 8.2 and Section 8.3 for definitions, for all continuous laboratory parameters above will be summarized per treatment group, by visit [Baseline, Visit 2 (Week 2), Visit 3 (Week 8), Visit 4 (Week 10), Visit 5 (Week 24), Visit 6 (Week 26), Visit 7 (Week 36), Visit 8 (Week 48), Visit 9 (Week 50), Follow-up Visit 10 (Week 67) and Follow-up Visit 11 (Week 74)] [**TFL Shell Table 14.3.3.1.1, 14.3.3.2.1, 14.3.3.3.1.1**]. Urinalysis categorical data will be summarized separately [**TFL Shell Table 14.3.3.3.1.2**]. Serology data, Vitamin B12, folate and thyroid function tests are collected at screening only [**TFL Shell Table 14.3.3.4**].

Shift tables will present shift from baseline to each subsequent post-baseline visit, i.e. Baseline, as defined in section 8.2 [**TFL Shell Table 14.3.3.1.2, 14.3.3.2.2, 14.3.3.3.2**].

A box plot will present Hematology and Biochemistry laboratory values per Visit and Treatment Group [**TFL Shell Figure 14.4.1, 14.4.2**].

All laboratory data will be listed [**TFL Shell Listing 16.2.8.1, 16.2.8.2, 16.2.8.3, 16.2.8.4, 16.2.8.5, 16.2.8.6**].

For Haematology, Chemistry. Coagulation, Urinalysis, Immunochemistry. Molecular Biology as per the vendor instructions. if the lab results are reported below the lower limit of quantification (LLOQ), then the value used in summaries could be LLOQ/2, i.e. if the result is reported as "<0.5", then 0.25 will be used. For results reported as above the upper limit of quantification (ULOQ), then the ULOQ could be used, i.e. if the result is ">54.4", then 54.4 will be used. Laboratory data will be listed as per the original result provided by the vendor.

11.5.3 Vital Signs

Vital signs will be measured at each visit. During dosing visits, vital signs (Systolic/Diastolic Blood Pressure (SBP/DBP), Heart Rate, Respiratory Rate and Temperature, Weight) will be measured before and after the administration of study vaccine (except weight which is measured as part of the Physical and Neurological Examination in the eCRF but will be analysed as part of vital signs). At Visit 1 (Week 0), measurements will be made just before the first injection (time 0) and at the following times after the injection at 1, 2, 4, 8, 12, and 24 hours. On subsequent dosing visits [Visit 3 (Week 8), Visit 5 (Week 24) and Visit 8 (Week 48)], measurements will be done at time 0, 1, 2, and 4 hours.

Blood Pressure and Heart Rate will be measured in the sitting and standing positions at the screening visit and at time 0 of Visit 1 (Week 0). The sitting blood pressure should be measured first and should be measured after the subject has been sitting down for at least 5 minutes. The subject should then be asked to stand and the standing blood pressure should be measured after the subject has been standing for 2 minutes. At all other timepoints for Visit 1 (Week 0) or for all other visits, blood pressure and heart rate will be measured in the sitting position only.

Blood pressures will be measured with a standardized mercury manometer; alternative validated methods of measurement may also be used. The point of disappearance of Korotkoff sounds (phase V) will be recorded as the diastolic blood pressure where a sphygmomanometer is used. Heart rate will be determined over 60 seconds following the recording of blood pressure in the corresponding position.

For vital signs parameters the change from baseline value is defined as the value at any given post-baseline timepoint minus the baseline value, i.e. post-baseline value – baseline value, for no dosing visits. For the post-dose vital signs at dosing visits instead of the change from baseline, the change from the last pre-dose assessment will be calculated, i.e. post-baseline value at dosing visit x – last pre-dose value at dosing visit x.

Strictly Confidential. © 2020, [redacted] All rights reserved.

All vital signs parameters [SBP, DBP, Heart Rate, Respiratory Rate and Temperature] actual values and change from baseline, in addition for dosing visits the change from the last pre-dose assessment on the dosing visit, will be summarized by Visit, Timepoint, Position and Treatment Group [**TFL Shell Table 14.3.4.1**].

For example for Visit 3 we will have:
Visit 3 timepoint 1 hour post-dose and change from Visit 3 timepoint 0 hours
Visit 3 timepoint 2 hours post-dose and change from Visit 3 timepoint 0 hours
Visit 3 timepoint 4 hours post-dose and change from Visit 3 timepoint 0 hours

The normal ranges for vital sign parameters are as follows:

Measurement (unit)	Minimum	Maximum
Diastolic Blood Pressure (mmHg)	40	130
Heart Rate (beats per minute)	40	140
Systolic Blood Pressure (mmHg)	80	200
Temperature (˚C)	35	40
Respiratory Rate (breaths/min)	10	25

Shift table categorizing results as low, normal or high based on the normal ranges above will present shift from baseline to each subsequent post-baseline visit, i.e. Baseline, as defined in Section 8.2 [**TFL Shell Table 14.3.4.2**].

A box plot will present vital signs values by Visit and Treatment Group [**TFL Shell Figure 14.5.1**]. In case of multiple timepoints per visit, only the last timepoint in the respective visit will be included.

All vital signs data will be listed for the Intention-to Treat Population [**TFL Shell Listing 16.2.9**].

11.5.4 Electrocardiogram (ECG)

12-lead ECG recording machines will be provided by the central reading company [redacted]. Printouts of each ECG must be kept at the site for archiving. [redacted] will manage ECG data through their Web-based platform. The system allows the acquisition, storage and display of ECG recordings from the ECG machines so that cardiologists can review them. Please refer to the [redacted] ECG manual for further details.

Normal, abnormal not clinically significant and abnormal clinically significant results as interpreted by the site will be summarized by Visit (Baseline, as defined in Section 8.2) and Treatment Group (and Overall) [**TFL Shell Table 14.3.5.1**].

ECG parameters [PR interval (msec), RR interval (msec), QRS duration (msec), QT Interval (msec), QTcB Interval (msec) and QTcF interval (msec)] actual values and change from baseline where baseline and change from baseline are as defined in Section 8.2 and Section 8.3, will be summarized by Visit, and Treatment Group (and overall) [**TFL Shell Table 14.3.5.2**].

Strictly Confidential. © 2020, [redacted] All rights reserved.

The number and percentage of subjects with an absolute QTcB Interval (msec) ≤480 or >480; and an absolute QTcF interval (msec) ≤480 or >480 will be summarized by Visit, and Treatment Group (and overall) [**TFL Shell Table 14.3.5.3**].

The number and percentage of subjects with a change from baseline in QTcB Interval (msec) and QTcF interval (msec) <30 or ≥30 or <60 or ≥60 will be summarized by Visit, and Treatment Group (and overall) [**TFL Shell Table 14.3.5.4**].

Shift table categorizing results as normal, abnormal (clinically significant / not clinically significant) will present shift from baseline to each subsequent post-baseline visit, i.e. Baseline, as defined in Section 8.2 [**TFL Shell Table 14.3.5.5**].

All ECG data will be listed for the Intention-to Treat Population [**TFL Shell Listing 16.2.10.1 and 16.2.10.2**].

11.5.5 Physical Examinations

A physical examination including the assessment of general appearance, the head, eyes, ears, nose, throat, heart, chest, lungs, abdomen, lymph nodes, extremities, peripheral pulses, skin and any other physical conditions of note is to be conducted at each visit [Screening, Visit 1 (Week 0), Visit 2 (Week 2), Visit 3 (Week 8), Visit 4 (Week 10), Visit 5 (Week 24), Visit 6 (Week 26), Visit 7 (Week 36), Visit 8 (Week 48), Visit 9 (Week 50), Follow-up Visit 10 (Week 67) and Follow-up Visit 11 (Week 74)].

A summary of physical examination finding will be presented by treatment group (and overall for each main cohort) and visit for the Intention-to Treat Population [**TFL Shell Table 14.3.6.1**].

A shift table will present shift from baseline to each subsequent post-baseline visit, i.e. Baseline, as defined in section 8.2 [**TFL Shell Table 14.3.6.2**].

Any clinically significant abnormalities identified after the subject signs the ICF and up to the last safety follow-up visit will be recorded on the AE eCRF and summarized as outlined in SAP Section 11.5.1. Any clinically significant abnormality that was pre-existing at the time of signing the ICF will be recorded on the Medical History eCRF and summarized as outlined in SAP Section 11.3.

Physical examination data will be listed for the ITT Population [**TFL Shell Listing 16.2.11**].

11.5.6 Neurological Examinations

Neurological examination data including the examination of the cranial nerves, upper and lower extremities for muscle strength, reflexes, sensation and cerebellar function will be conducted at each visit [Screening, Visit 1 (Week 0), Visit 2 (Week 2), Visit 3 (Week 8), Visit 4 (Week 10), Visit 5 (Week 24), Visit 6 (Week 26), Visit 7 (Week 36), Visit 8 (Week 48), Visit 9 (Week 50), Follow-up Visit 10 (Week 67) and Follow-up Visit 11 (Week 74)] [**TFL Shell Table 14.3.7.1**].

Data will be summarized by treatment group (and overall for each main cohort) and visit for the ITT Population.

A shift table will present shift from baseline to each subsequent post-baseline visit, i.e. Baseline, as defined in section 8.2 [**TFL Shell Table 14.3.7.2**].

All neurological examination data will be listed [**TFL Shell Listing 16.2.11**].

11.5.7 Magnetic Resonance Imaging (MRI)

Brain MRI scans will be conducted according to the schedule of assessments, see **Figure 2** above, and examined for evidence of encephalitis and other brain pathology. Scans will be systematically analyzed e.g. for the presence of micro hemorrhages or vasogenic edema by the Central reading company ([redacted]. Please refer to the [redacted] MRI manual for further details.

Strictly Confidential. © 2020, [redacted] All rights reserved.

Normal, abnormal not clinically significant and abnormal clinically significant results as interpreted by the site will be summarized by Visit (Baseline, as defined in section 8.2) and Treatment Group (and Overall) for the ITT Population [**TFL Shell Table 14.3.8.1**].

MRI results will be summarized by Visit (Baseline, as defined in section 8.2) and Treatment Group (and Overall) for the ITT Population [**TFL Shell Table 14.3.8.2**].

Any clinically significant abnormalities identified after the subject signs the ICF and up to the last safety follow-up visit will be recorded on the AE eCRF and summarized as outlined in section 11.5.1. Any clinically significant abnormality that was pre-existing at the time of signing the ICF will be recorded on the Medical History eCRF and summarized as outlined in section 11.3.

All MRI data will be listed [**TFL Shell Listing 16.2.7.7.1**].

11.5.8 Cognitive/Clinical Variables

The following cognitive/clinical assessments will be conducted for this study:

- CDR-SB (Clinical Dementia Rating Scale-Sum of Boxes) at visits: Screening (applicable to all subjects except those in Sub-cohort 1.1 and those enrolled prior to Protocol v4.0), Visit 1 (Week 0) (only applicable to sub-cohort 1.1 and to patients enrolled prior to protocol v4.0 being locally approved), Visit 6 (Week 26), Visit 9 (Week 50) and Visit 11 (Week 74). The baseline value is the score obtained at Screening except for subjects of sub-cohort 1.1 and to patients enrolled prior to protocol v4.0 being locally approved for which the baseline value is the score obtained at V1 [Week 0]. For other endpoints, the baseline value is the score obtained at V1 [Week 0].
- RBANS (Repeatable Battery for the Assessment of Neuropsychological Status) at visits: Screening, Visit 1 (Week 0), Visit 6 (Week 26), Visit 9 (Week 50) and Visit 11 (Week 74). Three versions of the scale are used during the study: form A (at V1 and V9), form B (at V6 and V11) and form C (at Screening).
- Mini Mental State Examination (MMSE) at Screening only
- NPI (Neuropsychiatric Inventory Scale) at visits: Visit 1 (Week 0), Visit 6 (Week 26), Visit 9 (Week 50) and Visit 11 (Week 74)
- Columbia-Suicide Severity Rating Scale (C-SSRS) at visits: Screening, Visit 1 (Week 0), Visit 6 (Week 26), Visit 9 (Week 50) and Visit 11 (Week 74)
- MacCAT-CR (MacArthur Competence Assessment Tool for Clinical Research, applicable only to subjects from the Netherlands) at visits: Screening, Visit 5 (Week 24) and Visit 8 (Week 48)

11.5.8.1 CDR-SB

The CDR-SB is a global rating of the function of AD subjects assessed in six categories: memory, orientation, judgement and problem solving, community affairs, home and hobbies and personal care. It is based on a semi-structured interview conducted with the subject and caregiver, by a rater without access to the results of the cognitive tests described above. Each category has scores from 0 (no symptoms) to 3 (severe) and the sum of these items (sum of boxes) may therefore range from 0 to 18 points. The CDR global score is derived from scores of each individual category and will be used as part of the selection criteria (score of 0.5 or 1 at Screening).

Actual values and change from baseline [Week 0 or Screening] will be summarized by Visit (Visit 6 [Week 10], Visit 9 [Week 50] and Visit 11 [Week 74]) and Treatment Group (and overall) for the Intention-to Treat Population [**TFL Shell Table 14.3.9**].

The CDR-SB data including CDR global score will also be listed for the ITT Population [**TFL Shell Listing 16.2.7.10**].

Strictly Confidential. © 2020, [redacted] All rights reserved.

Additionally, specific footnote will be included where data on CDR-SB have been collected remotely by telephone interview to specify any details, if needed. Sub-analyses related to the remote mode of administration will not be conducted.

11.5.8.2 RBANS

The RBANS is a test designed to measure cognitive decline or improvement in a short series of 12 subtests that measure attention, language, visuospatial/constructional abilities, and immediate and delayed memory. The test yields 5 Index scores and a Total Scale score. Normative information from the test manual is used to calculate the Index and Total scores.

RBANS Total Scale Score and change from baseline will be summarized by Visit (Visit 6 [week 10], Visit 9 [Week 50] and Visit 11 [Week 74]) and Treatment Group (and overall) for the Intention-to Treat Population [**TFL Shell Table 14.3.10**].

The RBANS data will also be listed for the ITT Population [**TFL Shell Listing 16.2.7.9**].

Additionally, specific footnote will be included where data on RBANS have been collected remotely by telephone interview to specify any details, if needed. Sub-analyses related to the remote mode of administration will not be conducted.

11.5.8.3 MMSE

The MMSE is a widely used test of overall cognitive function, assessing memory, orientation and praxis in a short series of tests. The score is from 0 to 30 with 30 being the best possible and 0 being the worst possible score. The test will only be performed at the screening visit to confirm subject's eligibility to participate.

The MMSE data will also be listed for the ITT Population [**TFL Shell Listing 16.2.7.11**].

11.5.8.4 NPI

The NPI is a 12-item scale which assesses domains/behavioral disturbances commonly occurring in dementia subjects. No score will be calculated if there is missing data in any item scale. Twelve domains/behavioral areas are included in the NPI: delusions, hallucinations, agitation/aggression, depression/dysphoria, anxiety, elation/euphoria, apathy/indifference, disinhibition, irritability/lability, aberrant motor behavior, night-time behaviors, and appetite/eating changes. Through a structured interview with the caregiver, each of these 12 domains will be assessed. For each behavior that has not occurred a frequency of 0 will be recorded. For each behavior that is determined to have occurred, the frequency (1: Occasionally, 2: Often, 3: Frequently, 4: Very Frequently) and severity (1: Mild, 2: Moderate, 3: Marked) will be determined. The frequency and severity will be multiplied together to provide a total score. The total NPI score is calculated by summing across all 12 domains/behaviors generating a score which ranges from 0 (no behaviors present) -144 [all behaviors present with greatest frequency (4: Very Frequently) and greatest severity (3: Marked), i.e. 12x4x3]; a higher score indicates more severe psychopathology. Additionally, the caregiver distress for each domain/behavior will be assessed (0: Not at all, 1: Minimally, 2: Mildly, 3: Moderately, 4: Severely, 5: Very severely or extremely). The NPI Total Distress Score is calculated by adding the individual distress scores for the individual domains/behaviors. The NPI Total Distress Score will range from 0 (0: Not at all recorded for each domain/behavior) to 60 (5: Very severely or extremely recorded for each domain/behavior, i.e. 12x5); a higher score indicates greater caregiver distress.

Strictly Confidential. © 2020, [redacted] All rights reserved.

The NPI Total Score and change from baseline will be summarized by Visit (Visit 6 [Week 10], Visit 9 [Week 50] and Visit 11 [Week 74]) and Treatment Group (and overall) for the Intention-to Treat Population [**TFL Shell Table 14.3.11.1**].

The NPI Total Distress Score and change from baseline will be summarized by Visit (Visit 6 [Week 10], Visit 9 [Week 50] and Visit 11 [Week 74]) and Treatment Group (and overall) for the Intention-to Treat Population [**TFL Shell Table 14.3.11.2**].

The NPI data will be also listed for the Intention-to Treat Population [**TFL Shell Listing 16.2.7.8**].

Additionally, specific footnote will be included where data on NPI have been collected remotely by telephone interview to specify any details, if needed. Sub-analyses related to the remote mode of administration will not be conducted.

11.5.8.5 C-SSRS

Recent guidance from the FDA has indicated the importance of collecting data on potential induction of suicidal behavior with all new agents acting on the central nervous system. Although no such effects are anticipated with the study vaccines investigated, this will be directly evaluated by collecting the C-SSRS, at the visits indicated above, during the study. Questions related to suicidal behavior or ideation are directly asked by the rater to the subject. The C-SSRS has a “Baseline Version” which will be completed at the Screening visit and a “Since Last Visit Version” that will be completed at all other study visits.

The frequency of suicidal ideation and whether or not subjects exhibited suicidal behavior will be summarized by Treatment Group (and overall) [**TFL Shell Table 14.3.12**].

The C-SSRS data will be also listed for the Intention-to Treat Population [**TFL Shell Listing 16.2.7.12**] individual narratives will be presented for all subjects who report serious suicidal intent or any suicidal acts during the study.

11.5.8.6 MacCAT-CR

In the Netherlands, the subject’s decisional capacity will be assessed by using the MacArthur Competence Assessment Tool for Clinical Research (MacCAT-CR). The MacCAT-CR assesses the subject capacities to consent to research participation (Appelbaum and Grisso, 2001). It is a 21-item structured interview with four subscales assessing the following four main dimensions of decision making: understanding, appreciation, reasoning, and expressing a choice. Questions are tailored to the research context defined in this protocol. The determination that the subject is able to give informed consent and is able to maintain his/her decisional capabilities during the study will be based on the subject’s responses to the questions in conjunction with the Investigator’s clinical judgement. The rater must be an investigator or delegate adequately trained.

The MacCAT-CR interview will be performed at study start prior to signing the informed consent form and subsequently at V5 and V8. In case it is determined that the subject is no longer able to maintain his/her decisional capacity, participation should be discontinued.

For the MacCAT-CR assessment, the baseline value is the score obtained at Screening. The proportion of subjects in the Netherlands (please refer to Protocol V7.0 / 22-Oct-2021) who are maintaining their decision-making capacity at Visit 5 [Week 24] and Visit 8 [Week 48] using the MacCAT-CR assessment will also be presented per dose group [**TFL Shell Table 14.3.13**]. The details will be listed [**TFL Shell Listing 16.1.7**].

Strictly Confidential. © 2020, [redacted] All rights reserved.

11.6 Immune Response Analyses

At all visits, blood will be collected for determination of the immune response in serum, i.e. anti-pTau and anti-Tau IgG and IgM, anti-ePHF IgG, anti-T50 IgG, anti-CRM IgG and anti-CpG IgG titers.

11.6.1 Immune Response for Primary Endpoints

Descriptive statistics of actual values and changes from baseline as outlined in section 11.0, with the addition of Geometric Mean and 95% normal approximation confidence intervals (CI) as outlined in sections 8.15 and 8.17, will be presented for the primary immune response variable, anti-pTau IgG and anti-ePHF IgG titers in serum by Visit [Baseline, Visit 2 (Week 2), Visit 3 (Week 8), Visit 4 (Week 10), Visit 4.1 (Week 15), Visit 4.2 (Week 20), Visit 5 (Week 24), Visit 6 (Week 26), Visit 6.1 (Week 31), Visit 7 (Week 36), Visit 7.1 (Week 42), Visit 8 (Week 48), Visit 9 (Week 50), Follow-up Visit 10 (Week 67) and Follow-up Visit 11 (Week 74)] and Treatment Group [**TFL Shell Table 14.2.1.1, 14.2.1.1.1; 14.2.5.1, 14.2.5.1.1**]. This variable will further be summarized by Treatment Group for responder rate [**TFL Shell Table 14.2.1.2, 14.2.1.2.1; 14.2.5.2, 14.2.5.2.1**], and area under the curve and peak [**TFL Shell Table 14.2.1.3, 14.2.1.3.1; 14.2.5.3, 14.2.5.3.1**] by Treatment Group (and overall for baseline data only) in the Intention-to-Treat Population.

The time-dependent development of the geometric mean titers will be displayed graphically per Treatment Group for the actual values as well as for change from baseline [**TFL Shell Figure 14.1.1.1, 14.1.1.1.1, 14.1.1.1.2; 14.1.5.1, 14.1.5.1.1, 14.1.5.1.2]** and by subject [**TFL Shell Figure 14.1.1.2, 14.1.5.2**]. Boxplot of the actual value summarized by Visit and Treatment Group will also be prepared [**TFL Shell Figure 14.1.1.4 and 14.1.5.4**].

Response rates by Visit will be displayed graphically in a response rate verses dose plot to investigate whether there is any dose response relationship [**TFL Shell Figure 14.1.1.3; 14.1.5.3**].

No statistical testing has been planned for antibody responses.

11.6.2 Immune Response for Secondary Endpoints

Anti-Tau IgG, anti-pTau IgM and anti-Tau IgM titers, in serum, will be presented in the same way as anti-pTau IgG, outlined in section 11.6.1 above [**TFL Shell Table 14.2.2.1, 14.2.2.1.1, 14.2.2.2, 14.2.2.2.1, 14.2.2.3, 14.2.2.3.1; 14.2.3.1, 14.2.3.1.1, 14.2.3.2, 14.2.3.2.1, 14.2.3.3, 14.2.3.3.1; 14.2.4.1, 14.2.4.1.1, 14.2.4.2, 14.2.4.2.1, 14.2.4.3, 14.2.4.3.1**].

The anti-ePHF avidity index will be summarized by visit only [**TFL Shell Table 14.2.6.1, 14.2.6.1.1**].

The time-dependent development of the geometric mean titers (Anti-Tau IgG, Anti-pTau IgM, Anti-Tau IgM) and anti-ePHF avidity index will be displayed graphically per Treatment Group for the actual values as well as for change from baseline [**TFL Shell Figure 14.1.2.1, 14.1.2.1.1, 14.1.2.1.2; 14.1.3.1, 14.1.3.1.1, 14.1.3.1.2; 14.1.4.1, 14.1.4.1.1, 14.1.4.1.2; 14.1.6.1, 14.1.6.1.1, 14.1.6.1.2]** and by subject [**TFL Shell Figure 14.1.2.2; 14.1.3.2; 14.1.4.2; 14.1.6.2**]. Boxplot of the actual value summarized by Visit and Treatment Group will also be prepared [**TFL Shell Figure 14.1.2.4; 14.1.3.4; 14.1.4.4**] (not required for anti-ePHF avidity index).

Response rates by visit will be displayed graphically in a response rate verses dose plot to investigate whether there is any dose response relationship [**TFL Shell Figure 14.1.2.3; 14.1.3.3; 14.1.4.3**]. Response rates by visit are not applicable for the avidity index.

Key antibody ratios Anti-ePHF IgG / Anti-pTau IgG, Anti-ePHF IgG / Anti-Tau IgG and Anti-pTau IgG / Anti-Tau IgG will also be summarized by Treatment Group and Visit and presented graphically including Geometric Mean (±95% CI) over time by Treatment Group and subjects profile of the change from baseline of the log10 values [**TFL Shell Table 14.2.9.4, 14.2.9.4.1, TFL Shell Figure 14.1.10.1, 14.1.10.2**].

Strictly Confidential. © 2020, [redacted] All rights reserved.

The Immunogenicity data will be also listed for the ITT Population [**TFL Shell Listing 16.2.12.1 16.2.12.2, 16.2.12.3 and 16.2.12.4**].

11.6.3 Immune Response for Exploratory Endpoints

Anti-T50, anti-CpG and anti-CRM IgG titers in serum will be presented in the same way as outlined in section 11.6.1 above [**TFL Shell Table 14.2.7.1, 14.2.7.1.1, 14.2.7.2, 14.2.7.2.1, 14.2.7.3, 14.2.7.3.1; 14.2.8.1, 14.2.8.1.1, 14.2.8.2, 14.2.8.2.1, 14.2.8.3, 14.2.8.3.1; 14.2.9.1, 14.2.9.1.1, 14.2.9.2, 14.2.9.2.1, 14.2.9.3, 14.2.9.3.1**]

The time-dependent development of the geometric mean titers (Anti-T50, anti-CRM and anti-CpG) will be displayed graphically per Treatment Group for the actual values as well as for change from baseline [**TFL Shell Figure 14.1.7.1, 14.1.7.1.1, 14.1.7.1.2; 14.1.8.1, 14.1.8.1.1, 14.1.8.1.2; 14.1.9.1, 14.1.9.1.1, 14.1.9.1.2**] and by subject [**TFL Shell Figure 14.1.7.2; 14.1.8.2; 14.1.9.2**].

Response rates by visit will be displayed graphically in a response rate verses dose plot to investigate whether there is any dose response relationship [**TFL Shell Figure 14.1.7.3; 14.1.8.3; 14.1.9.3**].

11.7 Other Exploratory Analyses

11.7.1 AD biomarkers in blood and CSF

The following putative AD biomarkers will be assessed in plasma:

- Brain-derived Tau (BD-Tau)
- pTau217+
- pTau181
- Aβ40
- Aβ42
- Aβ42/Aβ40 ratio
- NFL
- GFAP
- YKL-40

and in Cerebrospinal Fluid (CSF):

- pTau217+
- pTau181
- Total Tau
- Aβ40
- Aβ42
- Aβ42/Aβ40 ratio
- NFL
- GFAP
- Neurogranin
- YKL-40
- In a mass spectrometry assay not validated: pTauS396, pTauS199, pTauS202, pTauT205, pTauT231, pTauT217, pTauT181. All these biomarkers should be considered as purely exploratory, included pTau181 and pTau217 which have been tested with a validated assay (see above).

Strictly Confidential. © 2020, [redacted] All rights reserved.

Blood samples for AD biomarker testing will be collected at all visits except screening [Visit 1 (Week 0), Visit 2 (Week 2), Visit 3 (Week 8), Visit 4 (Week 10), Visit 4.1 (Week 15), Visit 4.2 (Week 20), Visit 5 (Week 24), Visit 6 (Week 26), Visit 6.1 (Week 31), Visit 7 (Week 36), Visit 7.1 (Week 42), Visit 8 (Week 48), Visit 9 (Week 50), Follow-up Visit 10 (Week 67) and Follow-up Visit 11 (Week 74)].

CSF samples for AD biomarker testing will be collected at Screening, Visit 6 (Week 26) and Visit 9 (Week 50).

All AD biomarkers data, change from baseline, percent (%) change from baseline and fold-change from baseline will be summarized by Visit and Treatment Group for each source (plasma and CSF) [**TFL Shell Table 14.2.10.1.1, 14.2.10.1.1.1, 14.2.10.2, 14.2.10.2.1**]. AD Biomarkers (in CSF) for eligibility are summarized separately [**TFL Shell Table 14.2.10.1.2.2, 14.2.10.2.3**].

Each biomarker will be presented graphically in a time series plot, presenting mean values per Treatment Group, and in a box plot per Visit and Treatment Group for each source (plasma and CSF) [**TFL Shell Figure 14.2.1, 14.2.2, 14.2.9, 14.2.10**]. Individual data by subject will be displayed graphically by treatment group [**TFL Shell Figure 14.2.3, 14.2.4**]. Mean time course of change from baseline and individual change from baseline will also be provided [**TFL Shell Figure 14.2.5, 14.2.6, 14.2.7, 14.2.8**]. Box plots of actual log10-transformed values, fold-change from baseline and change from baseline of the log10-transformed values will be shown [**TFL Shell Figure 14.2.11, 14.2.12, 14.2.13, 14.2.14, 14.2.15, 14.2.16**].

The AD biomarker data in blood and CSF will be also listed for the ITT Population [**TFL Shell Listing 16.2.13**].

11.7.2 T-cell activation (ELISPOT)

T-cell activation (as measured by the ELISPOT assay) will not be tested for this study since there is no safety concern. No outputs on T-cell activation will be prepared.

11.7.3 Inflammatory cytokines in blood

Inflammatory cytokines (e.g. IL-1β, IL-2, IL-6; IL-8, IL-10, IFN-γ and TNF-α) will not be tested for this study since there is no safety concern. No outputs on inflammatory cytokines in blood will be prepared.

11.7.4 Volumetric MRI

Volumetric MRI analyses will also be performed by [redacted] The volume of different brain regions (i.e. whole brain, intracranial, hippocampus and ventricles) will be measured at screening (baseline) using an automated FreeSurfer-based segmentation method. An automated Tensor-Based Morphometry (TBM) method will then be used to measure the change of volume at follow-up visits for each of the following structures:

- whole brain (bilateral)
- hippocampus (bilateral, left and right)
- ventricles (bilateral)

For each brain structure, volume change at follow-up visits [Visit 4 (Week 10), Visit 6 (Week 26), Visit 9 (Week 50) and Visit 11 (Week 74)] will be presented graphically in a time series plot, presenting mean values (CI 95%) per treatment group [**TFL Shell Figure 14.5.2**] and individual values [**TFL Shell Figure 14.5.3**]. Box plot of MRI volume by brain region, visit and treatment group will also be presented [**TFL Shell Figure 14.5.4**]. Baseline data and volume change at each follow-up visit data will be summarized

Strictly Confidential. © 2020, [redacted] All rights reserved.

per treatment group [**TFL Shell Table 14.3.8.3**] for each structure. Geometric mean, standard deviation, median, 25th quantile, 75th quantile, minimum and maximum will be reported. Individual baseline values and volume change at each follow-up visit will also be presented [**TFL Shell Listing 16.2.7.7.2**].

12.0 Changes from the Analysis Planned in the Protocol

Due to the COVID-19 pandemic a notification of an Urgent Safety Measure (USM) for a change to protocol study procedures, effective immediately, was submitted to FIMEA on **01-Apr-2020** and Finnish EC (TUKIJA) on **03-Apr-2020**. A protocol amendment following the USM including the below changes was submitted to FIMEA on **17-Apr-2020** and TUKIJA on **24-Apr-2020**:

- Replacement of Visit 5 (Week 24) and Visit 6 (Week 26) by remote visits in 7 of 8 Sub-cohort 1.1 subjects. As a consequence, the third immunization, as well as procedures and assessments requiring the subjects to travel at Visit 5 (Week 24) and Visit 6 (Week 26) were cancelled.
- Cancellation of the third phone call (P3), usually performed 48-72 hours after the third immunization. This change is impacting 7 of 8 subjects who have not had their immunization at Visit 5 (Week 24) prior to the at-risk period.

Due to the changes presented above IA #2 Sub-cohort 1.1 at Week 50 will be executed as follows:

- Individual data (listings and individual figures) will not be presented for IA #2 Sub-cohort 1.1 Week 50.
- Aggregate data (summary tables and figures) will be provided for all timepoints (up to Week 50) except the timepoints where the visits were missed by 7 of 8 subjects in Sub-cohort 1.1 for which we will have only 1 value (i.e. Visit 5 Week 24 and Visit 6 Week 26).

Footnotes will be included to all outputs affected by the selection criteria as given below:

- Aggregate data outputs: “Note: Aggregate data is provided for all visits except those missed by 7 out of 8 subjects in Sub-cohort 1.1 (Visits 5 and 6).”

In certain cases, where subjects missed any visit or dropped out from the study, IA will be executed as follows:

- If there is a risk of unblinding individual data (listings and individual figures) will be presented only up until the missed visit or data for those subjects will not be included.
- Aggregate data (summary tables and figures) will be provided for all timepoints

13.0 Validation

[redacted] goal is to ensure that each TFL delivery is submitted to the highest level of quality. [redacted] quality control procedures will be documented separately in the study specific quality control plan.

Strictly Confidential. © 2020, [redacted] All rights reserved.

Appendix 1 Glossary of Abbreviations

**AD**	Alzheimer's Disease
**AE**	Adverse Event
**ANA**	Anti-nuclear Antibodies
**Anti-dsDNA**	Anti-double stranded DNA Antibodies
**ApoE**	Apolipoprotein E
**aPTT**	activated Partial Thromboplastin Time
**AUC**	Area Under the Curve
**BLQ**	Below the Limit of Quantification
**CDR-SB**	Clinical Dementia Rating scale Sum of Boxes
**CI**	Confidence Interval
**cm (unit)**	Centimeter
**CRF**	Case Report Form
**CRP**	C-Reactive Protein
**CSF**	Cerebrospinal Fluid
**C-SSRS**	Columbia-Suicide Severity Rating Scale
**DLP**	Data Lock Point
**DSMB**	Data and Safety Monitoring Board
**ECG**	Electrocardiogram
**EDC**	Electronic Data Capture
**ELISPOT**	Enzyme-Linked Immunospot
**ePHF**	Enriched Paired Helical Filament
**Gamma-GT**	Gamma-glutamyl Transferase
**IA**	Interim Analysis(ses)
**ID**	Identifier / Identification Number
**IFN-γ**	Interferon-gamma
**IgG**	Immunoglobulin G
**IL-4**	Interleukin-4
**IMP**	Investigational Medicinal Product
**INR**	International Normalized Ratio
**IRT**	Interactive Response Technology
**ITT**	Intention To Treat
**kg (unit)**	Kilogram
**LLOQ**	Lower Limit of Quantification

Strictly Confidential. © 2020, [redacted] All rights reserved.

**μg (unit)**	Microgram
**MacCAT-CR**	MacArthur Competence Assessment Tool for Clinical Research
**MCI**	Mild Cognitive Impairment
**MMSE**	Mini Mental State Examination
**MRI**	Magnetic Resonance Imaging
**NPI**	Neuropsychiatric Inventory Scale
**PBS**	Phosphate Buffered Saline
**PP**	Per Protocol
**PSO**	Predictivv Study Operation
**pTau**	Phospho-Tau
**RBANS**	Repeatable Battery for the Assessment of Neuropsychological Status
**SAE**	Serious Adverse Event
**SAP**	Statistical Analysis Plan
**SD**	Standard Deviation
**SDRC**	Sponsor Data Review Committee
**SUSAR**	Suspected Unexpected Serious Adverse Reaction
**TEAE**	Treatment Emergent Adverse Event
**USM**	Urgent Safety Measure
**eTMF**	electronic Trial Master File
**TBM**	Tensor-Based Morphometry
**TNF-α**	Tumor Necrosis Factor alpha

Strictly Confidential. © 2020, [redacted] All rights reserved.

Appendix 2 Tables, Figures, Listings

The TFL shells and table of contents for this study are provided in a separate document titled “ACI-35-1802 Main TFLs”. Please see below. Additionally, the tables below summarize the DSMB SAP Safety TFLs and main SAP TFLs required for each Interim Analysis:

MAIN Statistical Analysis Plan	Cohort 1 Interim Analysis (IA1)	Cohort 1 IA2 to IA7 Cohort 2 IA1 to IA6	Final Analysis
Table 14.1.1.1 Subject Analysis Sets (Screened Subjects)			X
Table 14.1.1.2 Subject Disposition (Intention-to-Treat Population)			X
Table 14.1.1.3.1 Important Protocol Deviations (Intention-to-Treat Population)			X
Table 14.1.1.3.2 COVID-19 Related Protocol Deviations (Intention-to-Treat Population)			X
Table 14.1.1.3.3 Summary of Subjects Impacted by COVID-19 (Intention-to-Treat Population)			X
Table 14.1.1.4 Subjects in Study by Visit (Intention-to-Treat Population)			X
Table 14.1.2 Demographic and Baseline Characteristics (Intention-to-Treat Population)			X
Table 14.1.3.1 Past Medical History by System Organ Class and Preferred Term (Intention-to-Treat Population)			X
Table 14.1.3.2 Current Medical History by System Organ Class and Preferred Term (Intention-to-Treat Population)			X
Table 14.1.4 Prior Medications by ATC Classification and WHO Drug Dictionary Preferred Term (Safety Population)			X
Table 14.1.5 Concomitant Medications by ATC Classification and WHO Drug Dictionary Preferred Term (Safety Population)			X
Table 14.1.6 Treatment Compliance (Intention-to-Treat Population)			X
Table 14.2.1.1 Anti-pTau IgG (AU/mL) titers, in serum, and Change from Baseline (Intention-to-Treat Population) *	X	X	X
Table 14.2.1.1.1 Anti-pTau IgG (AU/mL) titers, in Serum, and Change from Baseline (Per Protocol Population)			X
Table 14.2.1.2 Anti-pTau IgG (AU/mL) titers, in serum – Responder Analysis (Intention-to-Treat Population) *	X	X	X
Table 14.2.1.2.1 Anti-pTau IgG (AU/mL) titers, in Serum – Responder Analysis (Per Protocol Population)			X
Table 14.2.1.3 Anti-pTau IgG (AU/mL) titers, in serum, Peak and Area Under the Curve (Intention-to-Treat Population) *	X	X	X
Table 14.2.1.3.1 Anti-pTau IgG (AU/mL) titers, in Serum, Peak and Area Under the Curve (Per Protocol Population)			X
Table 14.2.2.1 Anti-Tau IgG (AU/mL) titers, in serum, and Change from Baseline (Intention-to-Treat Population) *		X	X
Table 14.2.2.1.1 Anti-Tau IgG (AU/mL) titers, in Serum, and Change from Baseline (Per Protocol Population)			X
Table 14.2.2.2 Anti-Tau IgG (AU/mL) titers, in serum – Responder Analysis (Intention-to-Treat Population) *		X	X

Strictly Confidential. © 2020, [redacted] All rights reserved.

MAIN Statistical Analysis Plan	Cohort 1 Interim Analysis (IA1)	Cohort 1 IA2 to IA7 Cohort 2 IA1 to IA6	Final Analysis
Table 14.2.2.2.1 Anti-Tau IgG (AU/mL) titers, in Serum – Responder Analysis (Per Protocol Population)			X
Table 14.2.2.3 Anti-Tau IgG (AU/mL) titers, in serum, Peak and Area Under the Curve (Intention-to-Treat Population) *		X	X
Table 14.2.2.3.1 Anti-Tau IgG (AU/mL) titers, in Serum, Peak and Area Under the Curve (Per Protocol Population)			X
Table 14.2.3.1 Anti-pTau IgM (AU/mL) titers, in serum, and Change from Baseline (Intention-to-Treat Population) *	X	X	X
Table 14.2.3.1.1 Anti-pTau IgM (AU/mL) titers, in Serum, and Change from Baseline (Per Protocol Population)			X
Table 14.2.3.2 Anti-pTau IgM (AU/mL) titers, in serum – Responder Analysis (Intention-to-Treat Population) *	X	X	X
Table 14.2.3.2.1 Anti-pTau IgM (AU/mL) titers, in Serum – Responder Analysis (Per Protocol Population)			X
Table 14.2.3.3 Anti-pTau IgM titers, in serum, Peak and Area Under the Curve (Intention-to-Treat Population) *	X	X	X
Table 14.2.3.3.1 Anti-pTau IgM (AU/mL) titers, in Serum, Peak and Area Under the Curve (Per Protocol Population)			X
Table 14.2.4.1 Anti-Tau IgM (AU/mL) titers, in serum, and Change from Baseline (Intention-to-Treat Population) *		X	X
Table 14.2.4.1.1 Anti-Tau IgM (AU/mL) titers, in Serum, and Change from Baseline (Per Protocol Population)			X
Table 14.2.4.2 Anti-Tau IgM (AU/mL) titers, in serum – Responder Analysis (Intention-to-Treat Population) *		X	X
Table 14.2.4.2.1 Anti-Tau IgM (AU/mL) titers, in Serum – Responder Analysis (Per Protocol Population)			
Table 14.2.4.3 Anti-Tau IgM (AU/mL) titers, in serum, Peak and Area Under the Curve (Intention-to-Treat Population) *		X	X
Table 14.2.4.3.1 Anti-Tau IgM (AU/mL) titers, in Serum, Peak and Area Under the Curve (Per Protocol Population)			X
Table 14.2.5.1 Anti-ePHF IgG AU/mL) titers, in serum, and Change from Baseline (Intention-to-Treat Population) *	Optional	X	X
Table 14.2.5.1.1 Anti-ePHF IgG (AU/mL) titers, in Serum, and Change from Baseline (Per Protocol Population)			X
Table 14.2.5.2 Anti-ePHF IgG (AU/mL) titers, in serum – Responder Analysis (Intention-to-Treat Population) *	Optional	X	X
Table 14.2.5.2.1 Anti-ePHF IgG (AU/mL) titers, in Serum – Responder Analysis (Per Protocol Population)			X
Table 14.2.5.3 Anti-ePHF IgG (AU/mL) titers, in serum, Peak and Area Under the Curve (Intention-to-Treat Population) *	Optional	X	X
Table 14.2.5.3.1 Anti-ePHF IgG (AU/mL) titers, in Serum, Peak and Area Under the Curve (Per Protocol Population)			X
Table 14.2.6.1 Summary of Avidity Index on ePHF, in serum (Intention-to-Treat Population) *		X	X
Table 14.2.6.1.1 Summary of Anti-ePHF Avidity Index, in Serum (Per Protocol Population)			X

Strictly Confidential. © 2020, [redacted] All rights reserved.

MAIN Statistical Analysis Plan	Cohort 1 Interim Analysis (IA1)	Cohort 1 IA2 to IA7 Cohort 2 IA1 to IA6	Final Analysis
Table 14.2.7.1 Anti-T50 IgG (AU/mL) titers, in serum, and Change from Baseline (Intention-to-Treat Population) *		X[1]	X
Table 14.2.7.1.1 Anti-T50 IgG (AU/mL) titers, in Serum, and Change from Baseline (Per Protocol Population)			X
Table 14.2.7.2 Anti-T50 IgG (AU/mL) titers, in serum – Responder Analysis (Intention-to-Treat Population) *		X[1]	X
Table 14.2.7.2.1 Anti-T50 IgG (AU/mL) titers, in Serum – Responder Analysis (Per Protocol Population)			X
Table 14.2.7.3 Anti-T50 IgG (AU/mL) titers, in serum, Peak and Area Under the Curve (Intention-to-Treat Population) *		X[1]	X
Table 14.2.7.3.1 Anti-T50 IgG (AU/mL) titers, in Serum, Peak and Area Under the Curve (Per Protocol Population)			X
Table 14.2.8.1 Anti-CpG IgG (AU/mL) titers, in serum, and Change from Baseline (Intention-to-Treat Population) *	Optional	X	X
Table 14.2.8.1.1 Anti-CpG IgG (AU/mL) titers, in Serum, and Change from Baseline (Per Protocol Population)			X
Table 14.2.8.2 Anti-CpG IgG (AU/mL) titers, in serum – Responder Analysis (Intention-to-Treat Population) *	Optional	X	X
Table 14.2.8.2.1 Anti-CpG IgG (AU/mL) titers, in Serum – Responder Analysis (Per Protocol Population)			X
Table 14.2.8.3 Anti-CpG IgG (AU/mL) titers, in serum, Peak and Area Under the Curve (Intention-to-Treat Population) *	Optional	X	X
Table 14.2.8.3.1 Anti-CpG IgG (AU/mL) titers, in Serum, Peak and Area Under the Curve (Per Protocol Population)			X
Table 14.2.9.1 Anti-CRM IgG (AU/mL) titers, in serum, and Change from Baseline (Intention-to-Treat Population) *		X[2]	X
Table 14.2.9.1.1 Anti-CRM IgG (AU/mL) titers, in Serum, and Change from Baseline (Per Protocol Population)			X
Table 14.2.9.2 Anti-CRM IgG (AU/mL) titers, in serum – Responder Analysis (Intention-to-Treat Population) *		X[2]	X
Table 14.2.9.2.1 Anti-CRM IgG (AU/mL) titers, in Serum – Responder Analysis (Per Protocol Population)			X
Table 14.2.9.3 Anti-CRM IgG (AU/mL) titers, in serum, Peak and Area Under the Curve (Intention-to-Treat Population) *		X[2]	X
Table 14.2.9.3.1 Anti-CRM IgG (AU/mL) titers, in Serum, Peak and Area Under the Curve (Per Protocol Population)			X
Table 14.2.9.4 Key Antibody Ratios, in Serum, and Change from Baseline (Intention-to-Treat Population)			X
Table 14.2.9.4.1 Key Antibody Ratios, in Serum, and Change from Baseline (Per Protocol Population)			X
Table 14.2.10.1.1 Summary of Actual Value and Change from Baseline in AD Biomarkers (in Plasma) by Time Point (Intention-to-Treat Population)			X

[1] Not applicable to Cohort 2 subjects.

[2] Not applicable to Cohort 1 subjects.

Strictly Confidential. © 2020, [redacted] All rights reserved.

MAIN Statistical Analysis Plan	Cohort 1 Interim Analysis (IA1)	Cohort 1 IA2 to IA7 Cohort 2 IA1 to IA6	Final Analysis
Table 14.2.10.1.1.1 Summary of Actual Value and Change from Baseline in AD Biomarkers (in Plasma) by Time Point (Per Protocol Population)			X
Table 14.2.10.1.2 Summary of Actual Value and Change from Baseline in AD Biomarkers (in CSF) by Time Point (Intention-to-Treat Population)			X
Table 14.2.10.1.2.1 Summary of Actual Value and Change from Baseline in AD Biomarkers (in CSF) by Time Point (Per Protocol Population)			X
Table 14.2.10.1.2.2 Summary of Actual Value of AD Biomarkers for Eligibility (in CSF) by Time Point (Intention-to-Treat Population)			X
Table 14.2.10.1.2.3 Summary of Actual Value of AD Biomarkers for Eligibility (in CSF) by Time Point (Per Protocol Population)			X
Table 14.3.2.1 Overall Summary of Adverse Events and Treatment-Emergent Adverse Events (Safety Population)			X
Table 14.3.2.2 Treatment-Emergent Adverse Events by System Organ Class and Preferred Term (Safety Population)			X
Table 14.3.2.3 Serious Treatment-Emergent Adverse Events by System Organ Class and Preferred Term (Safety Population)			X
Table 14.3.2.4 Severe Treatment-Emergent Adverse Events by System Organ Class and Preferred Term (Safety Population)			X
Table 14.3.2.5 Adverse Drug Reaction Events by System Organ Class and Preferred Term (Safety Population)			X
Table 14.3.2.6 Treatment-Emergent Adverse Events Leading to Discontinuation of Study Treatment by System Organ Class and Preferred Term (Safety Population)			X
Table 14.3.2.7 Treatment-Emergent Adverse Events Leading to Discontinuation from the Study by System Organ Class and Preferred Term (Safety Population)			X
Table 14.3.2.8 Treatment-Emergent Adverse Events with Outcome of Death by System Organ Class and Preferred Term (Safety Population)			X
Table 14.3.2.9 Treatment-Emergent Adverse Events by System Organ Class, Preferred Term, and Maximum Severity (Safety Population)			X
Table 14.3.2.10 Treatment-Emergent Adverse Events by System Organ Class, Preferred Term, and Relationship to Study Drug (Safety Population)			X
Table 14.3.2.11 Most Frequent Treatment-Emergent Adverse Events by Preferred Term (Safety Population)			X
Table 14.3.3.1.1 Hematology Results and Change from Baseline by Visit (Intention-to-Treat Population)			X
Table 14.3.3.1.2 Shift from Baseline Classification of Hematology Results by Visit (Intention-to-Treat Population)			X
Table 14.3.3.2.1 Biochemistry Results and Change from Baseline by Visit (Intention-to-Treat Population)			X
Table 14.3.3.2.2 Shift from Baseline Classification of Biochemistry Results by Visit (Intention-to-Treat Population)			X
Table 14.3.3.3.1 Urinalysis Results and Change from Baseline by Visit – Continuous Data (Intention-to-Treat Population)			X
Table 14.3.3.3.1.2 Urinalysis Results by Visit – Categorical Data (Intention-to-Treat Population)			X
Table 14.3.3.3.2 Shift from Baseline Classification of Urinalysis Results by Visit (Intention-to-Treat Population)			X

Strictly Confidential. © 2020, [redacted] All rights reserved.

MAIN Statistical Analysis Plan	Cohort 1 Interim Analysis (IA1)	Cohort 1 IA2 to IA7 Cohort 2 IA1 to IA6	Final Analysis
Table 14.3.3.4 Summary of Additional Parameters Measured at Screening (Intention-to-Treat Population)			X
Table 14.3.4.1 Vital Signs Results and Change from Baseline by Visit, Timepoint and Position (Intention-to-Treat Population)			X
Table 14.3.4.2 Shift from Baseline Classification of Vital Signs Results by Visit, Timepoint and Position (Intention-to-Treat Population)			X
Table 14.3.5.1 Centrally Read Electrocardiogram (ECG) Interpretation by Visit (Intention-to-Treat Population)			X
Table 14.3.5.2 Centrally Read Electrocardiogram (ECG) Results and Change from Baseline by Visit (Intention-to-Treat Population)			X
Table 14.3.5.3 Centrally Read Electrocardiogram (ECG) Categorical Analysis of QTc by Visit (Intention-to-Treat Population)			X
Table 14.3.5.4 Centrally Read Electrocardiogram (ECG) Categorical Analysis of QTc Change from Baseline by Visit (Intention-to-Treat Population)			X
Table 14.3.5.5 Shift from Baseline Centrally Read Electrocardiogram (ECG) Interpretation by Visit (Intention-to-Treat Population)			X
Table 14.3.6.1 Physical Examination by Visit (Intention-to-Treat Population)			X
Table 14.3.6.2 Shift from Baseline Classification of Physical Examination Results by Visit (Intention-to-Treat Population)			X
Table 14.3.7.1 Neurological Examination by Visit (Intention-to-Treat Population)			X
Table 14.3.7.2 Shift from Baseline Classification of Neurological Examination Results by Visit (Intention-to-Treat Population)			X
Table 14.3.8.1 Magnetic Resonance Imaging (MRI) by Visit (Intention-to-Treat Population)			X
Table 14.3.8.2 Magnetic Resonance Imaging (MRI) Results by Visit (Intention-to-Treat Population)			X
Table 14.3.8.3 Volumetric MRI Analyses by Brain Region (Whole Brain, Intracranial, Hippocampus and Ventricles) (Intention-to-Treat Population)			X
Table 14.3.9 Clinical Dementia Rating Scale-Sum of Boxes (CDR-SB) and Change from Baseline by Visit (Intention-to-Treat Population)			X
Table 14.3.10 Repeatable Battery for the Assessment of Neuropsychological Status (RBANS) Total Scale Score and Change from Baseline by Visit (Intention-to-Treat Population)			X
Table 14.3.11.1 Neuropsychiatric Inventory (NPI) Total Score and Change from Baseline by Visit (Intention-to-Treat Population)			X
Table 14.3.11.2 Neuropsychiatric Inventory (NPI) Total Distress Score and Change from Baseline by Visit (Intention-to-Treat Population)			X
Table 14.3.12 Columbia-Suicide Severity Rating Scale (C-SSRS) by Visit (Intention-to-Treat Population)			X
Table 14.3.13 Subject Capacity to Consent: MacCAT-CR by Visit (Intention-to-Treat Population)			X
Figure 14.1.1.1 Geometric Mean Time Course of Anti-pTau IgG Titer by Treatment Group (Intention-to-Treat Population)	X	X	X
Figure 14.1.1.1.1 Geometric Mean Time Course of Anti-pTau IgG Titer by Treatment Group (Y-axis adjusted) (Intention-to-Treat Population)	X		X
Figure 14.1.1.1.2 Geometric Mean Time Course of Change from Baseline of Anti-pTau IgG Titer by Treatment Group (Intention-to-Treat Population)			X

Strictly Confidential. © 2020, [redacted] All rights reserved.

MAIN Statistical Analysis Plan	Cohort 1 Interim Analysis (IA1)	Cohort 1 IA2 to IA7 Cohort 2 IA1 to IA6	Final Analysis
Figure 14.1.1.2 Individual Time Course of Anti-pTau IgG Titer (Intention-to-Treat Population)	X	X	X
Figure 14.1.1.3 Plot of Anti-pTau IgG Titer Response Rates by Visit and Treatment Group (Intention-to-Treat Population)	X	X	X
Figure 14.1.1.4 Box Plot of the Actual Values of the Anti-pTau IgG Titer by Visit and Treatment Group (Intention-to-Treat Population)			X
Figure 14.1.2.1 Geometric Mean Time Course of Anti-Tau IgG Titer by Treatment Group (Intention-to-Treat Population)		X	X
Figure 14.1.2.1.1 Geometric Mean Time Course of Anti-Tau IgG Titer by Treatment Group (Y-axis adjusted) (Intention-to-Treat Population)			X
Figure 14.1.2.1.2 Geometric Mean Time Course of Change from Baseline of Anti-Tau IgG Titer by Treatment Group (Intention-to-Treat Population)			X
Figure 14.1.2.2 Individual Time Course of Anti-Tau IgG Titer (Intention-to-Treat Population)		X	X
Figure 14.1.2.3 Plot of Anti-Tau IgG Titer Response Rates by Visit and Treatment Group (Intention-to-Treat Population)		X	X
Figure 14.1.2.4 Box Plot of the Actual Values of the Anti-Tau IgG Titer by Visit and Treatment Group (Intention-to-Treat Population)			X
Figure 14.1.3.1 Geometric Mean Time Course of Anti-pTau IgM Titer by Treatment Group (Intention-to-Treat Population)	X	X	X
Figure 14.1.3.1.1 Geometric Mean Time Course of Anti-pTau IgM Titer by Treatment Group (Y-axis adjusted) (Intention-to-Treat Population)			X
Figure 14.1.3.1.2 Geometric Mean Time Course of Change from Baseline of Anti-pTau IgM Titer by Treatment Group (Intention-to-Treat Population)			X
Figure 14.1.3.2 Individual Time Course of Anti-pTau IgM Titer (Intention-to-Treat Population)	X	X	X
Figure 14.1.3.3 Plot of Anti-pTau IgM Titer Response Rates by Visit and Treatment Group (Intention-to-Treat Population)	X	X	X
Figure 14.1.3.4 Box Plot of the Actual Values of the Anti-pTau IgM Titer by Visit and Treatment Group (Intention-to-Treat Population)			X
Figure 14.1.4.1 Geometric Mean Time Course of Anti-Tau IgM Titer by Treatment Group (Intention-to-Treat Population)		X	X
Figure 14.1.4.1.1 Geometric Mean Time Course of Anti-Tau IgM Titer by Treatment Group (Y-axis adjusted) (Intention-to-Treat Population)			X
Figure 14.1.4.1.2 Geometric Mean Time Course of Change from Baseline of Anti-Tau IgM Titer by Treatment (Intention-to-Treat Population)			X
Figure 14.1.4.2 Individual Time Course of Anti-Tau IgM Titer (Intention-to-Treat Population)		X	X
Figure 14.1.4.3 Plot of Anti-Tau IgM Titer Response Rates by Visit and Treatment Group (Intention-to-Treat Population)		X	X
Figure 14.1.4.4 Box Plot of the Actual Values of the Anti-Tau IgM Titer by Visit and Treatment Group (Intention-to-Treat Population)			X
Figure 14.1.5.1 Geometric Mean Time Course of Anti-ePHF IgG Titer by Treatment Group (Intention-to-Treat Population)	Optional	X	X
Figure 14.1.5.1.1 Geometric Mean Time Course of Anti-ePHF IgG Titer by Treatment Group (Y-axis adjusted) (Intention-to-Treat Population)			X
Figure 14.1.5.1.2 Geometric Mean Time Course of Change from Baseline of Anti-ePHF IgG Titer by Treatment (Intention-to-Treat Population)			X

Strictly Confidential. © 2020, [redacted] All rights reserved.

MAIN Statistical Analysis Plan	Cohort 1 Interim Analysis (IA1)	Cohort 1 IA2 to IA7 Cohort 2 IA1 to IA6	Final Analysis
Figure 14.1.5.2 Individual Time Course of Anti-ePHF IgG Titer (Intention-to-Treat Population)	Optional	X	X
Figure 14.1.5.3 Plot of Anti-ePHF IgG Titer Response Rates by Visit and Treatment Group (Intention-to-Treat Population)	Optional	X	X
Figure 14.1.5.4 Box Plot of the Actual Values of the Anti-ePHF IgG Titer by Visit and Treatment Group (Intention-to-Treat Population)			X
Figure 14.1.6.1 Geometric Mean Time Course of Avidity Index on ePHF by Treatment Group (Intention-to-Treat Population)		X	X
Figure 14.1.6.1.1 Geometric Mean Time Course of Avidity Index on ePHF by Treatment Group (Y-axis adjusted) (Intention-to-Treat Population)			X
Figure 14.1.6.1.2 Geometric Mean Time Course of Change from Baseline of Avidity Index on ePHF by Treatment Group (Intention-to-Treat Population)			X
Figure 14.1.6.2 Individual Time Course of Avidity Index on ePHF (Intention-to-Treat Population)		X	X
Figure 14.1.7.1 Geometric Mean Time Course of Anti-T50 IgG Titer by Treatment Group (Intention-to-Treat Population)		X[3]	X
Figure 14.1.7.1.1 Geometric Mean Time Course of Anti-T50 IgG Titer by Treatment Group (Y-axis adjusted) (Intention-to-Treat Population)			X
Figure 14.1.7.1.2 Geometric Mean Time Course of Change from Baseline of Anti-T50 IgG Titer by Treatment Group (Intention-to-Treat Population)			X
Figure 14.1.7.2 Individual Time Course of Anti-T50 IgG Titer (Intention-to-Treat Population)		X[3]	X
Figure 14.1.7.3 Plot of Anti-T50 IgG Titer Response Rates by Visit and Treatment Group (Intention-to-Treat Population)		X[3]	X
Figure 14.1.8.1 Geometric Mean Time Course of Anti-CRM IgG Titer by Treatment Group (Intention-to-Treat Population)			X
Figure 14.1.8.1.1 Geometric Mean Time Course of Anti-CRM IgG Titer by Treatment Group (Y-axis adjusted) (Intention-to-Treat Population)			X
Figure 14.1.8.1.2 Geometric Mean Time Course of Change from Baseline of Anti-CRM IgG Titer by Treatment Group (Intention-to-Treat Population)			X
Figure 14.1.8.2 Individual Time Course of Anti-CRM IgG Titer (Intention-to-Treat Population)			X
Figure 14.1.8.3 Plot of Anti-CRM IgG Titer Response Rates by Visit and Treatment Group (Intention-to-Treat Population)			X
Figure 14.1.9.1 Geometric Mean Time Course of Anti-CpG IgG Titer by Treatment Group (Intention-to-Treat Population)	Optional	X	X
Figure 14.1.9.1.1 Geometric Mean Time Course of Anti-CpG IgG Titer by Treatment Group (Y-axis adjusted) (Intention-to-Treat Population)			X
Figure 14.1.9.1.2 Geometric Mean Time Course of Change from Baseline of Anti-CpG IgG Titer by Treatment (Intention-to-Treat Population)			X
Figure 14.1.9.2 Individual Time Course of Anti-CpG IgG Titer (Intention-to-Treat Population)	Optional	X	X

[3] Not applicable to Cohort 2 subjects.

Strictly Confidential. © 2020, [redacted] All rights reserved.

MAIN Statistical Analysis Plan	Cohort 1 Interim Analysis (IA1)	Cohort 1 IA2 to IA7 Cohort 2 IA1 to IA6	Final Analysis
Figure 14.1.9.3 Plot of Anti-CpG IgG Titer Response Rates by Visit and Treatment Group (Intention-to-Treat Population)	Optional	X	X
Figure 14.1.10.1 Geometric Mean (+/-95% Confidence Interval) of the Key Antibody Ratios over Time by Treatment Group (Intention-to-Treat Population)			X
Figure 14.1.10.2 Subjects Profile of the Change from Baseline of the log10 Values of the Key Antibody Ratios over Actual Time (Intention-to-Treat Population)			X
Figure 14.2.1 Mean Time Course of AD Biomarkers (in Plasma) by Treatment Group (Intention-to-Treat Population)		X	X
Figure 14.2.2 Mean Time Course of AD Biomarkers (in CSF) by Treatment Group (Intention-to-Treat Population)		X	X
Figure 14.2.3 Individual Value over Time of AD Biomarkers (in Plasma) (Intention-to-Treat Population)		X	X
Figure 14.2.4 Individual Value over Time of AD Biomarkers (in CSF) (Intention-to-Treat Population)		X	X
Figure 14.2.5 Mean Time Course of Change from Baseline AD Biomarkers (in Plasma) by Treatment Group (Intention-to-Treat Population)			X
Figure 14.2.6 Mean Time Course of Change from Baseline AD Biomarkers (in CSF) by Treatment Group (Intention-to-Treat Population)			X
Figure 14.2.7 Individual Change from Baseline over Time of AD Biomarkers (in Plasma) Intention-to-Treat Population)			X
Figure 14.2.8 Individual Change from Baseline over Time of AD Biomarkers (in CSF) (Intention-to-Treat Population)			X
Figure 14.2.9 Box Plot of the Actual Values of AD biomarkers (in Plasma) by Visit and Treatment Group (Intention-to-Treat Population)			X
Figure 14.2.10 Box Plot of the Actual Values of AD biomarkers (in CSF) by Visit and Treatment Group (Intention-to-Treat Population)			X
Figure 14.2.11 Box Plot of the Actual Log10-Transformed Values of AD biomarkers (in Plasma) by Visit and Treatment Group (Intention-to-Treat Population)			X
Figure 14.2.12 Box Plot of the Actual Log10-Transformed Values of AD biomarkers (in CSF) by Visit and Treatment Group (Intention-to-Treat Population)			X
Figure 14.2.13 Box Plot of the Fold-change from Baseline Values of AD biomarkers (in Plasma) by Visit and Treatment Group (Intention-to-Treat Population)			X
Figure 14.2.14 Box Plot of the Fold-change from Baseline Values of AD biomarkers (in CSF) by Visit and Treatment Group (Intention-to-Treat Population)			X
Figure 14.2.15 Box Plot of the Change from Baseline of the Log10-Transformed Values of AD biomarkers (in Plasma) by Visit and Treatment Group (Intention-to-Treat Population)			X
Figure 14.2.16 Box Plot of the Change from Baseline of the Log10-Transformed Values of AD biomarkers (in CSF) by Visit and Treatment Group (Intention-to-Treat Population)			X
Figure 14.3.1 Bar Chart of Most Frequent Treatment-Emergent Adverse Events (Safety Population)			X
Figure 14.3.2 Bar Chart of Time to first SAE (in days) by Cohort with Individual Points (Safety Population)			X
Figure 14.4.1 Box Plot of Hematology by Visit and Treatment Group (Intention-to-Treat Population)			X
Figure 14.4.2 Box Plot of Biochemistry by Visit and Treatment Group (Intention-to-Treat Population)			X

Strictly Confidential. © 2020, [redacted] All rights reserved.

MAIN Statistical Analysis Plan	Cohort 1 Interim Analysis (IA1)	Cohort 1 IA2 to IA7 Cohort 2 IA1 to IA6	Final Analysis
Figure 14.5.1 Box Plot of Vital Signs by Visit and Treatment Group (Intention-to-Treat Population)			X
Figure 14.5.2 MRI Volume Mean Change from Baseline by Brain Region by Treatment Group and Visit (Intention-to-Treat Population)			X
Figure 14.5.3 Individual Value over Time of MRI Volume by Brain Region (Intention-to-Treat Population)			X
Figure 14.5.4 Box Plot of MRI Volume by Brain Region by Visit and Treatment Group (Intention-to-Treat Population)			X
Listing 16.1.7 Randomization Assignments (All Randomized Subjects)			X
Listing 16.2.1.1 End of Study (Intention-to-Treat Population)			X
Listing 16.2.1.2 End of Investigational Product (IP) Treatment Phase (Intention-to-Treat Population)			X
Listing 16.2.2.1 Protocol Deviations (Intention-to-Treat Population)			X
Listing 16.2.2.2 COVID-19 Related Protocol Deviations (Intention-to-Treat Population)			X
Listing 16.2.3.1 Subject Analysis Sets (All Enrolled Subjects)			X
Listing 16.2.3.2 Subject Visits (Intention-to-Treat Population)			X
Listing 16.2.4.1 Demographic and Baseline Data (Intention-to-Treat Population)			X
Listing 16.2.4.2 Medical History (Intention-to-Treat Population)			X
Listing 16.2.4.3 Prior and Concomitant Medications (Safety Population)			X
Listing 16.2.4.4 Prior and Concomitant Procedures (Safety Population)			X
Listing 16.2.5.1 Administration of Investigational Product (Intention-to-Treat Population)			X
Listing 16.2.5.2 Treatment Compliance (Intention-to-Treat Population)			X
Listing 16.2.7.1 Adverse Events (Safety Population)			X
Listing 16.2.7.2 Serious Treatment-Emergent Adverse Events (Safety Population)			X
Listing 16.2.7.3 Treatment-Emergent Adverse Events Leading to Discontinuation of Study Treatment (Safety Population)			X
Listing 16.2.7.4 Treatment-Emergent Adverse Events Leading to Discontinuation from the Study (Safety Population)			X
Listing 16.2.7.5 Treatment-Emergent Adverse Events with Outcome of Death (Safety Population)			X
Listing 16.2.7.6.1 Injection Site Reaction Treatment-Emergent Adverse Events (Safety Population)			X
Listing 16.2.7.6.2 Injection Site Reaction Treatment-Emergent Adverse Events – Sub-components (Safety Population)			X
Listing 16.2.7.6.3 Presence or Absence of Injection Site Reactions by Visit at the Time of Injection Administration (Safety Population)			X

Strictly Confidential. © 2020, [redacted] All rights reserved.

MAIN Statistical Analysis Plan	Cohort 1 Interim Analysis (IA1)	Cohort 1 IA2 to IA7 Cohort 2 IA1 to IA6	Final Analysis
Listing 16.2.7.7.1 Magnetic Resonance Imaging (MRI) Results (Intention-to-Treat Population)			X
Listing 16.2.7.7.2 Volumetric Magnetic Resonance Imaging (MRI) Results (Intention-to-Treat Population)			X
Listing 16.2.7.8 Neuropsychiatric Inventory (NPI) Results (Intention-to-Treat Population)			X
Listing 16.2.7.9 Repeatable Battery for the Assessment of Neuropsychological Status (RBANS) Results (Intention-to-Treat Population)			X
Listing 16.2.7.10 Clinical Dementia Rating Scale Sum of Boxes (CDR-SB) and Global Score Results and Change from Baseline (Intention-to-Treat Population)			X
Listing 16.2.7.11 Mini Mental State Examination (MMSE) Results (Intention-to-Treat Population)			X
Listing 16.2.7.12 Columbia-Suicide Severity Rating Scale (C-SSRS) Results (Intention-to-Treat Population)			X
Listing 16.2.8.1 Hematology Results and Change from Baseline (Intention-to-Treat Population)			X
Listing 16.2.8.2 Biochemistry Results and Change from Baseline (Intention-to-Treat Population)			X
Listing 16.2.8.3 Urinalysis Results and Change from Baseline Intention-to-Treat Population)			X
Listing 16.2.8.4 Safety Parameters in Cerebrospinal Fluid (CSF) (Intention-to-Treat Population)			X
Listing 16.2.8.5 Pregnancy Test Results (Females of Childbearing Potential only) (Intention-to-Treat Population)			X
Listing 16.2.8.6 Additional Parameters Measured at Screening (Intention-to-Treat Population)			X
Listing 16.2.9 Vital Signs Results and Change from Baseline (Intention-to-Treat Population)			X
Listing 16.2.10.1 Centrally Read Electrocardiogram (ECG) Results – Interpretation (Intention-to-Treat Population)			X
Listing 16.2.10.2 Centrally Read Electrocardiogram (ECG) Individual Results and Change from Baseline (Intention-to-Treat Population)			X
Listing 16.2.11 Physical and Neurological Examination (Intention-to-Treat Population)			X
Listing 16.2.12.1 IgG and IgM Antibody Titers against Tau and pTau in Serum (Intention-to-Treat Population)	X	X	X
Listing 16.2.12.2 Anti-ePHF IgG, Anti-T50 IgG, Anti-CRM IgG, Anti-CpG IgG Titers and Avidity index on ePHF in Serum (Intention-to-Treat Population)	X	X	X
Listing 16.2.12.3 Peak and Area Under the Curve (AUC) of anti-pTau and anti-Tau IgG and IgM and anti-ePHF IgG in Serum (Intention-to-Treat Population)	X	X	X
Listing 16.2.12.4 Key Antibody Ratios Values and Change from Baseline (Intention-to-Treat Population)			X
Listing 16.2.13 Biomarkers Results and Change from Baseline (Intention-to-Treat Population)			X

Strictly Confidential. © 2020, [redacted] All rights reserved.

Data Safety Monitoring Board (DSMB) Table, Figure and Listing (TFL) Shells	Safety DSMB
Table 1 Subject Disposition	X
Table 2 Demographic Characteristics	X
Table 3 Summary of Treatment Duration	X
Table 4 Overall Summary of Treatment-Emergent Adverse Events	X
Table 4.1 Treatment-Emergent Adverse Events by System Organ Class, Preferred Term, and Maximum Severity	X
Table 4.2 Serious Treatment-Emergent Adverse Events by System Organ Class, Preferred Term, and Maximum Severity	X
Table 4.3 Severe Treatment-Emergent Adverse Events by System Organ Class and Preferred Term	X
Table 4.4 Serious Treatment-Emergent Adverse Events by System Organ Class, Preferred Term, and Maximum Severity	X
Table 4.5 Adverse Drug Reactions by System Organ Class and Preferred Term	X
Table 5 Summary of Cognitive Assessments by Visit	X
Table 6 Summary of Clinical Assessments by Visit	X
Table 7.x Summary of <Laboratory Parameter> by Visit	X
Table 7.5.1 Summary of Immunochemistry by Visit – Categorical Data	X
Figure 1.1 Box Plot of Vital Signs by Visit and Treatment – First 24 hours	X
Figure 1.2 Box Plot of Vital Signs by Visit and Treatment – After the First 24 hours	X
Figure 2.x Box Plot of <Lab Parameter> by Visit and Treatment	X
Listing 1 Treatment-Emergent Adverse Events	X
Listing 2 Serious Treatment-Emergent Adverse Events	X
Listing 3 Treatment-Emergent Adverse Events Leading to Discontinuation of Study Treatment	X
Listing 4 Treatment-Emergent Adverse Events Leading to Discontinuation from the Study	X
Listing 5 Treatment-Emergent Adverse Events with Outcome of Death	X
Listing 6 Injection Site Reactions Treatment-Emergent Adverse Events	X
Listing 7 Brain Magnetic Resonance Imaging (MRI) Results	X
Listing 8 Physical and Neurological Examination - New abnormalities	X
Listing 9 Abnormal ECG Results	X
Listing 10 Demographic and Baseline Characteristics	X

Strictly Confidential. © 2020, [redacted] All rights reserved.

**Data Safety Monitoring Board (DSMB) Table, Figure and Listing (TFL) Shells**	**Safety DSMB**
Listing 11 Vital Signs Results	X
Listing 12.x Abnormal Central Lab Blood Sample Results – [Hematology/Chemistry/Urinalysis/ANA and anti-dsDNA]	X
Listing 13 Local Lab CSF Cell Count Results	X
Listing 14 Subject Status and Exposure	X
Listing 15 Prior/Concomitant Medications	X
Listing 16 Medical History	X
Listing 17 Inflammatory Markers in Blood and CSF	X

Safety DSMB TFLs will be delivered at each IA and quarterly DSMB meeting.

Strictly Confidential. © 2020, [redacted] All rights reserved.

Appendix 3 Immunogenicity Testing for Cohort 1 and 2

**Type of endpoint**<br>**P: primary; S: secondary; E: exploratory**			**P**	**S**	**S**	**S**	**P**	**S**	**E**	**E**	**E**
**Cohort**	**IA#**	**Trigger**	**Anti-pTau IgG**	**Anti-Tau IgG**	**Anti-pTau IgM**	**Anti-Tau IgM**	**Anti-ePHF IgG**	**Avidity on ePHF**	**Anti-T50 IgG**	**Anti-CRM IgG**	**Anti-CpG IgG**
1	1	Sub-cohort 1.1 at Week 10	X		X		Optional[3]			N/A	Optional[3]
	2	Sub-cohort 1.1 at Week 50	X[1]	X[2]	X[2]	X[2]	X[1]	X[1]	X[2]	N/A	X[2]
	3	Sub-cohort 1.1 at Week 74	X[1]	X[2]	X[2]	X[2]	X[1]	X[1]	X[2]	N/A	X[2]
	4	Sub-cohort 1.2 at Week 10	X[1]	X[2]	X[2]	X[2]	X[1]	X[1]	X[2]	N/A	X[2]
	5	Sub-cohort 1.2 at Week 26	X[1]	X[2]	X[2]	X[2]	X[1]	X[1]	X[2]	N/A	X[2]
	6	Sub-cohort 1.2 at Week 50	X[1]	X[2]	X[2]	X[2]	X[1]	X[1]	X[2]	N/A	X[2]
	7	Sub-cohort 1.2 at Week 74	X[1]	X[2]	X[2]	X[2]	X[1]	X[1]	X[2]	N/A	X[2]
	8	Sub-cohort 1.2 Extension at Week 10	X[1]	X[2]	X[2]	X[2]	X[1]	X[1]	X[2]	N/A	X[2]
	9	Sub-cohort 1.2 Extension at Week 26	X[1]	X[2]	X[2]	X[2]	X[1]	X[1]	X[2]	N/A	X[2]
	10	Sub-cohort 1.2 Extension at Week 50	X[1]	X[2]	X[2]	X[2]	X[1]	X[1]	X[2]	N/A	X[2]
	11	Sub-cohort 1.2 Extension at Week 74	X[1]	X[2]	X[2]	X[2]	X[1]	X[1]	X[2]	N/A	X[2]
	12	Sub-cohort 1.3 at Week 10	X[1]	X[2]	X[2]	X[2]	X[1]	X[1]	X[2]	N/A	X[2]
	13	Sub-cohort 1.3 at Week 26	X[1]	X[2]	X[2]	X[2]	X[1]	X[1]	X[2]	N/A	X[2]
	14	Sub-cohort 1.3 at Week 50	X[1]	X[2]	X[2]	X[2]	X[1]	X[1]	X[2]	N/A	X[2]
	15	Sub-cohort 1.3 at Week 74	X[1]	X[2]	X[2]	X[2]	X[1]	X[1]	X[2]	N/A	X[2]
2	1	Sub-cohort 2.1 at Week 10	X[1]	X[2]	X[2]	X[2]	X[1]	X[1]	N/A	X[2]	X[2]
	2	Sub-cohort 2.1 at Week 26	X[1]	X[2]	X[2]	X[2]	X[1]	X[1]	N/A	X[2]	X[2]
	3	Sub-cohort 2.1 at Week 50	X[1]	X[2]	X[2]	X[2]	X[1]	X[1]	N/A	X[2]	X[2]
	4	Sub-cohort 2.1 at Week 74	X[1]	X[2]	X[2]	X[2]	X[1]	X[1]	N/A	X[2]	X[2]
	5	Sub-cohort 2.2 at Week 10	X[1]	X[2]	X[2]	X[2]	X[1]	X[1]	N/A	X[2]	X[2]
	6	Sub-cohort 2.2 at Week 26	X[1]	X[2]	X[2]	X[2]	X[1]	X[1]	N/A	X[2]	X[2]
	7	Sub-cohort 2.2 at Week 50	X[1]	X[2]	X[2]	X[2]	X[1]	X[1]	N/A	X[2]	X[2]
	8	Sub-cohort 2.2 at Week 74	X[1]	X[2]	X[2]	X[2]	X[1]	X[1]	N/A	X[2]	X[2]

Notes:
Considering the amount of immune response bioanalyses required by a single vendor [redacted] a 2-step data transfer may be performed. Timings as outlined in footnotes 1 and 2 below are proposed for split deliveries. This will be verified and detailed timelines will be produced for each IA. IAs may be merged if they are conducted in close proximity.

Strictly Confidential. © 2020, [redacted] All rights reserved.

[1] Transfer of anti-pTau IgG, anti-ePHF IgG and avidity on ePHF data. Assumes approximately 1 month from patient visit until data transferred to CRO
[2] Transfer of additional anti-T50 IgG, anti-CRM IgG, anti-pTau IgM and anti-Tau IgM. Assumes additional 1.5 month from first package delivery to full package delivered to CRO
[3] Optional means that the delivery will be done if the assay is fully validated and/or if the parameter is deemed necessary.

Strictly Confidential. © 2020, [redacted] All rights reserved.

Document History

Version Date	Modified/Reviewed By	Brief Summary of changes to current version
05Dec2023	[redacted]	Final version 4.0 – final clean version
04Dec2023	[redacted]	Final version 4.0 – figures updated, changes to summary statistics, minor clarifications added
11Oct2023	[redacted]	Draft Version 3.2 – updated with further Sponsor comments and include additional outputs.
04Aug2023	[redacted]	Draft Version 3.1 – updated with further Sponsor comments and according to the latest Protocol Amendment v7.0.
24Feb2021	[redacted]	Final Version 3.0 – updated with further Sponsor comments (JACI-35.054 dosage corrected).
23Feb2021	[redacted]	Draft Version 2.2 – updated with further Sponsor comments.
22Feb2021	[redacted]	Draft Version 2.1 includes changes following latest Protocol Amendment and COVID-19 pandemic impact.
26Nov2020	[redacted]	Final Version 2.0 created.
12Nov2020	[redacted]	Final Version 2.0 created – updated with further Sponsor comments available on 20Nov2020.
21Oct2020	[redacted]	Updated to include client comments on V1.1 (dated 14Oct2020).
14Oct2020	[redacted]	Created as Draft Version 1.1 incorporating changes due to protocol amendment and unscheduled visits mapping.
10Feb2020	[redacted]	Final Version 1.0 created.
08Feb2020	[redacted]	Created as Final Version 1.0; later updated to include TFLs on secondary and exploratory immune

Strictly Confidential. © 2020, [redacted] All rights reserved.

		response parameters.
27Jan2020	[redacted]	Updated to include client comments on V0.2 (dated 8Jan2020).
8Jan2020	[redacted]	Updated to include client comments on V0.1 (dated 22Nov2019).
22Nov2019	[redacted]	Initial version; internally and Study Matter Expert reviewed.

Strictly Confidential. © 2020, [redacted] All rights reserved.
