# Supplementary material for: Safety and immunogenicity of two Tau-targeting active immunotherapies, ACI-35.030 and JACI-35.054, in participants with early Alzheimer's disease: a phase 1b/2a, multicentre, double-blind, randomised, placebo-controlled study
Source: eBioMedicine. 2025 Sep 18;120:105940. doi: 10.1016/j.ebiom.2025.105940 (PMC12481106; doi:10.1016/j.ebiom.2025.105940)
Supplement: Author Statement ICJME Transfer Form [file mmc4.pdf]

THE LANCET

Authorisation to transfer author statement and ICMJE forms

Please insert the relevant text under the subheadings below. A completed form must be signed by the corresponding author. Please note that we will accept hand-signed and electronic (typewritten signatures). Please complete the form, scan and email to the handling Editor.

Article originally submitted to: The Lancet

Article now submitted to: eBioMedicine

Manuscript number at new journal: EBIOM-D-25-00907

Manuscript title: Safety and immunogenicity of two Tau-targeting active immunotherapies, ACI-35.030 and JACI-35.054, in participants with early Alzheimer's disease: a Phase 1b/2a, multicenter, double-blind, randomised, placebo-controlled study

Corresponding author: Olivier SOL

Article type: Randomised controlled trial

Olivier SOL Digitally signed by Olivier SOL Date: 2025.05.21 14:50:25 +02'00'

I ______________________________, the corresponding author of this manuscript confirm that all authors listed are aware that the manuscript is now being considered at eBioMedicine and have agreed to transfer the original author signature and ICMJE forms from The Lancet journal to which it was previously submitted. I (on behalf of all authors) agree that these signatures and statements are still accurate and applicable.
